# Targeting the TRIM14/USP14 axis enhances radiotherapy efficacy by inducing GPX4 degradation and disrupting ferroptotic defense in HCC

**DOI:** 10.1038/s41419-025-07807-6

**Published:** 2025-07-01

**Authors:** Xin Yue, Zhen Xiang, Yang Yi, Xuecen Wang, Weilin Zhou, Weijian Wu, Wenjing Qin, Yuxuan Zhao, Xianzhang Bu, Zhenwei Peng

**Affiliations:** 1 Department of Radiation Oncology, The First Affiliated Hospital, Sun Yat-sen University, Guangzhou, China; 2 State Key Laboratory of Bioactive Molecules and Druggability Assessment, The First Affiliated Hospital of Jinan University, Guangzhou, China; 3 State Key Laboratory of Anti-Infective Drug Discovery and Development, School of Pharmaceutical Sciences, Sun Yat-sen University, Guangzhou, China; 4 Laboratory of Chemical Oncogenomics, Guangdong Provincial Key Laboratory of Chemical Genomics, Peking University Shenzhen Graduate School, Shenzhen, China

**Keywords:** Translational research, Cancer therapy

## Abstract

Radiation resistance constitutes a formidable impediment in the treatment paradigm for hepatocellular carcinoma (HCC). Deubiquitinases (DUBs) exhibit notable efficacy in modulating cellular responses to stress and exogenous interventions, endowed with the critical trait of being targetable, thus facilitating the execution of precise therapeutic strategies. Here, we demonstrate that broad-spectrum inhibition of thiol hydrolase-type DUBs markedly augments radiotherapy sensitivity in HCC cells. Based on this, via CRISPR-based screening, we identified USP14 as the principal DUB orchestrating radioresistance. Ferroptosis emerged as a pivotal form of radiation-induced cell death, with our study singularly illustrating that USP14 is instrumental in directing cellular defenses against ferroptosis via the targeting and stabilization of glutathione peroxidase (GPX4). Mechanistically, we found that radiation triggers the assembly of Tripartite motif-containing protein 14 (TRIM14) at the GPX4 locus, subsequently recruiting USP14. The TRIM14/USP14 complex facilitates the excision of pronounced K48-linked polyubiquitination at lysine residues 48 or 118 on GPX4, thereby preserving GPX4’s structural integrity and antioxidative function to counteract ferroptosis. Intriguingly, TRIM14-mediated GPX4 stabilization is further amplified in radioresistant HCC, and subsequent radiation enables USP14-dependent blockade of GPX4 degradation. Consequently, pharmacological inhibition of USP14 substantially increases the susceptibility of HCC cells, thereby sensitizing patient-derived xenograft (PDX) tumors to radiotherapy. Concurrently, we explored the abscopal effect of radiotherapy and revealed that targeting USP14-enhanced ferroptosis augments antitumor immune responses post-radiation, suggesting a strategy to sustain therapeutic efficacy. In conclusion, our study uncovers the TRIM14/USP14 axis as a critical suppressor of radiation-induced ferroptosis and an actionable target to overcome radioresistance in HCC. These findings provide mechanistic insights and a translational framework for improving radiotherapy outcomes.

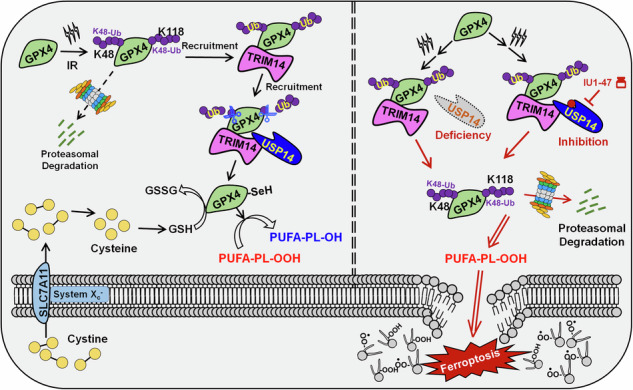

## Introduction

Constant advancements in technology have made adaptive radiotherapy (RT) the primary treatment approach for hepatocellular carcinoma (HCC) [1, 2]. This treatment strategy offers therapeutic benefits by minimizing damage to the non-tumor-bearing part of the liver, enabling delivery of ablative doses, and improving local control rates [3–5]. Nonetheless, the limited efficacy of RT significantly constrains its application for HCC. The principal reasons for the reduced effectiveness of RT are the considerable heterogeneity of hepatoma cells and the resistance of certain hepatoma cells to radiation [6, 7]. Consequently, an alternative strategy aimed at enhancing clinical outcomes is to sensitize HCC to RT within the confines of the standard radiation regimen. It is essential to deepen our understanding of the mechanisms driving radioresistance and to devise effective strategies for overcoming it to achieve this objective [8, 9].

Ubiquitin is a highly conserved peptide chain containing seven lysine residues (K) (sites-6, 11, 27, 29, 33, 48, and 63). E1 activating enzymes and E2 conjugating enzymes polymerize the ubiquitin chains to form different connecting types through different K sites, and the ubiquitin chains are attached to the target protein by E3 ligating enzymes. Different types of polyubiquitin chain-linking proteins form different protein skeletons and can recruit different functional molecules, thereby mediating different downstream functions of target proteins [10]. Deubiquitinases (DUBs) catalyze the removal of ubiquitin from substrates. As such, DUBs represent a significant class of molecular targets that impact the initiation, progression, and drug resistance of tumors by regulating processes related to protein degradation and function [11, 12]. Our preliminary work revealed that radioresistant HCC cells or PDXs exhibited increased deubiquitination activity. Thus, we hypothesize that certain DUBs may confer resistance to RT in HCC. To explore this hypothesis, we conducted CRISPR-based screening and validation of DUBs, identifying USP14 as a critical factor associated with radioresistance in HCC.

Ferroptosis is an iron-dependent type of regulated cell death (RCD) characterized by uncontrolled lipid peroxidation and subsequent plasma membrane rupture, recently identified as a significant contributor to radiation-induced cell death [13, 14]. During RT, X-rays or γ rays interact with cellular molecules such as water, causing energy transfer and ionization, which generates a substantial number of reactive oxygen species (ROS). Consequently, the ROS facilitates the electron transfer from ferrous iron (Fe^2+^) to generate peroxyl radicals, under which conditions lipids become highly prone to peroxidation [15–17]. This process eventually leads to the accumulation of lipid peroxides within cellular membranes, compromising membrane integrity and resulting in ferroptotic cell death [13, 18]. However, cancer cells have developed various strategies to resist ferroptotic death. Primarily, the solute carrier family 7 member 11 (SLC7A11), in concert with reduced glutathione (GSH) and glutathione peroxidase 4 (GPX4) (SLC7A11-GSH-GPX4 axis), serves as the main defense mechanism against ferroptosis. Recent studies have also identified the NAD(P)H, ferroptosis suppressor protein 1, and ubiquinone (NAD(P)H-FSP1-CoQ axis), as well as the tetrahydrobiopterin and guanosine triphosphate cyclohydrolase 1 (BH4-GCH1 axis), as alternative ferroptosis defense pathways, independent of GPX4 [19–21]. Noteworthy is the growing body of evidence suggesting that these defense mechanisms reduce the efficacy of radiation-induced lipid peroxidation and ferroptosis. This is evidenced by research showing that depletion or inhibition of SLC7A11 or GPX4 leads to a significant increase in radiosensitivity [12,22–24]. Collectively, targeting ferroptosis defense systems appears to be a viable approach to counteract tumor radioresistance [12]. In this study, we systematically confirmed that the mechanism by which USP14 mediates radioresistance is intricately connected to its role in orchestrating the timing of RT-induced ferroptosis, driven by RT.

GPX4 represents a central enzyme in the predominant defense mechanism against ferroptosis in cancer cells [25]. Despite this significance, the posttranslational regulatory mechanisms governing GPX4 remain inadequately understood. Our investigation identified GPX4 as a target for USP14-mediated deubiquitination following RT. Mechanistically, we revealed that RT triggers TRIM14 recruitment to GPX4, which in turn recruits USP14. The TRIM14/USP14 complex then removes K48-linked polyubiquitin chains from lysine 48 or 118 on GPX4, thereby preserving GPX4 stability and function. By sustaining GPX4’s reductive capacity, this mechanism establishes a continuous anti-ferroptotic defense, counteracting RT-induced lipid peroxidation accumulation and preventing the molecular prerequisites for ferroptosis. This process underlies radioresistance in HCC cells. Therefore, in exploring targeting therapeutic strategies, we thoroughly demonstrated that inhibiting USP14’s enzymatic activity is crucial for dismantling the GPX4-mediated ferroptosis defense, providing critical preclinical evidence for enhancing RT efficacy in clinical settings.

## Results

### Augmented deubiquitination activity correlates with increased radioresistance in HCC cells

**Fig. 1:**
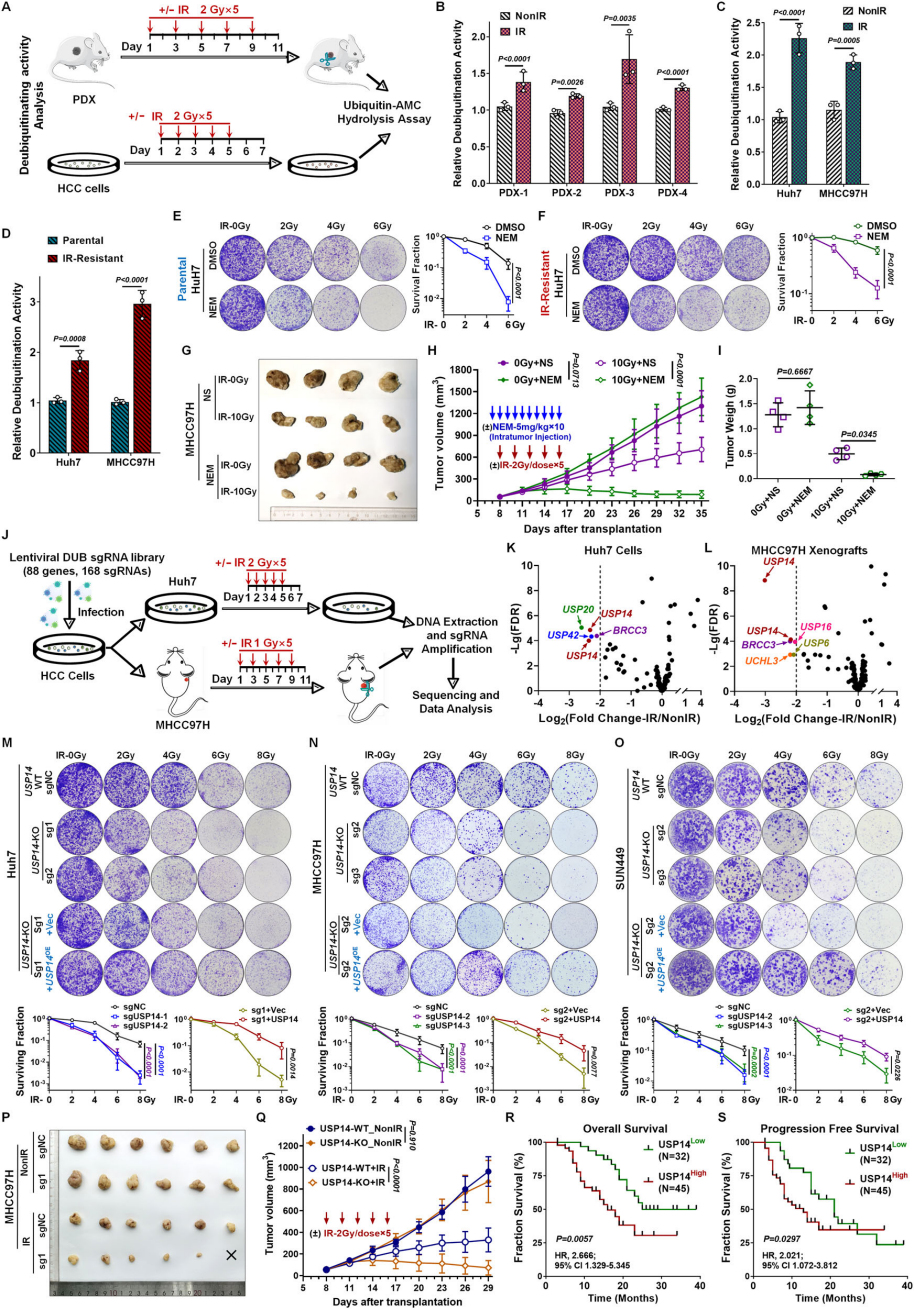
Identification of USP14 as a radioresistant DUB in HCC using a DUB-based CRISPR screen. **A** Schematic illustration of the experimental framework for analyzing deubiquitination activity in PDX models and HCC cells. Assessment of relative deubiquitinase (DUB) activity through ubiquitin-AMC hydrolysis assay in (**B**) PDX models, **C** Huh7 and MHCC97H cells, and **D** both parental and IR-resistant Huh7 and MHCC97H cells. DUB activity was adjusted to the respective control group. Non-IR indicates non-radiation; IR denotes ionizing radiation. Experiments were conducted in triplicate, with data presented as mean ± SD. Statistical significance was determined using a two-tailed Student’s *t*-test to compare the specified groups. Colony formation assays in (**E**) parental and (**F**) IR-resistant Huh7 cells treated with N-ethylmaleimide (NEM) for 12 h, followed by a single dose of 0, 2, 4, or 6 Gy IR. Representative images and survival curves are displayed. DMSO served as a vehicle control. Experiments were performed in triplicate, data presented as mean ± SD. Statistical significance was determined via two-way ANOVA. **G**–**I** MHCC97H xenografts were subjected to alternating treatments with NEM (5 mg/kg/day for 10 sessions) and IR (2 Gy/day for 5 sessions). **G** Representative tumor images. Quantitative assessment of (**H**) tumor volume and (**I**) weight. Experiments were triplicated, data shown as mean ± SD. Statistical significance determined by two-way ANOVA. **J** Treatment outline for DUB sgRNA library screening. Huh7 cells received continuous radiation at 2 Gy/dose × 5, while MHCC97H xenografts received interval radiation at 1 Gy/dose × 5. Relative DUB abundance from the CRISPR-Cas9 screen in (**K**) Huh7 cells and (**L**) nude mice with subcutaneous MHCC97H xenografts. DUBs with log_2_ (fold change) < −2 and a false discovery rate (FDR) < 0.01 were identified as radioresistance-related genes. Colony formation assays in (**M**) Huh7, (**N**) MHCC97H, and (**O**) SUN449 cells (including USP14-WT, knockout-KO, and KO + OE) groups with single doses of 0, 2, 4, 6, or 8 Gy IR. Representative images and survival curves are provided. Experiments run in triplicate, data as mean ± SD. Statistical significance assessed by two-way ANOVA. USP14-WT/KO MHCC97H xenografts exposed to IR (2 Gy/day for 5 sessions every alternate day). **P** Representative tumor images. **Q** Quantitative tumor volume analysis. Experiments duplicated in triplicate, data as mean ± SD. Statistical significance via two-way ANOVA. Kaplan–Meier analyses of (**R**) overall survival and (**S**) progression-free survival in HCC patients from SYSUFAH, stratified by USP14 immunoreactive scores (IRS). Results showcase three independent experiment representations.

Recent investigations have highlighted shifts in deubiquitination profiles as instrumental in the progression of radioresistance, thereby constituting a dynamic regulatory network that empowers cancer cells to endure external stress and sustain intracellular equilibrium. To elucidate the role of deubiquitination in HCC radioresistance, we established radioresistant HCC cell lines and PDX models. Utilizing these models, we assessed the activities of intracellular deubiquitinating enzymes post-radiation treatment by employing the substrate ubiquitin-7-amido-4-methylcoumarin (Ub-AMC) (Fig. 1A). Our findings revealed a substantial elevation in DUB activities in the cell lysates of radiation-treated PDXs (lines 1–4) and survival HCC cell lines (Huh7 and MHCC97H), compared to their untreated counterparts (Fig. 1B, C and Supplementary S1A, B). We subsequently scrutinized deubiquitinating enzyme activities in both parental and radioresistant HCC cells, specifically Huh7 and MHCC97H. An upsurge in activity was observed within the radioresistant HCC cells during intracellular deubiquitination events, as quantified by Ub-AMC assays (Figs. 1D and S1C, D). Given that most DUBs function as thiol hydrolases, we employed N-ethylmaleimide (NEM), a sulfhydryl-reactive agent, as a proof-of-concept inhibitor to investigate how thiol group blockade affects radiosensitivity and to evaluate the consequences of functional DUB inhibition. Initial colony formation assays demonstrated that NEM treatment markedly diminished the clonogenic capacity of both parental and radioresistant Huh7 cells following RT exposure (Fig. 1E, F). Subsequent in vivo studies involving nude mice with subcutaneous MHCC97H xenografts demonstrated that direct tumoral injection of NEM significantly heightened tumor radiosensitivity (Fig. 1G, H). Remarkedly, the addition of NEM enhanced the tumor inhibition rate post-IR treatment from 61.2 to 94.2% (Fig. 1I). Importantly, control experiments ruled out significant contributions from GSH/TrxR pathway modulation or general ubiquitination machinery inhibition to NEM’s radiosensitizing effects (Fig. S2). Collectively, these findings demonstrate that radiation considerably elevates intracellular DUB activity in HCC and that inhibiting DUBs can effectively counteract radioresistance. Consequently, these findings underscore the need to identify the specific DUBs mediating this radioprotective mechanism.

### Analysis and identification of USP14 as a key DUB involved in HCC radioresistance

To isolate a deubiquitinating enzyme linked to radioresistance, we employed a CRISPR library targeting DUBs to generate Cas9/sgRNA-expressing HCC cells. Our positive screening process uncovered specific DUB sgRNAs that enhance radiosensitization when inhibited. Subsequently, Huh7 cells were subjected to continuous radiation exposure, a treatment that elicited minimal cytotoxic effects in wild-type (WT) cells. Likewise, MHCC97H xenografts experienced intermittent radiation (Fig. 1J). The surviving cells or residual tumor tissues, along with their respective untreated control samples, were then subjected to deep sequencing to evaluate the presence of DUB sgRNAs. The sgRNAs of the top candidate DUBs were ranked based on the read number ratio of the RT-treated group to the untreated group (Log_2_ FC < −2; FDR < 0.01). Among them, USP14, USP20, and BRCC3 met these criteria (Fig. 1K, L and Tables S1 and S2). To determine the impact of their knockout (KO) on HCC cell radiosensitivity, we conducted a colony formation assay for validation. The results demonstrated that USP14-KO had the most pronounced effect (Fig. S3A). Notably, USP14 stood out among all candidates, with its sgRNAs meeting the screening criteria in both Huh7 cells and MHCC97H xenografts. Furthermore, we investigated whether USP14-KO enhances HCC cell radiosensitivity. We observed that at least two selected sgRNAs effectively reduced USP14 expression in three distinct HCC cell lines (Fig. S3B). An in vitro clonogenic assay showed that USP14-KO significantly impaired colony formation in three HCC cell lines (Huh7, MHCC97H, SNU449) following RT treatment (Fig. 1M–O). Crucially, reintroducing USP14 expression in USP14-KO HCC cells resulted in increased resistance to RT (Fig. 1M–O). Moreover, to explore USP14’s role in the RT response in vivo, we created a xenograft model of HCC in mice using the MHCC97H cell line. RT treatment significantly augmented tumor regression, as evidenced by reduced tumor volume in the USP14-KO group compared to control cells (Fig. 1P, Q). Furthermore, the tumor inhibition rate, indicated by tumor weight, was markedly higher in the USP14-KO group (94.9%) compared to the USP14-WT group (72.5%) (Fig. S3C). Histopathological analysis revealed that after RT treatment, the reduction in tumor cells was substantially greater in USP14-KO xenografts than in USP14-WT xenografts. Additionally, the presence of Ki67 was significantly reduced in USP14-KO xenografts relative to USP14-WT counterparts (Fig. S3D). Together, these results demonstrate that USP14 downregulation enhances the susceptibility of HCC cells to RT.

To understand the relationship between intertumoral USP14 levels and RT effectiveness in HCC patients, we performed an IHC assay to assess the immunoreactive scores (IRS) of 77 pre-treatment tumor biopsies, clinically annotated from patients scheduled for RT. Kaplan-Meier survival analysis indicated that elevated USP14 expression was significantly associated with reduced overall survival (OS, *P* = 0.0057, Fig. 1R) and shorter progression-free survival (PFS, *P* = 0.0297, Fig. 1S) among HCC patients treated with radiation. Collectively, these findings suggest that USP14 may be a critical factor influencing radioresistance in HCC.

### Inhibition of USP14’s deubiquitination activity enhances the radiosensitivity of HCC cells

**Fig. 2:**
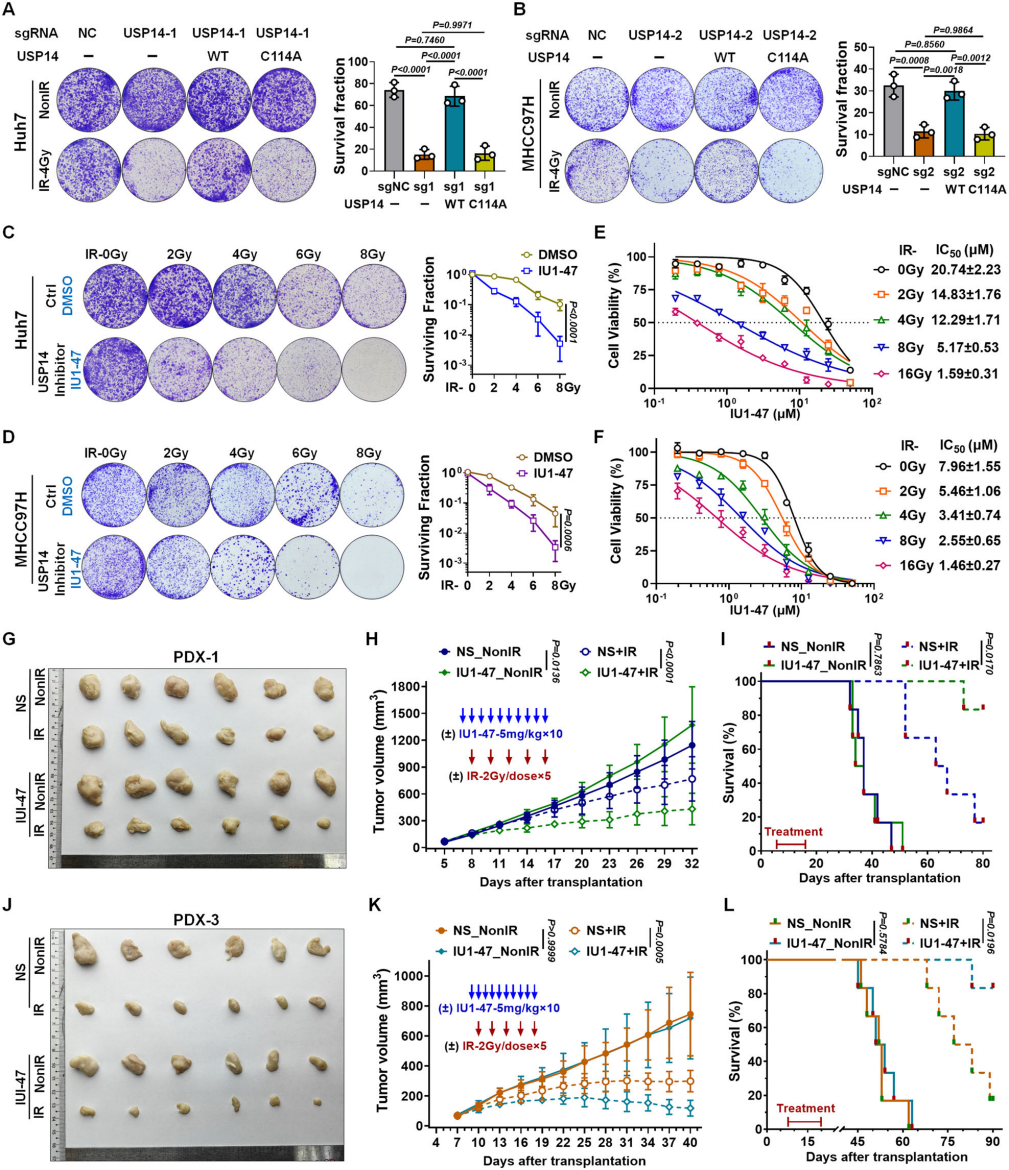
Inhibition of USP14 deubiquitinating activity sensitizes HCC cells and PDX models to radiation. Colony formation assays were conducted on (**A**) USP14-KO Huh7 and (**B**) USP14-KO MHCC97H cells overexpressing either WT or C114A mutant USP14. Cells were subjected to 4 Gy IR or left untreated. Representative images and survival curves are provided. Experiments were conducted in triplicate, and data are expressed as mean ± SD. Statistical significance was assessed using one-way ANOVA for group comparisons. Colony formation assays in (**C**) Huh7 and (**D**) MHCC97H cells pre-treated with the USP14 inhibitor IU1-47 (5 μM; DMSO served as a vehicle control) for 12 h before IR exposure (0, 2, 4, 6, or 8 Gy). Representative images and survival curves are provided. Experiments were conducted in triplicate, data presented as mean ± SD. Statistical significance was evaluated by two-way ANOVA. The impact of IU1-47 on the proliferation of Huh7 (**E**) and MHCC97H (**F**) cells following IR treatment (0, 2, 4, 6, or 8 Gy). Experiments were carried out in triplicate and data are presented as mean ± SD. The IC_50_ was calculated by normalizing cell viability to the radiation-only control group (without IU1-47), which was set as 100% survival. **G–L** Effect of IU1-47 combined with IR in PDX models. (**G**) PDX-1 and (**J**) PDX-3 models received IU1-47 (5 mg/kg/day for 10 sessions) and IR (2 Gy/day for 5 sessions every alternate day). **G**, **J** Representative tumor images. **H**, **K** Quantitative tumor volume analysis. **I**, **L** Kaplan–Meier survival analysis of mice bearing PDX (*n* = 6/group). Experiments were replicated in triplicate, and data are shown as mean ± SD. Statistical significance determined by two-way ANOVA. Results are exemplary of three independent studies.

To elucidate whether USP14 enhances radiation-induced deubiquitinating activity within cells, we conducted a Ub-AMC hydrolysis assay. Our results indicated a notable increase in USP14 activity in radiation-exposed HCC cells compared to untreated controls (Fig. S4A), implying that USP14’s deubiquitinating function may be necessary for its role in promoting radioresistance. Consequently, we introduced either an empty vector, WT-USP14, or a catalytically inert USP14 mutant (C114A) into USP14-KO HCC cells to evaluate the impact of USP14 restoration. The colony formation assays revealed that only the reintroduction of WT-USP14 could restore radioresistance in USP14-KO cells, as opposed to the mutant USP14 (C114A) (Fig. 2A, B).

The selective USP14 inhibitor IU1-47 serves as a valuable tool for examining the impact of USP14 enzymatic inhibition pharmacologically both in vitro and in vivo [26]. Clonogenic assays indicated that IU1-47 treatment markedly impaired colony formation in HCC cells following RT (Fig. 2C, D). Additionally, proliferation assays suggested that the inhibitory effect of IU1-47 on HCC cell viability increased in conjunction with the administered radiation dose (Fig. 2E, F), suggesting that RT treatment amplifies IU1-47 sensitivity and that the combination of IU1-47 with RT yields a synergistic effect. Furthermore, to elucidate the critical role of USP14 in modulating HCC cell sensitivity to IR in the context of IU1-47 treatment, cells expressing either USP14-KO or the enzymatically inactive C114A mutation of USP14 were subjected to RT and subsequent IU1-47 treatment. The findings revealed that IU1-47 was unable to sensitize USP14-KO or C114A-mutant cells to RT (Fig. S4B–E). Collectively, these data affirm that the anticancer potency of IU1-47 in combination with RT is significantly dependent on active USP14. To explore IU1-47’s influence on radiosensitivity in vivo, we conducted therapeutic experiments on NSG mice with subcutaneous PDX tumors. IU1-47 treatment significantly increased the radiosensitivity of otherwise radioresistant PDXs (Fig. 2G, H). Specifically, IU1-47 enhanced the tumor growth inhibition rate from 16.4% to 84.8% following RT, demonstrating a pronounced synergistic effect with RT (Fig. S5A). Furthermore, IU1-47 elicited a modest yet statistically significant increase in radiosensitivity in intrinsically radiosensitive PDXs (Fig. 2J, K), improving the tumor growth inhibition rate post-RT from 82.4 to 96.0% (Fig. S5B). No body weight loss or visible adverse health effects were noted in mice receiving combined IU1-47 and RT treatment (data not shown). The combined use of IU1-47 and RT in treating radioresistant or radiosensitive PDXs substantially enhanced survival rates and extended survival duration compared to RT treatment alone (Fig. 2I, L). Histopathological analysis revealed that the dual treatment markedly increased tumor cell apoptosis and reduced the proliferation marker Ki67 compared to RT only (Fig. S5C, D), indicating that IU1-47 distinctly amplifies the anti-proliferative effects of RT on tumors. These results suggest that USP14-promoted radioresistance is contingent upon its deubiquitinating activity, with IU1-47 effectively enhancing HCC’s susceptibility to RT.

### Targeting and inhibition of USP14 enhances ferroptosis induced by RT

**Fig. 3:**
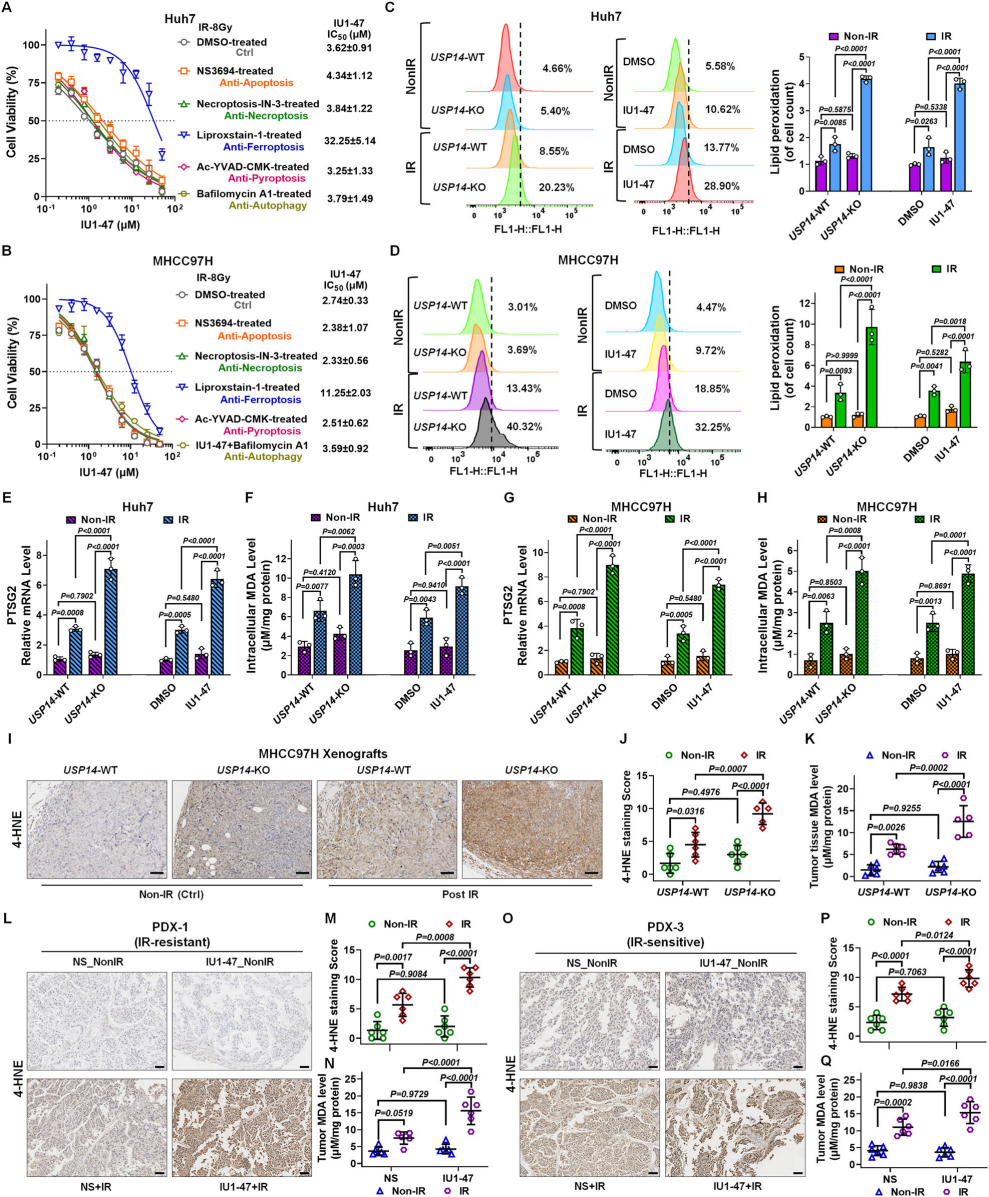
USP14 deficiency or inhibition enhances radiation-induced ferroptosis. Cell viability profiles in (**A**) Huh7 and (**B**) MHCC97H cells treated with IU1-47 at designated concentrations, in conjunction with NS3694, Necroptosis-IN-3, Liproxstatin-1, Ac-YVAD-CMK, or Bafilomycin A1 (each at 5 μM, DMSO as a control) for a duration of 12 h, followed by an 8 Gy IR exposure. Viability was assessed 72 h post-radiation. The IC_50_ was calculated by normalizing cell viability to the drug-pretreated and IR control group (without IU1-47), which was set as 100% survival. **C**, **D** Quantification of lipid peroxidation through BODIPY-581/591-C11 staining in (Left) USP14-knockout (KO)/wild-type (WT) Huh7 and MHCC97H cells treated with or without IR, (Middle) Huh7 cells pretreated with IU1-47 (5 μM) for 12 h prior to IR, and (Right) these previously mentioned groups. **E**, **G** Relative mRNA expression levels of PTSG2 and **F**, **H** measurements of intracellular malondialdehyde (MDA) in USP14-KO/WT cells exposed to non-irradiated (Non-IR) or irradiated conditions, or cells pretreated with IU1-47 (5 μM) for 12 h prior to IR in (**E**, **F**) Huh7 cells and (**G**, **H**) MHCC97H cells. Samples were collected 12 h following IR. In the context of USP14-WT/KO Huh7 xenografts treated with or without IR: **I** immunohistochemical (IHC) staining for 4-hydroxynonenal (4-HNE), **J** 4-HNE staining assessment, and **K** analysis of tumor tissue MDA levels. Scale bars: 10 μm. In the IR-resistant PDX-1 model receiving combined IU1-47 and IR treatment: **L** 4-HNE IHC staining, **M** scoring of 4-HNE staining, and **N** determination of tumor tissue MDA levels. Scale bars: 5 μm. **O–Q** In the IR-sensitive PDX-3 model undergoing combined treatment with IU1-47 and IR: (**L**) 4-HNE IHC staining, (**M**) evaluation of 4-HNE staining score, and (**Q**) quantification of tumor tissue MDA levels. Scale bars: 5 μm. Findings are indicative of three independent experiments and are expressed as mean ± SD. Bar graphs utilize one-way ANOVA for comparative analysis of specified groups.

Given that DNA double-strand breaks (DSBs) are the most deleterious lesions caused by radiation and are the primary source of its antitumor effects, our initial investigation focused on USP14’s potential impact on DNA DSB damage or repair mechanisms. We discovered that neither deletion nor suppression of USP14 affected homologous recombination (HR) or non-homologous end-joining (NHEJ) pathways in HCC cells, as evidenced by the DR-GFP and EJ5-GFP reporter assays, respectively (Fig. S6A). Concurrently, we noted a post-irradiation translocation of USP14 from the nucleus to the cytoplasm and cell membrane (Fig. S6B). Considering USP14’s role as a proteasome-associated deubiquitination enzyme, we ruled out its function in proteasome regulation by confirming unchanged proteasome activity (Fig. S6C). These observations led us to hypothesize that USP14 predominantly operates outside the nucleus, potentially playing a role in RCD. Subsequent investigations into how USP14 influences RT-induced RCD revealed that among various forms of RCD, inhibition of ferroptosis significantly counteracted the radiosensitizing effect of the USP14 inhibitor IU1-47 (Figs. 3A, B and S6D, E). Thus, it appears that USP14 contributes to HCC radioresistance primarily through the suppression of ferroptosis triggered by IR.

Our investigation further addressed the involvement of USP14 in ferroptosis triggered by RT. Considering that ferroptosis is driven by phospholipid peroxidation, a process signified by lipid peroxidation [27], we employed BODIPY-581/591-C11 staining to assess the levels of lipid peroxidation in HCC cells. The data indicated that both the loss and inhibition of USP14 significantly amplified RT-induced lipid peroxidation (Fig. 3C, D), upregulated the ferroptosis marker gene PTGS2 (Fig. 3E, G), and increased the accumulation of malondialdehyde (MDA), a byproduct of lipid peroxidation [28] (Fig. 3F, H). Additionally, 4-hydroxy-2-noneal (4-HNE) staining, an alternate marker for lipid peroxidation, revealed a conspicuous rise in 4-HNE levels in vivo within MHCC97H xenografts and PDXs due to the deletion or inhibition of USP14 post-RT treatment, relative to the RT-only group (Fig. 3I, J–M, O, P). Further post-RT analysis showed a significant elevation of MDA in tumors devoid of USP14 compared to WT counterparts (Fig. 3K); similarly, in PDXs treated with IU1-47 in combination with RT exhibited a more pronounced increase in MDA levels than the RT-alone treatment group (Fig. 3N, Q). Collectively, our findings propose that the inhibition or deletion of USP14 is associated with an increase in ferroptosis following RT in HCC cells.

### USP14 modulates cellular defense mechanisms against ferroptosis via GPX4 following RT

**Fig. 4:**
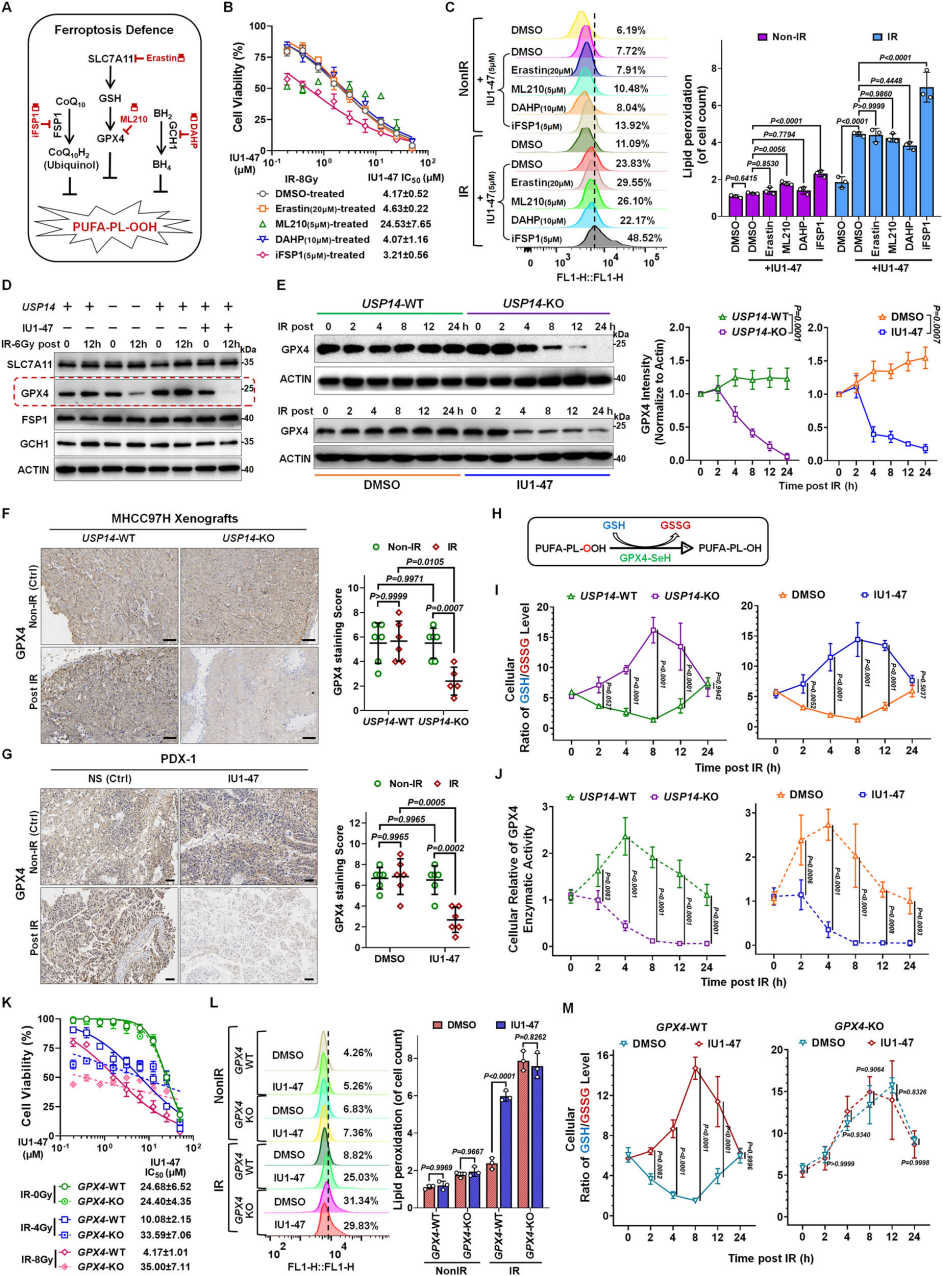
USP14 modulates defense mechanisms against radiation-induced ferroptosis via GPX4. **A** Diagrammatic representation of the ferroptosis defense pathways and associated inhibitors explored in this study. **B** Evaluation of IU1-47 on inhibiting Huh7 cell proliferation in conjunction with Erastin (20 μM), ML210 (5 μM), DAHP (10 μM), or iFSP1 (5 μM) for 12 h, followed by 8 Gy IR. Viability was assessed 72 h post-IR. The IC_50_ was calculated by normalizing cell viability to the drug-pretreated and IR control group (without IU1-47), which was set as 100% survival. **C** Lipid peroxidation levels were quantified by BODIPY-581/591-C11 staining in Huh7 cells pretreated with IU1-47 (5 μM) alongside Erastin (20 μM), ML210 (5 μM), DAHP (10 μM), or iFSP1 (5 μM) for 12 h prior to IR exposure. Lipid peroxidation levels in these groups are also shown. **D** Western Blot analysis for proteins SLC7A11, GPX4, FSP1, and GCH1 in Huh7 cells with/without USP14 expression 12 h after 6 Gy IR; and after treatment with IU1-47 (5 μM) for 2 h (DMSO as control) followed by 6 Gy IR 12 h later. Non-IR groups acted as controls. Actin was used as a loading control. **E** Detection of GPX4 via WB assay at the indicated time points in USP14-WT or USP14-KO Huh7 cells exposed to 6 Gy IR, or in parental Huh7 cells post IU1-47 treatment for 2 h (DMSO as control). Left: representative images. Right: normalized GPX4 grayscale intensity against GAPDH at specified times post IR. **F**, **G** GPX4 levels in PDX models. **F** USP14-WT/KO Huh7 xenografts and **G** PDX-1 model treated with IU1-47 combined with IR. Left: GPX4 IHC staining, Right: GPX4 staining score. Normal saline (NS) served as a control. Scale bars: 10 μm in (**F**) and 5 μm in (**G**). **H** Schematic illustration of GPX4’s function in this study. **I** GSH/GSSG ratio in USP14-WT/USP14-KO Huh7 cells at the indicated time points post IR (6 Gy) or cells treated with IU1-47 (5 μM) for 2 h before 6 Gy IR. DMSO was the control. **J** GPX4 activity detection in USP14-WT/USP14-KO Huh7 cells at the indicated time points post IR (6 Gy) or cells treated with IU1-47 (5 μM) for 2 h before 6 Gy IR. DMSO was the control. **K** Cell viability using the CCK-8 assay in GPX4-WT/GPX4-KO Huh7 cells following IU1-47 treatment and varying IR doses (0/4/8 Gy). DMSO served as vehicle control. Cells were collected 72 h post IR. The IC_50_ was calculated by normalizing cell viability to the drug-pretreated and IR control group or drug-pretreated control group (without IU1-47), which was set as 100% survival. **L** Quantification of lipid peroxidation levels by BODIPY-581/591-C11 staining in GPX4-WT/GPX4-KO Huh7 cells pretreated with IU1-47 (5 μM) for 2 h prior to IR, collected 12 h post. Lipid peroxidation levels in these groups are shown. **M** GSH/GSSG ratio in GPX4-WT/GPX4-KO Huh7 cells pretreated with IU1-47 (5 μM) for 2 h before 6 Gy IR exposure. Cells were collected at the indicated time points post IR. Results are indicative of three independent experiments, expressed as mean ± SD. For (**C**, **F**, **G**)-Right and (**I**–**M**), one-way ANOVA was employed for comparisons among indicated groups. For (**E**)-Right, statistical significance was determined by two-way ANOVA.

To elucidate how USP14 modulates resistance to ferroptosis induced by RT, we initially evaluated a range of ferroptosis inhibitors to establish the relationship between USP14 and ferroptosis. In vitro proliferation assays indicated that the enhanced cytotoxicity of IU1-47 following RT could be significantly counteracted by inhibitors targeting ACAC, ACSL4, lipoxygenases, antioxidants, or iron chelation (Fig. S7A, B). Notably, these agents disrupt the initial phase of lipid peroxidation. Thus, we propose that USP14’s potential role is in bolstering cellular defenses against ferroptosis rather than directly instigating lipid peroxidation due to radiation. Primary defenses against ferroptosis are conveyed through the SLC7A11-GSH-GPX4 pathway [25]; nonetheless, additional routes such as FSP1-CoQ_10_ and GCH1-BH_4_ also contribute to mitigating ferroptosis [29, 30]. The blockade of these protective mechanisms by ferroptosis inducers (FINs) precipitates a swift accumulation of lipid peroxides, culminating in ferroptosis [20] (Fig. 4A). Our pharmacological studies demonstrate that GPX4 inhibitors (ML210, RSL3, and FINO2) completely abolished both the radiosensitizing effects of IU1-47 and its induction of lipid peroxidation (LPO) accumulation following radiation (Figs. 4B, C and S7C–F). In contrast, SLC7A11 inhibition with Erastin exhibited concentration-dependent effects: while lower doses (20 μM) showed no interaction with IU1-47, higher effective doses (40 μM) produced additive effects (Figs. 4B, C and S7C, D-2G), confirming that USP14 functions downstream of SLC7A11. Notably, IU1-47 showed synergistic effects with the FSP1 inhibitor iFSP1 similar to those observed with high-dose Erastin (Figs. 4B, C and S7C), suggesting that USP14 likely does not act through the FSP1-CoQ_10_ pathway. Furthermore, GCH1 inhibition using DAHP (up to 80 μM) neither induced LPO alone nor showed synergy with IU1-47, effectively excluding involvement of the BH4 pathway (Figs. 4B, C and S7C, H). These comprehensive pharmacological analyses provide definitive evidence that USP14 specifically regulates the GPX4-mediated ferroptosis defense axis, with no detectable involvement of either FSP1- or GCH1-dependent protective pathways.

Considering the potential for USP14, through its deubiquitinating activity, to modulate the ubiquitin-mediated turnover of proteins to maintain their stability during RT treatment, we assessed RT’s impact on ferroptosis-related protein expression in the context of USP14 deficiency or inhibition. Indeed, these conditions resulted in a notable reduction in GPX4 levels, a key player in ferroptosis defense, while the abundance of other associated proteins remained unchanged (Figs. 4D and S8A). To substantiate these findings, we measured the GPX4 protein decay rate following RT and observed a significantly reduced half-life of GPX4 under both USP14 depletion and inhibition (Fig. 4E). Furthermore, immunostaining of tumor samples, including MHCC97H xenografts and PDXs, showed a considerable decrease in GPX4 expression following IR treatment in cases where USP14 was deleted or inhibited in vivo (Fig. 4F, G). Notably, IR alone fails to reduce GPX4 in any HCC line examined (New Fig. S8B), confirming USP14’s constitutive protective role. In USP14-deficient systems, we observed that IR serves as the critical trigger for GPX4 degradation—an effect strictly dependent on radiation exposure, as no GPX4 loss occurred in USP14-knockdown cells without irradiation (Fig. S8C, D). Intriguingly, while GPX4 mRNA displayed only transient suppression (with partial recovery) under normal conditions, USP14 inhibition triggered a paradoxical early upregulation of GPX4 transcript levels before returning to baseline by 24 h (Fig. S8E). We interpret this biphasic transcriptional response as a compensatory feedback mechanism activated by acute GPX4 protein degradation.

The obtained data elucidates a mechanism by which USP14 modulates GPX4 stability after RT treatment. Consequently, we examined USP14’s influence on GPX4 enzymatic activity. Given that extracellular cystine is imported via SLC7A11 for GSH biosynthesis, with GSH being essential for GPX4 to detoxify lipid hydroperoxides into lipid alcohols, the conversion rates of GSH to glutathione disulfide (GSSG) are critical for assessing intracellular GPX4 activity (Fig. 4H). Using GSH detection assays, we characterized the temporal redox dynamics in response to irradiation (IR). In USP14-proficient cells, IR triggered progressive oxidation of glutathione (GSH → GSSG conversion) over 0–8 h, followed by recovery to baseline levels by 24 h. Strikingly, USP14 inhibition or deletion abolished this oxidative shift, instead promoting an increase in the GSH/GSSG ratio post-IR (Figs. 4I and S9A), indicative of preserved reductive capacity. Complementary time-course analysis revealed that GPX4 enzymatic activity in USP14-proficient cells displayed a biphasic response to radiation: an initial compensatory increase followed by a gradual decline. In contrast, USP14-deficient or pharmacologically inhibited cells exhibited rapid and near-complete exhaustion of GPX4 activity within 8 h post-IR (Fig. 4J). These findings demonstrate that USP14 critically sustains GPX4 function, thereby enabling GSH-dependent peroxide detoxification and redox homeostasis following radiation stress.

Our findings reveal a critical functional interplay between USP14 inhibition (via IU1-47) and GPX4-mediated ferroptosis protection. Proliferation assays demonstrated that GPX4 depletion completely abolished the radiosensitizing effects of IU1-47 (Fig. 4K). This observation was further corroborated by lipid peroxidation assays, which revealed that GPX4 KO similarly negated IU1-47-induced lipid peroxide accumulation following IR treatment (Fig. 4L). These complementary findings establish GPX4 as an essential mediator of IU1-47’s pro-ferroptotic activity. To mechanistically dissect this dependency, we performed complementary pharmacological and genetic experiments: (1) GPX4 inhibitors (RSL3/FINO2) recapitulated IU1-47’s redox effects, inducing early elevation of the GSH/GSSG ratio followed by eventual equilibration (Fig. S9B). (2) In GPX4-KO cells, IR failed to induce GSH oxidation, and IU1-47 lost its pro-ferroptotic efficacy (Figs. 4M and S9C), confirming GPX4 as the non-redundant downstream effector of USP14. Together, these results establish that USP14 sustains post-IR redox homeostasis by preserving GPX4 function. Disruption of USP14 activity-whether through pharmacological inhibition or genetic deletion—compromises GPX4-dependent GSH cycling, leading to dysregulated GSH/GSSG ratios that ultimately prime cells for ferroptotic death. To mechanistically validate the USP14-GPX4 axis, we performed complementary genetic experiments: GPX4 knockdown in USP14-overexpressing cells dramatically enhanced radiosensitivity, completely abolishing the protective effect of USP14 overexpression (Fig. S10A, C). Conversely, ectopic GPX4 expression in USP14-KO cells significantly restored radiation resistance. Mechanistically, we found that USP14-mediated degradation was insufficient to counteract the high levels of exogenous GPX4 (Fig. S10B, D). These genetic epistasis experiments demonstrate that GPX4 is both necessary and sufficient to mediate USP14’s radioprotective function, operating as its essential downstream effector.

### USP14 cleaves K48-linked ubiquitination of GPX4 at either K48 or K118 following RT

**Fig. 5:**
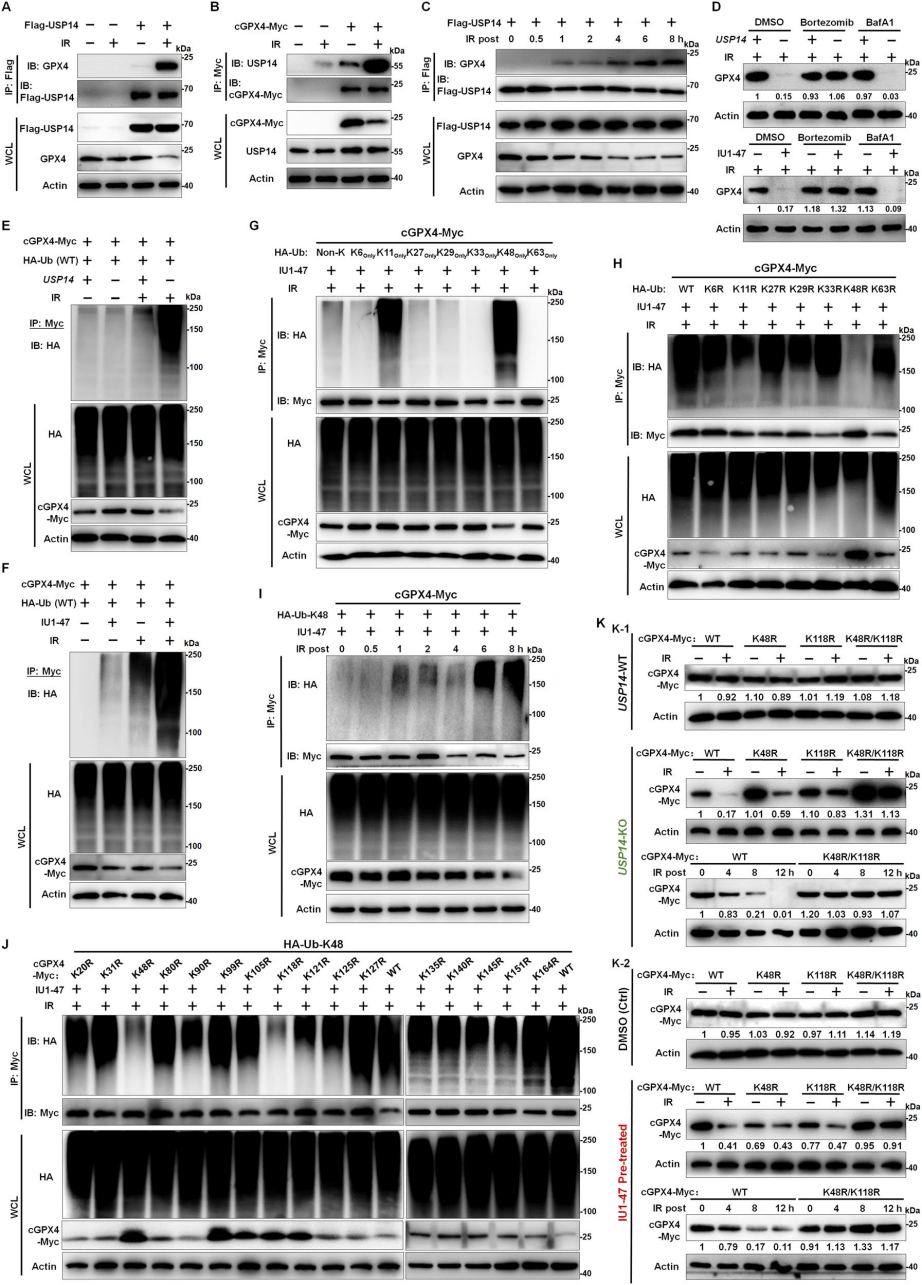
Interaction and deubiquitination of GPX4 by USP14 in response to IR. **A** Co-immunoprecipitation (Co-IP) and western blot (WB) analyses were conducted to explore the interaction between Flag-tagged USP14 and GPX4 post-6 Gy ionizing radiation (IR). Cell lysates were collected 8 h following IR, with untreated cells serving as controls. **B** The association between Myc-tagged cGPX4 and USP14 post-6 Gy IR was assessed utilizing Co-IP and WB. Cells were harvested 8 h post-IR, with non-irradiated samples as controls. **C** Co-IP and WB were conducted to evaluate the temporal interaction between GPX4 and Flag-USP14 following 6 Gy IR treatment, with samples collected at designated post-IR intervals. **D** GPX4 protein levels were examined in USP14-KO Huh7 cells pre-treated with Bortezomib (100 nM) alone or in combination with Bafilomycin A1 (250 nM) for 3 h preceding 6 Gy IR. Samples were harvested for 8 h post-IR, with non-irradiated cells as controls. **E** Co-IP and WB were used to determine the ubiquitination levels of GPX4 in USP14-KO Huh7 cells expressing Myc-tagged cGPX4 and wild-type (WT) HA-ubiquitin, following 6 Gy IR exposure. Cells were collected 8 h post-IR, with untreated samples as controls. **F** The ubiquitination of GPX4 was assessed in Huh7 cells expressing Myc-tagged cGPX4 and WT HA-ubiquitin, pre-treated with IU1-47 (5 μM) or DMSO as a control for 2 h prior to 6 Gy IR. Cell lysates were harvested 8 h post-IR, with non-irradiated samples serving as controls. The ubiquitin linkage type on GPX4 was identified using Co-IP and WB. In conditions (**G**) and (**H**), Huh7 cells expressed HA-ubiquitin mutants with selective lysine preservation (K-only, with other lysines mutated to arginine) or a singular lysine-to-arginine mutation, respectively. Cells were treated with IU1-47 or DMSO as a control for 2 h before 6 Gy IR and harvested 8 h later. **I** The kinetics of IU1-47-induced K48-linked ubiquitination of GPX4 were evaluated through Co-IP and WB; Huh7 cells expressing Myc-tagged cGPX4 and HA-Ub-K48 were treated with IU1-47 for 2 h prior to 6 Gy IR, with sample collection at specified times post-IR. **J** Sites of ubiquitination on GPX4 were identified by Co-IP and WB in Huh7 cells expressing single-lysine-to-arginine mutant cGPX4-Myc, treated with IU1-47 or DMSO for 2 h before 6 Gy IR, with sample collection 8 h later. **K** WB assays analyzed the expression of cGPX4-Myc. (**K-1**) USP14-WT or USP14-KO in Huh7 cells expressing various cGPX4-Myc mutants (WT, K48R, K118R, K48R + K118R) following 6 Gy IR, with sample collection 12 h post-IR; and at the indicated time points exposed to 6 Gy IR. (**K-2**) USP14-WT Huh7 cells expressing the same cGPX4-Myc mutants were pre-treated with IU1-47 or DMSO control prior to IR, with sample collection 12 h post-IR; and at the indicated time points exposed to 6 Gy IR. All results are indicative of three independent experiments.

These findings prompted us to investigate the mechanisms through which USP14 modulates GPX4 levels via ubiquitin-dependent proteolysis. Initially, we examined the potential interaction between USP14 and GPX4. IP followed by WB assays using both ectopically expressed Flag-tagged USP14 and the endogenous protein revealed a binding interaction between USP14 and GPX4 in response to RT, an association absent in untreated control cells (Figs. 5A and S11A). Similarly, an interaction was detected between the C-terminal Myc-tagged cytoplasmic form of GPX4 (cGPX4-Myc) and USP14 post-IR (Fig. 5B). Further IP/WB analyses indicated that the formation of USP14-GPX4 complexes was initiated as early as 1 h after RT, with the interaction strengthening over time, suggesting a radiation-induced association (Fig. 5C). We identified the proteasome as the primary degradation pathway for GPX4 in USP14-deficient cells. Proteasome inhibition (Bortezomib or MG132) prevented radiation-induced GPX4 degradation and reduced ubiquitin accumulation, while autophagy inhibition had no effect (Figs. 5D and S11B). To directly demonstrate GPX4 polyubiquitination, we developed an integrative approach: (1) Immunoprecipitation of endogenous GPX4 from cellular lysates; (2) Isolation of high-molecular-weight (HMW) fractions (>8 kDa shifts per ubiquitin); (3) Mass spectrometry identification of ubiquitin-specific KGG signatures. This revealed pronounced accumulation of polyubiquitinated GPX4 in USP14-KO cells following IR (Fig. S11C). These findings establish that USP14 maintains GPX4 stability by preventing its polyubiquitination and subsequent proteasomal degradation.

We further explored USP14’s role in modulating the ubiquitination and stability of GPX4. Considering that polyubiquitin chains serve as crucial signals for proteasome-mediated degradation, we performed IP/WB assay to determine whether USP14 affects the accumulation of polyubiquitinated GPX4. As expected, reducing USP14 expression or inhibiting its enzymatic activity in RT-treated HCC cells significantly elevated the ubiquitination of cGPX4. Despite the presence of overexpressed cGPX4, its protein levels were markedly diminished in the absence of USP14 or its enzymatic activity (Fig. 5E, F). This indicates USP14’s critical role in regulating and reversing GPX4’s ubiquitination, averting its unchecked ubiquitinated state following RT. To identify the specific polyubiquitin chains disassembled by USP14 on GPX4, we transfected cells with HA-tagged ubiquitin mutants, each containing a single lysine residue (with other lysines mutated to arginine), and conducted deubiquitination assays. USP14 inhibition led to the preferential accumulation of K11- and K48-linked polyubiquitinated cGPX4 after RT, rather than other linkages (Fig. 5G). This finding indicates USP14’s responsibility for removing K11 and K48-linked polyubiquitin chains from cGPX4. To further explore the nature of polyubiquitin chains on GPX4 induced by RT upon USP14 inhibition, we employed HA-tagged ubiquitin mutants with single lysine-to-arginine (K–R) mutations. The study revealed that only the K48R mutation prevented polyubiquitinated cGPX4 accumulation after radiation in the absence of USP14 activity (Fig. 5H) indicating that K48-linked ubiquitination is specifically negated by this mutation. In contrast to the weaker K63-linked ubiquitination observed in non-irradiated conditions upon USP14 inhibition (New Fig. S11D, E). Additional IP/WB analyses confirmed the progressive increase in K48-linked ubiquitinated cGPX4 levels post-RT (Fig. 5I). These findings collectively imply that USP14 recognizes and cleaves radiation-induced K48-linked polyubiquitination on GPX4. Subsequently, we aimed to pinpoint the exact ubiquitination sites on GPX4. Accordingly, we engineered mutant cGPX4 expression plasmids with single K to R substitutions at all potential ubiquitination sites (16 in total). We introduced these mutants into HCC cells and conducted in vitro ubiquitination assay, indicating that USP14 inhibition did not increase K48-linked ubiquitination for the K48R or K118R mutants post-RT (Fig. 5J), highlighting USP14’s role in specifically deubiquitinating cGPX4 at K48 or K118. Further mutational analysis identified K48 and K118 as critical ubiquitination sites on GPX4. Single mutations at either site stabilized GPX4 levels upon radiation treatment (RT) in USP14-deficient cells, while a K48/K118 double mutation completely blocked degradation (Fig. 5K). Importantly, in cells with functional USP14, RT failed to induce GPX4 degradation, further confirming USP14’s role in counteracting K48-linked ubiquitination. These findings demonstrate that USP14 preferentially cleaves K48-linked ubiquitin chains at lysines 48 and 118, thereby preserving GPX4 stability and its radioprotective function against ferroptosis.

### USP14 targets GPX4 for deubiquitination in a TRIM14-dependent manner

**Fig. 6:**
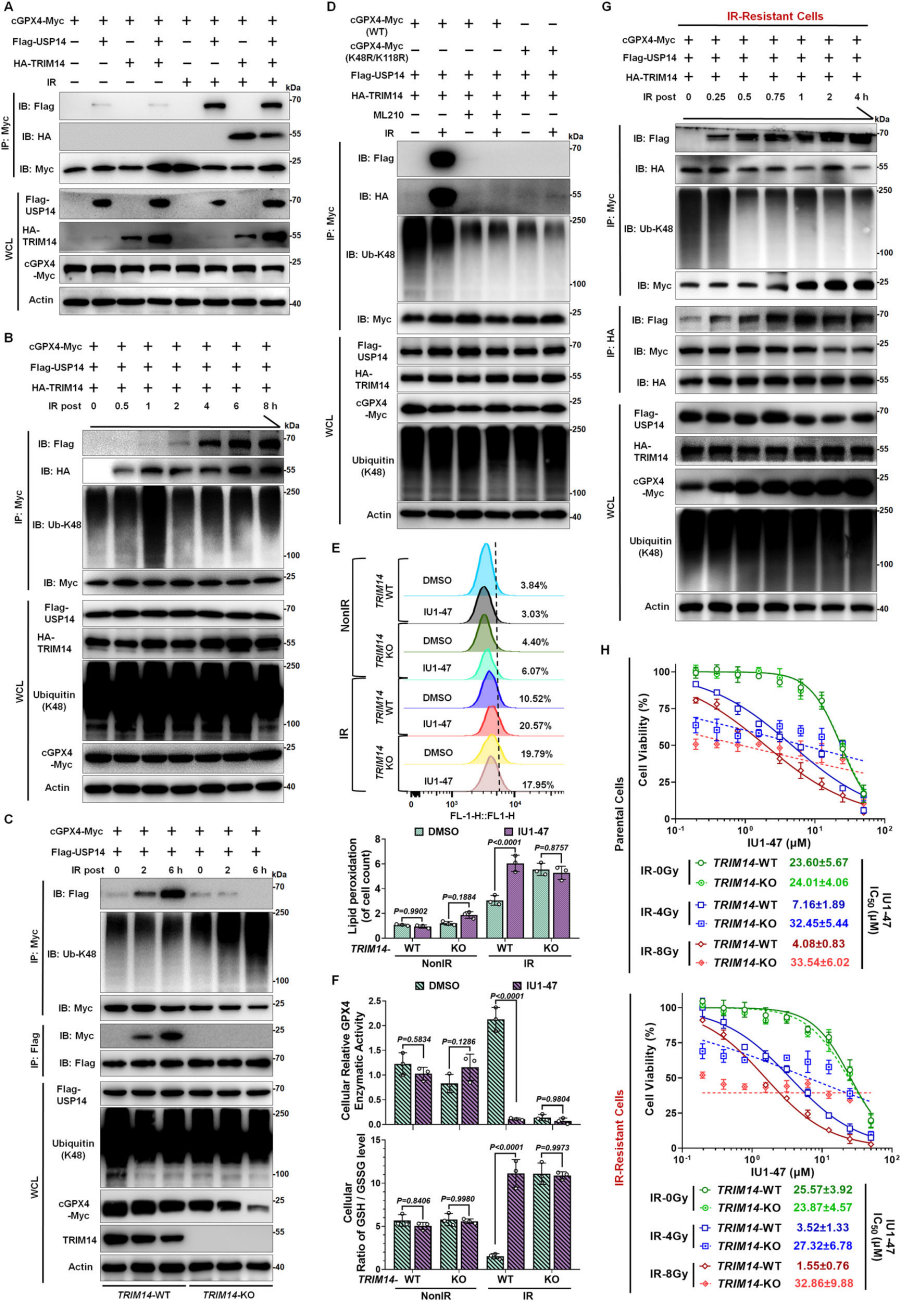
USP14 targets GPX4 for deubiquitination in a TRIM14-dependent manner. **A** Co-IP and WB assays elucidated interactions among TRIM14, USP14, and GPX4. Huh7 cells overexpressing cGPX4-Myc with Flag-USP14 or HA-TRIM14 underwent 6 Gy IR, with samples collected 6 h post-IR. Non-irradiated groups served as controls. **B** Co-IP and WB assays evaluated the temporal dynamics of TRIM14/USP14/GPX4 complex assembly. Huh7 cells overexpressing cGPX4-Myc with Flag-USP14 and HA-TRIM14 were exposed to 6 Gy IR, with cellular lysates collected at specified intervals. **C** Co-IP and WB assays explored the interaction between USP14 and GPX4 in the absence of TRIM14. Huh7 cells with TRIM14-WT or TRIM14-KO, overexpressing cGPX4-Myc with Flag-USP14, were subjected to 6 Gy IR, with lysates collected at 0/2/6 h post-IR. **D** Co-IP and WB assays assessed the impact of TRIM14/USP14/GPX4 complex formation on GPX4 mutations or ML210 treatment. Huh7 cells overexpressing Flag-USP14 and HA-TRIM14 were transfected with either wild-type cGPX4-Myc or cGPX4-Myc with K48R/K118R mutations. Alternatively, cells treated with ML210 were exposed to 6 Gy IR; lysates were gathered 6 h later. **E** Lipid peroxidation levels were quantified via BODIPY-581/591-C11 staining in TRIM14-WT and TRIM14-KO Huh7 cells pre-treated with IU1-47 (5 μM) before IR. Samples were harvested 12 h post-IR, with comparative data provided below. **F** The GSH/GSSG ratio (Upper) and GPX4 activity (Below) were evaluated in TRIM14-WT and TRIM14-KO Huh7 cells pre-treated with IU1-47 (5 μM) followed by 6 Gy IR. Cells were harvested 4 h post-IR for analysis. **G** Co-IP and WB assays investigated the temporal formation of the TRIM14/USP14/GPX4 complex in IR-resistant Huh7 cells. Those overexpressing cGPX4-Myc with Flag-USP14 and HA-TRIM14 underwent 6 Gy IR, with lysates prepared at specified intervals. **H** Cell viability was assessed in both wild-type and TRIM14-KO parental Huh7 cells, as well as in IR-resistant variants. Cells were treated with IU1-47 prior to varying IR doses (0/4/8 Gy), using DMSO as a control. Viability data collected 72 h post-IR, with results based on three independent experiments. The IC_50_ was calculated by normalizing cell viability to the drug-pretreated and IR control group or drug-pretreated control group (without IU1-47), which was set as 100% survival. Data are presented as mean ± SD. For (**E**) and (**F**): One-way ANOVA was employed for comparisons among the indicated groups.

Based on the protein interaction patterns outlined above, we propose a hypothesis suggesting the existence of a recruitment mechanism whereby USP14 targets GPX4 for deubiquitination, facilitated by RT. Existing reports have indicated that TRIM14 as a key adaptor that enables USP14 to remove ubiquitin from cGAS or p100/p52 in response to external stressors [31, 32]. We hypothesized that TRIM14 collaborates with USP14 to modulate GPX4 stability post-RT. To investigate this, we conducted Co-IP and WB assays to delineate interactions among TRIM14, USP14, and GPX4. Our findings confirm that USP14 and TRIM14 form a complex with cGPX4 after RT (Figs. 6A and S12A). To elucidate the temporal sequence, we immunoprecipitated cGPX4-Myc at various intervals post-RT and observed dynamic changes. TRIM14 associated with cGPX4 at 0.5 h post-IR, while USP14’s interaction occurred at 1 h. Notably, K48-linked polyubiquitination of cGPX4 increased shortly after RT but was rapidly counteracted by USP14 bound to cGPX4 (Fig. 6B). This illustrates TRIM14’s role in recruiting USP14 for GPX4 deubiquitination. The absence of TRIM14 compromised USP14’s interaction with cGPX4 and the deubiquitination process post-RT (Fig. 6C). This supports TRIM14’s role as a fundamental adaptor connecting USP14 and GPX4 for deubiquitination.

Significantly, the formation of the TRIM14/USP14/GPX4 complex post-IR was impeded by either using ML210, a covalent inhibitor targeting the active site of GPX4, or by mutating its ubiquitination site (Fig. 6D). This suggests that the ubiquitination of active GPX4 post-IR might be key in recruiting TRIM14 and USP14. Moreover, while USP14 inhibition with IU1-47 did not affect TRIM14’s recruitment of USP14 post-RT, it did prevent USP14-mediated cGPX4 deubiquitination (Fig. S12B). This implies that while USP14’s deubiquitinating activity is crucial for GPX4’s stability, it is not essential for initial TRIM14 recruitment. Phenotypically, increased lipid peroxidation induced by IU1-47 post-RT were mitigated in TRIM14-deficient cells (Fig. 6E). Time-course analysis identified 4 h post-IR as the optimal window for assessing IU1-47 effects. TRIM14 knockout significantly reduced GPX4 enzymatic activity (Figs. 6F and S12C), consistent with its role in regulating GPX4 proteostasis. These results demonstrate that TRIM14 is essential for mediating IU1-47’s effects on both lipid peroxidation and GPX4 function following radiation.

Given the significant increase in USP14 deubiquitinating activity during radiation resistance induction, we examined whether the TRIM14/USP14/GPX4 complex mechanism differs in radiation-resistant cells. Our experiments revealed that in these cells, TRIM14 binding to cGPX4 is constitutive, independent of radiation. Upon IR exposure, USP14 is rapidly recruited to deubiquitinate and monitor cGPX4’s ubiquitination status (Fig. 6G). This suggests that in radiation-resistant cells, TRIM14 facilitates a self-regulation system for GPX4 stability, ensuring timely removal of degradation signals via USP14. Radiation-resistant cells exhibit an accelerated stress response compared to parental cells. Furthermore, this complex’s ultimate function relies on USP14’s deubiquitinating activity. The USP14 inhibitor IU1-47 heightened sensitivity to RT in both parental and radiation-resistant cells, but this effect depended on TRIM14’s presence. In the absence of TRIM14, although RT efficacy was enhanced, IU1-47’s sensitizing effect was absent (Fig. 6H). These results collectively demonstrate that TRIM14 and USP14 cooperatively maintain GPX4 stability, facilitating ferroptosis defense in response to RT.

### Targeting and inhibition of USP14 amplifies RT-induced anti-tumor immunity via enhancing ferroptosis

**Fig. 7:**
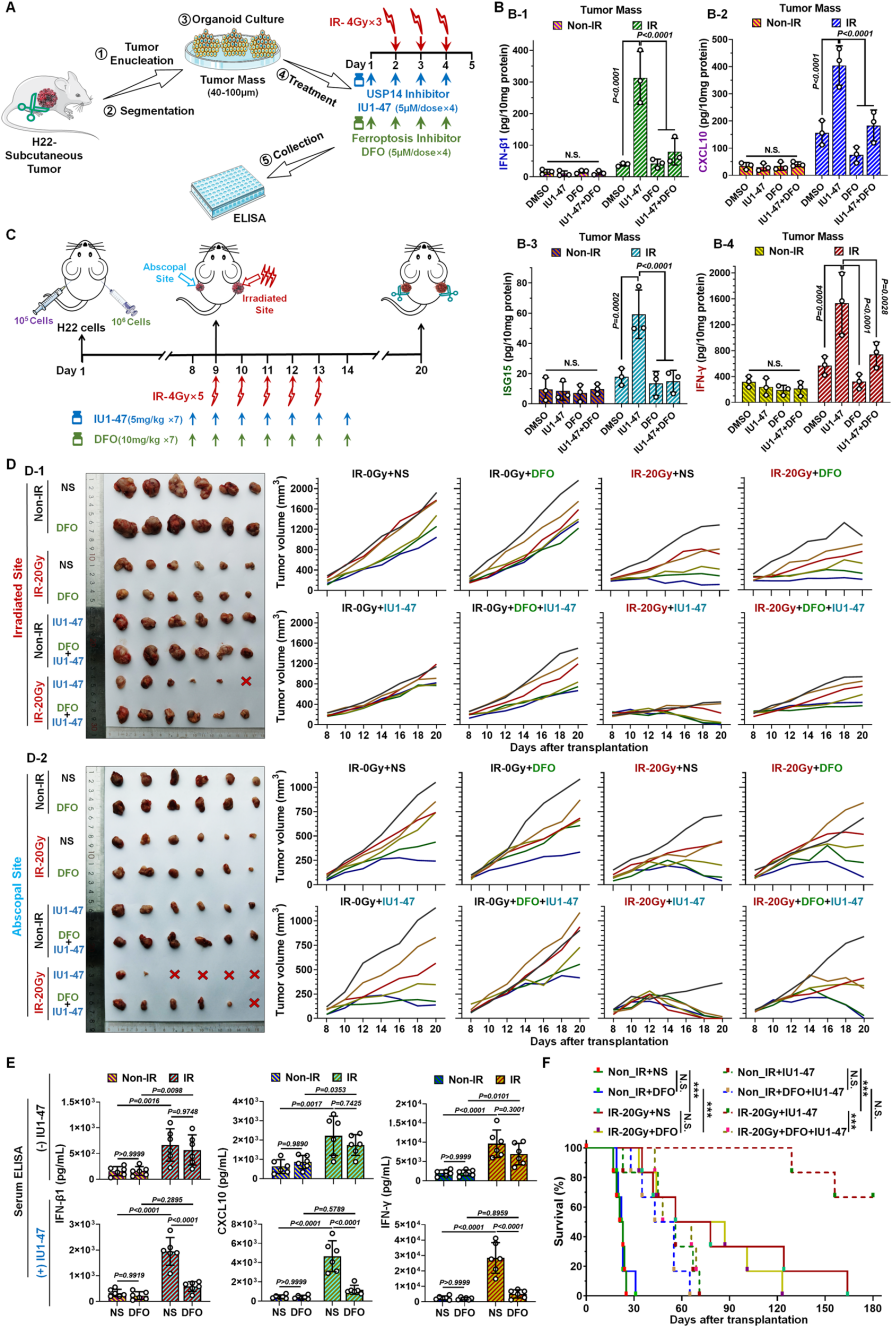
Inhibition of USP14 with IU1-47 enhances radiotherapeutic abscopal effect via inducing ferroptosis. Evaluation of the impact based on RT of tumor masses: **A** Schematic illustration. **B** Assessment of IU1-47 or (and) DFO on the secretion levels of IFN-β1 (**B-1**), CXCL10 (**B-2**), ISG15 (**B-3**), and IFN-γ (**B-4**) in the specified treatment groups of murine tumor masses. Results are presented as mean ± SD (*n* = 3). **C** Schematic representation of the experimental setup demonstrating the abscopal effect of RT on tumor-bearing mice (H22 tumors implanted bilaterally in the axillae) subjected to RT and IU1-47 or DFO. **D** Tumor growth trajectories for irradiated (IR) and non-irradiated (non-IR) mice administered IU1-47 or (and) DFO alongside vehicle (NS normal saline), divided into the 8 groups (*n* = 6/group) denoted in the figure. **D-1**, Irradiated tumor site; **D-2**, Abscopal tumor site. Left: tumor images; Right: tumor growth curves for each group. **E** Quantification of serum levels of IFN-β1, IFN-γ, and CXCL10 across the 8 groups. **F** Kaplan–Meier survival analyses of treated mice, illustrating survival outcomes across the aforementioned 8 groups (*n* = 6/group). Statistical significance was assessed via two-way ANOVA (n.s. not significant; ***, *P* < 0.001).

The elucidated mechanism convincingly illustrates that targeting USP14 remarkably magnifies the ferroptosis effect triggered by RT in HCC cells. The lethality of ferroptosis primarily arises from the integration of lipid peroxides into the membrane, resulting in membrane disruption. This disruption facilitates the release of substantial amounts of intracellular damage-associated molecular patterns (DAMPs) into the tumor microenvironment (TME), which plays a pivotal role in activating innate immune responses within the tumor [33]. Consequently, it is imperative to evaluate the influence of targeting USP14 on type I interferon (IFN) -mediated anti-tumor immune responses. Initially, a fundamental assessment was conducted using in vitro treatment of murine tumor organoids, which preserve the comprehensive architecture of the tumor and serve as an exceptional modality for evaluating treatments ex vivo. In this study, the assessment of innate immune response cells within the tumor, such as the activation of dendritic cells (DCs) following DAMPs uptake, can directly convey the authentic impact of RT on remodeling the TME. Thus, the method delineated in Fig. 7A was utilized to procure tumor organoids derived from H22 tumor-bearing models, subjecting them to RT and performing combinatory drug trials. The findings revealed that co-treatment with IU1-47 notably augmented the secretion of type I interferon (IFN-β1) within the tumor post-RT (Fig. 7B-1); the secretion of downstream effectors, C-X-C motif chemokine 10 (CXCL10) and Ubiquitin-like protein ISG15 (IFN-induced 15 kDa protein, ISG15) (Fig. 7B-2/3), was also similarly elevated. Crucially, the introduction of the ferroptosis inhibitor, the iron chelator DFO, abrogated the RT enhancement effect mediated by IU1-47. Remarkably, the independent application of IU1-47 and DFO showed no impact on untreated tumors, maintaining baseline conditions (Fig. 7B1–3). In conclusion, these results indicate that the strategy of targeting USP14 significantly amplifies the RT-induced enhancement of innate immune activation within tumors, derived from the augmented ferroptosis effect post-RT, rendering tumor cells immunogenic. Subsequently, indicators of adaptive immune responses were assessed, revealing that the secretion pattern of type II IFN (IFN-γ) paralleled that of type I IFN, with the immune response following RT mediated by IU1-47 also dissipating upon DFO introduction, underscoring IU1-47’s prospective efficacy in vivo applications(Fig. 7B-4).

The assessment of the abscopal effect of RT constitutes a crucial method for evaluating the in vivo anti-tumor immune response subsequent to RT. Utilizing a bilateral tumor implantation approach with unilateral IR, we assessed the growth inhibition of both irradiated and non-irradiated tumors, adhering to the protocol outlined in Fig. 7C. Given that the abscopal effect of RT typically manifests under medium to high doses of continuous IR, we selected an IR regimen of 4 Gy/day over a span of five consecutive days. The therapeutic outcome observed in the irradiated tumors demonstrated a distinct radiosensitization effect facilitated by IU1-47, which was completely nullified by the addition of DFO, underscoring IU1-47’s role in enhancing HCC sensitivity to RT (Figs. 7D-1 and S13A). Analysis of the distant tumors revealed that IU1-47 significantly enhanced the abscopal effect, resulting in tumor regression in two-thirds of the mice; likewise, this enhancement was fully negated by the inclusion of DFO (Figs. 7D-2 and S13B). Subsequent serum analysis of the mouse groups showed that the secretion patterns of IFN-β1, along with downstream mediators CXCL10 and IFN-γ, mirrored the observed enhancement of the abscopal effect (Fig. 7E). Furthermore, survival analysis related to the abscopal effect of RT underscored the remarkable capacity of IU1-47 to aid in controlling the growth of both irradiated and distant tumors, thereby prolonging survival-a capability largely driven by the intensified ferroptosis effect. Upon inhibition of ferroptosis at its inception, the survival-prolonging capability of IU1-47 was also nullified (Figs. 7F and S13C). In conclusion, the strategy of targeting USP14 with IU1-47 emerges as a potent driver in amplifying the ferroptosis efficacy mediated by RT, playing a pivotal role not only in radiosensitization but also in post-RT immune activation, ultimately enhancing the overall effectiveness of RT.

## Discussion

The human genome contains approximately 100 DUBs. The primary subgroups of DUBs are cysteine proteases, including ubiquitin C-terminal hydrolases (UCHs), ubiquitin-specific proteases (USPs), and ovarian tumor proteases (OTUs) [11, 34]. In studies investigating the prevention of ferroptosis, DUBs typically perform their function by maintaining the homeostasis of specific functional proteins. For instance, USP11 helps stabilize the NRF2 transcription factor of GPX4 [35], USP35 stabilizes the iron transporter FPN to inhibit the initiation of ferroptosis [36], and OTUB1 stabilizes SLC7A11 to promote the construction of the GPX4-dependent defense mechanism [37]. Thus, given the crucial role of deubiquitination regulation in suppressing ferroptosis, the study of DUBs has gained prominence in mechanistic research [34]. In our work, we discovered that DUB-mediated deubiquitination potentially confers radioresistance in HCC; therefore, we further applied activity-based profiling using a CRISPR-based DUB screen and identified the targetable DUB USP14 as a significant factor underlying radioresistance, which stabilizes GPX4 in the defense against ferroptosis. Notably, USP14 does not regulate GPX4 in the absence of radiation, suggesting that RT induction is the driving force for USP14 activity. Notably, previous studies have suggested that USP31 is a deubiquitinating enzyme for GPX4, involved in regulating its stability [38]. To investigate this, we conducted experiments and confirmed that USP31 does indeed bind to GPX4. However, this interaction is significantly weakened following RT. Moreover, the presence or absence of USP31 does not influence the accumulation of K48 ubiquitination modification on GPX4 (Fig. S14A). Furthermore, functional verification revealed that USP31-KO does not affect the radiosensitivity of HCC cells (Fig. S14B). These findings indicate that the radiation-driven process involving USP14 targeting and deubiquitinating GPX4 is relatively specific.

USP14, a thiol hydrolase, belongs to the ubiquitin-specific family of hydrolytic enzymes [39]. It possesses unique structural features, including a UBL (Ubiquitin-like) domain (amino acids 1-80) containing the N-terminal and a catalytic hydrolysis domain (amino acids 96-494) containing the C-terminal [40]. The UBL domain recognizes specific substrates with its unique conformation, and deubiquitination is dependent on the activation of the C-terminal catalytic domain. Functionally, USP14 is a member of the DUB system of proteasome 19S regulatory particles (RP). However, the precise deubiquitylation of specific protein substrates by USP14 is often independent of proteasome function [41, 42]. Our results showed that loss of USP14 did not affect the radiation-induced elevation of proteasome activity, suggesting that USP14 function is not dependent on the proteasome. Several studies have indicated that USP14 promotes radioresistance by regulating autophagy or DNA repair in various cancers [43–45]. However, the precise molecular mechanism for acquired resistance following radiation has not been fully defined. Additionally, the role of USP14 in regulating ferroptosis, particularly when driven by IR, remains unclear. Our work not only revealed the important function of USP14 in the RT tolerance of liver cancer but also provided insights into the mechanism of radiation-driven ferroptosis defenses. Unlike E3 ligases, DUBs are more susceptible to targeting by small-molecule inhibitors due to their well-defined catalytic residues. This provides a promising avenue for developing novel therapies [34, 46]. Accordingly, developed USP14 inhibitors could be used to elucidate the role of ubiquitin-conjugation machinery in ferroptosis defense and may represent a promising therapeutic strategy for radiosensitization in HCC.

**Fig. 8:**
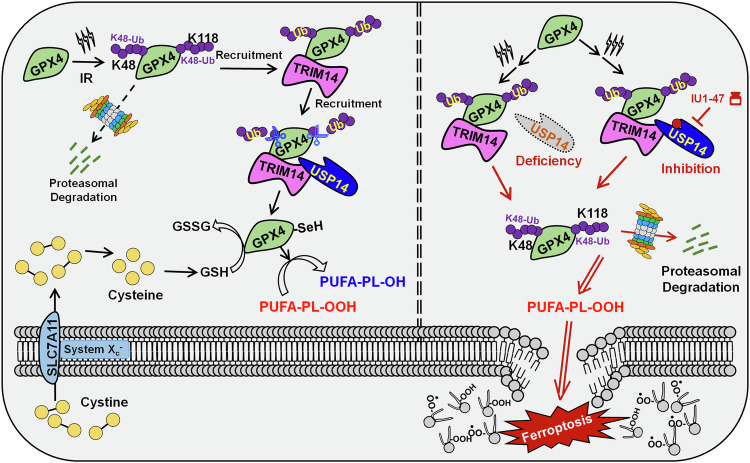
This diagram delineates the regulatory role of USP14 in GPX4-mediated anti-ferroptotic defense, which contributes to radioresistance in HCC. Radiation prompts the interaction of TRIM14 with GPX4, recruiting USP14 into the complex. USP14 subsequently cleaves K48-linked polyubiquitin chains from GPX4 at lysine 48 or 118, thereby counteracting radiation-induced ubiquitination that would otherwise target GPX4 for degradation. By stabilizing GPX4 and preserving its antioxidant activity, USP14 suppresses radiation-triggered ferroptosis. Pharmacological inhibition of USP14 significantly sensitizes HCC cells to radiation-induced ferroptosis while also potentiating the immune response elicited by radiotherapy, ultimately enhancing therapeutic efficacy.

GPX4, a primary enzyme, constitutes the main surveillance system that defends against ferroptosis in cancer cells, primarily through the SLC7A11-GSH-GPX4 signaling axis [25]. As a selenoprotein, GPX4 requires selenium supplementation for protein synthesis and selenocysteine-mediated GSH biosynthesis for activation. GPX4 utilizes GSH as a cofactor to convert toxic lipid hydroperoxides (PL-PUFA-OOH) into non-toxic lipid alcohols (PL-PUFA-OH), maintaining the integrity of lipid bilayers and preventing ferroptotic cell death [47]. However, SLC7A11 inhibition (i.e., inhibition of extracellular cysteine uptake) blocks or depletes GSH synthesis, inactivating or degrading GPX4, leading to the accumulation of toxic lipid peroxides and triggering ferroptosis [48, 49]. GPX4 activity is implicated in persistent drug tolerance or therapy resistance in various cancers [50, 51]. Although the precise regulation mechanisms of GPX4 remain largely unknown, this study reveals that USP14 targets GPX4 and cleaves the radiation-induced K48-linked ubiquitination of GPX4 at K48 or K118. This maintains GPX4 stability and activity in response to radiation. These findings not only enhance our understanding of the precise regulation of GPX4 ubiquitination and degradation but also suggest a potential strategy for inducing GPX4 degradation after radiation treatment. FINs targeting GPX4 fall into two mechanistic classes: (1) direct covalent inhibitors, such as (1S, 2 R)-RSL3, ML162, or ML210^5, 48^, and (2) inducers of degradation, such as FIN56 [49]. Interestingly, targeting USP14 with IU1-47 could overcome off-target or tolerance effects of direct covalent GPX4 inhibitors like ML210 by inducing active GPX4 degradation. Our study revealed that the degradation of GPX4 mediated by IU1-47 was significantly superior to that mediated by FIN56 (Fig. S14C), highlighting the advantage of targeting USP14 after IR. Furthermore, combining USP14 deletion or inhibition with FSP1 targeting using iFSP1 further potentiated the induced ferroptosis effect. However, targeting GCH1 with DAHP did not have the same effect. This observation suggests that an alternative defense mechanism mediated by FSP1 may partially compensate for GPX4-dependent ferroptosis inactivation in HCC. Our research has elucidated the mechanistic foundations of the central function of deubiquitination in radioresistance of HCC. Our findings have unveiled a new mechanism wherein TRIM14/USP14 axis governs the stability and functionality of GPX4 in shielding against ferroptosis and radioresistance in HCC (Fig. 8). In radiation-resistant cells, TRIM14 establishes a stable complex with GPX4, which in turn recruits USP14, facilitating an efficient and sensitive response to radiation-induced ferroptosis.

Our central discovery is that radiation latently triggers GPX4 degradation (via ubiquitination) but is counteracted by the TRIM14-USP14 axis, a natural radioprotective mechanism. This is now robustly validated across multiple HCC lines (Huh7/SNU449/MHCC97H + 7 additional models in new Fig. S8B–D): Baseline Conditions: Radiation alone fails to reduce GPX4 in any HCC line (Fig. S8B), confirming USP14’s constitutive protective role. USP14-Deficient States: Radiation induces pronounced GPX4 degradation universally (Fig. S8D), proving that: (i) Radiation provides the trigger for GPX4 ubiquitination (Fig. 5); (ii) USP14 is the obligatory barrier preventing this degradation (Fig. 5); (iii) The effect is IR-dependent (no GPX4 loss in USP14-KO cells without radiation) (Fig. S8C). These data solidify our model of USP14 as a radiation-activated GPX4 stabilizer and explain why HCC cells intrinsically resist radiation-induced ferroptosis under physiological conditions. One Study reports GPX4 transcriptional downregulation after IR in intestinal epithelial cells [52]. In our HCC system, however, GPX4 mRNA shows only transient suppression (with partial recovery) under normal conditions but exhibits a paradoxical upregulation upon USP14 inhibition before returning to baseline by 24 h (Fig. S8E). We interpret this as compensatory feedback triggered by GPX4 protein degradation. Meanwhile, to further validate this interpretation, we conducted complementary experiments showing that: (1) GPX4-KD in USP14-OE cells substantially increased radiosensitivity, with cellular sensitivity remaining elevated regardless of USP14 overexpression status. This observation strongly indicates that GPX4 serves as the critical downstream effector of USP14, and its absence completely abolishes USP14’s regulatory function (Fig. S10A, C); (2) Ectopic expression of GPX4 in USP14-KO cells significantly restored the IR resistance phenotype. This restoration can be attributed to the fact that USP14-mediated degradation could not fully counteract the high expression levels of exogenous GPX4-a phenomenon we subsequently confirmed through mechanistic studies. These results demonstrate that GPX4 overexpression effectively rescues the compromised cell viability caused by USP14 ablation (Fig. S10B, D).

Our comprehensive review of seminal studies revealed that IR promotes GSH oxidation to GSSG via free radical-mediated thiol oxidation [53]. This aligns with reports demonstrating IR-induced GSH depletion in HT1080 and normal hepatocytes [22, 54]. Notably, while SLC7A11 inhibitors (e.g., Erastin or sorafenib) trigger rapid GSH depletion within 5–24 h due to cysteine import blockade, GPX4 inhibitors (e.g., RSL3) show no acute GSH modulation (5 h) [48, 55]. These findings motivated our time-resolved analysis (1–24 h post-IR) to capture dynamic GSH/GSSG fluctuations, avoiding single-timepoint artifacts. Time-course analysis reveals USP14-dependent GSH/GSSG homeostasis: our kinetic studies demonstrated that: In USP14-proficient cells, IR induced progressive GSH → GSSG conversion (0–8 h), followed by recovery to baseline by 24 h. Upon USP14 inhibition/deletion, this oxidative shift was abolished, with GSH/GSSG ratios instead of increasing post-IR. This suggests USP14 sustains GPX4 activity to facilitate GSH-dependent peroxide detoxification. Mechanistic link between USP14 and GPX4: to validate USP14’s reliance on GPX4, we conducted two parallel experiments: pharmacological inhibition: RSL3/FINO2 mimicked IU1-47’s effects, causing early GSH/GSSG elevation followed by equilibration. Genetic ablation: GPX4 KO cells exhibited no IR-induced GSH oxidation, and IU1-47 lost efficacy, confirming GPX4 as the obligatory downstream effector of USP14. Collectively, these data establish that USP14 maintains post-IR redox balance by stabilizing GPX4. USP14 dysfunction (via inhibition or deletion) disrupts GPX4-mediated GSH cycling, leading to aberrant GSH/GSSG ratios that predispose cells to ferroptosis. We have incorporated these findings into the revised manuscript, including new time-course datasets and mechanistic validation. Thank you for highlighting this pivotal aspect of our study.

Our research raises the question of why radiation triggers K48-linked polyubiquitination of GPX4. One possible explanation is that radiation induces endoplasmic reticulum (ER) stress, leading to the overactivation of the ubiquitin-proteasome pathway (UPP) [56–58]. GPX4 is readily ubiquitinated by E3 ubiquitin ligases. However, due to its crucial role in resisting radiation-induced ferroptosis, the TRIM14/USP14-mediated deubiquitylation process promptly alleviates the repressive state of GPX4. Therefore, investigating the molecular mechanism of radiation-induced GPX4 ubiquitination will be a compelling area for future research. Furthermore, a variety of USP14 inhibitors, including IU1-47, are presently undergoing preclinical studies. Their drug performance, safety evaluation, and further structural optimization will be the focus of upcoming research efforts.

An expanding corpus of evidence underscores that the DNA damage and cytotoxic effects induced by RT in tumor cells are pivotal to its antitumor efficacy, significantly influencing the immunomodulatory impact on the TME. The immune response elicited by RT constitutes a critical determinant of both the efficacy and the duration of its therapeutic benefit [59]. From an efficacy research standpoint, the predominant hypothesis is that RT primarily operates by triggering the release of pro-inflammatory mediators or DAMPs. This cascade elevates the innate immune response of antigen-presenting cells, such as DCs, along with other immune-stimulatory cells, facilitating their activation and infiltration. As a result, this enhances the cross-presentation of tumor antigens, thereby modulating the tumor’s immunogenicity. Such phenomena are often encapsulated in the notion that RT converts the immune status (inflammatory phenotype) of tumors from “cold” to “hot“ [60]. This study thoroughly demonstrates the theoretical correlation between ferroptosis and the degree of tumor immune response, further revealing that targeting USP14 to enhance ferroptosis plays a positive role in the remodeling of the tumor immune microenvironment post-RT. This not only deepens the understanding of the intrinsic nature of RT-induced ferroptosis but also, on an application level, proves that targeted strategies to enhance ferroptosis increase RT sensitivity. Moreover, it significantly promotes immune response and the formation of immune memory within tumors following RT. As such, it provides robust support for the contribution of RT to the efficacy and durability of comprehensive cancer treatment, paving the way for its extensive future application.

## Material and methods

### Cell culture

Huh-7, MHCC97H, SNU449, and HEK293T were cultured in DMEM medium, supplemented with 10% fetal bovine serum and 1% penicillin-streptomycin. Cells were maintained at 37 °C in 5% CO_2_. All cell lines were authenticated by short tandem repeat analysis at China Center for Type Culture Collection (Wuhan, China), and the absence of mycoplasma contamination was verified using a PCR detection kit. Cells were frozen in liquid nitrogen and used for experiments at passages 4–10 after thaw.

### Colony formation assay

The colony formation assay was performed in a 2D culture model as described previously [24, 26]. Briefly, HCC cells were seeded into 6-well plates at a density of 1500–2500 cells/well. After 24 h, the cells were treated with inhibitors and then radiation and cultured for 10–14 days, and then followed by staining with 0.5% crystal violet fixed in methanol. Colonies containing more than 50 cells were counted. The plating efficiency (PE) was defined as the number of colonies/the number of seeded cells × 100%. The survival fraction was defined as the number of colonies at one IR dose divided by the number of colonies with a correction for the PE [8].

### Human HCC samples

This research was approved by the Institutional Ethics Committee for Clinical Research and Animal Trials of the First Affiliated Hospital, Sun Yat-sen University (SYSUFAH) (Approval No. [2023]-044). A total of 77 formalin-fixed and paraffin-embedded (FFPE) tumor biopsy specimens were collected from patients pathologically diagnosed with HCC prior to RT from SYSUFAH. They all received radical RT (VMAT, 50 Gy, 2 Gy/day, 5 days/week). Informed consent was obtained from the patients and the study is compliant with all relevant ethical regulations regarding research involving human participants. The OS was defined as the interval between the first resection to the date of death from any cause or to the date of the last follow-up visit; PFS was defined as the time from achieving treatment to radiological or clinical disease progression or death, and this was calculated.

### Plasmid constructs and lentiviral transduction or transient transfection

The coding sequences of full-length human wild-type (WT) USP14, mutated USP14 (C114A), tagged with 3× Flag at the N-terminus; and ubiquitin (WT, K6-only, K11-only, K27-only, K29-only, K33-only, K48-only, K63-only, non-K, K6R, K11R, K27R, K29R, K33R, K48R, K63R), full-length human TRIM14 tagged with HA at the N-terminus; cytoplasmic GPX4 tagged with Myc at the C-terminus were inserted into the pCDH-EF1-MCS-T2A-Puro plasmid, respectively. DUB sgRNAs and sgRNA-Negative control (Scrambled); USP14 sgRNA1-3; GPX4 sgRNA and TRIM14 sgRNA were cloned into the lenti-CRISPR v2 vector. Lentiviruses were produced by co-transfection of the above constructs, psPAX2 and pMD2.G plasmids, into HEK293T cells, and viruses were transduced into CRC cells, followed by puromycin selection to generate stable cell lines.

GPX4, as a Selenoprotein, is an elite group of proteins containing a rare amino acid, selenocysteine (Sec), encoded by the codon UGA. In eukaryotes, incorporation of Sec requires a Sec insertion sequence (SECIS) element, a stem–loop structure located in the 3’-untranslated regions of selenoprotein mRNAs [61]. For GPX4 expression, cGPX4-Myc plasmid was co-transfected with SBP2L (SECIS-binding protein2L) to HEK293T cells as described above.

Transient transfection was performed using Lipofectamine 3000 transfection reagent or electroporation (Gene Pulser Xcell, Bio-Rad) according to the manufacturer’s instructions.

### The activity assays of deubiquitination

As described previously, cells or xenografts were treated with or without IR, then were lysed using lysis buffer to obtain whole-cell or tissue protein according to the manufacturer’s instructions. Then, lysates were subjected to Ub-AMC hydrolysis assay. In brief, whole cell lysates (WCL, 5 μL) were incubated in assay buffer (50 mM Tris-HCl, 5 nM MgCl_2_, 1 mM DTT, 2 mM ATP, 250 nM sucrose, pH 7.5) for 30 min at 37 °C in 96-well plates, and then Ub-AMC was added to 400 nM (final reaction volume was 100 μL).

For the deubiquitinating activity assays of USP14 from cells, Flag-USP14 was purified from cell lysates using Anti-Flag Affinity Gel according to the manufacturer’s instructions was used. In brief, enzymes (final concentration, 2 nM) were incubated in assay buffer (50 mM Tris-HCl, 1 mM EDTA, 5 nM MgCl_2_, 1 mM DTT, 2 mM ATP, 250 nM sucrose, and 1 mg/mL ovalbumin, pH 7.5) with or without inhibitors of indicated concentrations for 30 min at 37 °C in 96-well plates, and then Ub-AMC was added to 400 nM (final reaction volume was 100 μL).

The plates were subjected to measure fluorescence density on a BioTek Epoch Multi-Mode Microplate Reader at 380 nm of excitation wavelength and 460 nm of emission wavelength every 2 min for 1 h. The deubiquitinating activities were calculated as the fluorescence increment (folds) per minute, indicating the deubiquitination rate of substrate Ub-AMC.

### Real-time PCR assay

Real-time PCR was performed as described previously [8]. A RNeasy kit was used to extract total RNA from cells, cDNA reverse transcription assay was performed to prepare first-strand cDNA. Subsequently, QuantiTect SYBR Green PCR kit or TaqMan Universal PCR Master Mix was used to perform real-time PCR on Stratagene MX3000P. For quantification of gene expression, the 2^−ΔΔCt^ method was used with expression normalized to Actin. The sequences of the primers: PTSG2-F, 5’-CTGATGATTGCCCGACTCCC-3’, PTSG2-R, 5’-TCGTAGTCGAGGTCATAGTTC-3’; Actin-F, 5’-CGGAACCGCTCATTGCC-3’, Actin-R, 5’-ACCCACACTGTGCCCATCTA-3’.

### Lipid peroxidation assay

HCC cells were seeded in 6-well plates 1 day before treatment, pretreated with or without drugs for 12 h, and/or then treated with radiation. At 6–12 h post-radiation, the cell culture medium of each well was replaced with a fresh medium containing 5 μM BODIPY 581/591 C11 dye for lipid peroxidation measurements and incubated for 30 min in a cell culture condition. Subsequently, cells were washed with PBS and trypsinized to obtain a cell suspension. Lipid peroxidation levels were analyzed by flow cytometry using a CytoFLEX flow cytometer.

### Co-immunoprecipitation (co-IP) and western blot (WB) assays

Co-IP and WB assays were performed as described previously [46]. Briefly, cells were lysed in lysis buffer supplemented with protease and phosphatase inhibitors, and then protein concentration was quantified using a BCA protein assay kit. For co-IP assay, cell lysates were incubated with antibody beads or primary antibodies overnight at 4 °C plus protein G agarose beads for 4 h at 4 °C. After washing, the pulldown products were subjected to SDS-PAGE assay. For WB assay, protein bands separated by SDS-PAGE were transferred onto PVDF membrane, and the membrane was blocked with 5% milk in TBS and incubated overnight at 4 °C with specific primary antibodies, followed by incubation with HRP-conjugated secondary antibodies. Signals were visualized using an enhanced chemiluminescence reagent and captured by ChemiDoc IRS system, and quantified using Image J.

### DUBs CRISPR library screening

Human DUBs CRISPR Knockout Pooled Library was generated in the lenti-CRISPR v2 vector. sgRNAs containing 87 DUB genes (2 sgRNAs per target gene) [46] were transduced with lentivirus particles containing the DUBs sgRNA library with no greater than one sgRNA per cell. The Cas9-sgRNA-HCC cells were selected with 2 μg/mL puromycin for 2–3 weeks to achieve >95% gene knockdown. These Cas9/sgRNA-Huh7 cells were then treated as Fig. 1J model, and the surviving resistant population was expanded and subjected to deep sequencing analysis for candidate genes. For in vivo screening, Cas9/sgRNA-MHCC97H cells (3 × 10^6^) were subcutaneously injected into right flank to establish primary xenografts. Then, primary xenografts were cut into sections (~5 mm^3^) and transplanted into new mice. Once the xenograft volume reached ~100 mm^3^, mice were treated alternately with radiation (Fig. 1J), and the residual xenografts were expanded and subjected to deep sequencing analysis for candidate genes. Animal experiments were approved by Affidavit of Approval of Animal Use Protocol, IACUC, SYSU (Approval number: SYSU-IACUC-2022-001788). Female BALB/c nude mice (4–5 weeks, 15–18 g; SLRC Laboratory Animal Co., Shanghai, China) were used in these experiments.

### Cell viability assay

HCC cells (2500/well) were cultured in 96-well plates for 24 h, pretreated with or without drugs for 12 h, After 72 h, the medium was replaced by a 100 μL CCK8 medium mixture (1:9), followed by 2 h of incubation at dark. Then, the absorbance at 450 nm was measured by a microplate reader.

### GSH/GSSG ratio detection assay

For cell samples, first, harvest the number of cells necessary for each assay and tried starting with 10,000 cells/well. And then washed cells with cold PBS; resuspended cells in 100 µL of ice-cold 1× Mammalian Lysis Buffer and homogenized cells quickly by pipetting up and down a few times. Next, centrifuged samples for 15 min at 4 °C at top speed using a cold microcentrifuge to remove any insoluble material and then collected supernatant and transfer to a clean tube. Samples should be removed from the samples by using Deproteinizing Sample Kit-TCA.

Reaction well set up according to the manufacturer’s instructions. For GSH detection, prepared GSH assay mixture (GAM) by adding 100 μL of 100× Thiol Green Stock solution into 10 mL of assay buffer in 96-well plates. For GSH + GSSG detection, prepare total glutathione assay mixture (TGAM) by adding 5 mL of GAM solution into the bottle of the GSSG Probe and mix well by vertexing in 96-well plate.

Run GSH and total GSH assay: for GSH detection, added 50 µL of GAM into each GSH standard and sample well (Panel A) to make the total assay volume 100 µL/well. For Total GSH + GSSG (reduced and oxidized), added 50 µL of total TGAM into each GSSG standard and sample well (Panel B) to make total assay volume 100 µL/well. And then, incubated at room temperature for 30 min protected from light. Monitor fluorescence at Ex/Em = 490/520 nm with a fluorescence microplate reader.

### GPX4 enzyme activity detection

HCC cells were plated in 10-cm culture dishes at a density of 2 × 10^6^ cells/dish and allowed to adhere for 24 h. For experimental groups, cells were either pretreated with IU1-47 for specified durations or subjected to USP14 knockout prior to IR treatment. Following these interventions, cells were harvested, and endogenous GPX4 was immunoprecipitated using co-IP methodology. GPX4 enzymatic activity was subsequently determined using a commercial GSH peroxidase assay kit (Abcam, ab102530) according to the manufacturer’s instructions.

### Nude mouse xenograft model and experimental therapy

Animal experiments were approved by Affidavit of Approval of Animal Use Protocol, IACUC, SYSU (Approval number: SYSU-IACUC-2022-001788). Female BALB/c nude mice (4–5 weeks, 15–18 g; SLRC Laboratory Animal Co., Shanghai, China) were used in animal experiments. For *USP14*-WT and *USP14*-KO MHCC97H cells, 1 × 10^6^ cells were subcutaneously injected into right flank to generate xenografts directly. Once the xenograft volume reached ~75 mm^3^ at day 8, mice were treated alternately with radiation. Subsequently, mice were sacrificed 2 weeks after the last treatment, and then xenografts were removed, weighed, and subjected to pathological analysis.

For NEM in combination with IR treatment, 3 × 10^6^ cells were subcutaneously injected into right flank to establish primary xenografts. Then, primary xenografts were cut into sections (~50 mm^3^) and transplanted into new mice. Once the xenograft volume reached ~50 mm^3^, mice were treated alternately with NEM and IR. The mice were randomized into four groups and treated with Normal Saline (NS), NEM, IR, or NEM + IR in combination groups. IR treatment was applied locally to the tumor in the flank of mice at 2 Gy × 5 times. NEM was dissolved in DMSO (1%) and diluted in HS-15 (5%), ultimately, the preparation was prepared into a NS (94%) solution. And then intratumorally injected into mice ten times at a dose of 5 mg/kg followed by continued injection every day until the endpoint, as indicated in the corresponding figures. The tumor volume was calculated using the following formula: tumor volume = 0.52 × width^2^ × length. Subsequently, mice were sacrificed 3 weeks after the last treatment, and then xenografts were removed, weighed and subjected to pathological analysis.

### Patient-derived xenograft (PDX) experiments

PDXs were generated in accordance with protocols approved by Institutional Ethics Committee for Clinical Research and Animal Trials of the First Affiliated Hospital, Sun Yat-sen University (SYSUFAH) (Approval No. [2023]-044). Informed consent was obtained from the patients and the study is compliant with all relevant ethical regulations regarding research involving human participants. Animal experiments were approved by Affidavit of Approval of Animal Use Protocol, IACUC, SYSU (Approval number: SYSU-IACUC-2022-001788). 4–5 weeks old NOD SCID gamma (NSG) female mice were purchased from SLRC Laboratory Animal Co., Shanghai, China. The PDX model used in this study was originally obtained from the HCC PDX platform at SYSUFAH. PDX experiments were performed as previously described [8]. Briefly, PDX tumors in cold cell culture media were minced into fragments 5–10 mm^3^. Then each PDX tumor fragment was subcutaneously injected into right flank of NSG mice. When the tumors reached 50–75 mm^3^, the mice were randomized into four groups and treated with Normal Saline (NS), IU1-47, IR, or IU1-47 + IR in combination groups. IR treatment was applied locally to the tumor in the flank of mice at 2 Gy × 5 times. IU1-47 was dissolved in DMSO (1%) and diluted in HS-15 (5%), ultimately, the preparation was prepared into a NS (94%) solution. And then intraperitoneally injected into mice ten times at a dose of 5 mg/kg followed by continued injection every day until the endpoint as indicated in the corresponding figures. The tumor volume was measured three times per week until the endpoint and calculated according to the equation volume = 0.5 × width^2^ × length. Subsequently, mice were sacrificed 16 or 22 days after the last treatment, and then xenografts were removed, weighed, and subjected to pathological analysis. In the case of survival analysis, mice were observed for an additional 7 weeks.

### Determination of cytokine secretion from tumor spheroids derived from tumor-bearing mice

Inject H22 cells subcutaneously into the left axillary region of Balb/c mice (10^7^ cells per mouse). When the tumor volume reaches 500 mm^3^, euthanize the mice and excise the tumors. Immediately divide the tumors into 40–100 mm^3^ fragments and place them into culture flasks. Culture the tumor fragments for 3–4 days using organoid culture methods. After this period, evenly divide the tumor fragments into groups, ensuring each group contains at least five fragments. Follow the treatment protocols indicated in the main text figure for irradiation and drug treatment of the tumor fragments. Post-treatment, collect the tumor fragments, homogenize them, and extract proteins. Use ELISA to measure the secretion levels of cytokines or chemokines, such as IFN-β1, CXCL10, ISG15, and IFN-γ.

### Evaluation of the abscopal effect of radiotherapy

#### Bilateral tumor implantation was performed to evaluate the impact of various drugs on the irradiated and contralateral tumors

H22 cells were subcutaneously implanted into the left and right axillae of Balb/c mice according to the methodology depicted in the main text. On the 8th day post-implantation, when the tumor volume in the irradiated side exceeded 100 mm^3^, treatment was administered as illustrated in the main text. The drugs were administered via intraperitoneal injection (dissolved in 1% DMSO, diluted in HS-15, and then prepared into a 94% NS solution). During irradiation, mice were anesthetized, and all areas except the tumor were shielded with lead plates. Tumor volumes on both sides were recorded every 2 days starting from the 8th day. Tumor volume was calculated using the formula: Volume = 0.5 × width^2^ × length. Following the completion of the treatment cycle, the mice were observed for an additional 6 days. On the 20th day, the mice were sacrificed, tumors were excised, and blood was collected via orbital puncture. Serum levels of various cytokines and chemokines were evaluated using ELISA assay [62].

#### Survival rate studies in mice

A separate group of female Balb/c mice was purchased and subjected to tumor transplantation, grouping, administration, and RT in accordance with the experimental protocol for unilateral axillary tumors described previously. Tumor volume was recorded, and when the tumor volume on either side reached 2000 mm^3^, the survival endpoint was noted, and the mice were euthanized. The survival rate of each group of mice was recorded daily.

### Histology and immunohistochemistry (IHC) staining

Histology and immunohistochemistry assays were as previously described in ref. ^8^. The tissue and tumor specimens were fixed in buffered 4% formalin for 24 h followed by paraffin embedding. For the hematoxylin and eosin (H&E) staining, after was dewaxed in xylene, rehydrated in ethanol, and washed in PBS, the tissue sections were stained with H&E. Then, the sections were dehydrated using ethanol and xylene. The Edmonson-Steiner system, based on nuclear and cellular atypia, was used to grade the cytological features of HCC cells. For the IHC assay, after deparaffinization, rehydration, antigen retrieval, and non-specific signal blocking, the tissue sections were incubated with the primary antibody at 4 °C overnight. Then the tissue sections were added with HRP-labeled secondary antibody for 1 h at room temperature. DAB was used to visualize these tissue sections and hematoxylin was used to counterstain. Finally, the stained tissue sections were scanned by KF-PRO-020 Digital Pathological Slides Scanner. Analysis was performed by Image J software as we previously reported. The expression level of 4-HNE, GPX4 and Ki-67 was evaluated by the mean intensity of positive cells in 5 randomly selected residual tumor fields.

### Statistical analysis

All statistical analysis was performed using GraphPad Prism 8.0 or SPSS 20.0 software. Differences in the average between two groups with one variation were determined by Student’s *t* test. Differences in the average between more than two groups with one variation were determined by one-way analysis of variance (ANOVA). Differences between groups with dose factors or with more than one variation were determined by two-way ANOVA. Survival was analyzed using the Log-rank test.

## Supplementary information

Supplementary Information

Uncut WB Blot image

Raw data

## Data Availability

All data are available within the article, supplementary information, or available from the corresponding author upon reasonable request.
