# Supplementary material for: Targeting the TRIM14/USP14 axis enhances radiotherapy efficacy by inducing GPX4 degradation and disrupting ferroptotic defense in HCC
Source: Cell Death Dis. 2025 Jul 1;16(1):481. doi: 10.1038/s41419-025-07807-6 (PMC12219831; doi:10.1038/s41419-025-07807-6)
Supplement: Supplementary file 1 — Supplementary Information [file 41419_2025_7807_MOESM1_ESM.pdf]

Supplementary Information

Targeting the TRIM14/USP14 axis enhances radiotherapy efficacy by inducing GPX4 degradation and disrupting ferroptotic defense in HCC

Xin Yue, Zhen Xiang, Yang Yi, Xuecen Wang, Weilin Zhou, Weijian Wu, Wenjing Qin, Yuxuan Zhao, Xianzhang Bu, and Zhenwei Peng

Correspondence: yuex2504@jnu.edu.cn (X.Y.), phsbxzh@mail.sysu.edu.cn (X.B.) and pzhenw@mail.sysu.edu.cn (Z.P.).

This file including:

Supplemental Figures

Supplemental Tables

Supplemental Methods

Materials Source Table

Supplementary Figures

Figure S1

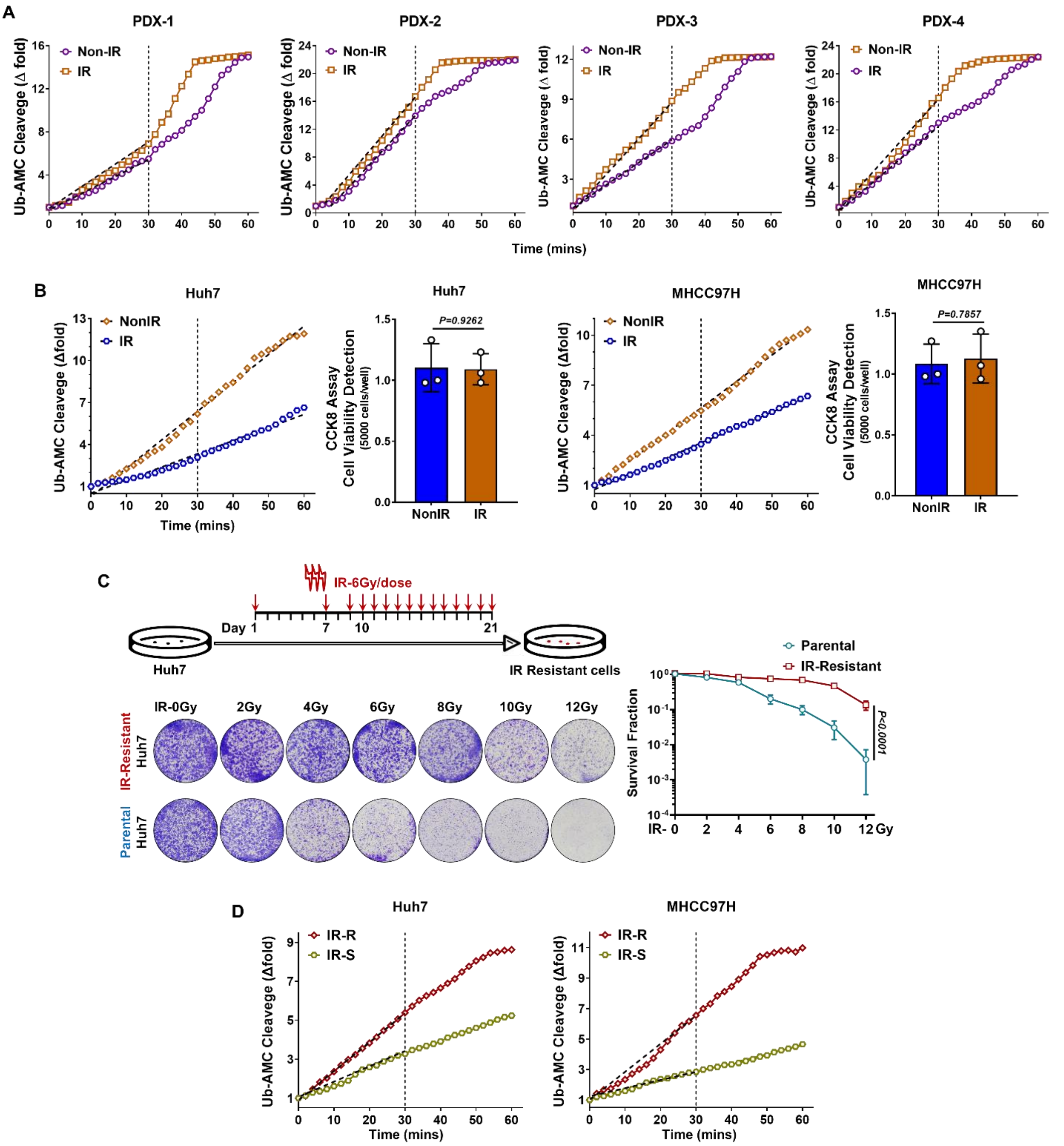

Figure S1. Elevation of intracellular deubiquitylation contributes to radioresistance in HCC. (**A-B**) Ub-AMC hydrolysis assay was used to examine deubiquitinating activities in (**A**) PDX 1-4 models and (**B**) Huh7 and MHCC97H cells treated IR or not, left panel: Hydrolysis kinetics curve; right panel: Cell viability analysis, **related to Figs.1B-C**. (**C**) (Upper) Treatment schemes of

the generation of IR resistant cells. (Below) Colony formation assays in parental and IR-resistant Huh7 cells treated with a single dose of 0, 2, 4, 6, 8, 10, 12 Gy IR respectively. (Left) Representative images. (Right) Survival curves, shown as Means ± SD. from 3 independent experiments (two-way ANOVA), **related to Fig. 1D**. (**D**) Ub-AMC hydrolysis assay was used to examine deubiquitinating activities in IR-R/IR-S Huh7 and MHCC97H cells. IR-R, IR-resistant; IR-S, IR-sensitive, **related to Fig. 1D**.

Figure S2

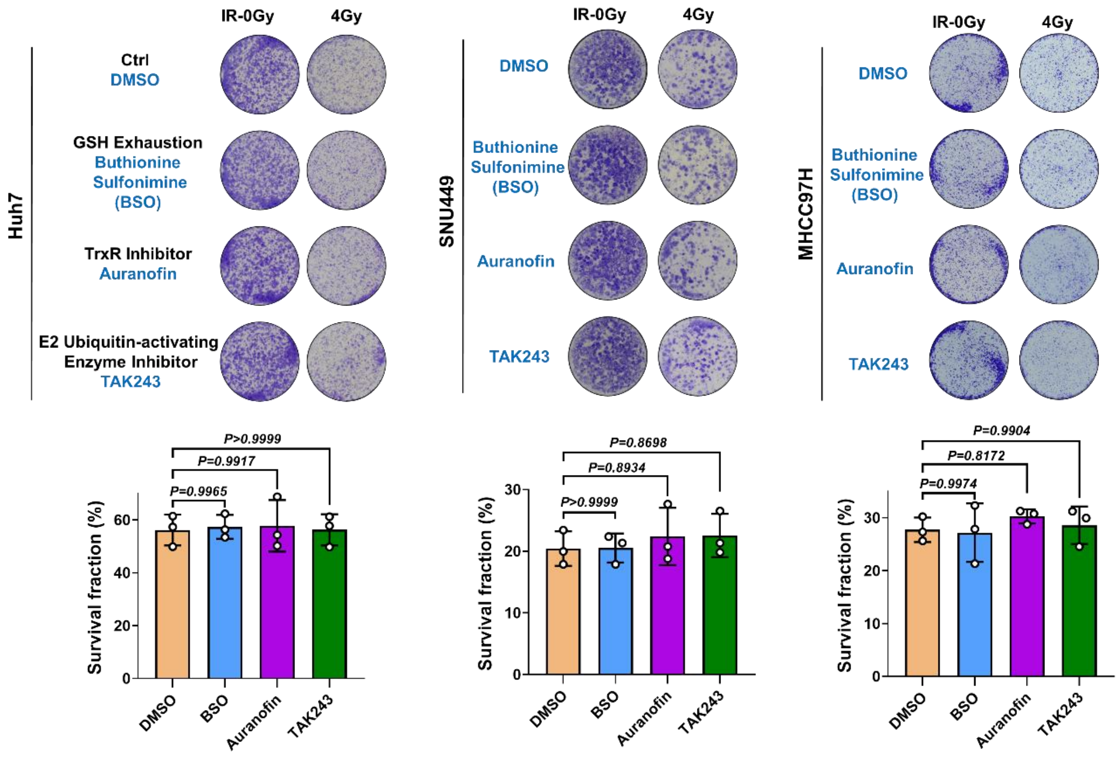

Figure S2. Colony formation assays in Huh7/SNU449/MHCC97H cells pretreated with BSO (25 μM), Auranofin (10 μM) or TAK243 (1 μM) for 12 h and subsequently exposed to 4 Gy IR. DMSO as a control. (Left) Representative images. (Right) Survival curves. Results are representative of three independent experiments. Data are represented as mean ± S.D. Two-tailed Student's t test

(2-sample t test) was used for comparisons of the indicated two groups.

Figure S3

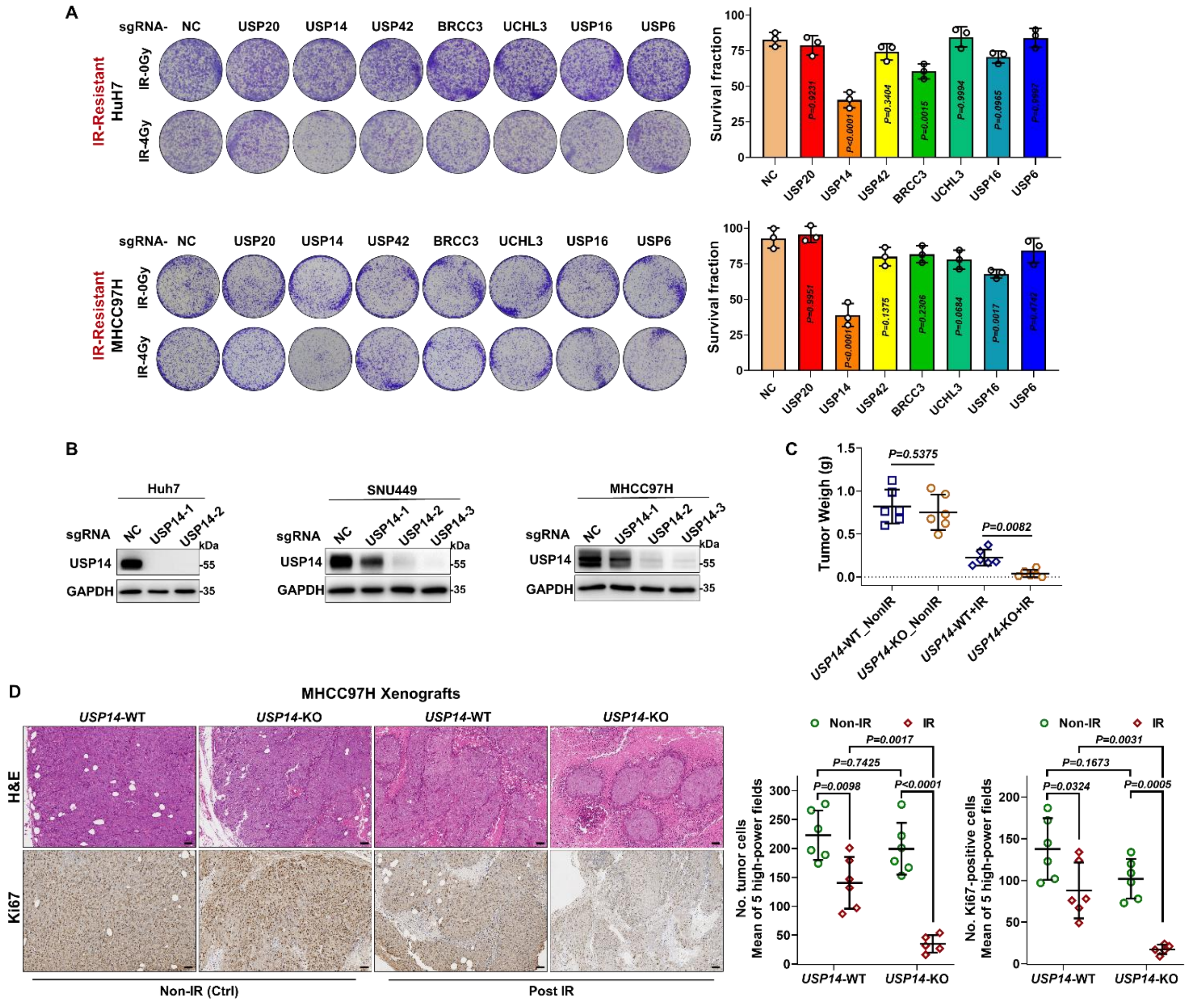

Figure S3. USP14 deficiency of HCC cells confers sensitivity to RT *in vitro* and *in vivo*. (**A**) Colony formation assays in (upper) Huh7 and (below) MHCC97H cells (including USP20/USP14/USP42/BRCC3/UCHL3/USP16/USP6-KO) groups treated with a single dose of 0, 2, 4, 6, or 8 Gy IR. Representative images and survival curves are shown. Experiments were performed in triplicate, and data are represented as mean ± SD. Statistical significance was determined by two-way ANOVA. (**B**) WB analysis showing the validation of the USP14-1/2/3 sgRNAs in Huh7, SNU449 and MHCC97H cells. (**C**) Quantitative analysis of tumor weight in USP14-WT or KO MHCC97H xenografts were treated with IR (2 Gy/day for 5 times every other day) or not, **related to Figs.1P-Q**.

(**D**) H&E staining in USP14-WT/USP14-KO MHCC97H xenografts treated with IR or not: (Left) Representative images. (Right) The mean number of tumor cells and Ki-67-positive cells/5 high-power fields, scale bars, 10 μm, **related to Figs.1P-Q**. Results are representative of three independent experiments. Data are represented as mean ± S.D. (**C**) and (**D**): one-way ANOVA was used for comparisons of the indicated groups in bar graphs.

Figure S4

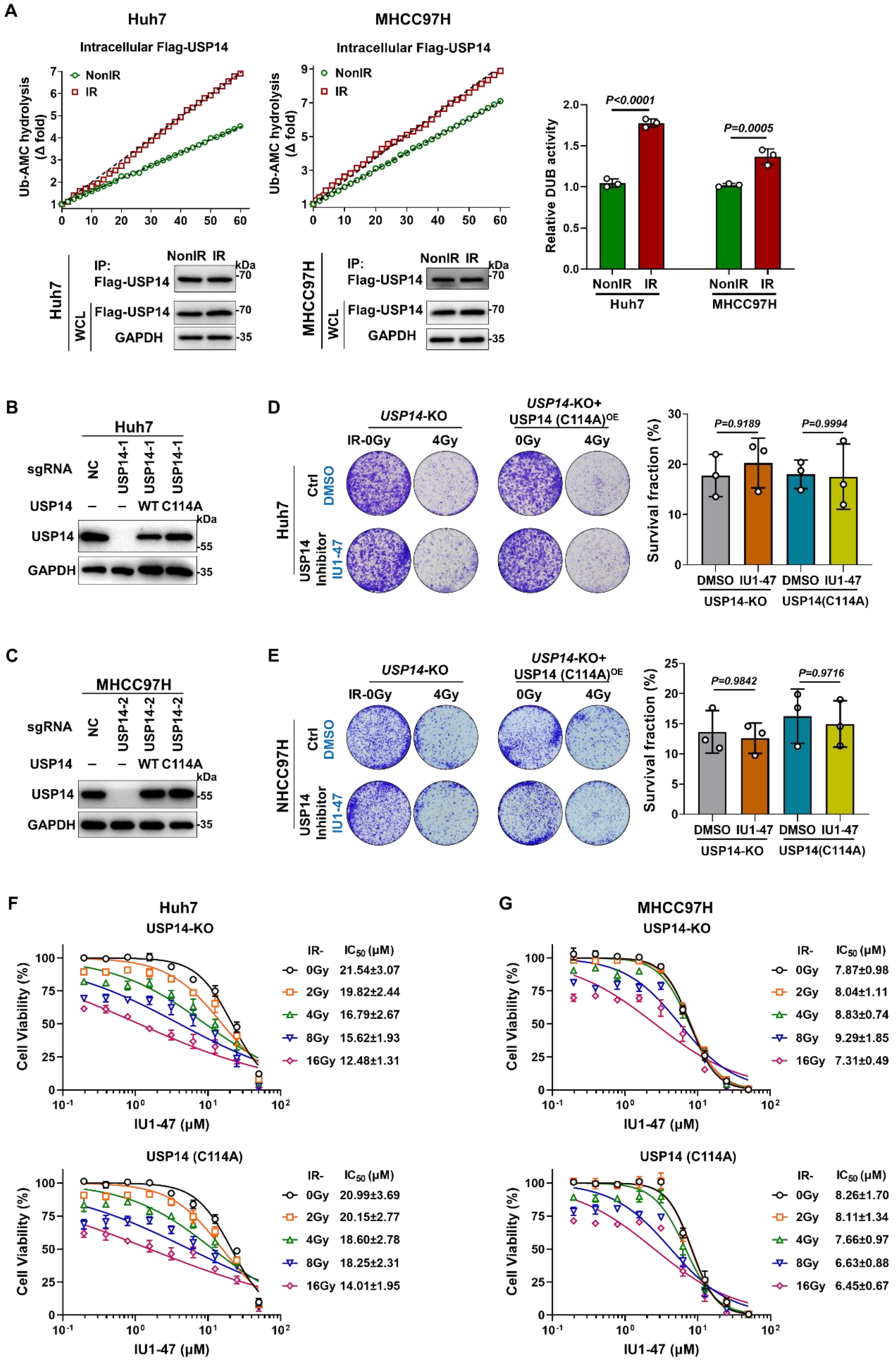

A
Huh7
MHCC97H
Intracellular Flag-USP14
Ub-AMC hydrolysis (Δ fold)
NonIR
IR
IP: Flag-USP14
WCL
Flag-USP14
GAPDH
kDa
Relative DUB activity
P<0.0001
P=0.0005
B
C
sgRNA
NC
USP14-1
USP14-2
USP14
WT
C114A
D
E
USP14-KO
USP14-KO+ USP14 (C114A)OE
IR-0Gy
4Gy
0Gy
Ctrl DMSO
USP14 Inhibitor IU1-47
NHCC97H
Survival fraction (%)
P=0.9189
P=0.9994
P=0.9842
P=0.9716
DMSO
IU1-47
USP14(C114A)
F
G
Cell Viability (%)
IU1-47 (μM)
USP14 (C114A)
IR-
IC50 (μM)
0Gy 21.54±3.07
2Gy 19.82±2.44
4Gy 16.79±2.67
8Gy 15.62±1.93
16Gy 12.48±1.31
0Gy 20.99±3.69
2Gy 20.15±2.77
4Gy 18.60±2.78
8Gy 18.25±2.31
16Gy 14.01±1.95
0Gy 7.87±0.98
2Gy 8.04±1.11
4Gy 8.83±0.74
8Gy 9.29±1.85
16Gy 7.31±0.49
0Gy 8.26±1.70
2Gy 8.11±1.34
4Gy 7.66±0.97
8Gy 6.63±0.88
16Gy 6.45±0.67

Figure S4. Loss of the deubiquitinating activity of USP14 confers HCC radiosensitization. (**A**) (Left) Ub-AMC hydrolysis assay was used to examine deubiquitinating activities of purified Flag-USP14 in Huh7 and MHCC97H cells expressing and treated with or without IR. (Upper) Representative images. (Below) Co-IP and WB analyses for detecting Flag-USP14 in Huh7 and MHCC97H cells transfected with Flag-USP14 followed by IR treatment or not. (Right) Relative DUB activity in Huh7 and MHCC97H cells after Co-IP with Flag-USP14. (**B-C**) WB analysis showing the effects of USP14-KO cells rescued by USP14-WT and USP14-C114A in (**B**) Huh7 and (**C**) MHCC97H cells, **related to Figs. 2A-B**. (**D-E**) Colony formation assays in USP14-KO and USP14 (C114A) mutation expression of (**D**) Huh7 and (**E**) MHCC97H cells pretreated with IU1-47 (5 μM) for 12 h and subsequently exposed to 4 Gy IR. DMSO as a control. (Left) Representative images. (Right) Survival curves. (**F-G**) The effect of IU1-47 on (**F**) USP14-KO and USP14 (C114A) mutation expression of Huh7 and (**G**) USP14-KO and USP14 (C114A) mutation of MHCC97H cells proliferation inhibitory activity after treated with IR (0, 2, 4, 6, 8 Gy respectively). The IC_{50} was calculated by normalizing cell viability to the radiation-only control group (without IU1-47), which was set as 100% survival. Results are representative of three independent experiments. Data are represented as mean ± S.D. (**A**) and (**D-E**) Two-tailed Student's t test (2-sample t test) was used for comparisons of the indicated two groups.

Figure S5

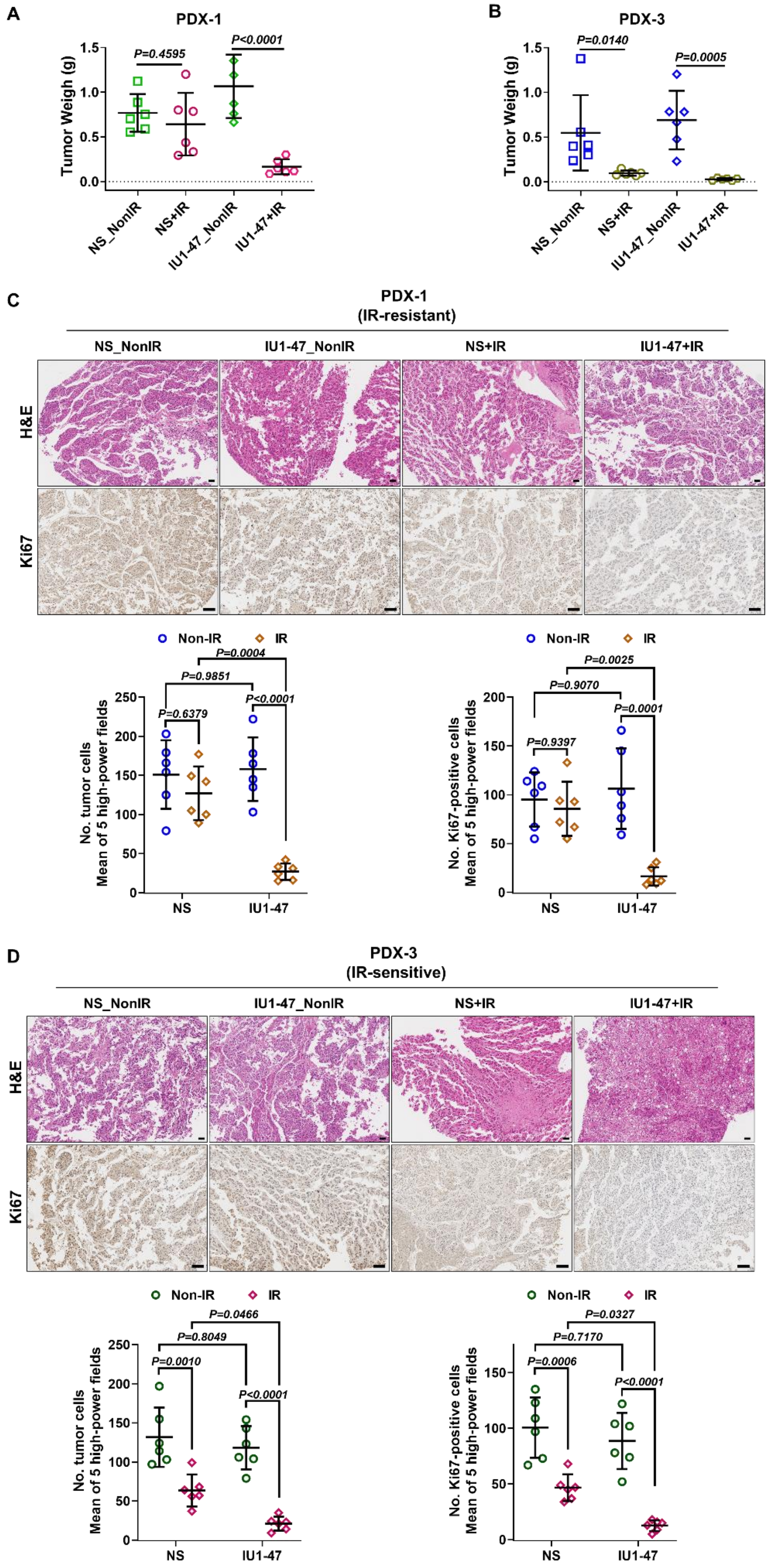

A
PDX-1
Tumor Weigh (g)
P=0.4595
P<0.0001
NS_NonIR
NS+IR
IU1-47_NonIR
IU1-47+IR
B
PDX-3
P=0.0140
P=0.0005
C
PDX-1
(IR-resistant)
H&E
Ki67
Non-IR
IR
P=0.0004
P=0.9851
P=0.6379
P<0.0001
No. tumor cells
Mean of 5 high-power fields
NS
IU1-47
P=0.0025
P=0.9070
P=0.9397
P=0.0001
No. Ki67-positive cells
D
PDX-3
(IR-sensitive)
P=0.0466
P=0.8049
P=0.0010
P<0.0001
P=0.0327
P=0.7170
P=0.0006

Figure S5. Inhibition of the deubiquitinating activity of USP14 confers HCC sensitivity to radiation *in vivo*. (**A-B**) Quantitative analysis of tumor weight in PDX-1 and PDX-3 models were treated with IU1-47 (5 mg/kg/day for 10 times， NS (normal saline) as a control) and IR (2 Gy/day for 5 times every other day), **related to Figs. 2G-J**. (**C-D**) Histopathological examination in PDX-1 (resistant)/ PDX-3 (sensitive) models. (Left) Representative images. (Right) The mean number of tumor cells and Ki67-positive cells/5 high-power fields, scale bars, 5 μm, **related to Figs. 3L-Q**. Results are representative of three independent experiments. Data are represented as mean ± S.D. (**A-B**): Two-tailed Student's t test (2-sample t test) was used for comparisons of the indicated two groups. (**C-D**) right: one-way ANOVA was used for comparisons of the indicated groups.

Figure S6

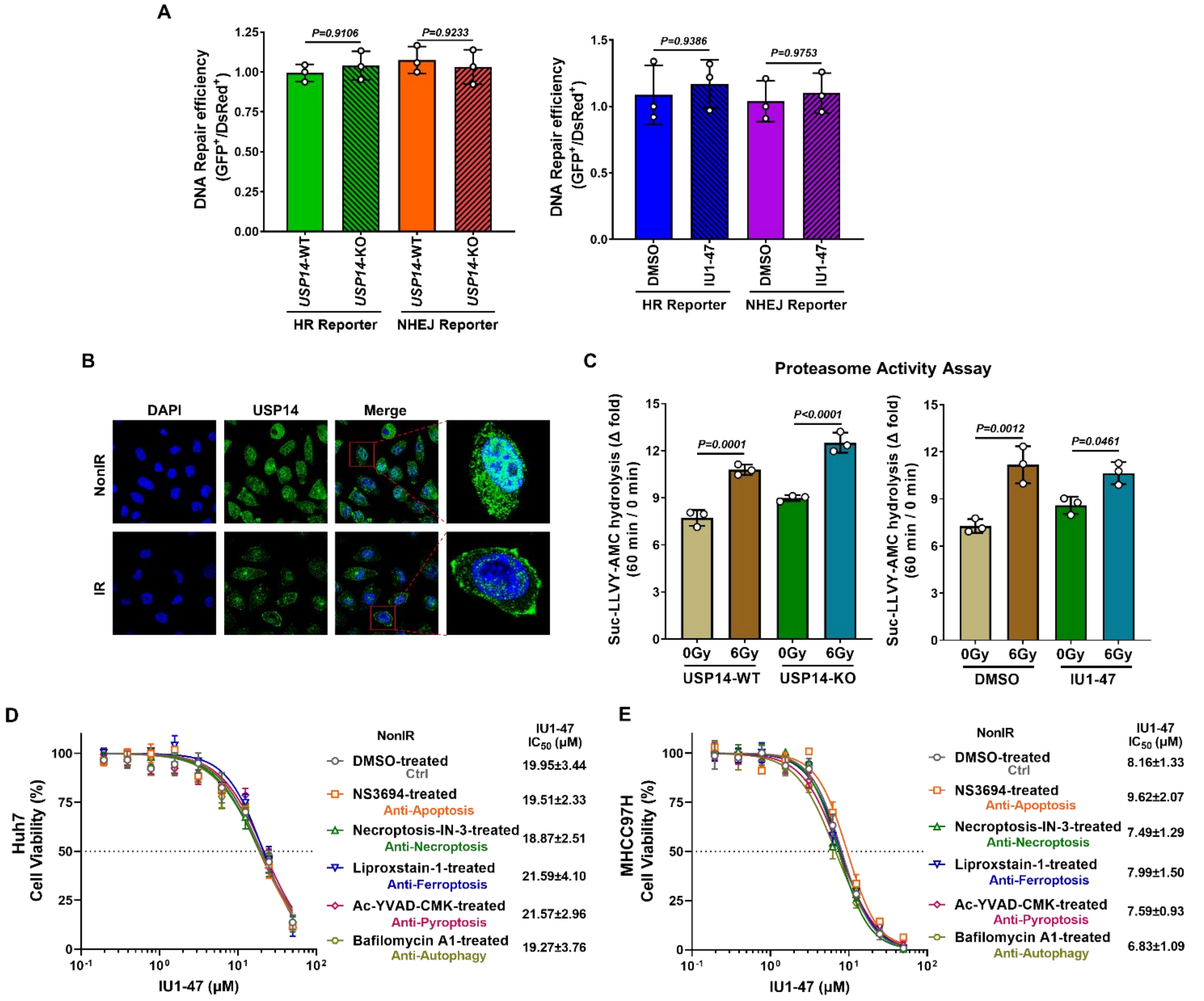

Figure S6. USP14 confers radioresistance in HCC by impeding ferroptosis triggered by IR. (**A**) DNA repair efficiency assays with HR/NHEJ reporter in (Left) USP14-WT and USP14-KO Huh7 cells, (Right) Huh7 cells treated with IU1-47 for 12 h (DMSO as a vehicle control). (**B**) Immunofluorescence analysis of distribution of USP14 in Huh7 cells treated with a single dose of 6 Gy. (**C**) Proteasome activity assay in (Left) USP14-WT/USP14-KO Huh7 cells and (Right) Huh7 cells treated with IU1-47 for 12 h (DMSO as a vehicle control). (**D-E**) Cell viability assays using CCK-8 in (**D**) Huh7 and (**E**) MHCC97H cells treated with IU1-47 at the indicated concentrations in combination with NS3694, Necroptosis-IN-3, Liproxstain-1, Ac-YVAD-CMK or Bafilomycin A1 respectively, **related to Figs. 3A-B**. The IC_{50} was calculated by normalizing cell viability to the

drug-pretreated control group (without IU1-47), which was set as 100% survival. Results are representative of three independent experiments. Data are represented as mean ± S.D. (**A**), (**C**) Two-tailed Student's t test (2-sample t test) was used for comparisons of the indicated two groups.

Figure S7

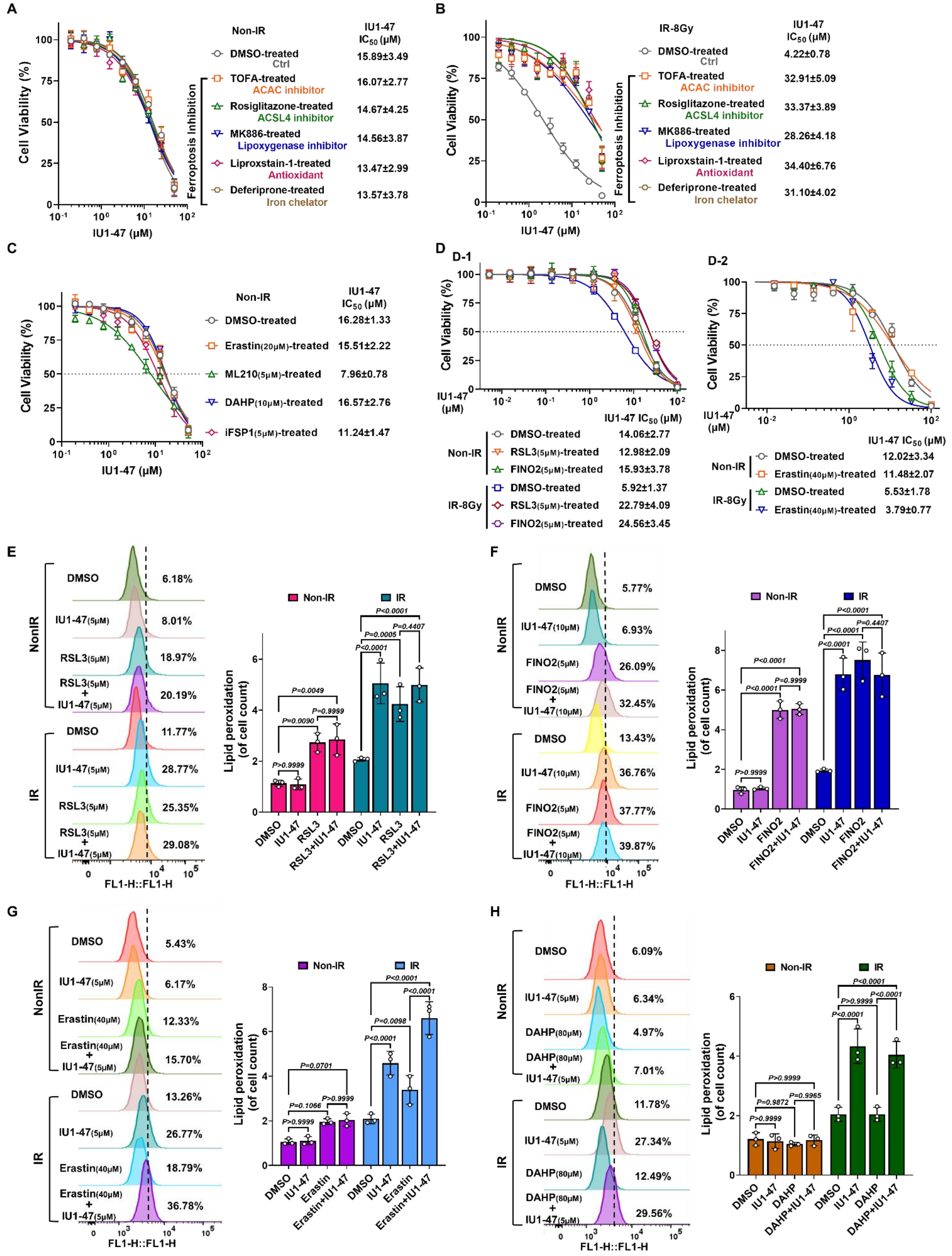

A
Non-IR
IU1-47 IC50 (μM)
DMSO-treated Ctrl 15.89±3.49
TOFA-treated ACAC inhibitor 16.07±2.77
Rosiglitazone-treated ACSL4 inhibitor 14.67±4.25
MK886-treated Lipoxygenase inhibitor 14.56±3.87
Liproxstain-1-treated Antioxidant 13.47±2.99
Deferiprone-treated Iron chelator 13.57±3.78
Ferroptosis Inhibition
Cell Viability (%)
IU1-47 (μM)
B
IR-8Gy
IU1-47 IC50 (μM)
DMSO-treated Ctrl 4.22±0.78
TOFA-treated ACAC inhibitor 32.91±5.09
Rosiglitazone-treated ACSL4 inhibitor 33.37±3.89
MK886-treated Lipoxygenase inhibitor 28.26±4.18
Liproxstain-1-treated Antioxidant 34.40±6.76
Deferiprone-treated Iron chelator 31.10±4.02
C
Non-IR
IU1-47 IC50 (μM)
DMSO-treated 16.28±1.33
Erastin(20μM)-treated 15.51±2.22
ML210(5μM)-treated 7.96±0.78
DAHP(10μM)-treated 16.57±2.76
iFSP1(5μM)-treated 11.24±1.47
D
D-1
IU1-47 IC50 (μM)
Non-IR DMSO-treated 14.06±2.77
RSL3(5μM)-treated 12.98±2.09
FINO2(5μM)-treated 15.93±3.78
IR-8Gy DMSO-treated 5.92±1.37
RSL3(5μM)-treated 22.79±4.09
FINO2(5μM)-treated 24.56±3.45
D-2
IU1-47 IC50 (μM)
Non-IR DMSO-treated 12.02±3.34
Erastin(40μM)-treated 11.48±2.07
IR-8Gy DMSO-treated 5.53±1.78
Erastin(40μM)-treated 3.79±0.77
E
NonIR
DMSO 6.18%
IU1-47(5μM) 8.01%
RSL3(5μM) 18.97%
RSL3(5μM) + IU1-47(5μM) 20.19%
IR
DMSO 11.77%
IU1-47(5μM) 28.77%
RSL3(5μM) 25.35%
RSL3(5μM) + IU1-47(5μM) 29.08%
FL1-H::FL1-H
Non-IR
IR
Lipid peroxidation (of cell count)
P>0.9999
P=0.0090
P=0.0049
P=0.9999
P<0.0001
P=0.0005
P=0.4407
P<0.0001
DMSO
IU1-47
RSL3
RSL3+IU1-47
F
NonIR
DMSO 5.77%
IU1-47(10μM) 6.93%
FINO2(5μM) 26.09%
FINO2(5μM) + IU1-47(10μM) 32.45%
IR
DMSO 13.43%
IU1-47(10μM) 36.76%
FINO2(5μM) 37.77%
FINO2(5μM) + IU1-47(10μM) 39.87%
FL1-H::FL1-H
Non-IR
IR
Lipid peroxidation (of cell count)
P>0.9999
P<0.0001
P<0.0001
P=0.9999
P<0.0001
P<0.0001
P<0.0001
P=0.4407
DMSO
IU1-47
FINO2
FINO2+IU1-47
G
NonIR
DMSO 5.43%
IU1-47(5μM) 6.17%
Erastin(40μM) 12.33%
Erastin(40μM) + IU1-47(5μM) 15.70%
IR
DMSO 13.26%
IU1-47(5μM) 26.77%
Erastin(40μM) 18.79%
Erastin(40μM) + IU1-47(5μM) 36.78%
FL1-H::FL1-H
Non-IR
IR
Lipid peroxidation (of cell count)
P>0.9999
P=0.1066
P=0.0701
P>0.9999
P<0.0001
P=0.0098
P<0.0001
P<0.0001
DMSO
IU1-47
Erastin
Erastin+IU1-47
H
NonIR
DMSO 6.09%
IU1-47(5μM) 6.34%
DAHP(80μM) 4.97%
DAHP(80μM) + IU1-47(5μM) 7.01%
IR
DMSO 11.78%
IU1-47(5μM) 27.34%
DAHP(80μM) 12.49%
DAHP(80μM) + IU1-47(5μM) 29.56%
FL1-H::FL1-H
Non-IR
IR
Lipid peroxidation (of cell count)
P>0.9999
P=0.9872
P>0.9999
P=0.9965
P<0.0001
P>0.9999
P<0.0001
P<0.0001
DMSO
IU1-47
DAHP
DAHP+IU1-47

Figure S7. USP14 regulates ferroptosis defense through GPX4 after RT. (**A-B**) The effect of IU1-47 on Huh7 cells proliferation inhibitory activity in combination with TOFA (ACAC inhibitor, 20 μM), Rosiglitazone (ACSL4 inhibitor, 10 μM), MK886 (Lipoxygenase inhibitor, 10 μM), Liproxstain-1 (Antioxidant, 20 μM) or Deferiprone (Iron chelator, 20 μM) for 12 h before (**A**) Non-IR / (**B**) 8 Gy IR. Cell viability detection at 72 h after IR. (**C**) The effect of IU1-47 on Huh7 cells proliferation inhibitory activity in combination with Erastin (20 μM), ML210 (5 μM), DAHP (10 μM) or iFSP1 (5 μM) treatment, Cell viability detection at 84 h after adding drugs, **related to Fig. 4B**. (**A-C**) The IC_{50} was calculated by normalizing cell viability to the drug-pretreated control group (without IU1-47), which was set as 100% survival. (**D**) Evaluation of IU1-47 on inhibiting Huh7 cell proliferation in conjunction with RSL3, FINO2 (**D-1**) or Erastin (**D-2)**, followed by 8 Gy IR. Viability was assessed 72 hours post-IR, non-IR groups as control. The IC_{50} was calculated by normalizing cell viability to the drug-pretreated and IR control group or drug-pretreated control group (without IU1-47), which was set as 100% survival. (**E-H**) Lipid peroxidation levels were quantified by BODIPY-581/591-C11 staining in Huh7 cells pretreated with IU1-47 alongside RSL3 (**E**), FINO2 (**F**), Erastin (**G**), or DAHP (**H**) for 12 hours prior to IR exposure. Lipid peroxidation levels in these groups are also shown. Results are representative of three independent experiments. Data are represented as mean ± S.D. (**E-H**): One-way ANOVA was used for comparisons of the indicated groups.

Figure S8

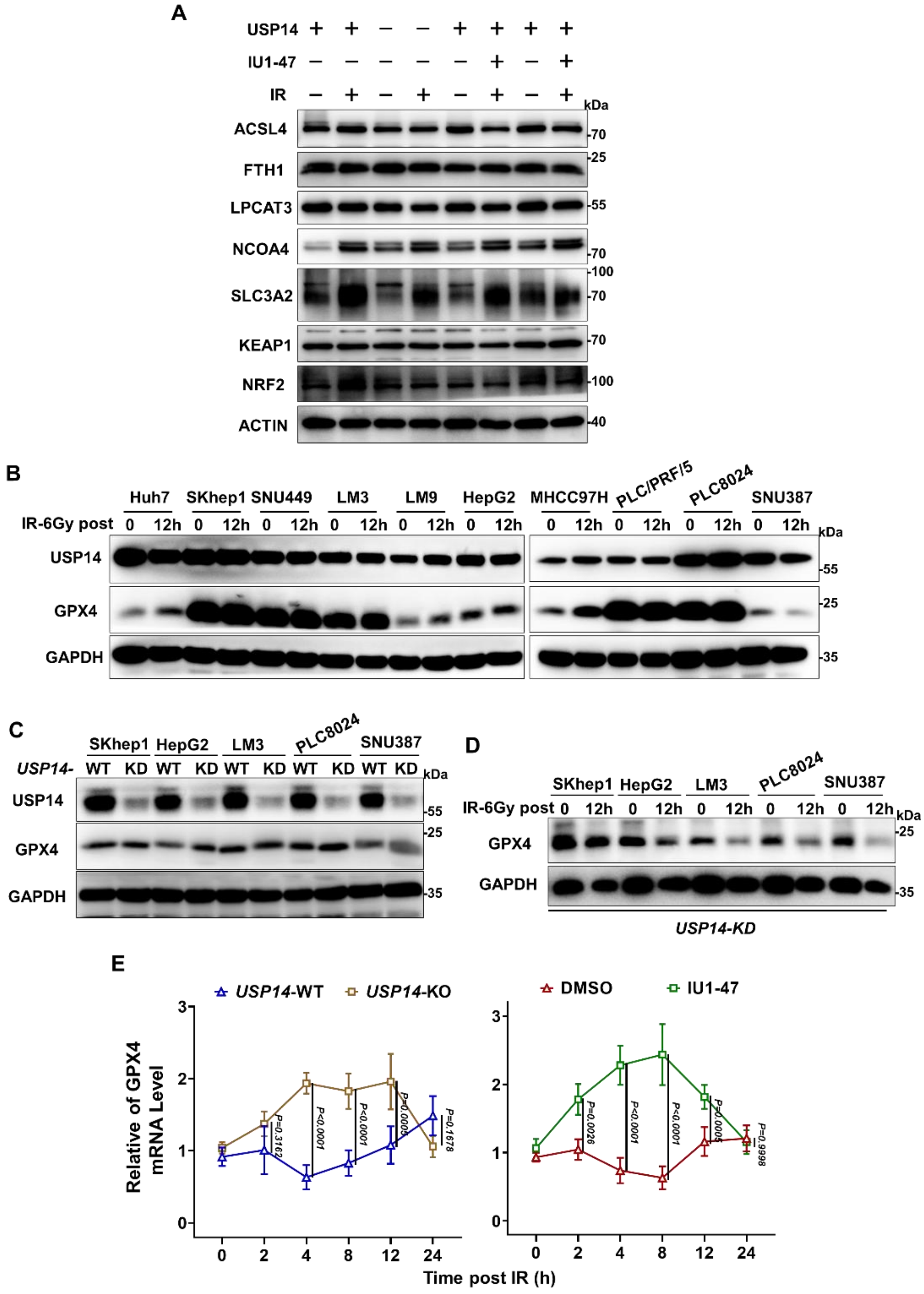

Figure S8. (**A**) WB assay for protein levels of ACSL4, FTH1, LPCAT3, NCOA4, SLC3A2, KEAP1, NRF2 in Huh7 cells with or without USP14 expression at 12 h after 6 Gy IR treatment; and in Huh7 cells pretreated with IU1-47 (5 μM) for 2 h (DMSO as a control) followed by 6 Gy IR treatment (post 12 h). Non-IR groups as control, Actin as protein loading control, **related to Fig. 4D**. (**B-D**) Western Blot analysis for proteins USP14 and GPX4 in 10 HCC cell lines, (**B**) USP14 and GPX4

levels in 10 HCC cell lines at 12 hours after 6 Gy IR; (**C**) GPX4 levels under non-irradiated conditions with/without USP14 expression; (**D**) GPX4 levels in USP14-KD HCC cell lines at 12 hours after 6 Gy IR. (**E**) GPX4 mRNA levels were performed by Q-PCR in GPX4-WT/GPX4-KO Huh7 cells pretreated with IU1-47 (5 μM) for 2 hours before 6 Gy IR exposure. Cells were collected at the indicated time post IR. Results are indicative of three independent experiments, expressed as mean ± S.D., statistical significance was determined by two-way ANOVA.

Figure S9

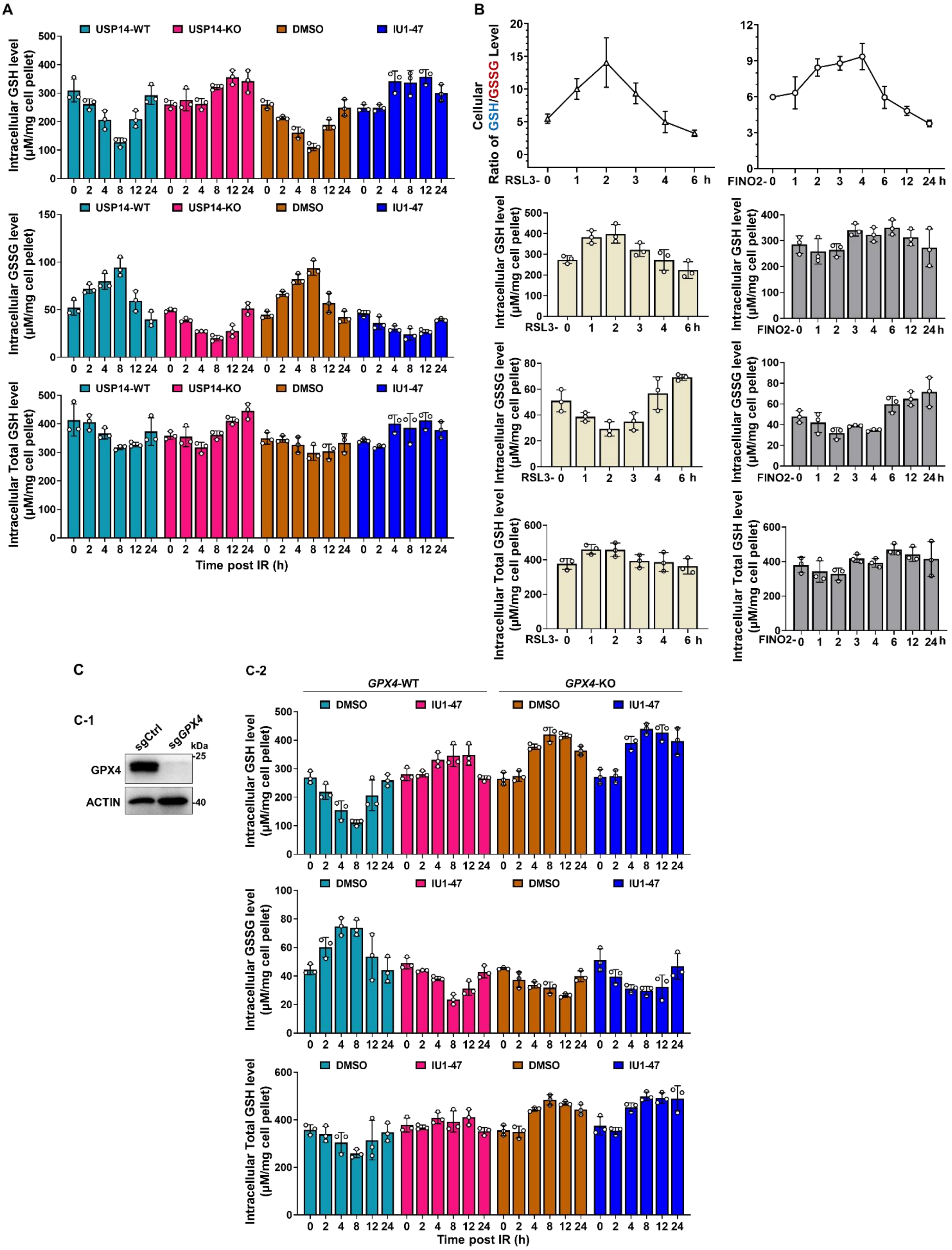

Figure S9. Intracellular GSH, GSSG and Total GSH levels. (**A**) Intracellular GSH, GSSG and

Total GSH levels in USP14-WT/USP14-KO Huh7 cells exposed to IR (6 Gy) or cells exposed to IU1-47 (5 μM) for 2 h before 6 Gy IR treatment, DMSO as control. Cells were collected at the indicated time points after IR, **related to Fig. 4I**. (**B**) Intracellular GSH/GSSG ratio, GSH, GSSG and Total GSH levels in Huh7 cells treated with RSL3 (5 μM, left) and FINO2 for the indicated time points. (**C-1**) WB analysis showing the validation of the GPX4 sgRNAs in Huh7 cells. (**C-2**) Intracellular GSH, GSSG and Total GSH levels in GPX4-WT/GPX4-KO Huh7 cells pretreated with IU1-47 (5 μM) for 2 h and then exposed to 6 Gy IR. Cells were collected at the indicated time points after IR, **related to Fig. 4M**. Results are representative of three independent experiments. Data are represented as mean ± S.D.

Figure S10

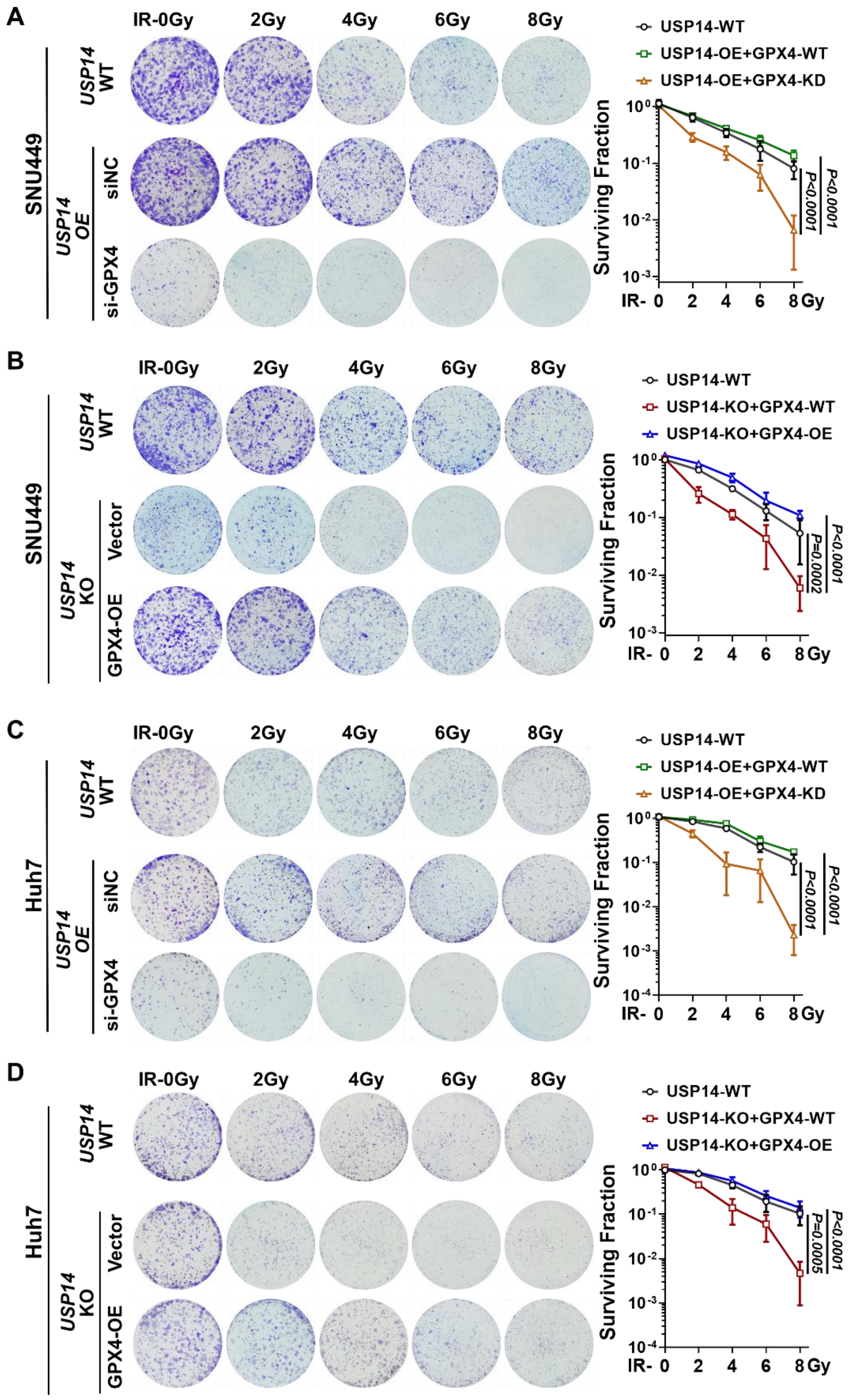

Figure S10. Radiosensitivity detection in two models: GPX4 depletion in USP14-OE cells and Ectopic expression of GPX4 in USP14-KO cells. (**A/C**) Colony formation assays of GPX4-KD in USP14-OE SNU449 (**A**)/Huh7 (**C**) cells treated with a single dose of 0, 2, 4, 6, 8 Gy IR respectively.

(**B/D**) Colony formation assays of GPX4-OE in USP14-KO SNU449 (**B**)/Huh7 (**D**) cells treated with a single dose of 0, 2, 4, 6, 8 Gy IR respectively. (Left) Representative images. (Right) Survival curves, shown as Means ± SD. from 3 independent experiments (two-way ANOVA).

Figure S11

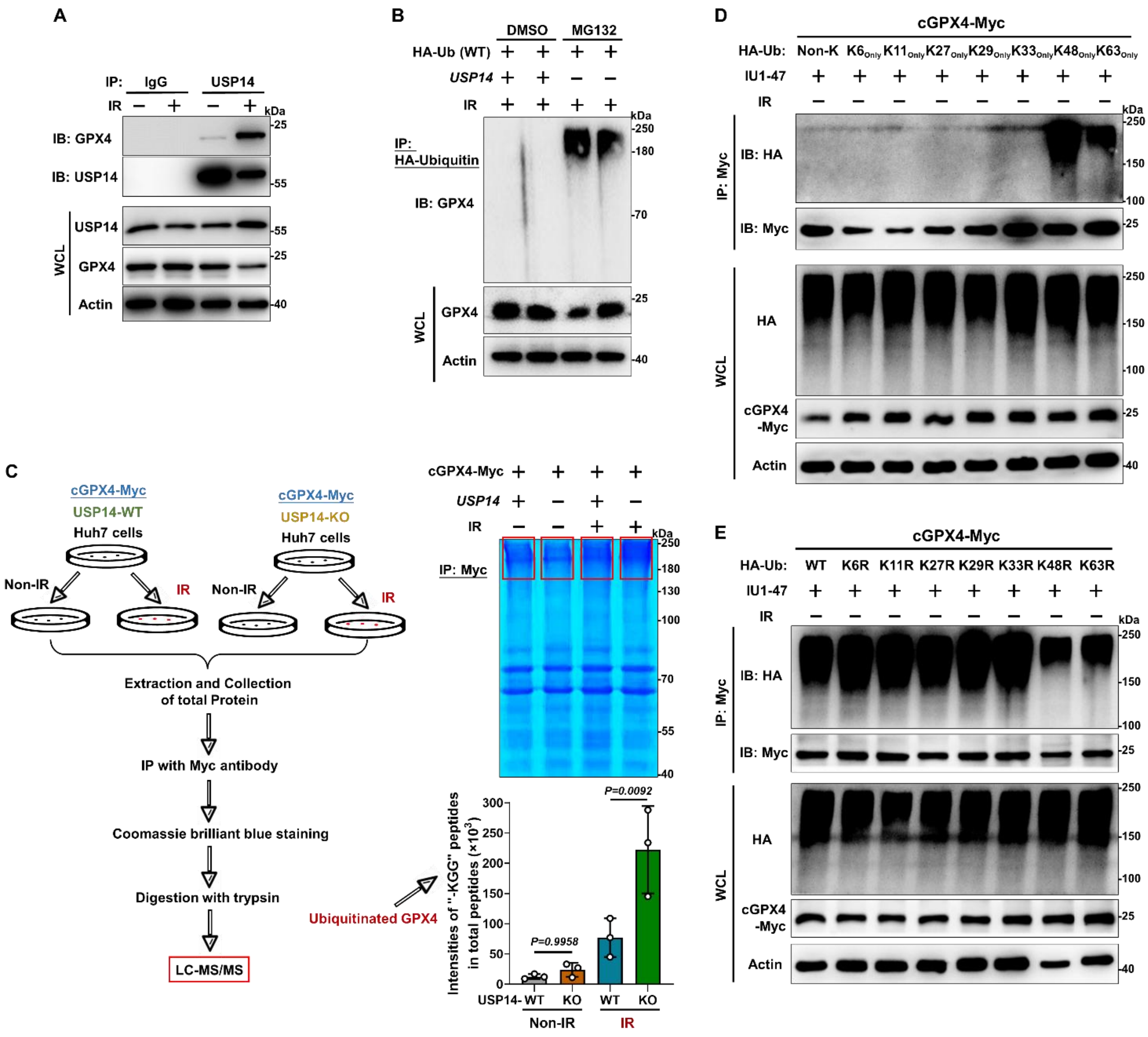

Figure S11. Deubiquitination of GPX4 by USP14 in response to IR. (**A**) Co-IP and WB assays were used to detect the interaction between endogenous USP14 and GPX4 in Huh7 cells treated with 6 Gy IR, Cells were collected at 8 h after IR. Non-IR groups as a control, **related to Fig. 5A**. (**B**)

Co-IP and WB were used to determine the ubiquitination levels of GPX4 in USP14-WT/KO Huh7 cells expressing wild-type (WT) HA-ubiquitin, pre-treated with MG132 (5 μM) or DMSO as a control for 2 hours prior to 6 Gy IR. Cells were collected 8 hours post-IR, with untreated samples as controls. (**C**) In Huh7 cells (either USP14-WT or USP14-KO), Myc-tagged cGPX4 was overexpressed. Cells were exposed to 6 Gy of IR and harvested 8 hours post-IR. cGPX4-Myc was IP using an anti-Myc antibody, with non-irradiated cells serving as controls. IP protein samples were separated by electrophoresis and Coomassie-stained. High-molecular-weight regions (boxed) were excised for gel extraction, followed by MS analysis (Left and Right-top). Bottom-right panel shows MS-based quantification of KGG-modified peptide abundance (Right-bottom). (**D-E**) The ubiquitin linkage type on GPX4 was identified using Co-IP and WB. In conditions (**D**) and (**E**), Huh7 cells expressed HA-ubiquitin mutants with selective lysine preservation (K-only, with other lysines mutated to arginine) or a singular lysine-to-arginine mutation, respectively. Cells were treated with IU1-47 or DMSO as a control for 8 hours and harvested, **related to Figs. 5G-H**. All results are indicative of three independent experiments.

Figure S12

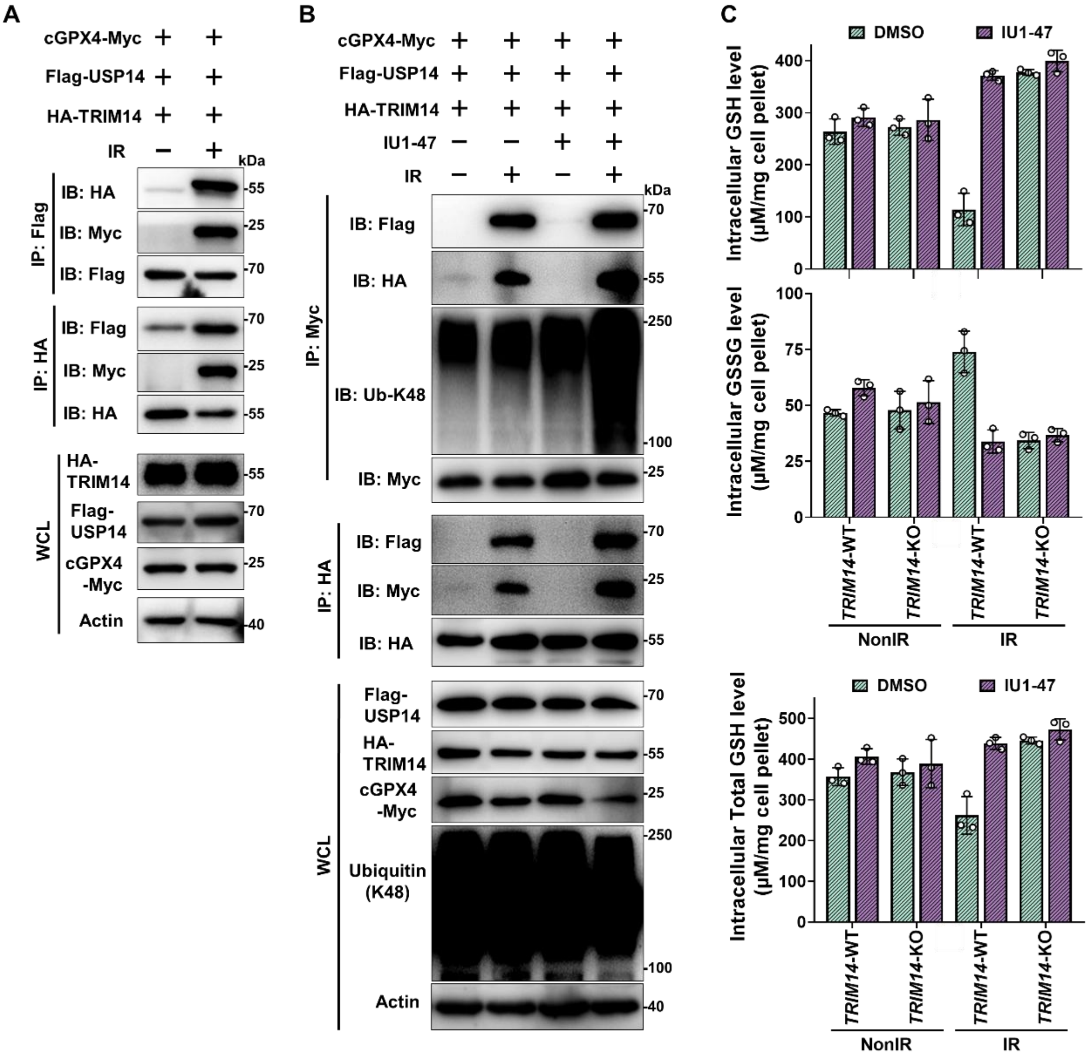

Figure S12. USP14 targets GPX4 in a TRIM14-dependent manner. (**A**) Co-IP and WB assay for the relationships between GPX4 and TRIM14 or GPX4 and USP14. Huh7 cells expressing cGPX4-Myc with Flag-USP14 or HA-TRIM14 were exposed to by 6 Gy IR. Cells were collected at 6 h after IR. Non-IR groups as a control, **related to Fig. 6A**. (**B**) Co-IP and WB assays for the effect of TRIM14/USP14/GPX4 complex in presence of IU1-47. Huh7 cells expressing Flag-USP14, HA-TRIM14 and cGPX4-Myc were pretreated with IU1-47 for 2 h, these cells were exposed to 6 Gy IR before collecting cell lysis at 6 h. (**C**) Intracellular GSH, GSSG and Total GSH levels in TRIM14-WT/TRIM14-KO Huh7 cells pretreated with IU1-47 (5 μM) for 2 h and then exposed to 6 Gy IR. Cells were collected at 4 h after IR, **related to Fig. 6F**.

Figure S13

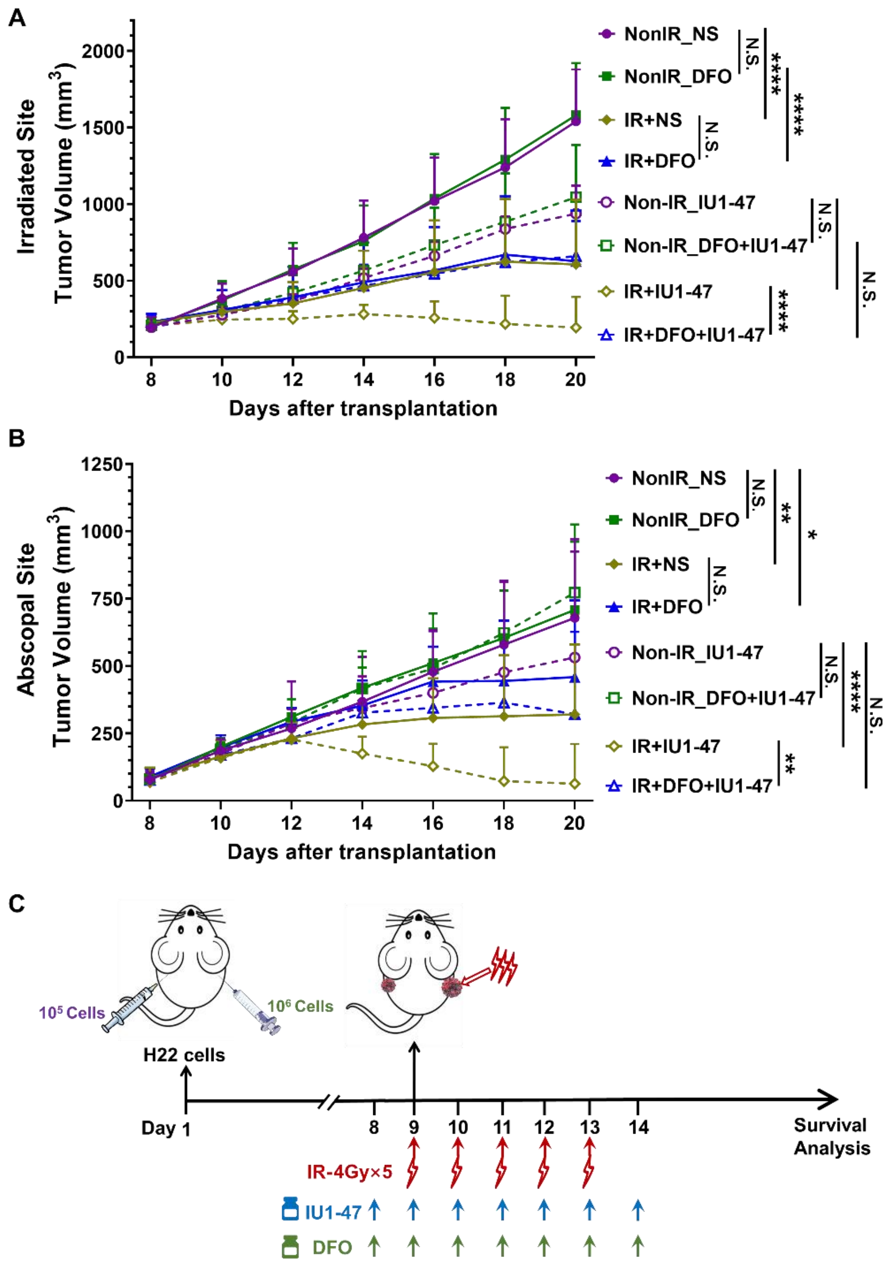

Figure S13. Inhibition of USP14 with IU1-47 enhances radiotherapeutic abscopal effect via inducing ferroptosis. Tumor growth trajectories for irradiated (IR) and non-irradiated (Non-IR) mice administered IU1-47 or (and) DFO alongside vehicle (NS, Normal Saline), divided into the 8 groups (n=6/group) denoted in the figure. (**A**) Irradiated tumor site; (**B**) Abscopal tumor site, **related to Fig. 7D**. Statistical significance was assessed via two-way ANOVA (n.s., not significant; *, P<0.05, **, P<0.01, ***, P<0.001, ****, P<0.0001). (**C**) Kaplan-Meier survival analyses of treated

mice, schematic representation of the experimental setup demonstrating the abscopal effect of RT on tumor-bearing mice (H22 tumors implanted bilaterally in the axillae) subjected to RT and IU1-47 or DFO, **related to Fig. 7F**.

Figure S14

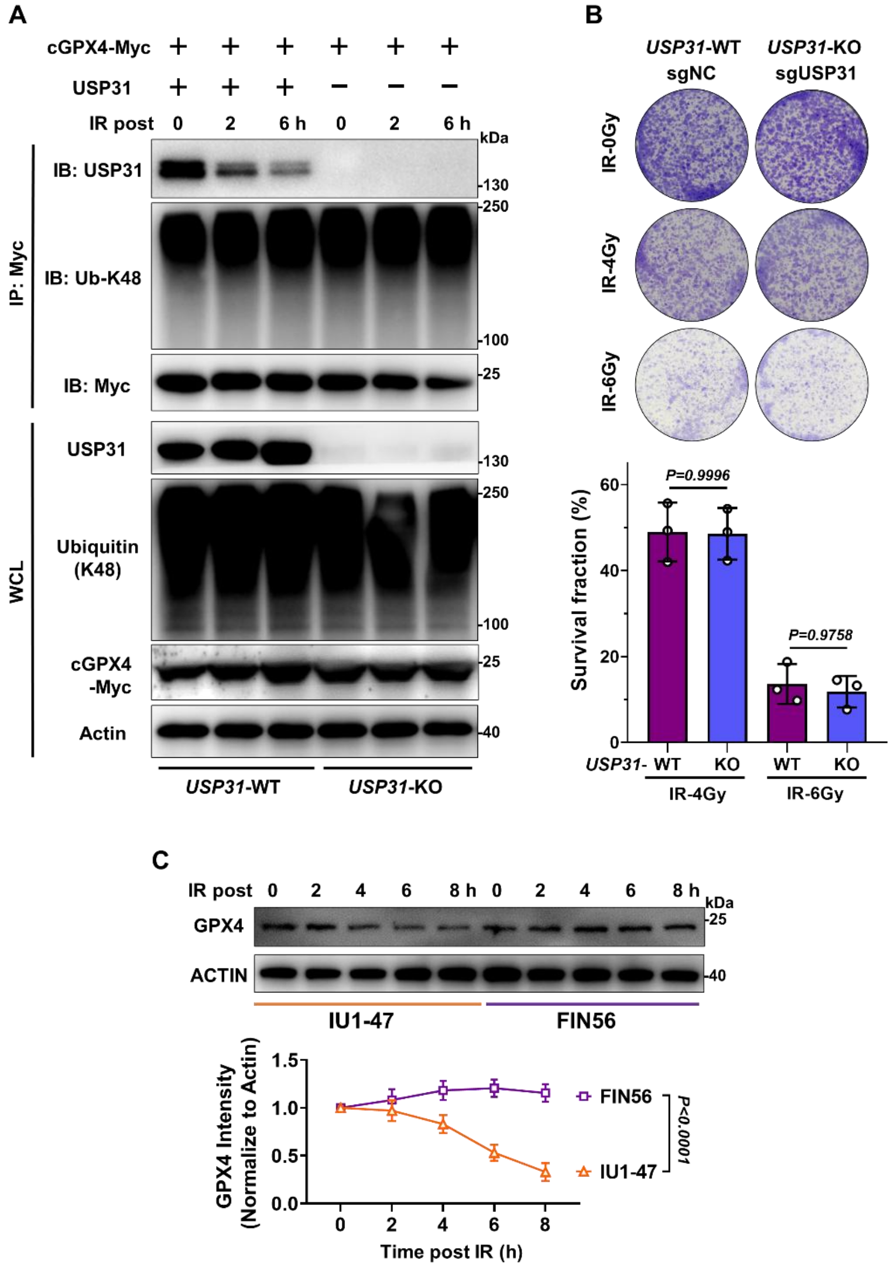

Figure S14. RT-driven process involving USP14 targeting and deubiquitinating GPX4 is relatively specific. (**A**) Co-IP and WB assays explored the interaction between USP31 and GPX4. Huh7 cells with WT USP31 or USP31-KO, overexpressing cGPX4-Myc, were irradiated with 6 Gy

IR and cellular lysates were collected at 0/2/6 hours post-IR. (**B**) Colony formation assays in Huh7 cells (including USP31-WT, KO) groups treated with a single dose of 0, 2, 4, 6, or 8 Gy IR. (**C**) GPX4 levels were detected by WB assay at the indicated time points in Huh7 cells exposed to 6 Gy IR after IU1-47 (5 μM) or FIN56 (5 μM) treatment for 2 h (DMSO as a control). Left: representative images. Right: the relative GPX4 grayscale intensity normalized to ACTIN at indicated times after IR treatment. Results are representative of three independent experiments. Data are represented as mean ± SD. (**C**): One-way ANOVA was used for comparisons of the indicated groups. (**D**): statistical significance was determined by two-way ANOVA.

Supplemental Methods

HR and NHEJ reporter assays

HCC cells were transfected with the DR-GFP (HR) or EJ5-GFP (NHEJ) reporter plasmid and pCMV-NLS-I-SceI plasmid. Meanwhile, the transfection system in absence of pCMV-NLS-I-SceI plasmid was set as the negative control. Transfection was accomplished via electroporation with a DT-130 program on a Lonza 4D machine (Cologne, Germany). 48 h after transfection, cells were harvested and collected for analysis of DNA repair efficiency (presented as GFP^+ rate of cells for *USP14*-WT or *USP14*-KO cells) on a CytoFLEX flow cytometer. For drugs, before transfection, cells were treated with drugs at the indicated concentration for 12 h and then performed above transfection as described previously.

Immunofluorescence (IF) assay

Cells on confocal dishes were fixed with 4% paraformaldehyde for 20 min, then permeabilized using 0.5% Triton X-100 in PBS and blocked with 3% goat serum for 1 h at 37°C. The cells were then incubated with primary antibodies at 4°C overnight followed by incubation with fluorogenic secondary antibodies at room temperature for 1 h. Cell nuclei were stained with DAPI for 2 min. Images were acquired using a LSM880 with Airyscan FAST Confocal microscopy (ZEISS, Oberkochen, Germany).

RT-qPCR Analysis

Total RNA was extracted using 1 mL VeZol Reagent followed by chloroform purification (200 μL added per sample, centrifuged at 13,000 rpm for 15 min at 4°C). The aqueous phase was mixed with an equal volume of isopropanol and centrifuged (13,000 rpm, 10 min, 4°C). The RNA pellet was washed with 500 μL of 75% ethanol (prepared with DEPC-treated water) and centrifuged again (13,000 rpm, 10 min, 4°C) before resuspension in 20 μL DEPC-treated water. RNA concentration was quantified using a Nanodrop 2000 spectrophotometer (Thermo Fisher Scientific), and 2 μg of total RNA was reverse transcribed to cDNA using HiScript® Q RT SuperMix with gDNA wiper (Vazyme). Quantitative PCR was performed on a Bio-Rad CFX96™ system using ChamQ SYBR® Color qPCR Master Mix with the following cycling conditions: [insert cycling parameters if available]. All reactions were run in technical triplicates with three biological replicates. Gene-specific primers were designed as follows: GPX4 (Glutathione peroxidase 4; NCBI Gene ID: 2879): F: 5'-GAGGCAAGACCGAAGTAAACTAC-3', R: 5'-CCGAACTGGTTACACGGGAA-3' (amplicon size: 120 bp). GAPDH (Glyceraldehyde-3-phosphate dehydrogenase; NCBI Gene ID: 2597, housekeeping gene): F: 5'-GGAGCGAGATCCCTCCAAAAT-3', R: 5'-GGCTGTTGTCATACTTCTCATGG-3'. Relative gene expression was calculated using the $2^{(-\Delta\Delta Ct)}$ method with Bio-Rad CFX Manager Software, and statistical analysis was performed using GraphPad Prism 8.

Supplemental Tables

Table S1. The CRISPR-sgRNAs screen results *in vitro* of Huh7 cells

sgRNA	DUB_Gene	Ctrl_count	IR _count	FC(IR/Ctrl)	Log_2(FC)	FDR	-Lg(FDR)
DUB_084	USP10	32870	21227	0.645786	-0.63087	8.52E-08	7.069469
DUB_105	USP20	26935	4427	0.164359	-2.60508	8.95E-06	5.048172
DUB_094	USP14	17418	3370	0.193478	-2.36976	9.89E-05	4.004685
DUB_095	USP14	25815	5145	0.199303	-2.32697	1.4E-05	4.85319
DUB_138	USP42	6898.6	1415.3	0.205158	-2.2852	4.66E-05	4.33153
DUB_014	BRCC3	11058	2559	0.231416	-2.11144	4.14E-05	4.38344
DUB_012	BAP1	26068.4	7309.7	0.280405	-1.83442	0.00217	2.6635

DUB_070	TRAF2	9677.9	2748.4	0.283987	-1.8161	0.087911	1.055957
DUB_128	USP34	10601	3119.2	0.294236	-1.76495	0.000528	3.277621
DUB_078	UCHL1	19977.1	6216.9	0.311201	-1.68408	0.000197	3.705798
DUB_106	USP20	11776	3907	0.331776	-1.59172	0.000476	3.322019
DUB_017	DDX58	18342	6465	0.35247	-1.50443	0.00036	3.4433
DUB_093	USP13	11375	4118	0.362022	-1.46585	0.005874	2.231081
DUB_104	USP19	3079.9	1168.5	0.379395	-1.39823	0.019555	1.708753
DUB_079	UCHL1	17429	6939	0.39813	-1.32869	0.008109	2.091054
DUB_101	USP18	21045	8826	0.419387	-1.25365	0.00163	2.787706
DUB_111	USP22	38136	16068	0.421334	-1.24696	0.033345	1.476969
DUB_042	OTUD1	18822.3	8075.5	0.429039	-1.22082	0.028254	1.54892
DUB_011	BAP1	7384.7	4025.9	0.545168	-0.87523	0.00331	2.480133
DUB_090	USP12	20712	11748	0.567207	-0.81805	0.12076	0.918077
DUB_092	USP13	21338	12282	0.575593	-0.79688	0.98897	0.004817
DUB_041	OTUD1	33873	22666	0.669147	-0.57961	0.53882	0.268556
DUB_003	ASXL1	27116	19370	0.714338	-0.48532	0.04105	1.386687
DUB_064	TNFAIP3	17122	13321	0.778005	-0.36215	0.33345	0.476969
DUB_164	VCPIP1	1579	1250.8	0.792147	-0.33616	1.11E-09	8.953934
DUB_071	TRAF2	8993	7818.4	0.869387	-0.20193	0.021971	1.65815
DUB_168	WDR48	1914.9	1685.9	0.880412	-0.18375	0.082898	1.081456
DUB_076	UBC	23563	20903	0.887111	-0.17281	0.023683	1.625563
DUB_059	RIPK1	12739	11326	0.889081	-0.16961	0.012145	1.915602
DUB_150	USP51	6285.3	5608.3	0.892288	-0.16442	0.071467	1.145894
DUB_081	UCHL3	2306.9	2065.6	0.895401	-0.15939	0.027499	1.560683
DUB_130	USP36	24232	21767	0.898275	-0.15477	0.044756	1.349149
DUB_171	ZC3H12A	11422	10262	0.898442	-0.1545	0.025207	1.598479
DUB_029	JOSD2	35092	32029	0.912715	-0.13176	0.035205	1.453396
DUB_074	UBB	5533.2	5075.1	0.917209	-0.12468	0.23185	0.634793
DUB_109	USP2	4837.3	4443.8	0.918653	-0.12241	0.36862	0.433421
DUB_061	RPS27A	30422	28007	0.920617	-0.11933	0.35206	0.453383
DUB_085	USP10	5270	4854.4	0.921139	-0.11851	0.066262	1.178735
DUB_151	USP5	3382.7	3129.4	0.925119	-0.11229	0.35206	0.453383
DUB_054	PSMD14	25807	23968	0.92874	-0.10665	0.25049	0.60121
DUB_055	PSMD14	21391	19875	0.929129	-0.10605	0.12036	0.919518
DUB_008	ATXN7L3	17544	16354	0.932171	-0.10133	0.15527	0.808912
DUB_018	DDX58	22169	20676	0.932654	-0.10059	0.21945	0.658664
DUB_004	ASXL1	16229	15156	0.933884	-0.09869	0.16685	0.777674
DUB_145	USP48	35702	33362	0.934457	-0.0978	0.25049	0.60121
DUB_056	RCE1	17167	16143	0.940351	-0.08873	0.10362	0.984556
DUB_123	USP3	16464	15570	0.9457	-0.08055	0.29604	0.52865
DUB_098	USP16	19185	18160	0.946573	-0.07921	0.33345	0.476969
DUB_147	USP49	27964	26471	0.94661	-0.07916	0.33062	0.480671

DUB_027	JOSD1	12920	12251	0.94822	-0.07671	0.26021	0.584676
DUB_142	USP46	19510	18510	0.948744	-0.07591	0.41358	0.38344
DUB_133	USP38	13521	12846	0.950078	-0.07388	0.33345	0.476969
DUB_139	USP44	37656	35799	0.950685	-0.07296	0.42545	0.371151
DUB_153	USP6	8062.5	7673	0.95169	-0.07144	0.24934	0.603208
DUB_124	USP3	24794	23617	0.952529	-0.07017	0.53882	0.268556
DUB_015	CYLD	41976	40105	0.955427	-0.06578	0.53438	0.27215
DUB_117	USP26	11697	11228	0.959904	-0.05904	0.28353	0.547401
DUB_005	ATXN3	25000	24019	0.96076	-0.05775	0.56601	0.247176
DUB_065	TNFAIP3	10049	9659.7	0.96126	-0.057	0.42631	0.370274
DUB_022	ENY2	17772	17119	0.963257	-0.05401	0.59775	0.22348
DUB_163	VCPIP1	16811	16225	0.965142	-0.05119	0.42631	0.370274
DUB_165	WDR20	28971	28077	0.969142	-0.04522	0.56733	0.246164
DUB_021	ENY2	1611	1565.1	0.971508	-0.0417	0.53882	0.268556
DUB_028	JOSD1	6003.4	5834.5	0.971866	-0.04117	0.91848	0.03693
DUB_033	MDM2	30390	29565	0.972853	-0.03971	0.65787	0.18186
DUB_131	USP37	11389	11081	0.972956	-0.03955	0.59004	0.229119
DUB_173	ZRANB1	14334	13986	0.975722	-0.03546	0.73487	0.133789
DUB_075	UBB	42893	41891	0.97664	-0.0341	0.7437	0.128602
DUB_162	USP9Y	26388	25785	0.977149	-0.03335	0.59775	0.22348
DUB_050	OTUD7A	8368.6	8209.9	0.981036	-0.02762	0.68893	0.161825
DUB_096	USP15	27381	26932	0.983602	-0.02385	0.89819	0.046632
DUB_034	MDM2	2277.2	2240.1	0.983708	-0.0237	0.81086	0.091054
DUB_010	BABAM1	16280	16042	0.985381	-0.02125	0.95545	0.019792
DUB_026	IKBKG	10013	9880.4	0.986757	-0.01923	0.89819	0.046632
DUB_066	TNIP1	11243	11099	0.987192	-0.0186	0.92924	0.031872
DUB_001	ADRM1	23452	23155	0.987336	-0.01839	0.92924	0.031872
DUB_040	OTUB2	16546	16342	0.987671	-0.0179	0.89819	0.046632
DUB_058	RIPK1	22043	21828	0.990246	-0.01414	0.91848	0.03693
DUB_148	USP49	32260	31964	0.990825	-0.0133	0.92934	0.031825
DUB_107	USP21	11123	11031	0.991729	-0.01198	0.92832	0.032302
DUB_049	OTUD6B	12667	12568	0.992184	-0.01132	0.95545	0.019792
DUB_025	IKBKG	28593	28390	0.9929	-0.01028	0.95545	0.019792
DUB_077	UBC	18230	18106	0.993198	-0.00985	0.95326	0.020789
DUB_116	USP25	3046.8	3037.6	0.99698	-0.00436	0.98897	0.004817
DUB_125	USP33	15014	14977	0.997536	-0.00356	0.98897	0.004817
DUB_032	MAP3K7	16295	16263	0.998036	-0.00284	0.98897	0.004817
DUB_057	RCE1	4016.9	4015.9	0.999751	-0.00036	0.98897	0.004817
DUB_053	OTUD7B	41793	41784	0.999785	-0.00031	0.99689	0.001353
DUB_044	OTUD3	16834	16849	1.000891	0.001285	0.99689	0.001353
DUB_167	WDR48	11961	11977	1.001338	0.001929	0.9941	0.00257
DUB_110	USP2	19671	19750	1.004016	0.005782	0.99248	0.003278

DUB_108	USP21	9209.9	9249.1	1.004256	0.006127	0.97536	0.010835
DUB_062	TANK	13908	13975	1.004817	0.006933	0.97536	0.010835
DUB_097	USP15	13628	13694	1.004843	0.00697	0.98709	0.005643
DUB_052	OTUD7B	7924.9	7971	1.005817	0.008368	0.97536	0.010835
DUB_080	UCHL3	8036.1	8089.1	1.006595	0.009484	0.97536	0.010835
DUB_091	USP12	38589	38849	1.006738	0.009688	0.97536	0.010835
DUB_023	FOXO4	25881	26080	1.007689	0.011051	0.97536	0.010835
DUB_134	USP38	26300	26505	1.007795	0.011202	0.95034	0.022121
DUB_155	USP7	14690	14810	1.008169	0.011737	0.95034	0.022121
DUB_135	USP4	9071.2	9148.2	1.008488	0.012194	0.95034	0.022121
DUB_031	MAP3K7	32084	32546	1.0144	0.020626	0.95545	0.019792
DUB_086	USP11	14006	14219	1.015208	0.021775	0.95545	0.019792
DUB_009	BABAM1	5831.6	5937.1	1.018091	0.025867	0.83049	0.080666
DUB_045	OTUD4	16034	16347	1.019521	0.027892	0.89819	0.046632
DUB_127	USP34	45216	46241	1.022669	0.032339	0.91848	0.03693
DUB_166	WDR20	37496	38364	1.023149	0.033016	0.59775	0.22348
DUB_157	USP8	20549	21025	1.023164	0.033038	0.81086	0.091054
DUB_132	USP37	15195	15577	1.02514	0.035821	0.7214	0.141824
DUB_152	USP5	2689	2757.8	1.025586	0.036448	0.62266	0.205749
DUB_119	USP28	39568	40658	1.027548	0.039205	0.72384	0.140357
DUB_016	CYLD	10870	11178	1.028335	0.04031	0.91332	0.039377
DUB_046	OTUD4	22859	23579	1.031497	0.04474	0.53882	0.268556
DUB_170	YOD1	17729	18296	1.031981	0.045417	0.72384	0.140357
DUB_100	USP17L2	27962	28873	1.03258	0.046253	0.56601	0.247176
DUB_048	OTUD6B	22928	23691	1.033278	0.047229	0.59775	0.22348
DUB_115	USP25	8506.3	8817.6	1.036596	0.051854	0.53438	0.27215
DUB_082	UCHL5	5807.4	6024.3	1.037349	0.052901	0.53882	0.268556
DUB_006	ATXN3	15920	16661	1.046545	0.065635	0.32187	0.492319
DUB_136	USP4	22452	23551	1.048949	0.068944	0.37295	0.428349
DUB_035	MYSM1	24675	25890	1.04924	0.069345	0.42631	0.370274
DUB_174	ZRANB1	11952	12563	1.051121	0.071929	0.33345	0.476969
DUB_002	ADRM1	6049.6	6360.4	1.051375	0.072278	0.32826	0.483782
DUB_160	USP9X	6093.7	6420.4	1.053613	0.075345	0.42631	0.370274
DUB_103	USP19	20946	22137	1.05686	0.079785	0.53882	0.268556
DUB_154	USP6	10746	11360	1.057138	0.080163	0.33345	0.476969
DUB_140	USP44	15831	16743	1.057608	0.080806	0.28353	0.547401
DUB_007	ATXN7L3	26017	27516	1.057616	0.080816	0.39299	0.405619
DUB_063	TANK	31218	33096	1.060158	0.084279	0.32826	0.483782
DUB_089	USP1	16711	17732	1.061097	0.085557	0.23185	0.634793
DUB_037	OTUB1	11366	12081	1.062907	0.088015	0.16486	0.782885
DUB_083	UCHL5	2128.5	2264.6	1.063942	0.089419	0.28677	0.542466
DUB_102	USP18	12532	13344	1.064794	0.090575	0.33345	0.476969

DUB_126	USP33	23124	24625	1.064911	0.090733	0.62312	0.205428
DUB_144	USP47	23598	25163	1.066319	0.092639	0.31826	0.497218
DUB_072	TRAF6	11107	11852	1.067075	0.093661	0.17982	0.745162
DUB_020	EIF3F	10351	11063	1.068786	0.095973	0.16486	0.782885
DUB_146	USP48	31283	33457	1.069495	0.096929	0.31826	0.497218
DUB_118	USP26	33926	36320	1.070565	0.098373	0.31826	0.497218
DUB_036	MYSM1	35818	38370	1.071249	0.099294	0.099168	1.003628
DUB_099	USP16	9640.5	10375	1.076189	0.105931	0.087911	1.055957
DUB_047	OTUD5	18356	19766	1.076814	0.106769	0.082898	1.081456
DUB_149	USP51	17490	18864	1.078559	0.109105	0.2775	0.556737
DUB_113	USP24	22669	24685	1.088932	0.122914	0.13952	0.855364
DUB_039	OTUB2	7788.3	8576.9	1.101254	0.139148	0.064066	1.193372
DUB_051	OTUD7A	27501	30304	1.101924	0.140024	0.12076	0.918077
DUB_143	USP47	19009	20967	1.103004	0.141438	0.15527	0.808912
DUB_060	RPS27A	3710.8	4188.5	1.128732	0.174703	0.018211	1.739666
DUB_114	USP24	14142	16057	1.135412	0.183216	0.16486	0.782885
DUB_158	USP8	8756.2	10097	1.153126	0.20555	0.008552	2.067958
DUB_073	TRAF6	4651.2	5365.8	1.153638	0.20619	0.012145	1.915602
DUB_141	USP46	37995	43930	1.156205	0.209397	0.018211	1.739666
DUB_169	YOD1	24988	29065	1.163158	0.218047	0.068429	1.16476
DUB_043	OTUD3	21859	25489	1.166064	0.221647	3.74E-06	5.426769
DUB_019	EIF3F	4342.9	5075.1	1.168597	0.224778	4.99E-05	4.301508
DUB_013	BRCC3	11427	13464	1.178262	0.23666	8.53E-05	4.069148
DUB_112	USP22	3758.2	4438.3	1.180964	0.239965	0.046844	1.329346
DUB_030	JOSD2	8751.8	10387	1.186842	0.247127	0.001096	2.960031
DUB_087	USP11	26707	31944	1.196091	0.258327	0.044731	1.349391
DUB_038	OTUB1	9489.6	11505	1.21238	0.277842	1.72E-07	6.764598
DUB_121	USP30	14247	17794	1.248965	0.320733	0.003625	2.440716
DUB_067	TNIP1	2894.9	3850.6	1.330132	0.41157	1.3E-07	6.885489
DUB_129	USP36	6815	27954	4.101834	2.036269	7.11E-07	6.148112
DUB_088	USP1	7537	49422	6.557251	2.713091	6.57E-06	5.18223
DUB_161	USP9Y	1467	15288	10.42127	3.381459	5.65E-08	7.248213

FC, Fold Change; FDR, False Discover Rate

Table S2. The CRISPR-sgRNAs screen results *in vivo* of MHCC97H xenografts

sgRNA	DUB_Gene	Ctrl_count	IR _count	FC(IR/Ctrl)	Log_2(FC)	FDR	-Lg(FDR)
DUB_057	RCE1	3035.2	1950.8	0.642717113	-0.637746696	2.33E-05	4.63286
DUB_028	JOSD1	3916.4	1849.7	0.472286856	-1.082264709	1.71E-07	6.767552
DUB_013	BRCC3	8911.9	1939.4	0.217619297	-2.200121602	0.000103	3.98799
DUB_027	UCHL3	7842.5	1711.0	0.218174595	-2.196444974	0.001206	2.918676
DUB_154	USP6	8050.5	1893.7	0.235226393	-2.08787815	0.001206	2.918676

DUB_004	ASXL1	8498.5	2518.9	0.296392697	-1.754418192	0.00107	2.970418
DUB_098	USP16	13318.0	3234.7	0.242879701	-2.041686178	0.000114	3.94397
DUB_014	BRCC3	7553.5	2226.3	0.294741184	-1.762479435	0.001206	2.918676
DUB_138	USP42	4768.4	1570.4	0.329342393	-1.602339866	0.012659	1.897598
DUB_131	USP37	7296.4	2784.3	0.381594398	-1.389888104	0.001206	2.918676
DUB_116	USP25	1926.9	825.9	0.428634949	-1.22217861	0.001333	2.87523
DUB_041	OTUD1	22600.7	10826.4	0.479029534	-1.061813487	2.19E-07	6.659825
DUB_128	USP34	6964.0	3529.2	0.506772097	-0.980591002	0.016409	1.784929
DUB_040	OTUB2	11696.4	6044.1	0.516747686	-0.952468072	0.001822	2.739538
DUB_136	USP4	15175.6	9713.4	0.640063642	-0.643712734	0.016781	1.775185
DUB_059	RIPK1	8449.3	6490.2	0.768141593	-0.380555825	0.013099	1.882757
DUB_002	ADRM1	5657.6	4519.4	0.79881054	-0.324074726	0.002511	2.600123
DUB_021	ENY2	1039.4	838.1	0.806369427	-0.310487154	0.177506	0.750787
DUB_151	USP5	2244.2	1900.0	0.846631729	-0.240193538	0.138679	0.857989
DUB_171	ZC3H12A	8425.1	7269.8	0.862873255	-0.212779433	0.014522	1.837969
DUB_111	USP22	11136.4	9613.7	0.86326759	-0.212120269	0.005954	2.225159
DUB_123	USP3	10434.6	9070.8	0.869299097	-0.202075449	0.011023	1.957683
DUB_101	USP18	14702.9	12896.6	0.87715209	-0.189101081	0.005388	2.268569
DUB_070	TRAF2	5317.3	4721.9	0.888016523	-0.171341575	0.111932	0.951044
DUB_008	ATXN7L3	10837.3	9732.1	0.898016386	-0.155186324	0.044708	1.349619
DUB_071	TRAF2	6167.6	5573.6	0.903695098	-0.146091999	0.142605	0.845867
DUB_036	MYSM1	24857.1	22576.5	0.908253431	-0.138833184	0.008752	2.057881
DUB_033	MDM2	21740.8	19750.0	0.908429764	-0.138553119	0.013693	1.863512
DUB_129	USP36	18570.8	16925.8	0.911420543	-0.133811207	0.024581	1.609394
DUB_105	USP20	16973.3	5487.2	0.323283774	-1.629126997	0.003499	2.456062
DUB_152	USP5	2029.4	1851.9	0.912569564	-0.131993556	0.400055	0.397881
DUB_169	YOD1	17458.5	16018.4	0.917510596	-0.124203276	0.044708	1.349619
DUB_016	CYLD	7537.7	6925.9	0.918843515	-0.122108913	0.177506	0.750787
DUB_068	TP53	25727.1	23651.4	0.919318216	-0.121363767	0.018085	1.742688
DUB_056	RCE1	11483.8	10619.2	0.924715138	-0.112919088	0.138679	0.857989
DUB_094	USP14	11216.6	1383.2	0.123317825	-3.019546744	1.43E-09	8.845867
DUB_102	USP18	9256.2	8582.4	0.92721306	-0.109027208	0.182366	0.739056
DUB_121	USP30	9750.0	9044.3	0.927624221	-0.108387604	0.177506	0.750787
DUB_077	UBC	12353.8	11462.7	0.9278734	-0.108000119	0.138734	0.857819
DUB_081	UCHL3	1434.5	1331.6	0.928276686	-0.10737321	0.514908	0.288271
DUB_148	USP49	22361.6	20828.7	0.931452456	-0.102445964	0.064911	1.187683
DUB_119	USP28	27927.4	26055.8	0.932981928	-0.100078959	0.044708	1.349619
DUB_029	JOSD2	22947.3	21525.0	0.938021859	-0.092306553	0.095249	1.02114
DUB_130	USP36	14286.2	13416.2	0.939101516	-0.090646975	0.177506	0.750787
DUB_153	USP6	5362.4	5052.0	0.942118871	-0.086018993	0.395187	0.403198
DUB_061	RPS27A	22207.3	20990.0	0.945180976	-0.081337502	0.142605	0.845867
DUB_142	USP46	13401.4	12667.7	0.945251656	-0.081229623	0.241405	0.617254

DUB_054	PSMD14	16840.1	15956.9	0.947550992	-0.077724511	0.213747	0.6701
DUB_127	USP34	31971.3	30307.7	0.94796336	-0.077096797	0.103887	0.98344
DUB_069	TP53	14948.2	14193.5	0.949510213	-0.074744577	0.256694	0.590584
DUB_032	MAP3K7	10862.2	10384.8	0.956044744	-0.064849955	0.37883	0.421555
DUB_005	ATXN3	17458.5	16717.0	0.957528441	-0.062612756	0.30479	0.515999
DUB_053	OTUD7B	26861.9	25731.0	0.957898399	-0.062055452	0.220492	0.656607
DUB_042	OTUD1	6131.4	5891.5	0.960874481	-0.057580111	0.506278	0.295611
DUB_104	USP19	2116.8	2034.5	0.961108249	-0.057229164	0.626645	0.202978
DUB_162	USP9Y	17795.0	17104.1	0.961176522	-0.057126686	0.339364	0.469334
DUB_145	USP48	22754.9	21928.5	0.96368304	-0.05336938	0.324636	0.488603
DUB_149	USP51	13035.3	12617.8	0.967973231	-0.046960944	0.469561	0.328308
DUB_076	UBC	15111.0	14631.2	0.968249059	-0.0465499	0.456344	0.340708
DUB_114	USP24	10650.4	10312.3	0.968260933	-0.046532208	0.502781	0.298621
DUB_146	USP48	22043.0	21377.8	0.969824388	-0.044204562	0.406262	0.391194
DUB_015	CYLD	30005.5	29129.2	0.970797425	-0.042757813	0.366656	0.435741
DUB_134	USP38	18668.1	18192.2	0.974507677	-0.037254544	0.493255	0.306929
DUB_072	TRAF6	7006.4	6838.1	0.975976877	-0.035081127	0.621855	0.206311
DUB_163	VCPIP1	11199.5	10961.9	0.978788511	-0.030930928	0.604105	0.218888
DUB_055	PSMD14	13867.9	13628.0	0.982701488	-0.025174854	0.626599	0.203011
DUB_052	OTUD7B	5448.2	5355.4	0.982973309	-0.024775852	0.678838	0.168234
DUB_035	MYSM1	15048.7	14810.3	0.98416231	-0.023031827	0.632923	0.198649
DUB_091	USP12	26365.8	25958.4	0.984549939	-0.022463709	0.58806	0.230578
DUB_003	ASXL1	11891.9	11711.2	0.984804821	-0.022090271	0.654716	0.183947
DUB_001	ADRM1	15795.6	15574.4	0.985996055	-0.02034622	0.652138	0.18566
DUB_155	USP7	11057.7	10911.3	0.986757766	-0.019232127	0.678838	0.168234
DUB_103	USP19	15315.8	15116.4	0.986981286	-0.018905365	0.660791	0.179936
DUB_132	USP37	10913.6	10775.0	0.987296603	-0.018444532	0.678838	0.168234
DUB_133	USP38	11261.0	11120.0	0.987480979	-0.018175135	0.678838	0.168234
DUB_075	UBB	28452.4	28151.7	0.989433342	-0.015325578	0.654155	0.184319
DUB_046	OTUD4	17240.4	17085.4	0.991009713	-0.013028897	0.694283	0.158463
DUB_045	OTUD4	12686.3	12592.9	0.992632613	-0.01066824	0.729457	0.137
DUB_023	FOXO4	16938.2	16854.1	0.995033798	-0.007182565	0.739107	0.131292
DUB_170	YOD1	12716.7	12664.5	0.995896368	-0.00593247	0.754031	0.122611
DUB_093	USP13	7074.5	7059.0	0.997820079	-0.003148393	0.763572	0.11715
DUB_089	USP1	11685.5	11660.6	0.997867093	-0.00308042	0.763572	0.11715
DUB_126	USP33	13914.6	13892.0	0.998376714	-0.002343809	0.763572	0.11715
DUB_141	USP46	26999.8	26974.8	0.999076879	-0.001332398	0.763572	0.11715
DUB_165	WDR20	19126.9	19483.9	1.018664833	0.026679445	0.778877	0.108531
DUB_044	OTUD3	10721.2	10736.0	1.001380312	0.001989997	0.763572	0.11715
DUB_166	WDR20	25244.2	25305.7	1.002437444	0.00351221	0.754031	0.122611
DUB_139	USP44	24659.2	24721.6	1.002526848	0.003640873	0.754031	0.122611
DUB_006	ATXN3	11042.9	11085.0	1.003808718	0.005484381	0.754031	0.122611

DUB_147	USP49	18191.4	18292.7	1.005566022	0.008007807	0.732425	0.135237
DUB_150	USP51	3985.4	4008.6	1.005843496	0.008405847	0.754031	0.122611
DUB_095	USP14	10167.5	2243.0	0.220606711	-2.180451418	7.32E-05	4.135237
DUB_037	OTUB1	8198.5	8261.5	1.007695231	0.011059374	0.732425	0.135237
DUB_058	RIPK1	14142.1	14254.2	1.007930826	0.01139663	0.722533	0.141142
DUB_092	USP13	13990.2	14128.0	1.009854137	0.014146925	0.694283	0.158463
DUB_050	OTUD7A	6092.5	6154.4	1.010150597	0.014570391	0.729457	0.137
DUB_157	USP8	13196.5	13352.3	1.011804285	0.016930254	0.678838	0.168234
DUB_124	USP3	17884.6	18226.5	1.019118544	0.027321875	0.576065	0.239528
DUB_107	USP21	7987.4	8143.9	1.019600195	0.028003554	0.647948	0.18846
DUB_096	USP15	19845.8	20239.9	1.019858713	0.028369301	0.547543	0.261582
DUB_088	USP1	33473.0	34175.6	1.020988459	0.029966558	0.470317	0.327609
DUB_010	BABAM1	11057.7	11363.8	1.027681905	0.03939378	0.529971	0.275748
DUB_062	TANK	9700.1	9978.2	1.028665489	0.04077391	0.534839	0.271777
DUB_125	USP33	10385.5	10696.3	1.029923504	0.042537187	0.514908	0.288271
DUB_161	USP9Y	11063.9	11445.6	1.034494896	0.048926527	0.469561	0.328308
DUB_090	USP12	8259.2	8544.3	1.034515277	0.04895495	0.506278	0.295611
DUB_026	IKBKG	6828.8	7064.6	1.034525235	0.048968837	0.529021	0.276527
DUB_031	MAP3K7	20647.2	21422.2	1.037534422	0.053159202	0.324636	0.488603
DUB_174	ZRANB1	7599.5	7888.5	1.038023983	0.053839776	0.493255	0.306929
DUB_118	USP26	22895.1	23776.8	1.038509951	0.05451504	0.300312	0.522428
DUB_097	USP15	8740.6	9092.6	1.040278025	0.056969155	0.456344	0.340708
DUB_066	TNIP1	7226.5	7533.4	1.042465591	0.059999765	0.46534	0.33223
DUB_106	USP20	8417.3	8785.7	1.043767928	0.061800978	0.426669	0.369909
DUB_038	OTUB1	6704.1	7002.8	1.044554686	0.062888024	0.46129	0.336026
DUB_109	USP2	3379.9	3531.6	1.04486692	0.063319205	0.536662	0.270299
DUB_083	UCHL5	1654.6	1733.9	1.047921668	0.06753088	0.607968	0.216119
DUB_110	USP2	13750.3	14451.3	1.050979948	0.071735144	0.270683	0.567539
DUB_073	TRAF6	3121.8	3288.6	1.053416831	0.075076415	0.506278	0.295611
DUB_007	ATXN7L3	18558.3	19549.8	1.053426785	0.075090047	0.18274	0.738166
DUB_039	OTUB2	5338.6	5625.1	1.053675119	0.075430107	0.426669	0.369909
DUB_049	OTUD6B	9460.2	9997.7	1.056808826	0.07971442	0.300312	0.522428
DUB_022	ENY2	13043.1	13793.1	1.05750627	0.080666217	0.221435	0.654754
DUB_099	USP16	6247.2	6621.2	1.059856872	0.08386945	0.352146	0.453277
DUB_009	BABAM1	4658.0	4958.6	1.064544178	0.090235822	0.374391	0.426675
DUB_115	USP25	5906.0	6314.4	1.069144236	0.096456497	0.300312	0.522428
DUB_082	UCHL5	3916.4	4189.2	1.069645805	0.097133151	0.374391	0.426675
DUB_144	USP47	16213.9	17383.0	1.07210453	0.100445575	0.095249	1.02114
DUB_173	ZRANB1	9168.2	9832.5	1.072466231	0.100932221	0.182935	0.737704
DUB_143	USP47	13802.5	14889.8	1.078776593	0.109396123	0.09021	1.044748
DUB_117	USP26	7922.7	8549.0	1.079040503	0.109749019	0.177506	0.750787
DUB_011	BAP1	3155.4	3425.2	1.085505529	0.118367075	0.324636	0.488603

DUB_019	EIF3F	3513.4	3819.2	1.087057728	0.120428557	0.300312	0.522428
DUB_113	USP24	15489.5	16842.4	1.087343491	0.120807758	0.044708	1.349619
DUB_108	USP21	6478.8	7058.4	1.089467547	0.123623222	0.166454	0.778706
DUB_064	TNFAIP3	13052.4	14236.3	1.090702948	0.125258238	0.052211	1.282236
DUB_100	USP17L2	19789.7	21620.1	1.092490554	0.127620806	0.016409	1.784929
DUB_025	IKBKG	19336.4	21180.8	1.095383872	0.131436544	0.014522	1.837969
DUB_085	USP10	3380.8	3711.4	1.09779754	0.134612011	0.253595	0.59586
DUB_020	EIF3F	7148.7	7873.7	1.101414221	0.139357141	0.101963	0.991558
DUB_047	OTUD5	13244.0	14620.3	1.103916725	0.142631346	0.023042	1.637474
DUB_167	WDR48	7642.8	8478.1	1.109288058	0.149634051	0.065667	1.182652
DUB_017	DDX58	12381.0	13740.9	1.109838953	0.150350345	0.019593	1.707907
DUB_048	OTUD6B	16053.4	17844.1	1.11154238	0.152562956	0.008752	2.057881
DUB_043	OTUD3	17161.8	19092.6	1.112507942	0.153815636	0.006137	2.21206
DUB_051	OTUD7A	18420.4	20553.0	1.11577167	0.158041826	0.003752	2.425728
DUB_079	UCHL1	11418.3	12746.3	1.116302865	0.158728499	0.017104	1.766899
DUB_063	TANK	21745.5	24282.3	1.116658906	0.159188568	0.001459	2.835808
DUB_065	TNFAIP3	7488.1	8368.3	1.117536925	0.160322501	0.048533	1.313966
DUB_074	UBB	3437.3	3852.2	1.120683404	0.164378771	0.156741	0.804817
DUB_018	DDX58	15544.0	17426.6	1.121110387	0.164928336	0.004944	2.305935
DUB_140	USP44	10960.4	12349.9	1.126776578	0.17220148	0.011663	1.933193
DUB_164	VCPIP1	1158.7	1312.4	1.132620824	0.17966496	0.324636	0.488603
DUB_030	JOSD2	6858.8	7964.8	1.161253691	0.215683182	0.008752	2.057881
DUB_078	UCHL1	4728.7	5503.0	1.163740282	0.21876912	0.022973	1.638783
DUB_084	USP10	23444.2	27338.6	1.166112957	0.221707544	2.66E-06	5.574505
DUB_080	UCHL3	7040.0	8255.3	1.172624382	0.229740959	0.004728	2.32535
DUB_067	TNIP1	2260.1	2657.8	1.175965813	0.23384612	0.087406	1.058461
DUB_112	USP22	2729.4	3218.6	1.179236937	0.23785362	0.053197	1.274117
DUB_168	WDR48	1532.6	1810.1	1.181074351	0.240099788	0.138679	0.857989
DUB_012	BAP1	5213.0	6833.2	1.310802331	0.390450143	2.06E-06	5.686927
DUB_135	USP4	7874.4	10557.7	1.340751731	0.423042116	1.11E-10	9.956243
DUB_034	MDM2	3037.0	4117.8	1.35586787	0.439216594	2.37E-05	4.625658
DUB_087	USP11	10384.8	20835.0	2.006300158	1.00453746	0.000178	3.748715
DUB_158	USP8	6423.9	14129.6	2.199522291	1.137190222	0.000191	3.717896
DUB_160	USP9X	5628.5	16480.3	2.928013949	1.549922427	3.06E-09	8.514139
DUB_060	RPS27A	3801.5	13315.8	3.502724963	1.808477711	4.62E-10	9.335477
DUB_086	USP11	1975.2	11183.1	5.661671924	2.501228152	0.005934	2.226656

FC, Fold Change; FDR, False Discover Rate

Materials Source Table

Materials	Source	Identifier
**Antibodies**		

Rabbit anti-USP14	Cell Signaling Technology	Cat#11931; RRID: AB_2721157
Rabbit anti-GAPDH	Cell Signaling Technology	Cat#2118; RRID: AB_561053
Rabbit anti-Ki-67	Cell Signaling Technology	Cat#12202; RRID: AB_2620142
Rabbit anti-4-Hydroxynonenal (4-HNE)	Abcam	Cat#ab46545; RRID: AB_722490
Rabbit anti-xCT/SLC7A11	Cell Signaling Technology	Cat#12691; RRID: AB_2687474
Rabbit anti-GPX4-1	Cell Signaling Technology	Cat#52455; RRID: AB_2924984
Rabbit anti-GPX4-2	Abcam	Cat#ab125066; RRID: AB_10973901
Rabbit anti-FACL4 (ASCL4)	Abcam	Cat#ab155282; RRID: AB_2714020
Rabbit anti-FTH1	Cell Signaling Technology	Cat#4393; RRID: AB_11217441
Rabbit anti-LPCAT3	Abcam	Cat#ab232958; RRID: AB_2927484
Rabbit anti-NCOA4	Cell Signaling Technology	Cat#66849
Rabbit anti-KEAP1	Cell Signaling Technology	Cat#8047; RRID: AB_10860776
Rabbit anti-NRF2	Cell Signaling Technology	Cat#12721; RRID: AB_2715528
Rabbit anti-AIFM2/FSP1	Cell Signaling Technology	Cat#24972
Rabbit anti-GCH1	Abcam	Cat#ab236387
Mouse anti-β-Actin	Cell Signaling Technology	Cat#3700; RRID: AB_2242334
Mouse anti-HA tag	Abcam	Cat#ab18181; RRID: AB_444303
Mouse anti-Myc-Tag	Cell Signaling Technology	Cat#2276; RRID: AB_331783
Mouse anti-DYKDDDDK (Flag) Tag	Cell Signaling Technology	Cat#8146; RRID: AB_10950495
Mouse anti-TRIM14	Sigma-Aldrich	Cat#SAB1410027
Mouse anti-Ubiquitin	Santa Cruz	Cat#sc-8017; RRID: AB_628423
Mouse anti-Flag Affinity Gel	Beyotime Biotechnology	Cat#P2271
Rabbit anti-K48-linkage Specific Polyubiquitin	Cell Signaling Technology	Cat#8081; RRID: AB_10859893
Anti-rabbit IgG, HRP-linked Antibody	Cell Signaling Technology	Cat#7074; RRID: AB_2099233
Anti-mouse IgG, HRP-linked Antibody	Cell Signaling Technology	Cat#7076; RRID: AB_330924
**Chemicals, peptides and Reagents**		
DMSO	Sigma-Aldrich	Cat#D4540
N-Ethylmaleimide	Topscience	Cat#T3088
IU1-47	Topscience	Cat#T15604
NS3694	Topscience	Cat#T22119
Necroptosis-IN-3	Topscience	Cat#T64349
Liproxstatin-1	Topscience	Cat#T2376
Ac-YVAD-CMK	Topscience	Cat#T36347

Erastin	Topscience	Cat#T1765
ML210	Topscience	Cat#T8375
2,4-Diamino-6-hydroxypyrimidine	Topscience	Cat#T7461
iFSP1	Topscience	Cat#T11631
FIN56	Topscience	Cat#T4066
TAK243	Topscience	Cat#T16974
RSL3	Topscience	Cat#T3646
FINO2	Topscience	Cat#T60084
Auranofin	Topscience	Cat#T1303
Buthionine Sulfonimine (BSO)	Topscience	Cat#T60084
DMEM, high glucose, pyruvate	Corning	Cat#10-013-CV
DAPI	ThermoFisher	Cat#D3571
Tris-HCl	Amresco	Cat#VWRV0497
EDTA	Sigma-Aldrich	Cat#60-00-4
$MgCl_2$	Sigma-Aldrich	Cat#M4880
Dithiothreitol (DTT)	Sigma-Aldrich	Cat#3483-12-3
ATP	Sigma-Aldrich	Cat#34369-07-8
Sucrose	Sigma-Aldrich	Cat#V900116
Ovalbumin	Sigma-Aldrich	Cat#S7951
Triton X-100	Sigma-Aldrich	Cat#X100
Antibody Diluent	ThermoFisher	Cat#003218
Puromycin	Gibco	Cat#A1113803
Goat anti-Rabbit IgG (H+L) Cross-Adsorbed Secondary Antibody, Alexa Fluor™ 488	ThermoFisher	Cat#A-11008
Penicillin-Streptomycin	New Cell & Molecular Biotech	Cat#C100C5
Trypsin-EDTA	New Cell & Molecular Biotech	Cat#C100C1
ubiquitin-7-amido-4-methyl coumarin (Ub-AMC)	Bostom Biochem	Cat#S-280
BODIDY™ 581/590 C11	ThermoFisher	Cat#D3861
Lysis buffer	Cell Signaling Technology	Cat#9803
Protease and Phosphatase inhibitors cocktail	ThermoFisher	Cat#78442
PVDF membrane	Sigma-Aldrich	Cat#03010040001
Kolliphor HS-15	Sigma-Aldrich	Cat#42966
Enhanced chemiluminescence Reagent	ThermoFisher	Cat#WP20005
**Critical Commercial Assays**		
Bicinchoninic acid protein assay kit	Epizymebio	Cat#PG110/112/113
CCK-8 assay kit	MedChemExpress	Cat#HY-K0301
BCA protein assay kit	KeyGen Biotech	Cat#KGP903
DT-130 program kit	Cologne	Cat#V4XP-2032
Lipofectamine 3000 transfection reagent	ThermoFisher	Cat#L3000008
GSH/GSSG Ratio Detection Assay Kit	Abcam	Cat#ab205811
Deproteinizing Sample Kit	Abcam	Cat#ab204708
Glutathione peroxidase assay kit	Abcam	Cat#ab102530
cDNA Reverse Transcription Kit	Applied Biosystems	Cat#43-688-14
TaqMan™ Universal PCR Master Mix	Applied Biosystems	Cat#4305719
QuantiTect SYBR Green PCR Kit	Qiagen	Cat#204143
DNA Extraction Kit	Tiangen	Cat#DP304-02

Mouse IFN-beta Quantikine ELISA Kit	Bio-Techne China Co. Ltd.	Cat#DY466
Mouse IFN-gamma Quantikine ELISA Kit	Bio-Techne China Co. Ltd.	Cat#SMIF00
Mouse CXCL10/IP-10/CRG-2 DuoSet ELISA	Bio-Techne China Co. Ltd.	Cat#DY466
Mouse ISG15 ELISA	Cusabio	Cat#CSB-EL011843MO
**Knockout or control Cells**		
Huh-7 cells with CRISPR-DUB sgRNA Library	This paper	N/A
Huh-7 cells with CRISPR-sgRNA-Negative Control expression	This paper	N/A
Huh-7 cells with CRISPR knockout of USP14 (sgRNA-1)	This paper	N/A
Huh-7 cells with CRISPR knockout of USP14 (sgRNA-2)	This paper	N/A
MHCC97H cells with CRISPR-DUB sgRNA Library	This paper	N/A
MHCC97H cells with CRISPR-sgRNA-Negative Control expression	This paper	N/A
MHCC97H cells with CRISPR knockout of USP14 (sgRNA-1)	This paper	N/A
MHCC97H cells with CRISPR knockout of USP14 (sgRNA-2)	This paper	N/A
MHCC97H cells with CRISPR knockout of USP14 (sgRNA-3)	This paper	N/A
SUN-449 cells with CRISPR-sgRNA-Negative Control expression	This paper	N/A
SUN-449 cells with CRISPR knockout of USP14 (sgRNA-1)	This paper	N/A
SUN-449 cells with CRISPR knockout of USP14 (sgRNA-2)	This paper	N/A
SUN-449 cells with CRISPR knockout of USP14 (sgRNA-3)	This paper	N/A
Huh-7 cells with CRISPR knockout of GPX4	This paper	N/A
Huh-7 cells with CRISPR knockout of TRIM14	This paper	N/A
**Overexpressed or control cells**		
Huh-7 cells with CRISPR knockout of USP14 (sgRNA-1) stably expressing vector vehicle control	This paper	N/A
Huh-7 cells with CRISPR knockout of USP14 (sgRNA-1) stably expressing Flag-USP14	This paper	N/A
Huh-7 cells with CRISPR knockout of USP14 (sgRNA-1) stably expressing Flag-USP14 mutation (C114A)	This paper	N/A
MHCC97H cells with CRISPR knockout of USP14 (sgRNA-2) stably expressing vector vehicle control	This paper	N/A
MHCC97H cells with CRISPR knockout of	This paper	N/A

USP14 (sgRNA-2) stably expressing Flag-USP14		
MHCC97H cells with CRISPR knockout of USP14 (sgRNA-2) stably expressing Flag-USP14 mutation (C114A)	This paper	N/A
Huh-7 cells stably expressing vector vehicle control	This paper	N/A
Huh-7 cells stably expressing Flag-USP14	This paper	N/A
Huh-7 cells stably expressing cytoplasmic GPX4-Myc (cGPX4-Myc)	This paper	N/A
Huh-7 cells stably expressing cGPX4-Myc with HA-Ubiquitin (WT)	This paper	N/A
Huh-7 cells stably expressing cGPX4-Myc with HA-Ubiquitin (non-K, all K mutated to R)	This paper	N/A
Huh-7 cells stably expressing cGPX4-Myc with HA-Ubiquitin (K6-only, other K mutated to R)	This paper	N/A
Huh-7 cells stably expressing cGPX4-Myc with HA-Ubiquitin (K11-only, other K mutated to R)	This paper	N/A
Huh-7 cells stably expressing cGPX4-Myc with HA-Ubiquitin (K27-only, other K mutated to R)	This paper	N/A
Huh-7 cells stably expressing cGPX4-Myc with HA-Ubiquitin (K29-only, other K mutated to R)	This paper	N/A
Huh-7 cells stably expressing cGPX4-Myc with HA-Ubiquitin (K33-only, other K mutated to R)	This paper	N/A
Huh-7 cells stably expressing cGPX4-Myc with HA-Ubiquitin (K48-only, other K mutated to R)	This paper	N/A
Huh-7 cells stably expressing cGPX4-Myc with HA-Ubiquitin (K63-only, other K mutated to R)	This paper	N/A
Huh-7 cells stably expressing cGPX4-Myc with HA-Ubiquitin (K6R, only K6 mutated to R)	This paper	N/A
Huh-7 cells stably expressing cGPX4-Myc with HA-Ubiquitin (K11R, only K11 mutated to R)	This paper	N/A
Huh-7 cells stably expressing cGPX4-Myc with HA-Ubiquitin (K27R, only K27 mutated to R)	This paper	N/A
Huh-7 cells stably expressing cGPX4-Myc with HA-Ubiquitin (K29R, only K29 mutated to R)	This paper	N/A
Huh-7 cells stably expressing cGPX4-Myc with HA-Ubiquitin (K33R, only K33 mutated	This paper	N/A

to R)		
Huh-7 cells stably expressing cGPX4-Myc with HA-Ubiquitin (K48R, only K48 mutated to R)	This paper	N/A
Huh-7 cells stably expressing cGPX4-Myc with HA-Ubiquitin (K63R, only K63 mutated to R)	This paper	N/A
Huh-7 cells stably expressing mutation cGPX4-Myc (K20R)	This paper	N/A
Huh-7 cells stably expressing mutation cGPX4-Myc (K31R)	This paper	N/A
Huh-7 cells stably expressing mutation cGPX4-Myc (K48R)	This paper	N/A
Huh-7 cells stably expressing mutation cGPX4-Myc (K80R)	This paper	N/A
Huh-7 cells stably expressing mutation cGPX4-Myc (K90R)	This paper	N/A
Huh-7 cells stably expressing mutation cGPX4-Myc (K99R)	This paper	N/A
Huh-7 cells stably expressing mutation cGPX4-Myc (K105R)	This paper	N/A
Huh-7 cells stably expressing mutation cGPX4-Myc (K118R)	This paper	N/A
Huh-7 cells stably expressing mutation cGPX4-Myc (K121R)	This paper	N/A
Huh-7 cells stably expressing mutation cGPX4-Myc (K125R)	This paper	N/A
Huh-7 cells stably expressing mutation cGPX4-Myc (K127R)	This paper	N/A
Huh-7 cells stably expressing mutation cGPX4-Myc (K135R)	This paper	N/A
Huh-7 cells stably expressing mutation cGPX4-Myc (K140R)	This paper	N/A
Huh-7 cells stably expressing mutation cGPX4-Myc (K145R)	This paper	N/A
Huh-7 cells stably expressing mutation cGPX4-Myc (K151R)	This paper	N/A
Huh-7 cells stably expressing mutation cGPX4-Myc (K164R)	This paper	N/A
Huh-7 cells stably expressing mutation cGPX4-Myc (K48R and K118R)	This paper	N/A
Huh-7 cells stably expressing cGPX4-Myc with vector vehicle control	This paper	N/A
Huh-7 cells stably expressing cGPX4-Myc with Flag-USP14 and vector vehicle control	This paper	N/A
Huh-7 cells stably expressing cGPX4-Myc with HA-TRIM14 and vector vehicle control	This paper	N/A
Huh-7 cells stably expressing cGPX4-Myc with HA-TRIM14 and Flag-USP14	This paper	N/A
Huh-7 cells stably expressing cGPX4-Myc	This paper	N/A

with Flag-USP14		
Huh-7 cells stably expressing mutation cGPX4-Myc (K48R and K118R) with HA-TRIM14 and Flag-USP14	This paper	N/A
**Oligonucleotides**		
DUB sgRNA Library	Tables S1-2	N/A
sgNC (Scrambled) 5'-CGAGCTTGACTACTTGGCAA-3'	This paper	N/A
USP14 sgRNA-1 5'-ACCGGAGTAGAGCGGCATGG-3'	This paper	N/A
USP14 sgRNA-2 5'-CAAGCAGTACTAACACACCA-3'	This paper	N/A
USP14 sgRNA-3 5'-CAGTACTAACACACCAGGGA-3'	This paper	N/A
GPX4 sgRNA 5'-CGTGTGCATCGTCACCAACG-3'	This paper	N/A
GPX4 siRNA-1 5'-GCAAGACCGAAGUAAACUATT-3'	This paper	N/A
GPX4 siRNA-2 5'-GGAUGAAGAUCCAACCCAATT-3'	This paper	N/A
GPX4 siRNA-3 5'-CCAAGUUCCUCAUCGACAATT-3'	This paper	N/A
GPX4 siRNA-4 5'-CCCUGGUGAUAGAGAAGGATT-3'	This paper	N/A
TRIM14 sgRNA 5'-GCCTTCTACGACGTGACGGG-3'	This paper	N/A
**Recombinant DNA**		
pCDH-EF1-MCS-T2A-Puro vector	Addgene	Cat#72263
pLVX-IRSE vector	Takara	Cat#631849
Lenti-CRISPR v2 vector	Addgene	Cat#52961
psPAX2	Laboratory of Dr. Ziyang Wang	N/A
pMD2.G	Laboratory of Dr. Ziyang Wang	N/A
pLVX-SECISBP2L-T2A-puro	This paper	N/A
DR-GFP (HR) reporter	Addgene	Cat#26475
EJ5-GFP (NHEJ) reporter	Addgene	Cat#44026
pCMV-NLS-I-SceI	Addgene	Cat#26477
**Software and Algorithms**		
GraphPad Prism	GraphPad 8.0 Software	GraphPad
SPSS	SPSS 20.0 Software	SPSS
FlowJo	FlowJo V10	FlowJo
ImageJ	National Institutes of Health	ImageJ
QuPath	QuPath-0.3.0 Software	QuPath
