# Supplementary material for: Targeting the TRIM14/USP14 axis enhances radiotherapy efficacy by inducing GPX4 degradation and disrupting ferroptotic defense in HCC
Source: Cell Death Dis. 2025 Jul 1;16(1):481. doi: 10.1038/s41419-025-07807-6 (PMC12219831; doi:10.1038/s41419-025-07807-6)

Figure 4D

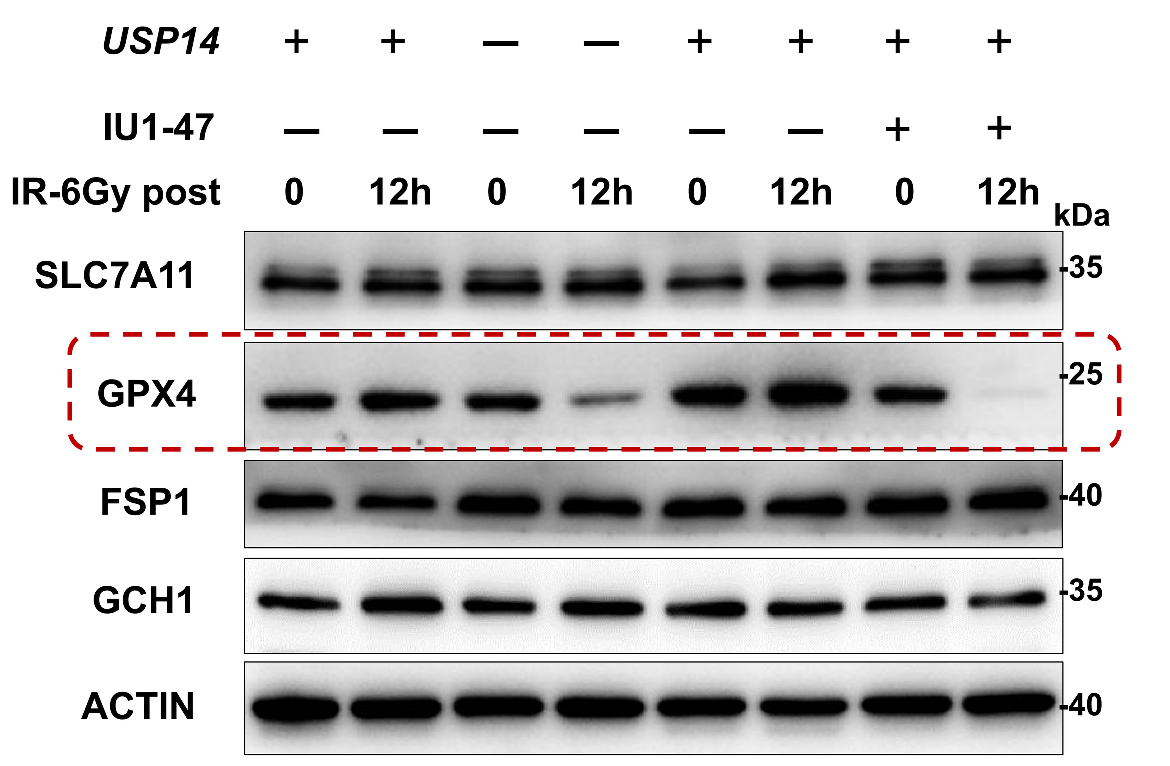

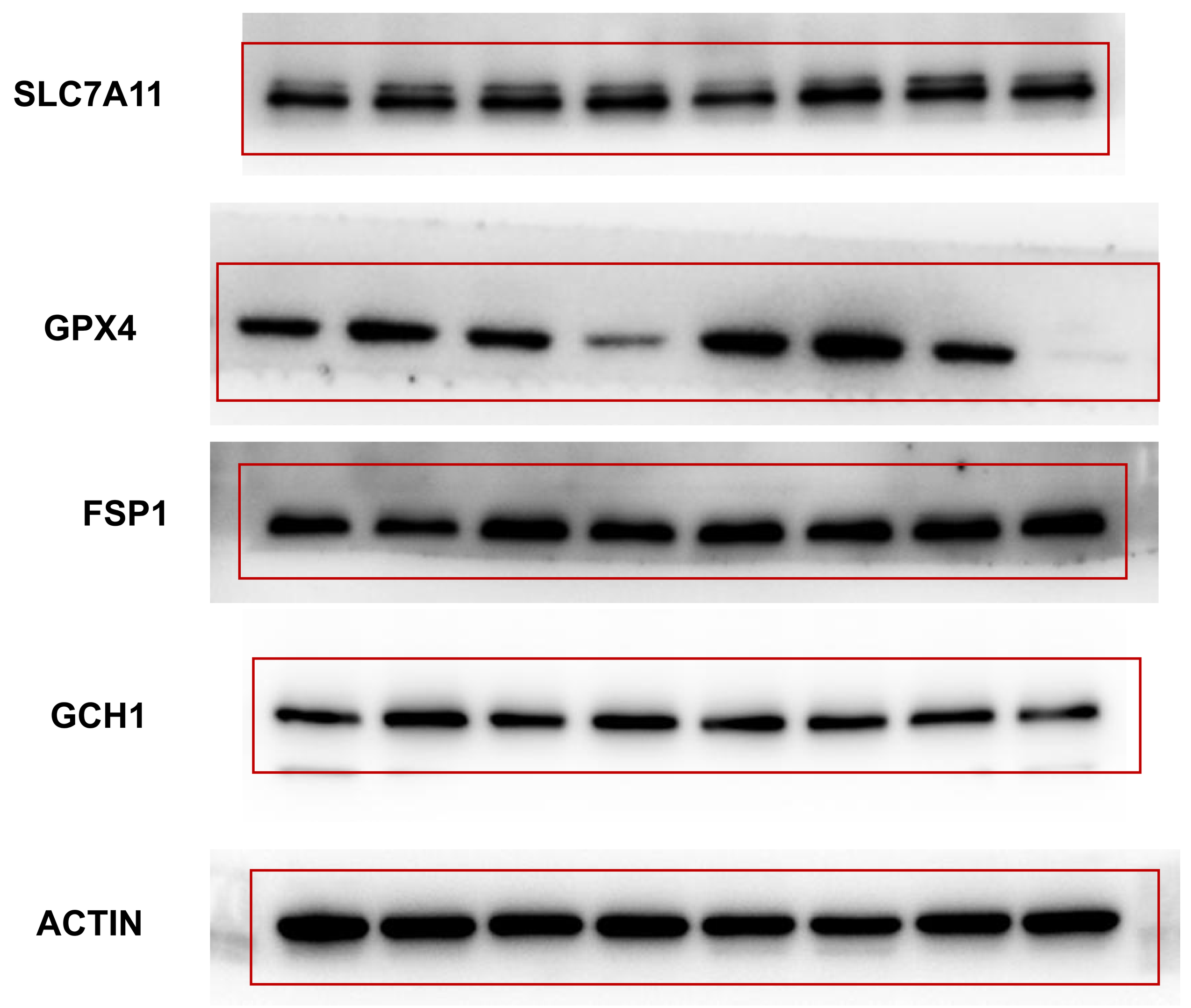

Figure 4E

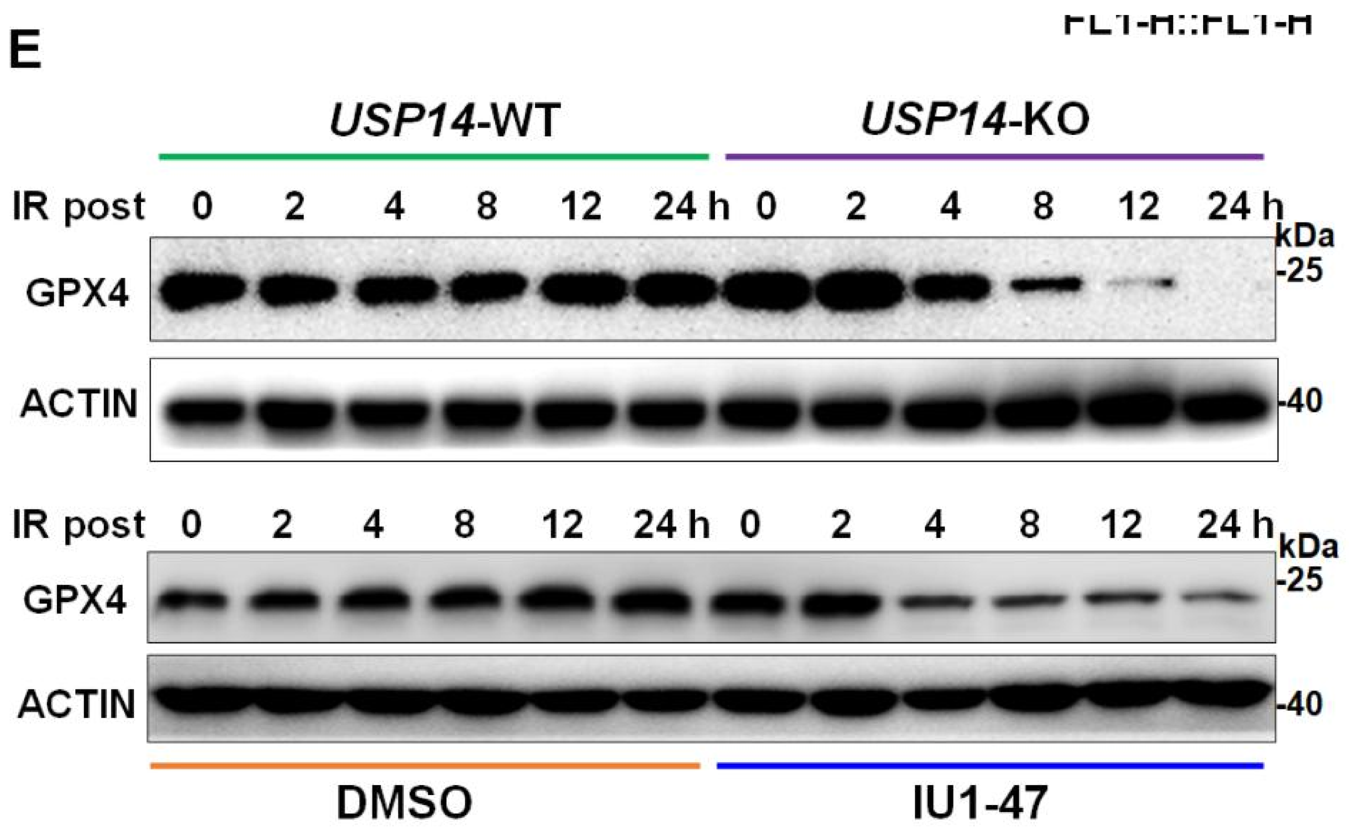

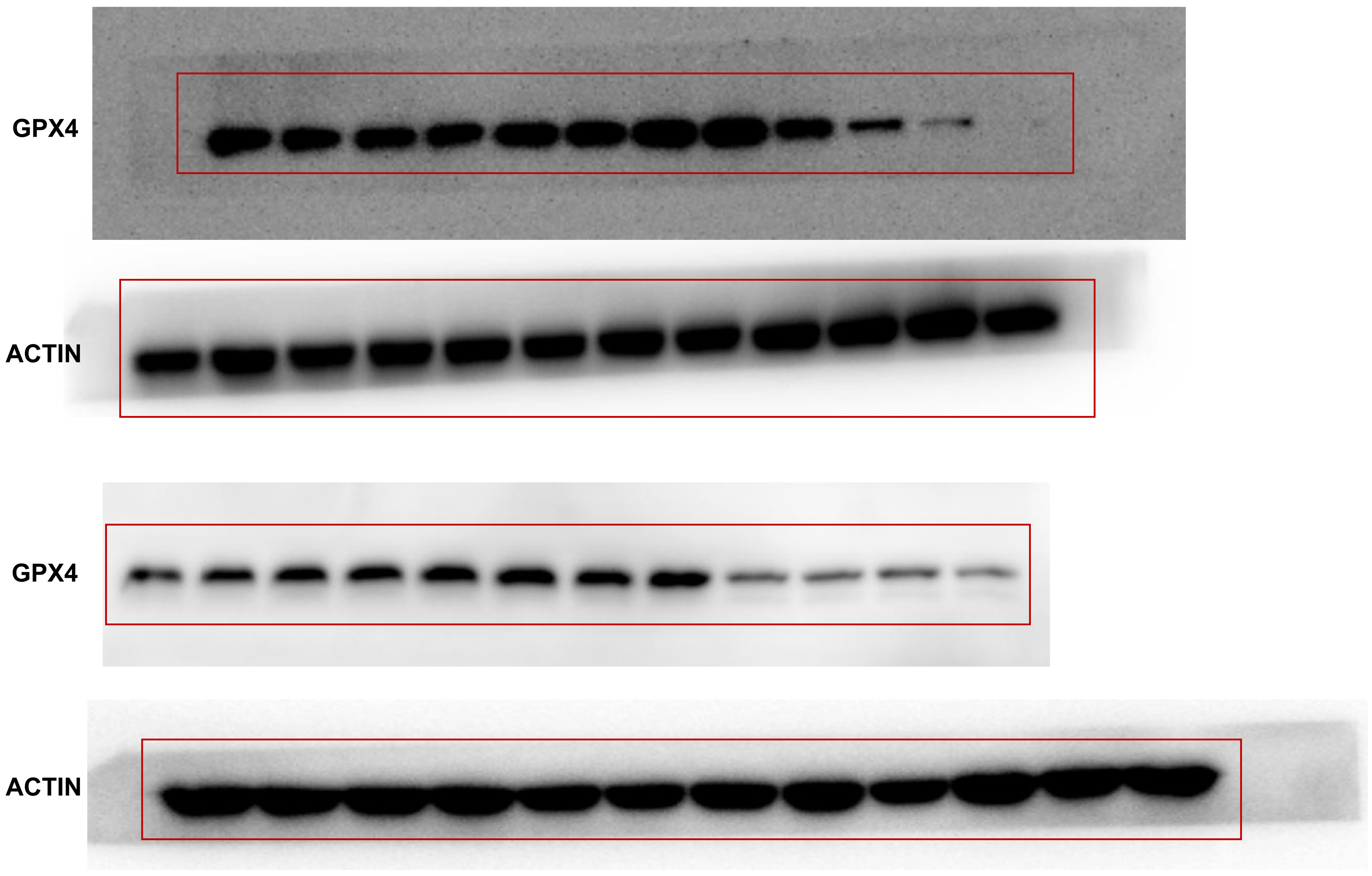

Figure 5A

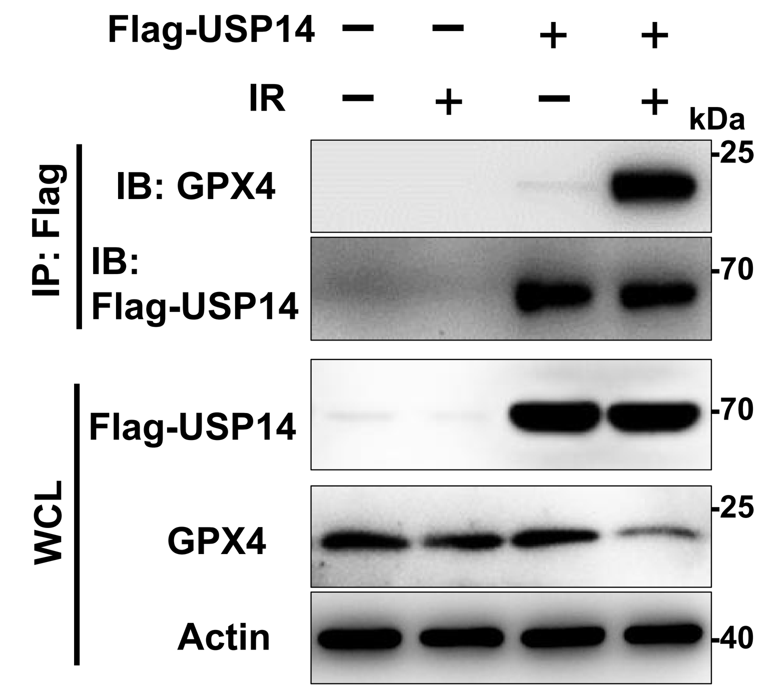

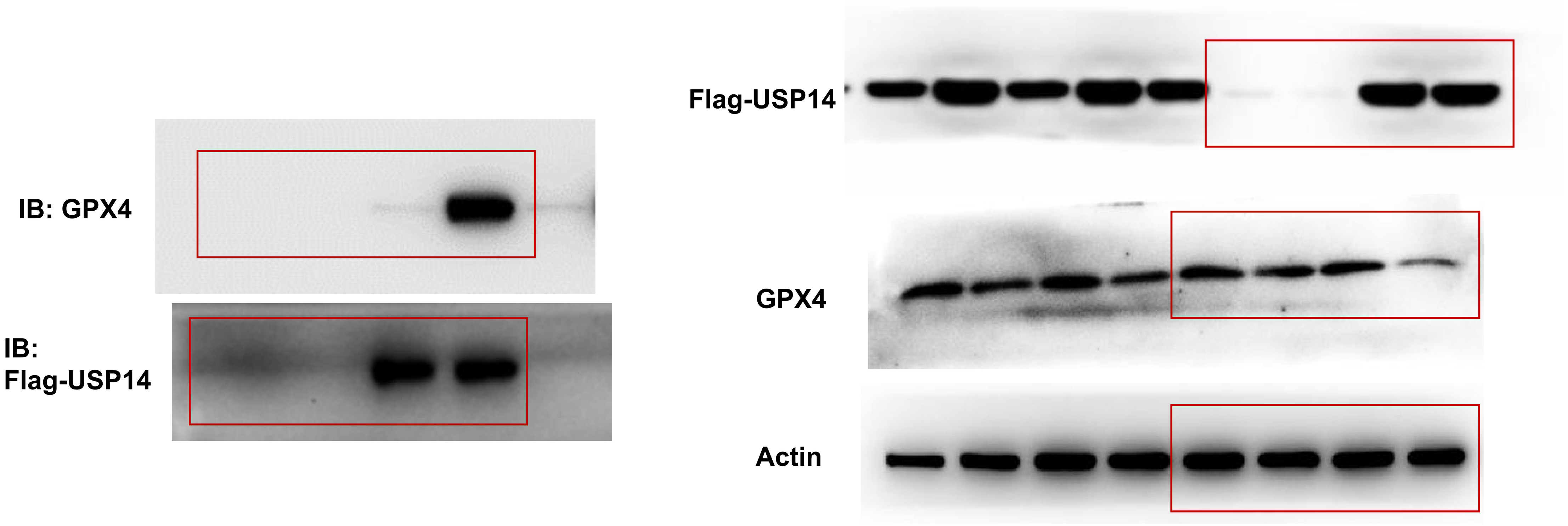

Figure 5B

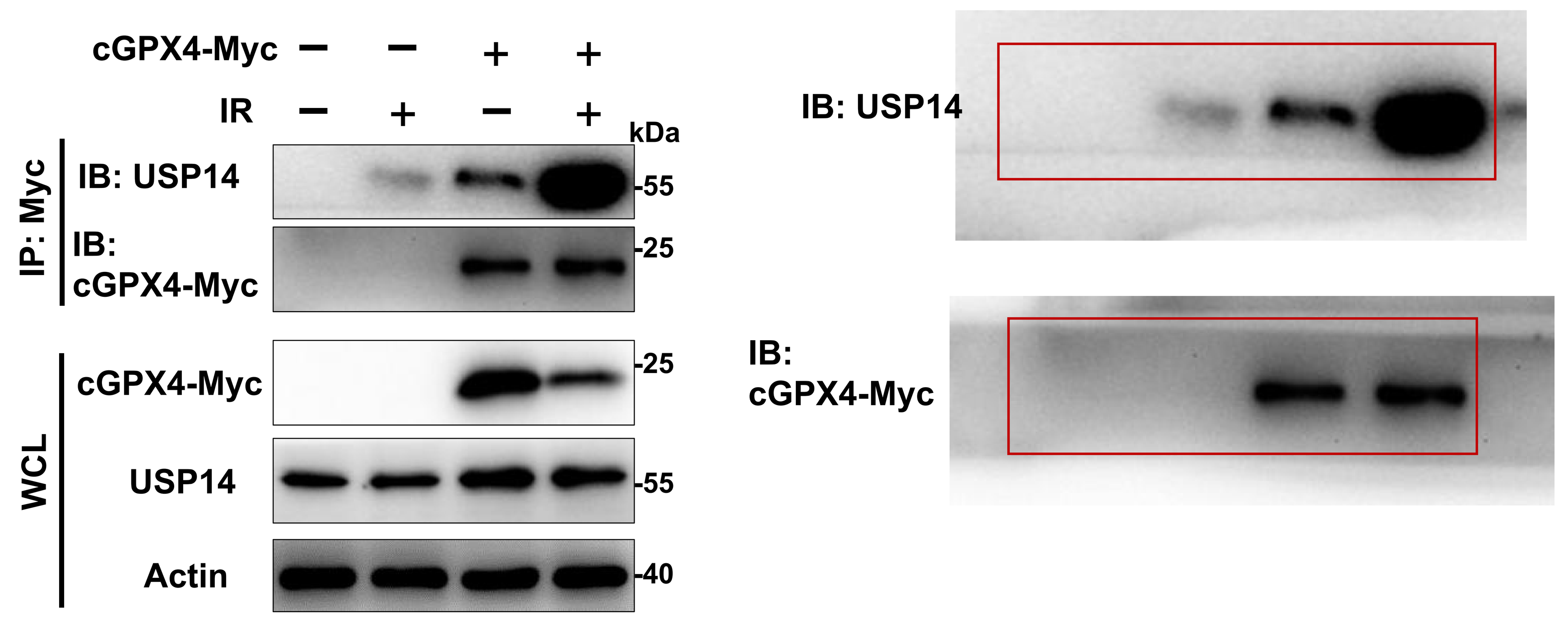

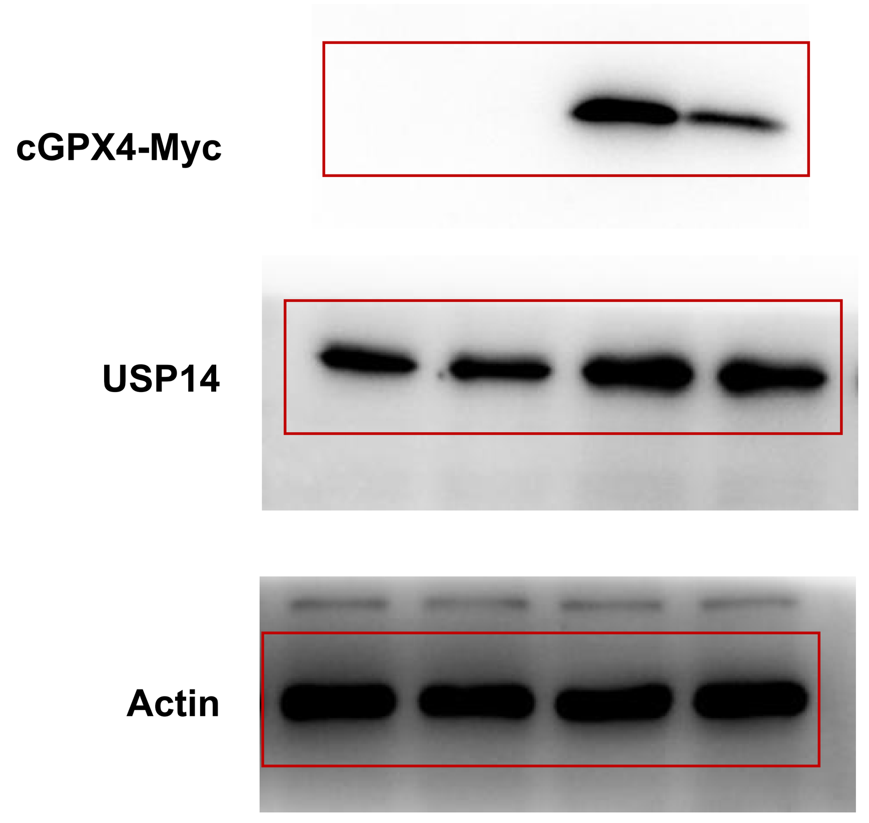

Figure 5C

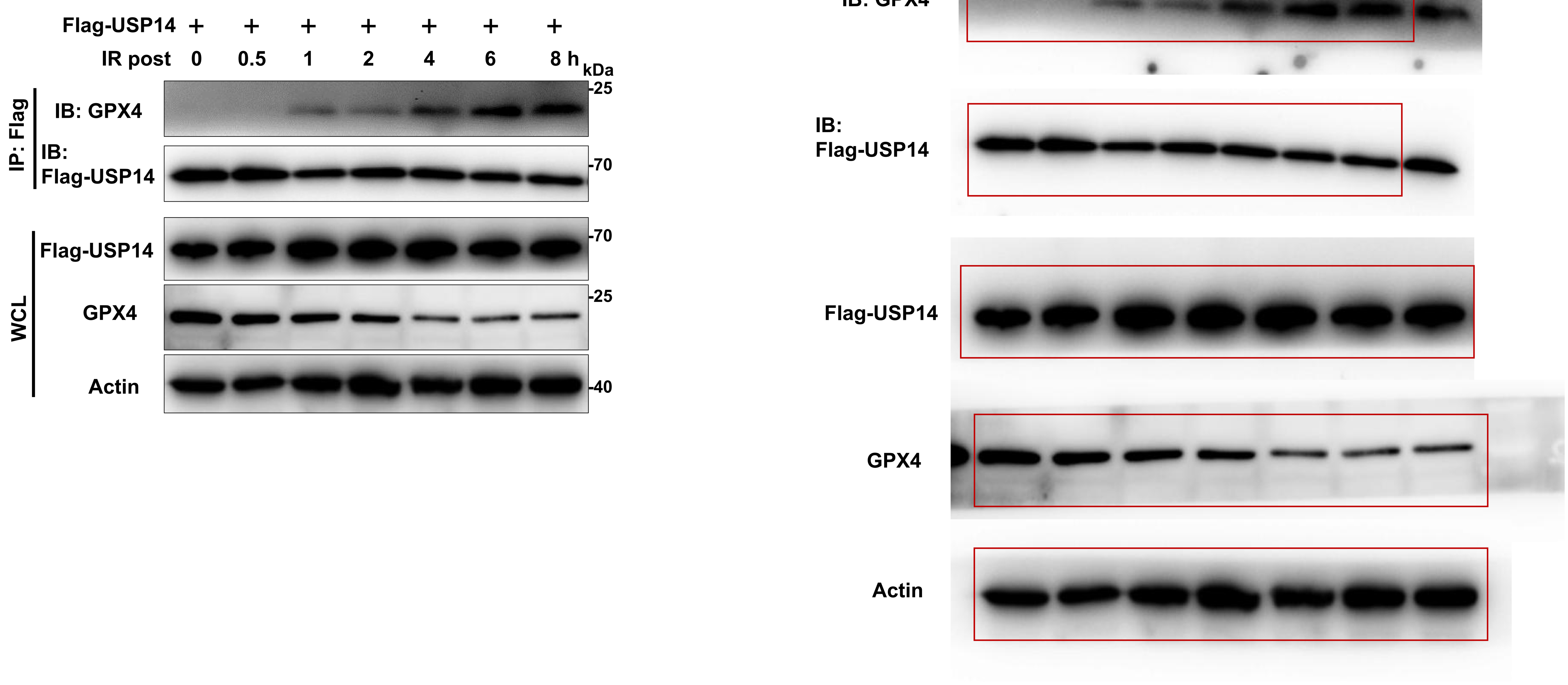

Figure 5D

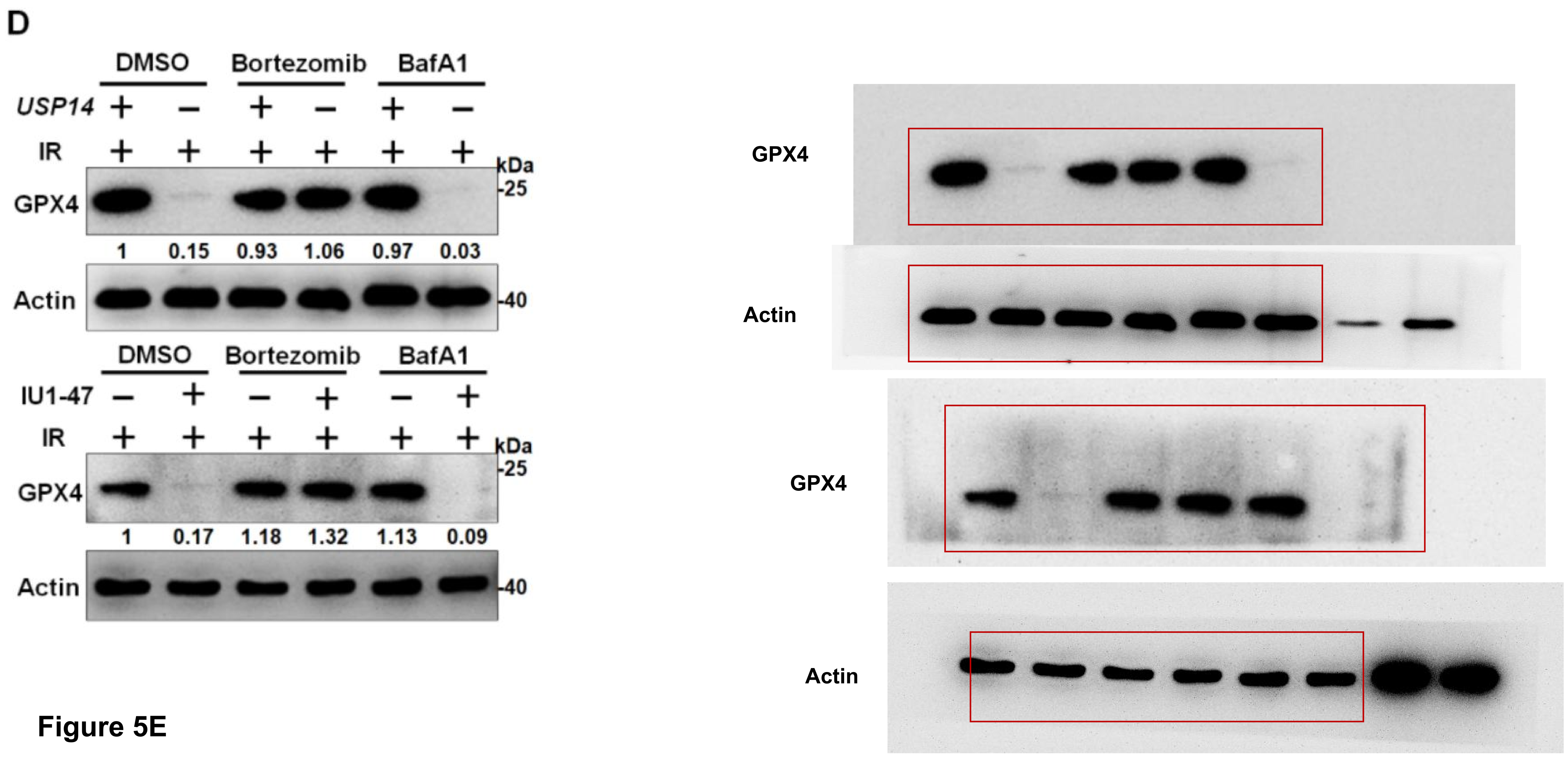

Figure 5E

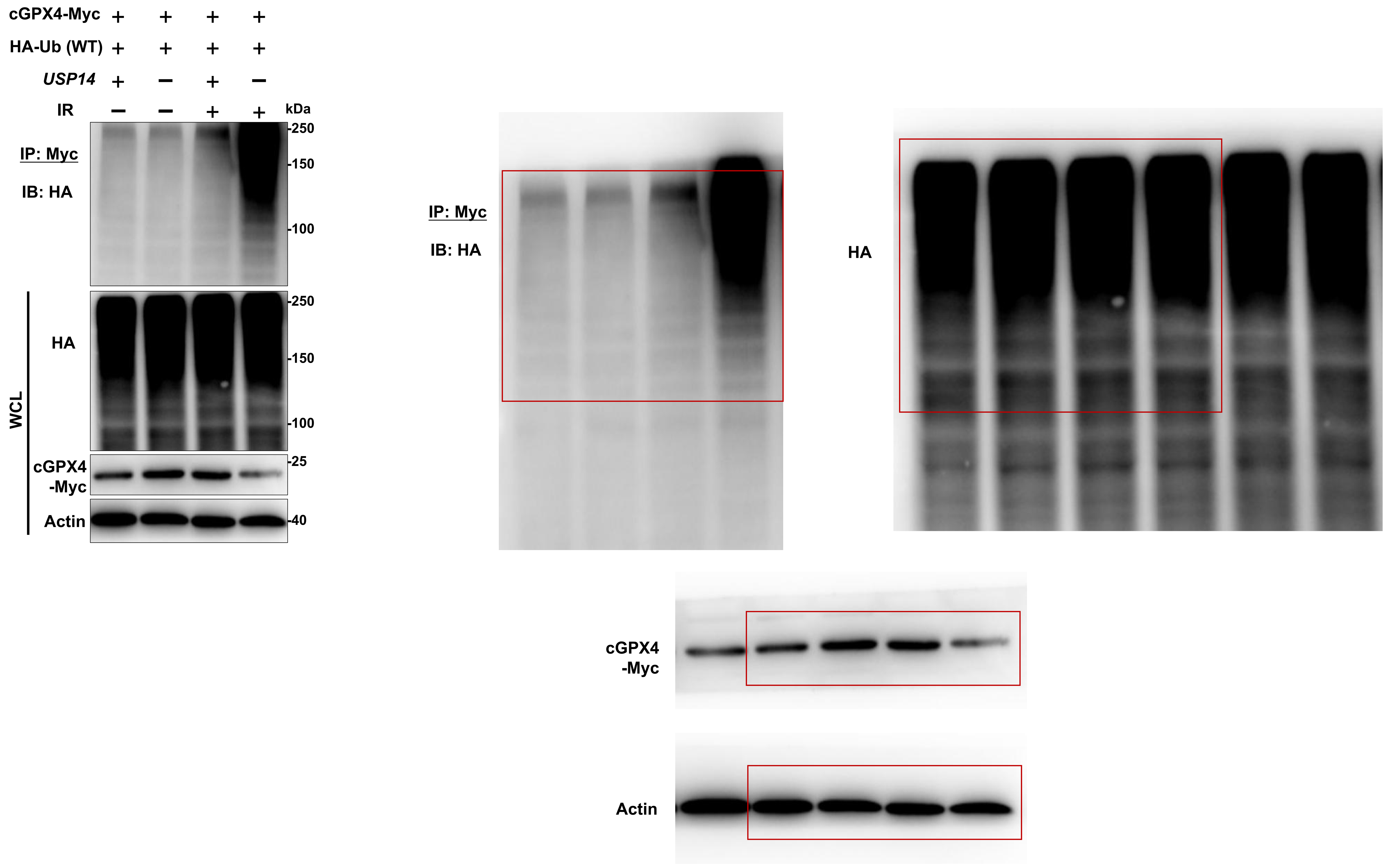

Figure 5F

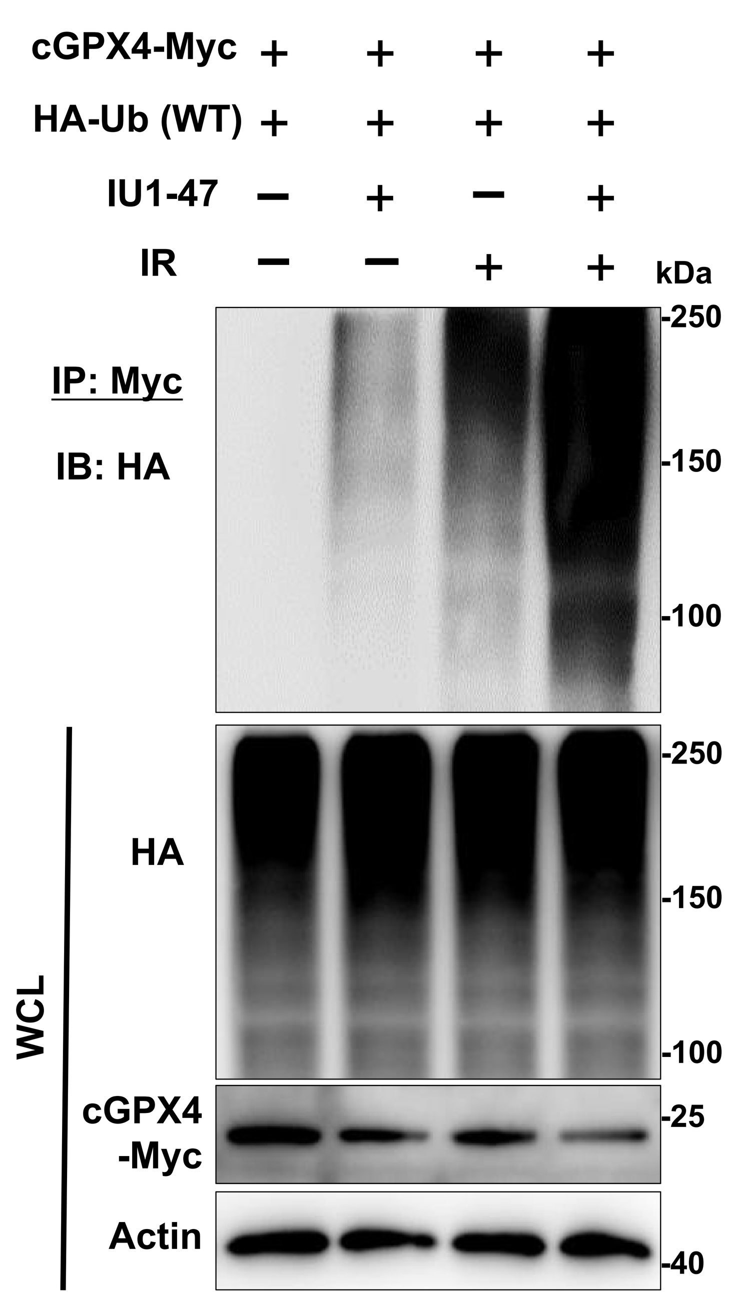

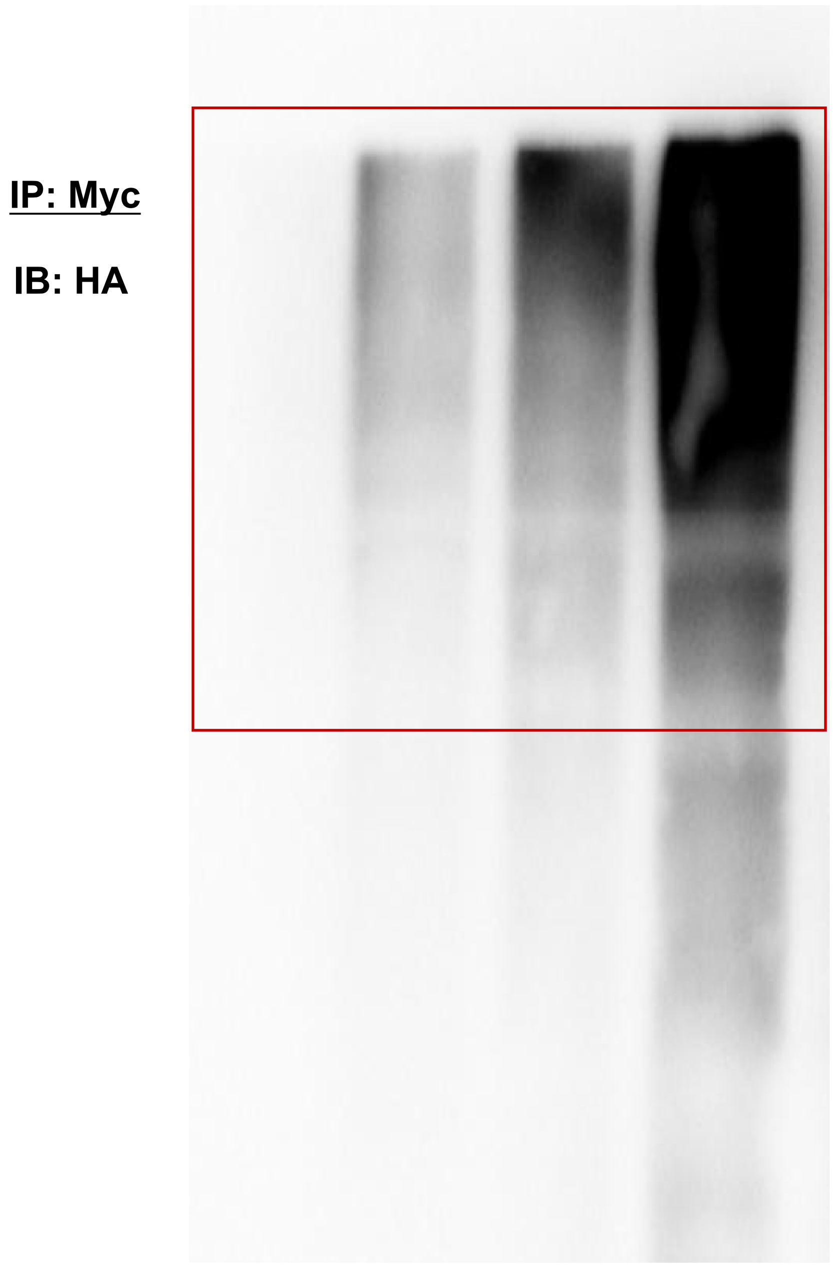

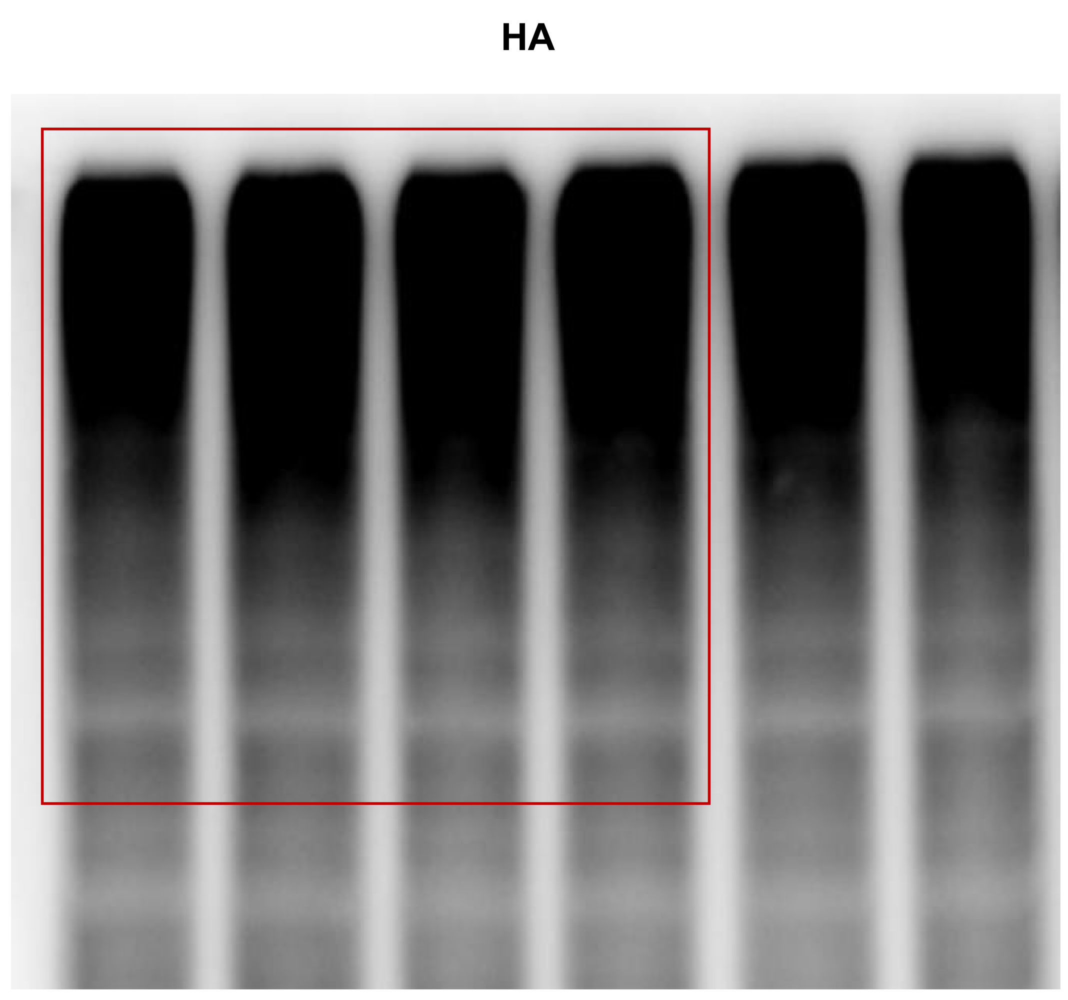

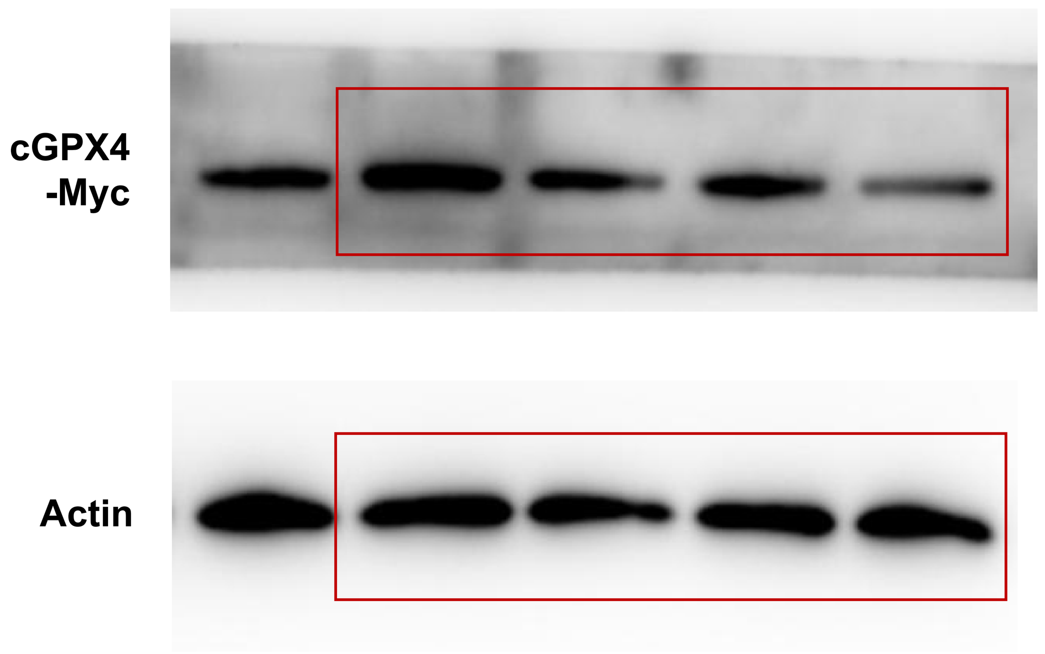

Figure 5G

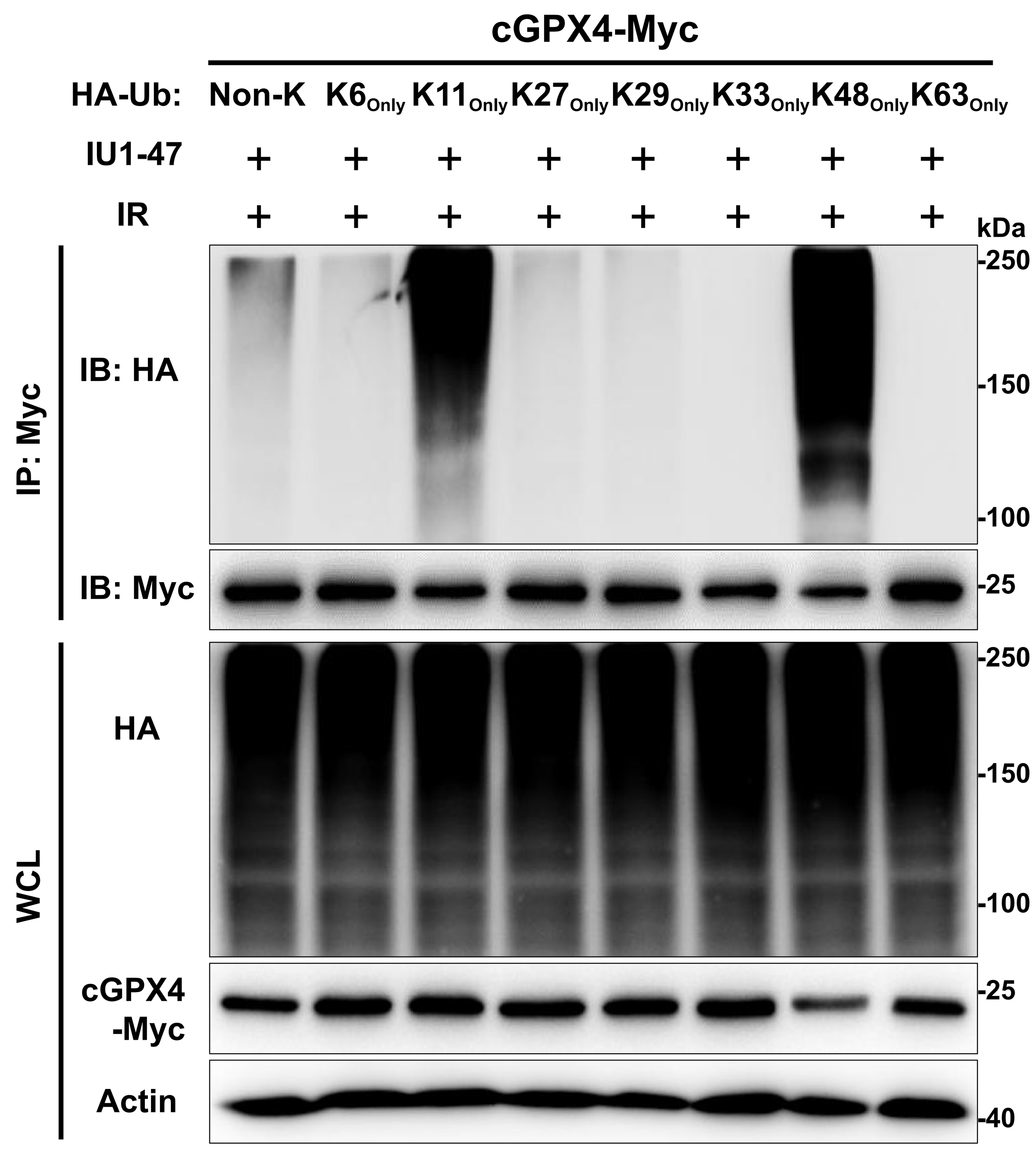

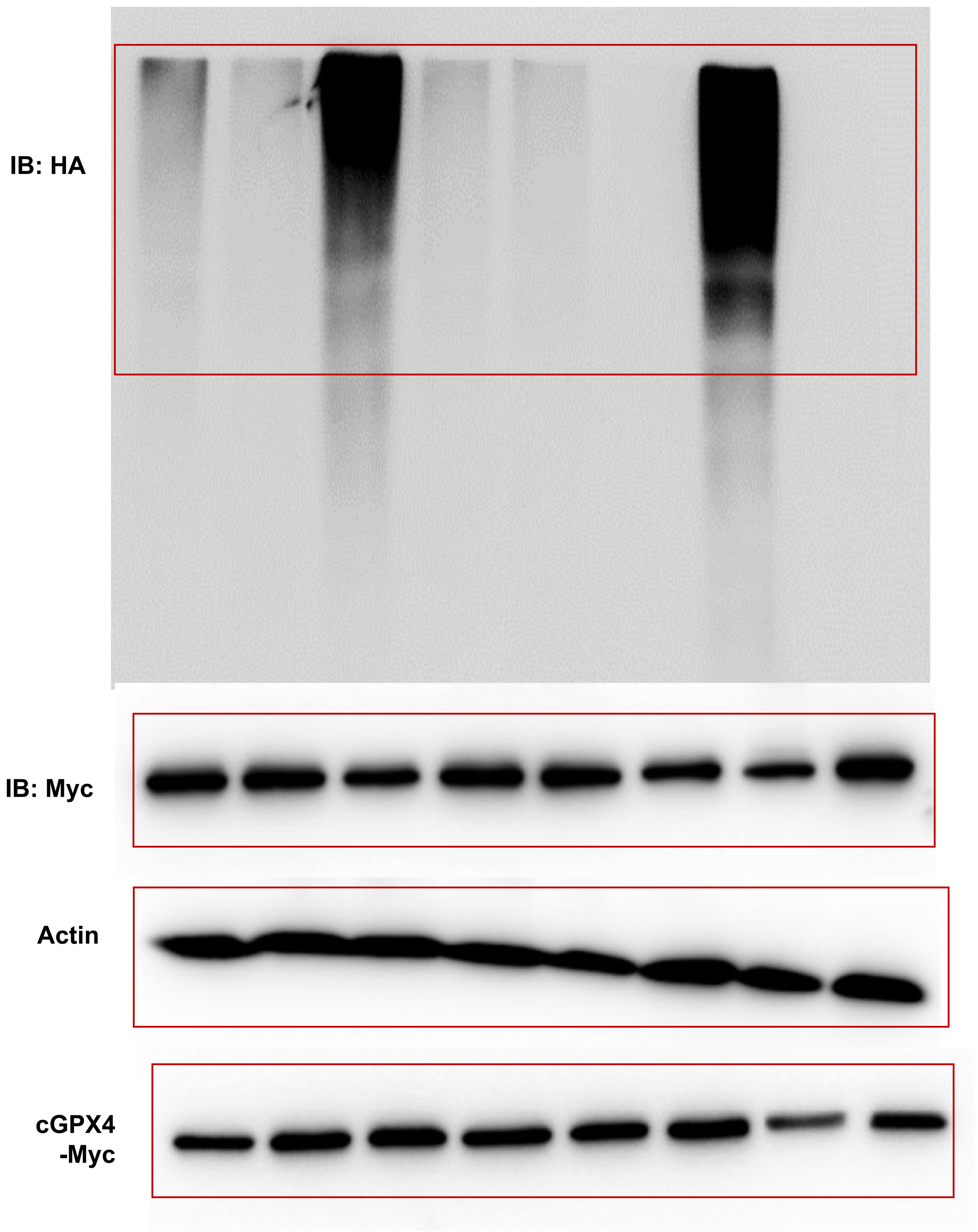

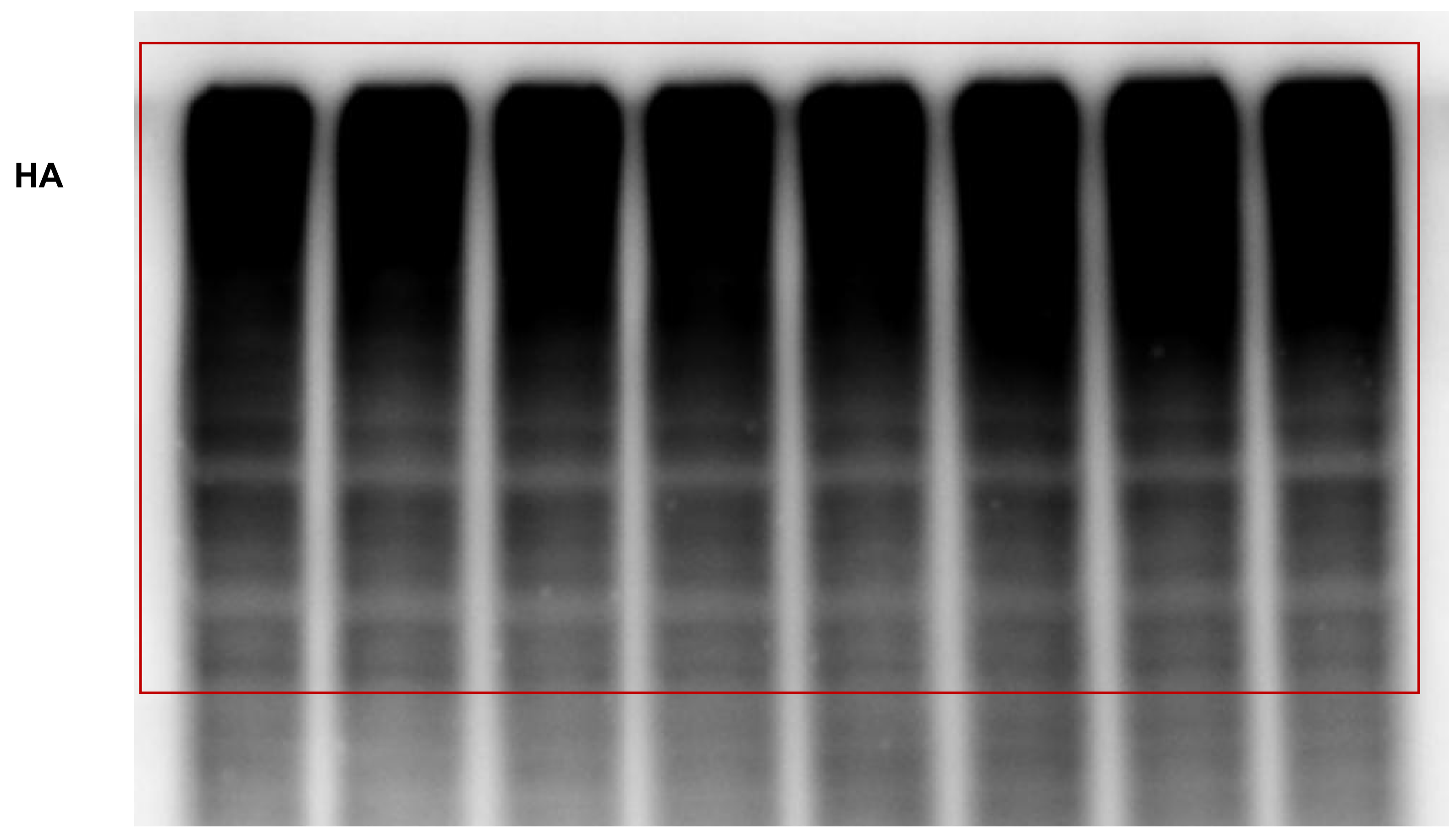

Figure 5H

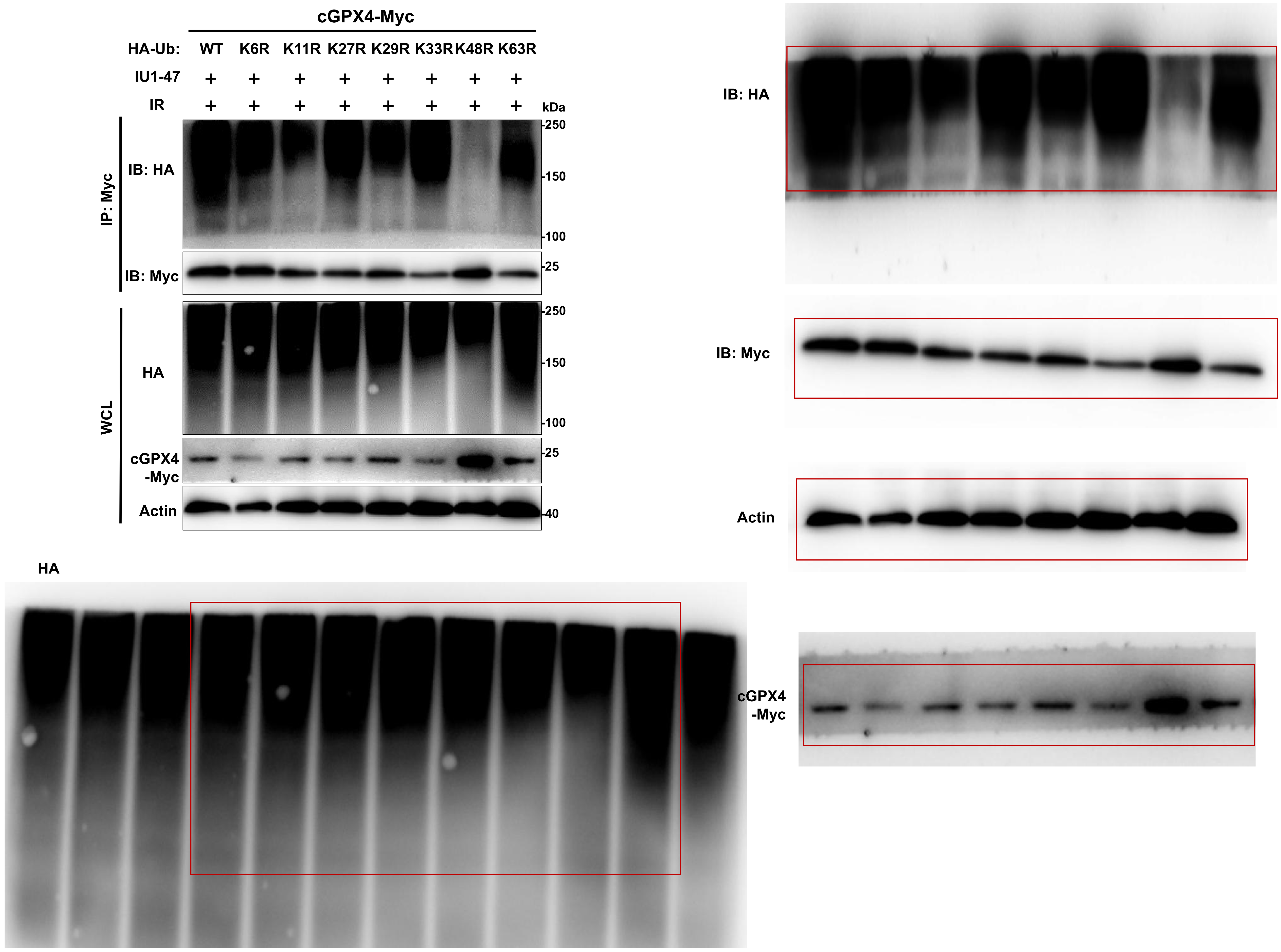

Figure 5I

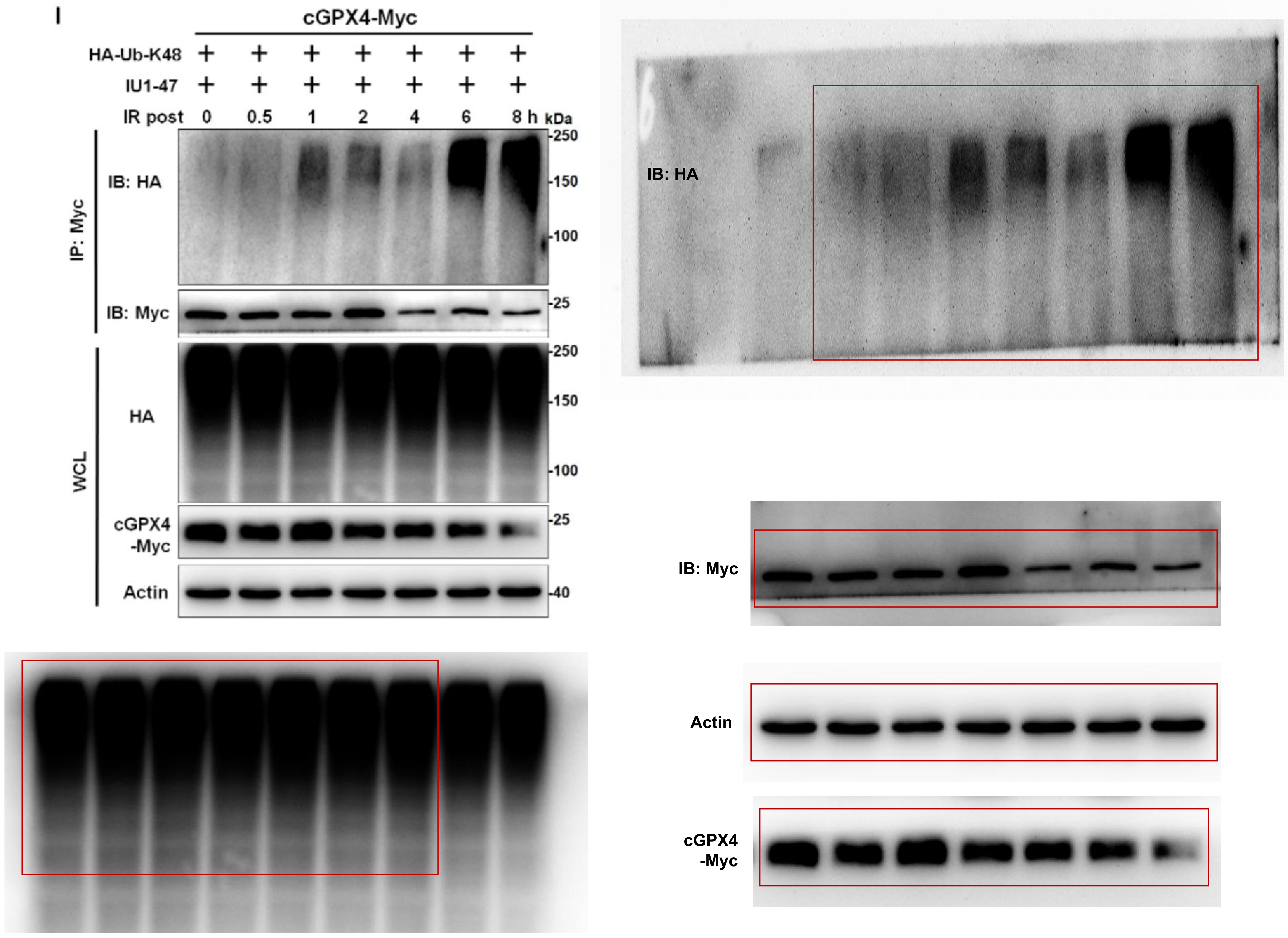

Figure 5J

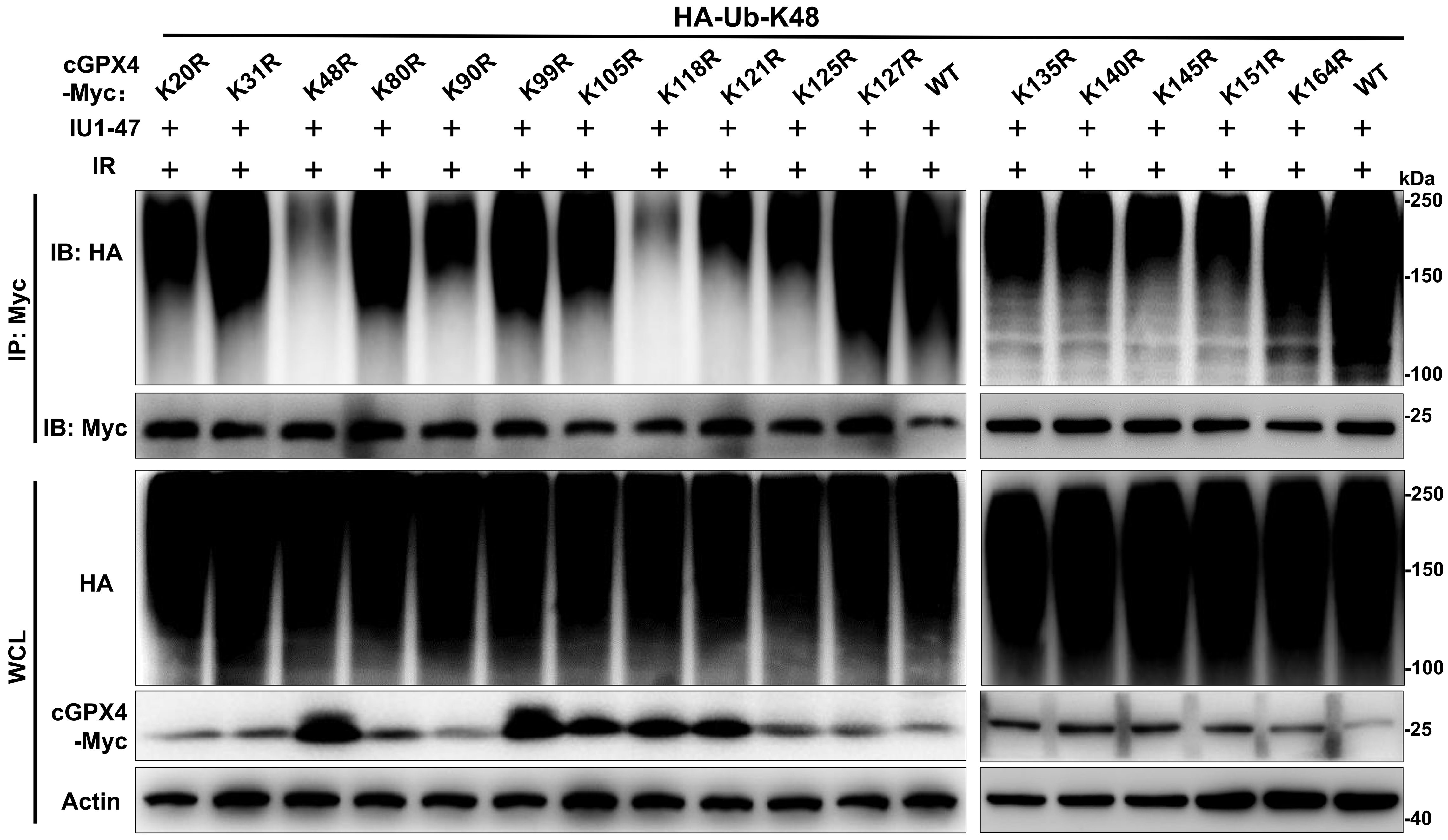

IB: HA

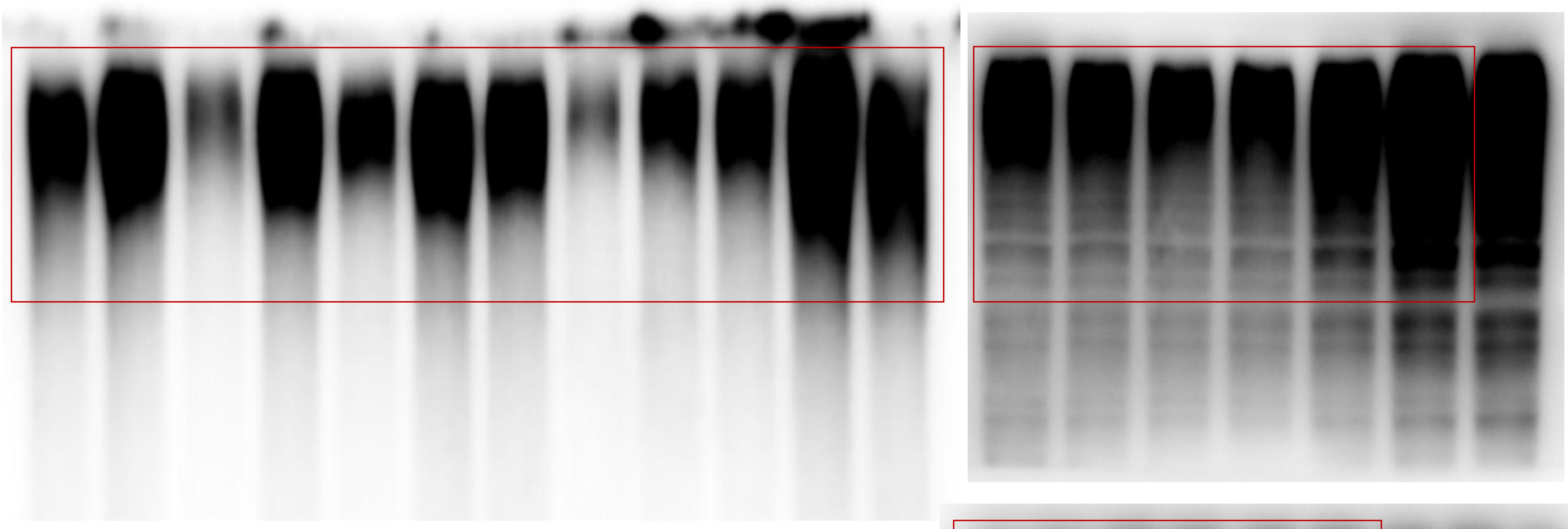

HA

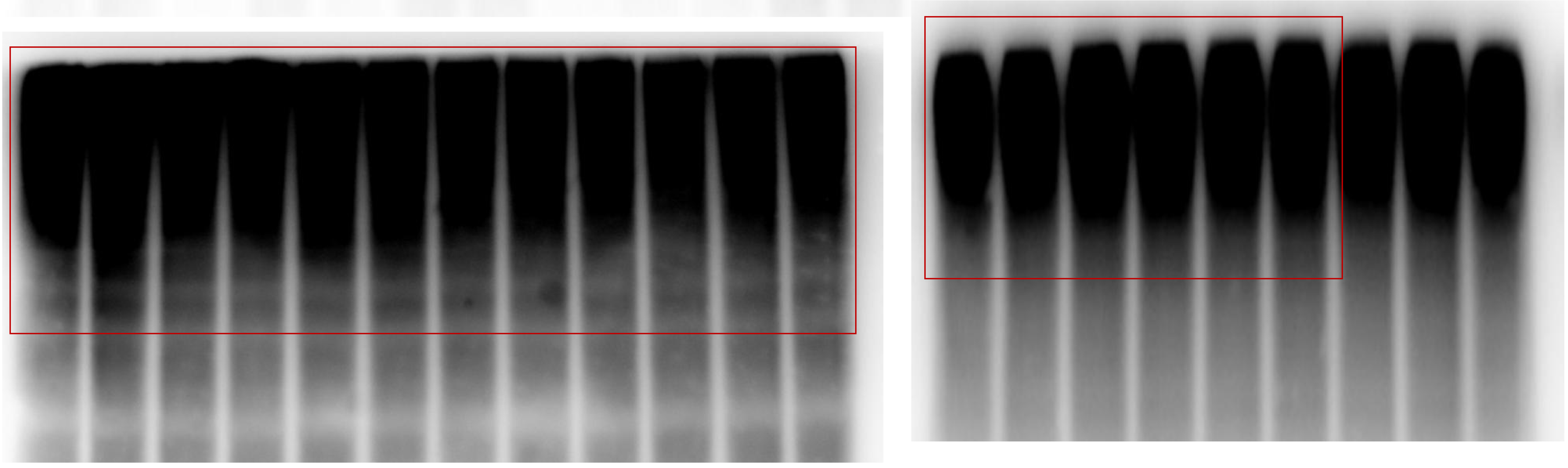

IB: Myc

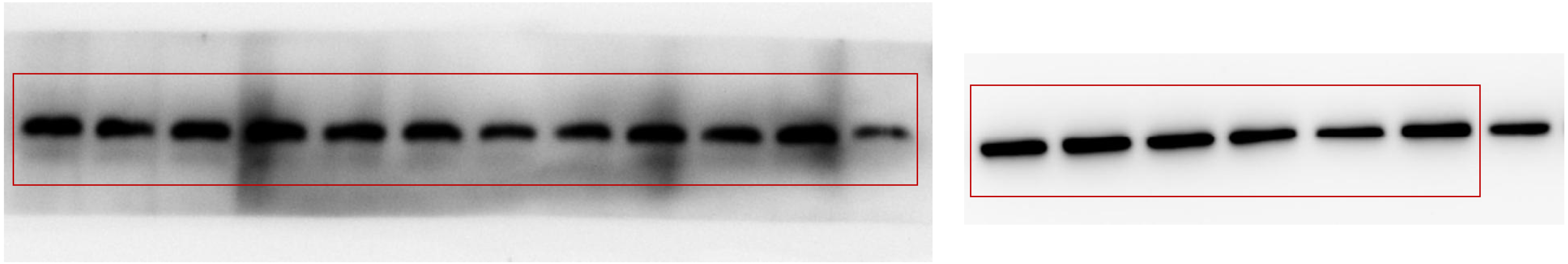

cGPX4
-Myc

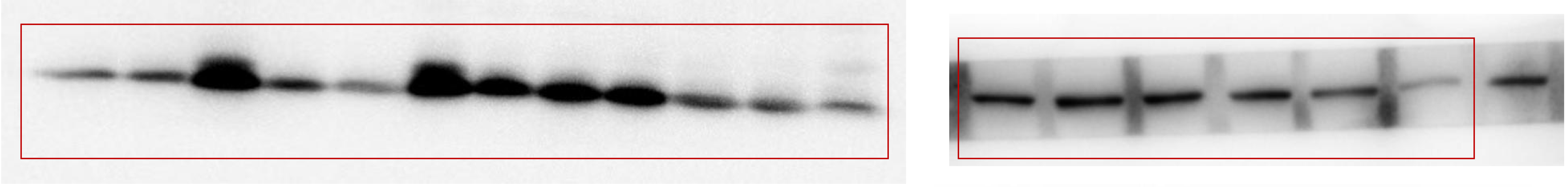

Actin

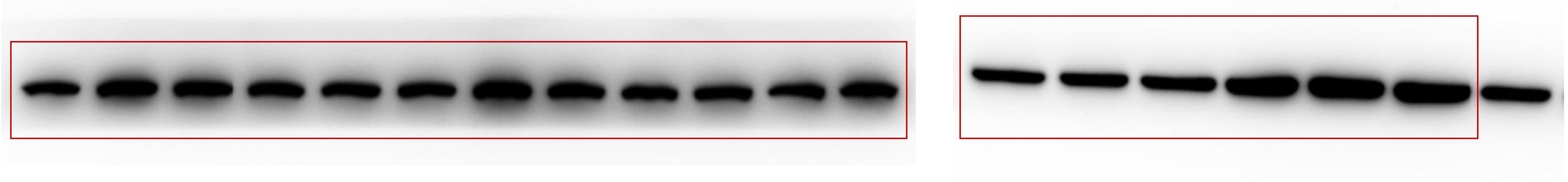

Figure 5K

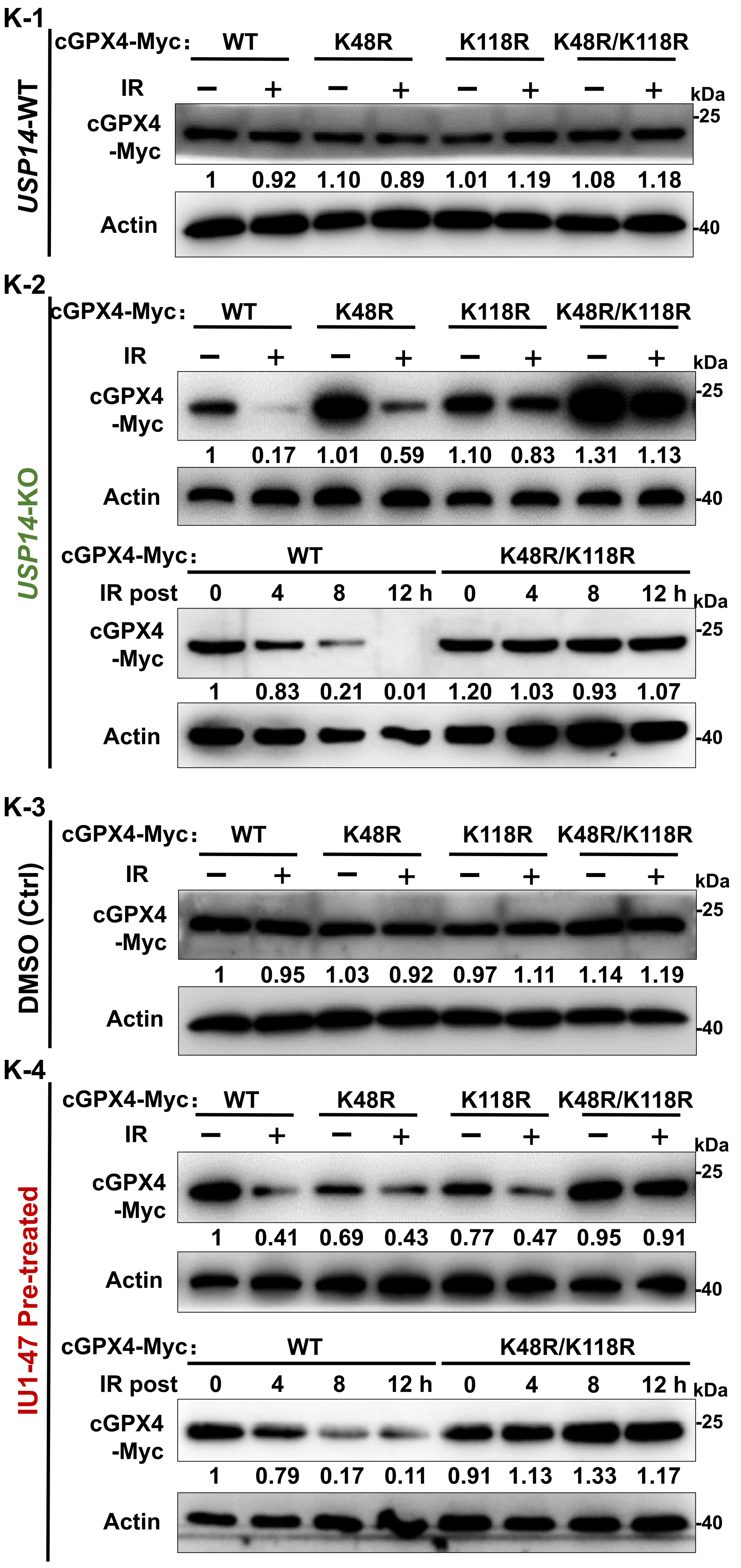

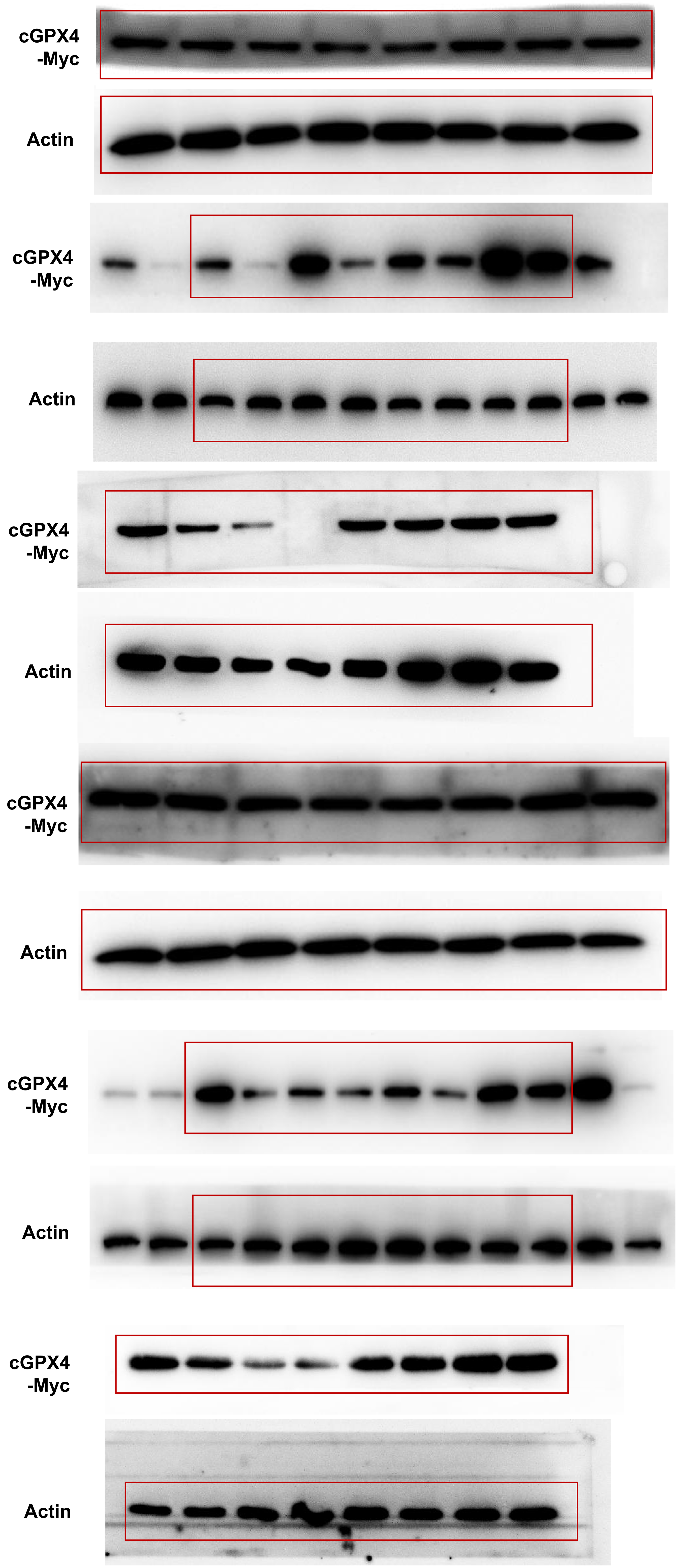

Figure 6A

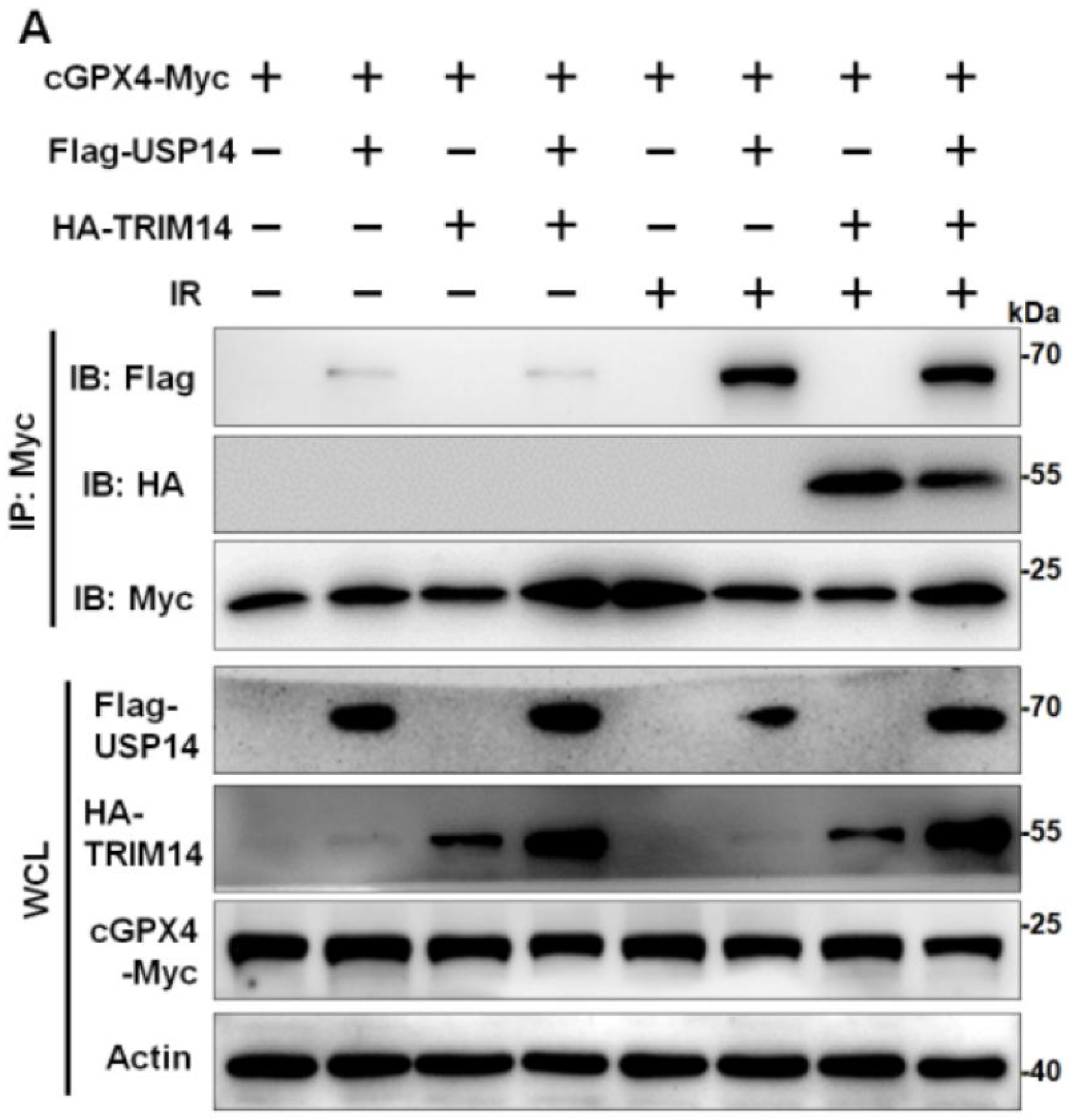

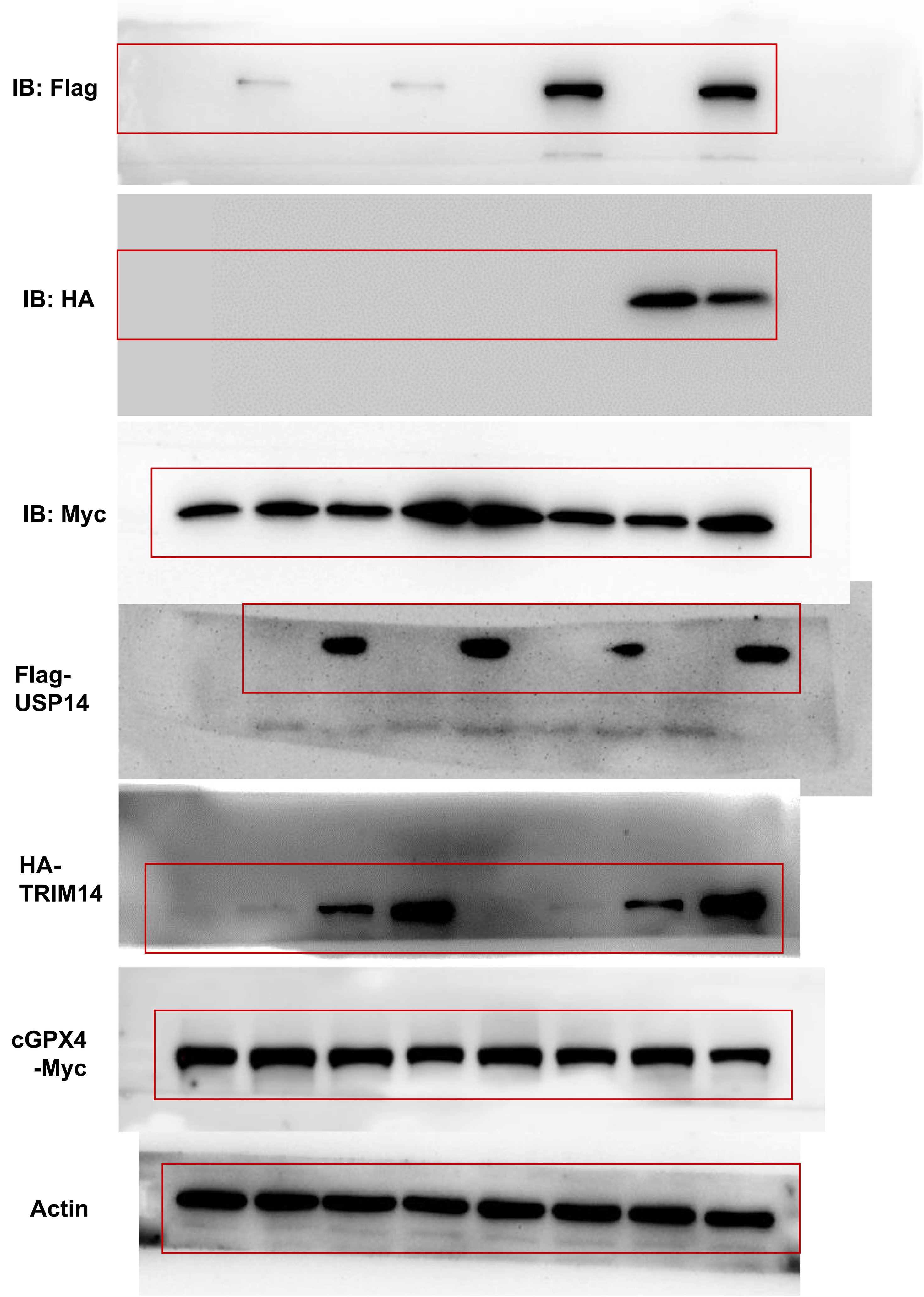

Figure 6B

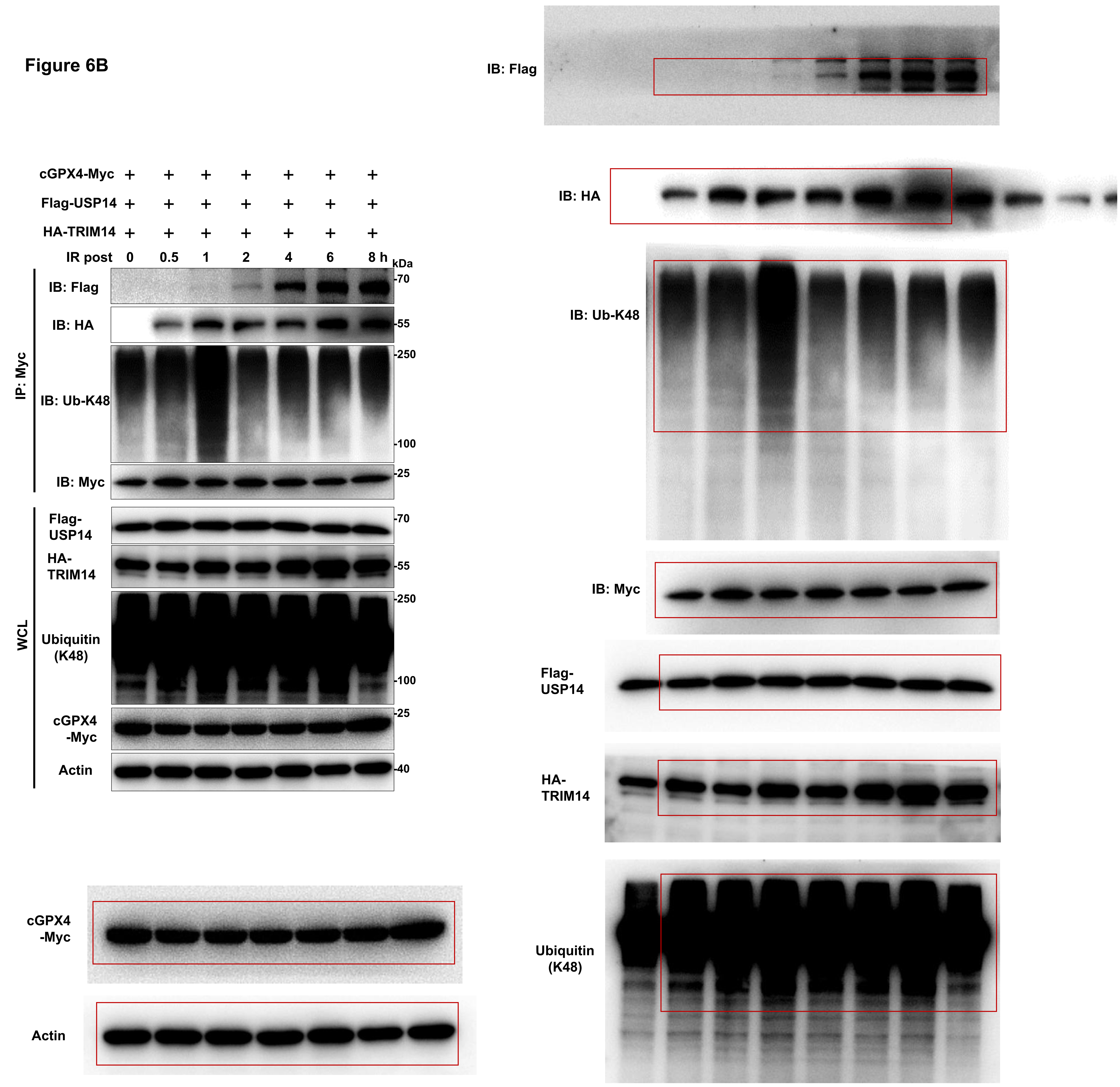

cGPX4-Myc + + + + + + +
Flag-USP14 + + + + + + +
HA-TRIM14 + + + + + + +
IR post 0 0.5 1 2 4 6 8 h
kDa
IP: Myc
IB: Flag
IB: HA
IB: Ub-K48
IB: Myc
WCL
Flag-USP14
HA-TRIM14
Ubiquitin (K48)
cGPX4 -Myc
Actin
70
55
250
100
25
40
IB: Flag
IB: HA
IB: Ub-K48
IB: Myc
Flag-USP14
HA-TRIM14
Ubiquitin (K48)
cGPX4 -Myc
Actin

Figure 6C

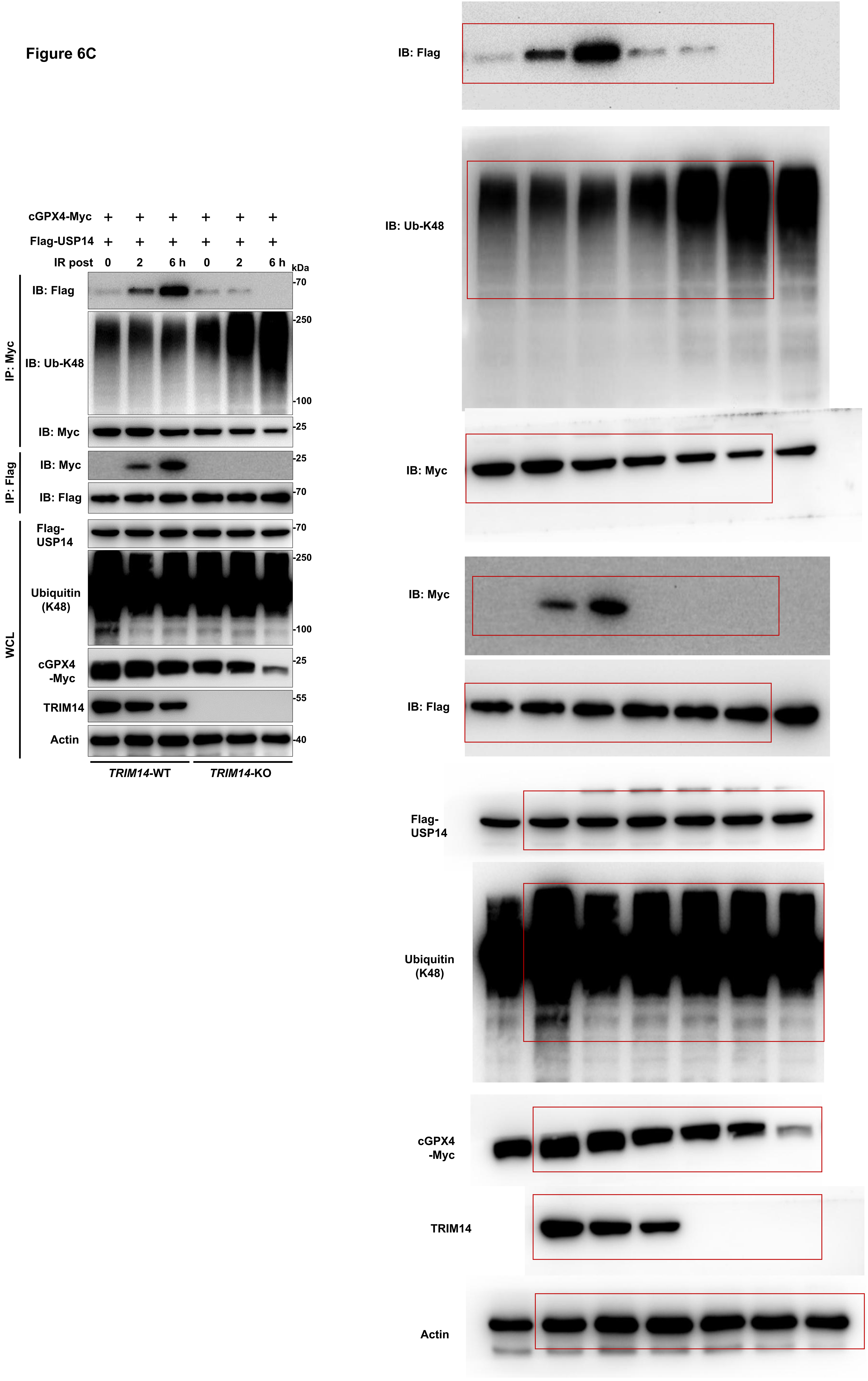
cGPX4-Myc + + + + + +
Flag-USP14 + + + + + +
IR post 0 2 6 h 0 2 6 h
kDa
IP: Myc
IB: Flag
IB: Ub-K48
IB: Myc
IP: Flag
IB: Myc
IB: Flag
WCL
Flag-USP14
Ubiquitin (K48)
cGPX4 -Myc
TRIM14
Actin
70
250
100
25
25
70
70
250
100
25
55
40
TRIM14-WT
TRIM14-KO
IB: Flag
IB: Ub-K48
IB: Myc
IB: Myc
IB: Flag
Flag-USP14
Ubiquitin (K48)
cGPX4 -Myc
TRIM14
Actin

Figure 6D

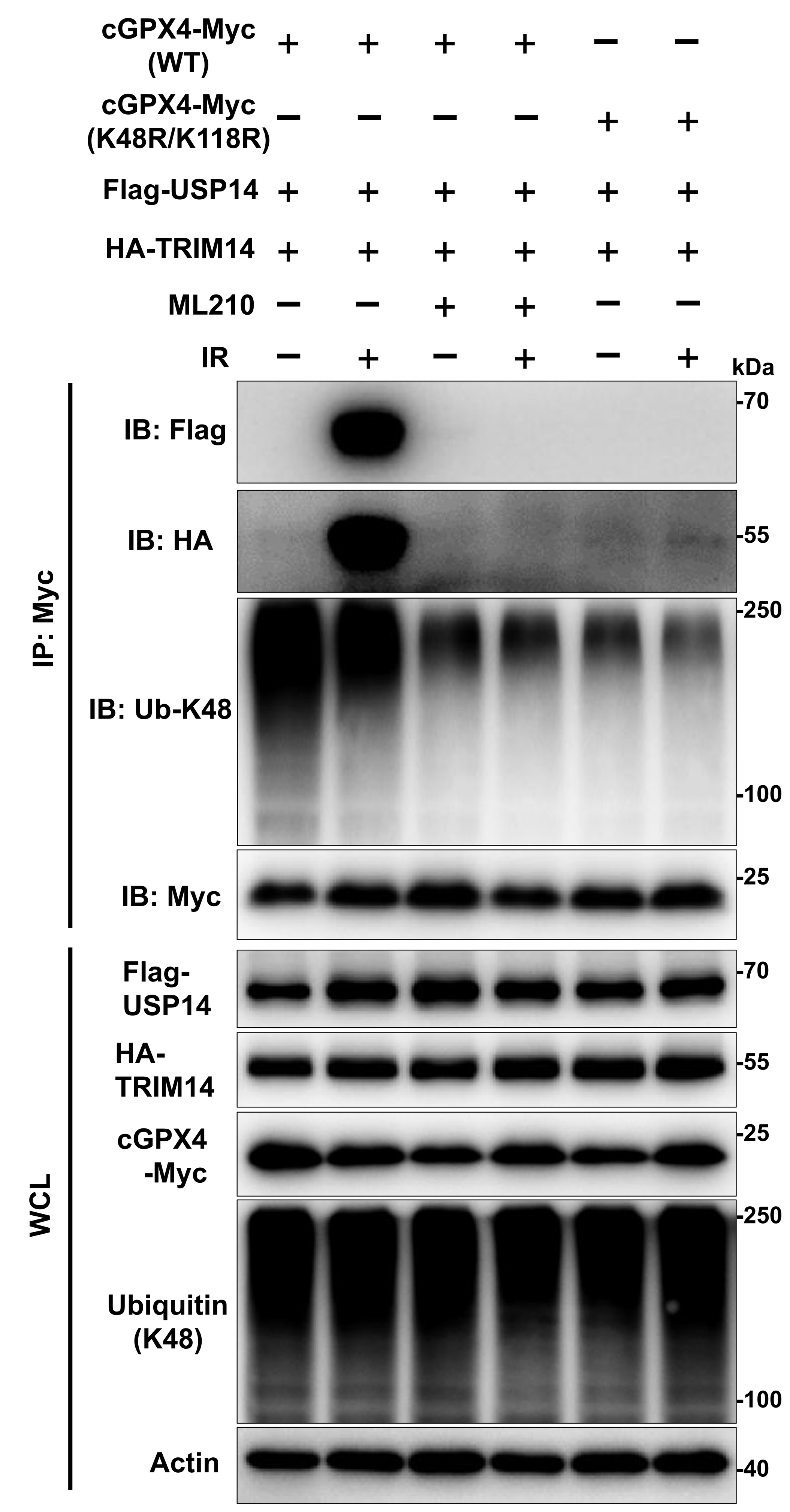

IB: Flag

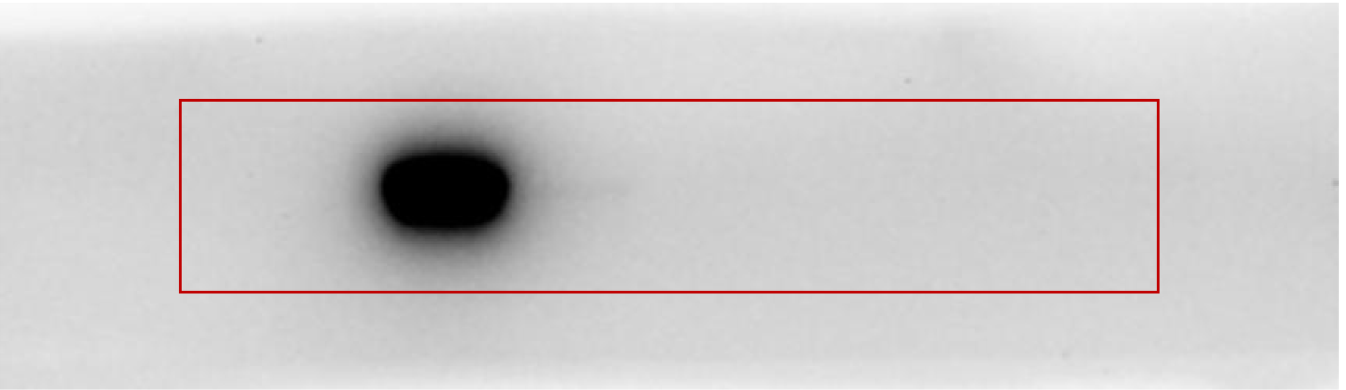

IB: HA

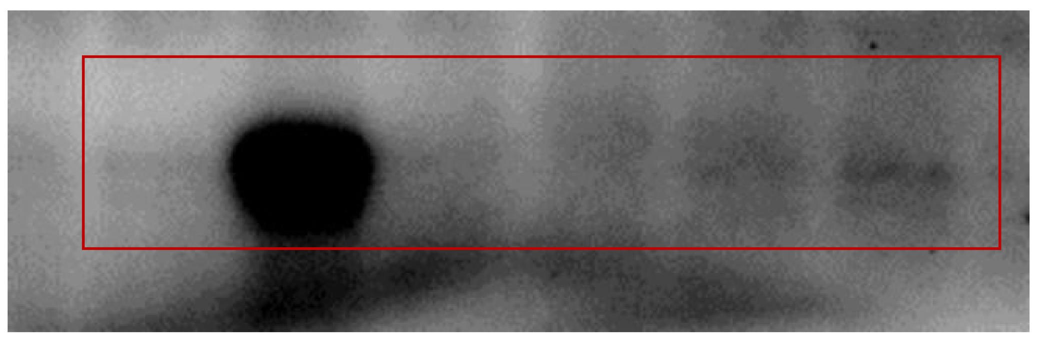

IB: Myc

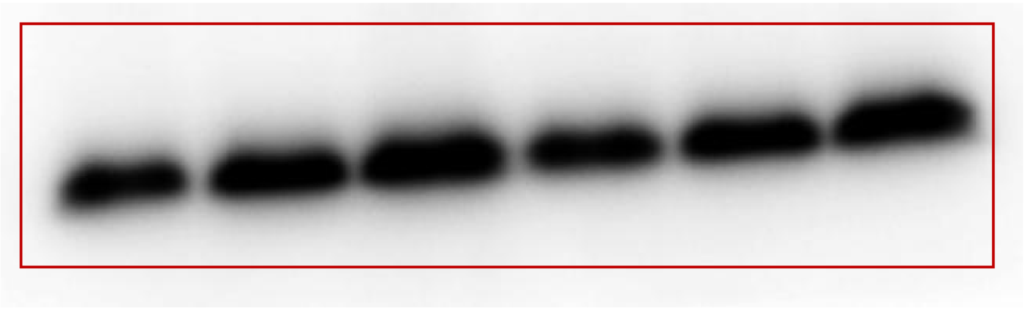

Flag-USP14

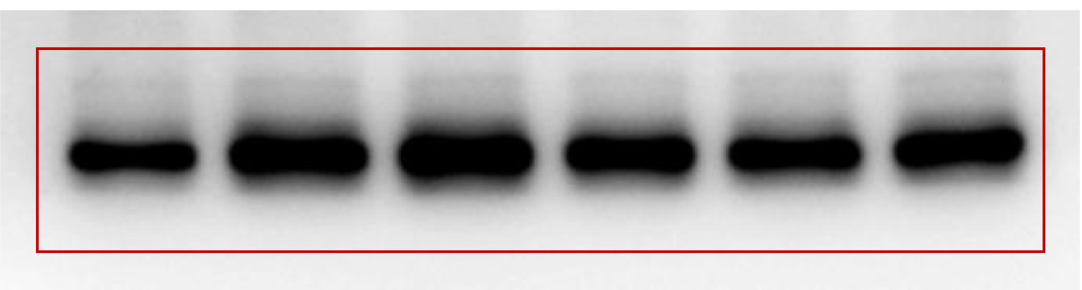

HA-TRIM14

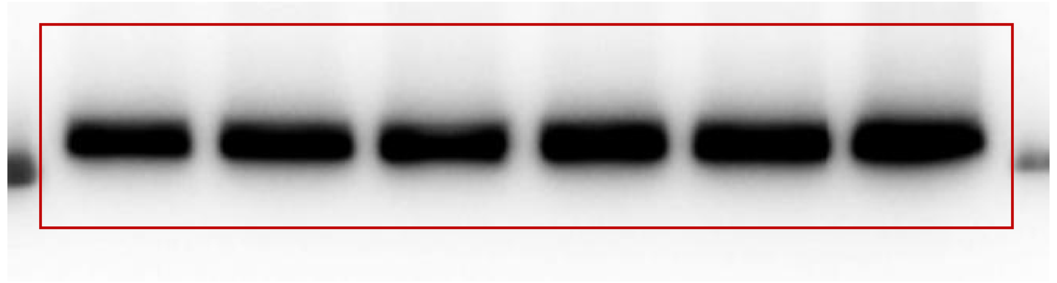

cGPX4 -Myc

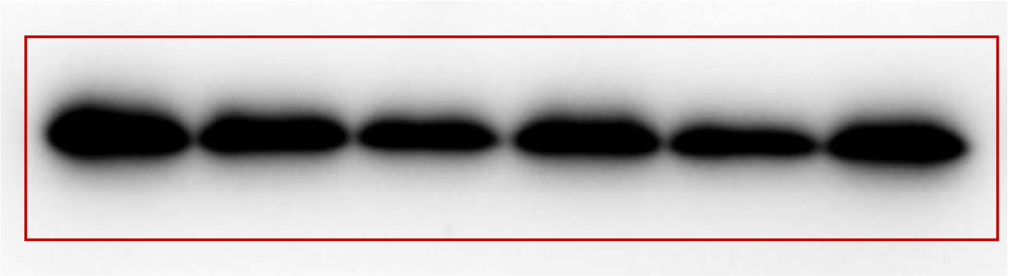

Actin

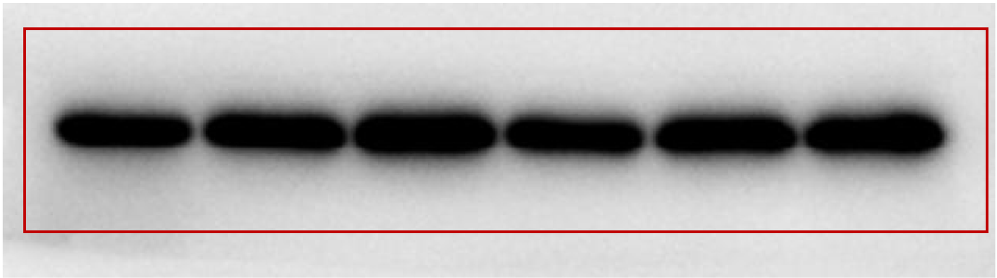

Ubiquitin (K48)

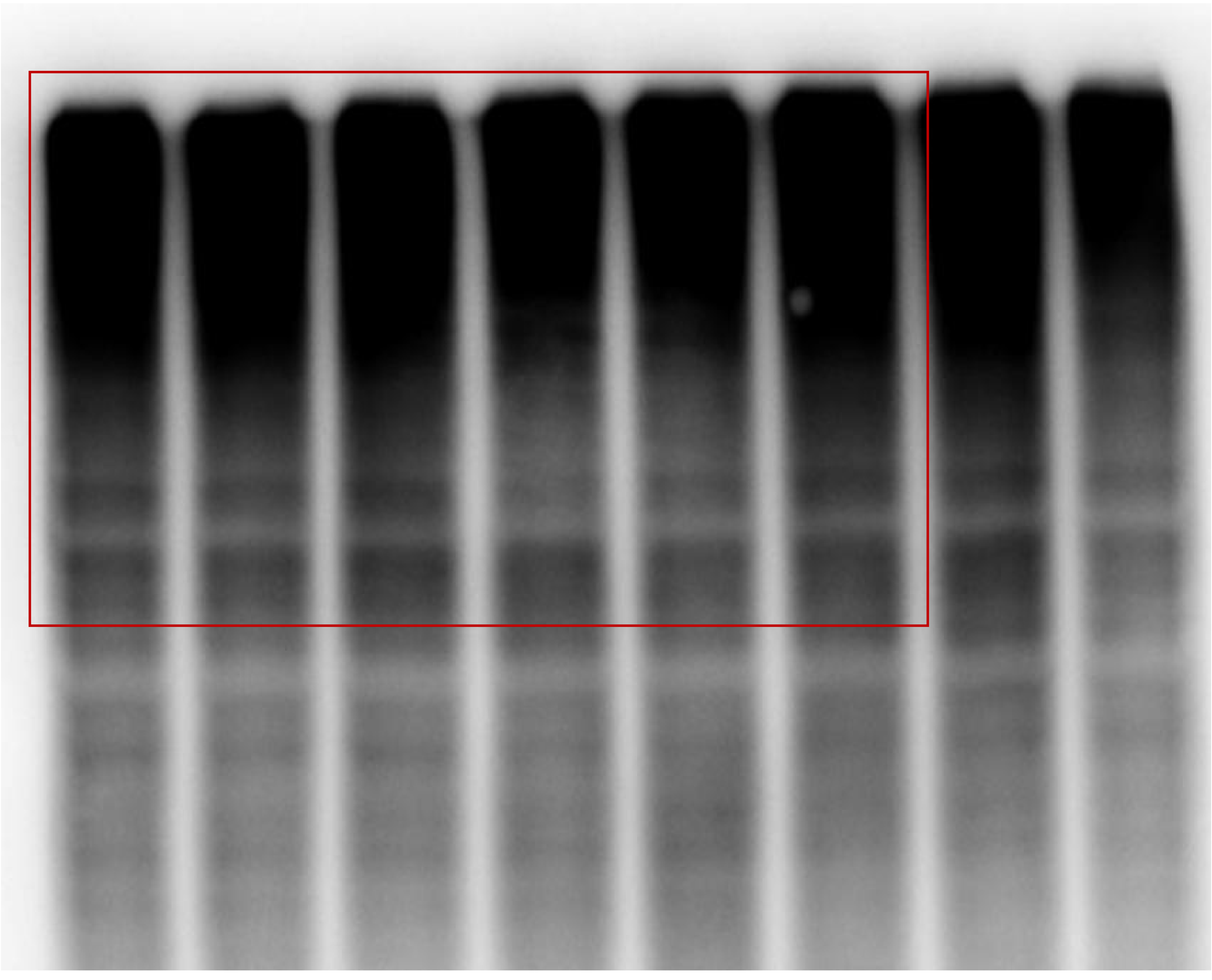

Figure 6G

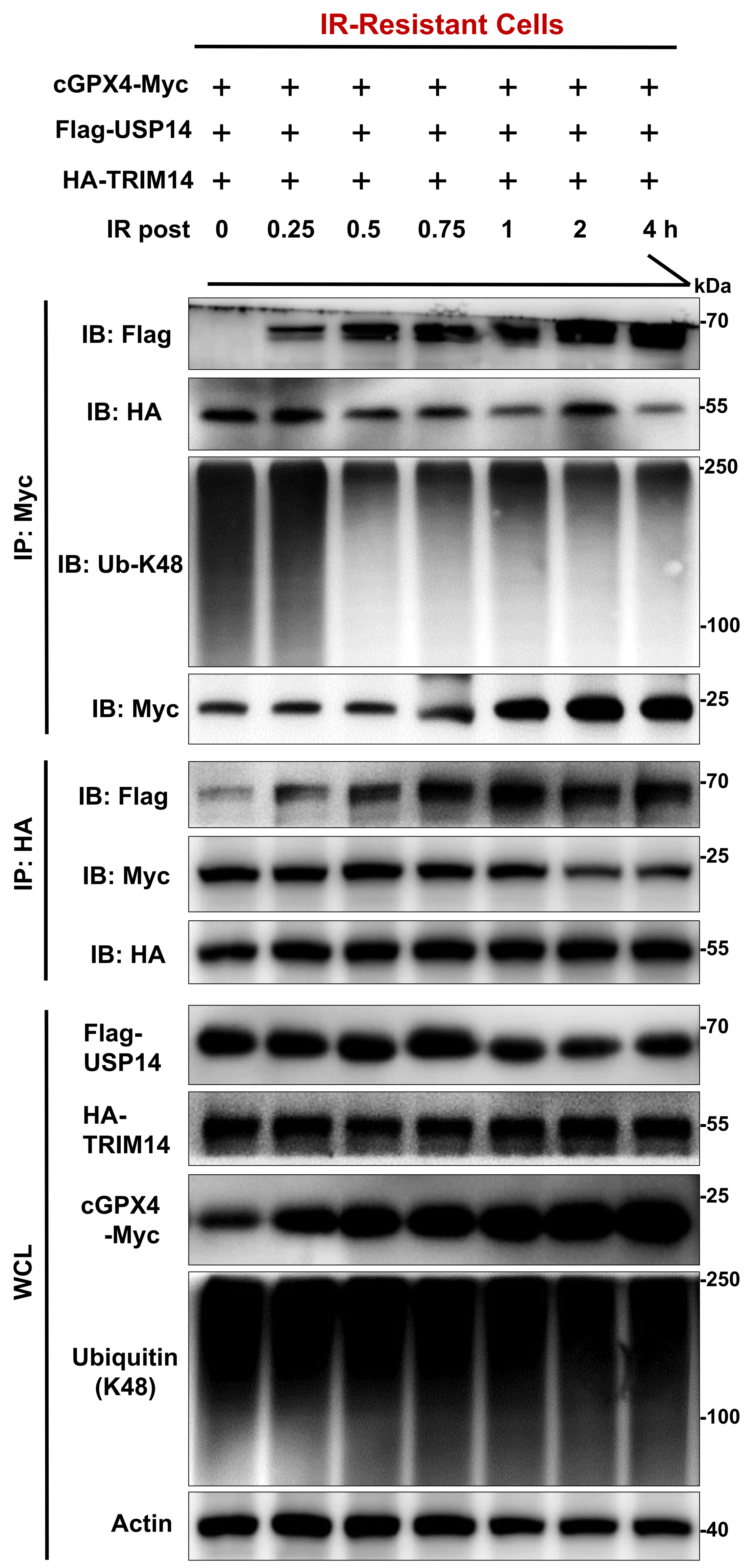
IR-Resistant Cells
cGPX4-Myc + + + + + + +
Flag-USP14 + + + + + + +
HA-TRIM14 + + + + + + +
IR post 0 0.25 0.5 0.75 1 2 4 h
kDa
IP: Myc
IB: Flag
IB: HA
IB: Ub-K48
IB: Myc
IP: HA
IB: Flag
IB: Myc
IB: HA
WCL
Flag-USP14
HA-TRIM14
cGPX4-Myc
Ubiquitin (K48)
Actin
70
55
250
100
25
70
25
55
70
55
25
250
100
40

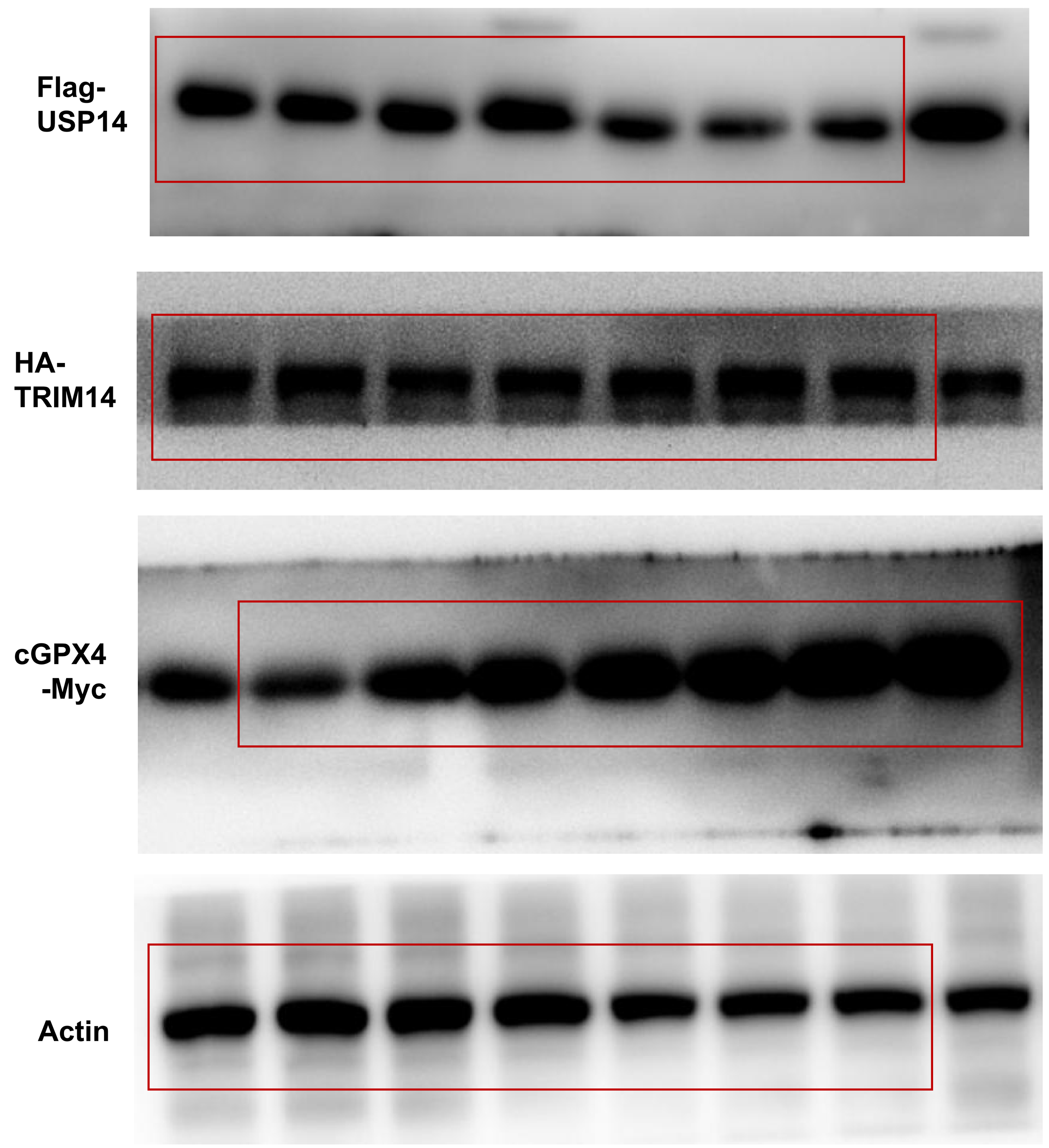
Flag-USP14
HA-TRIM14
cGPX4-Myc
Actin

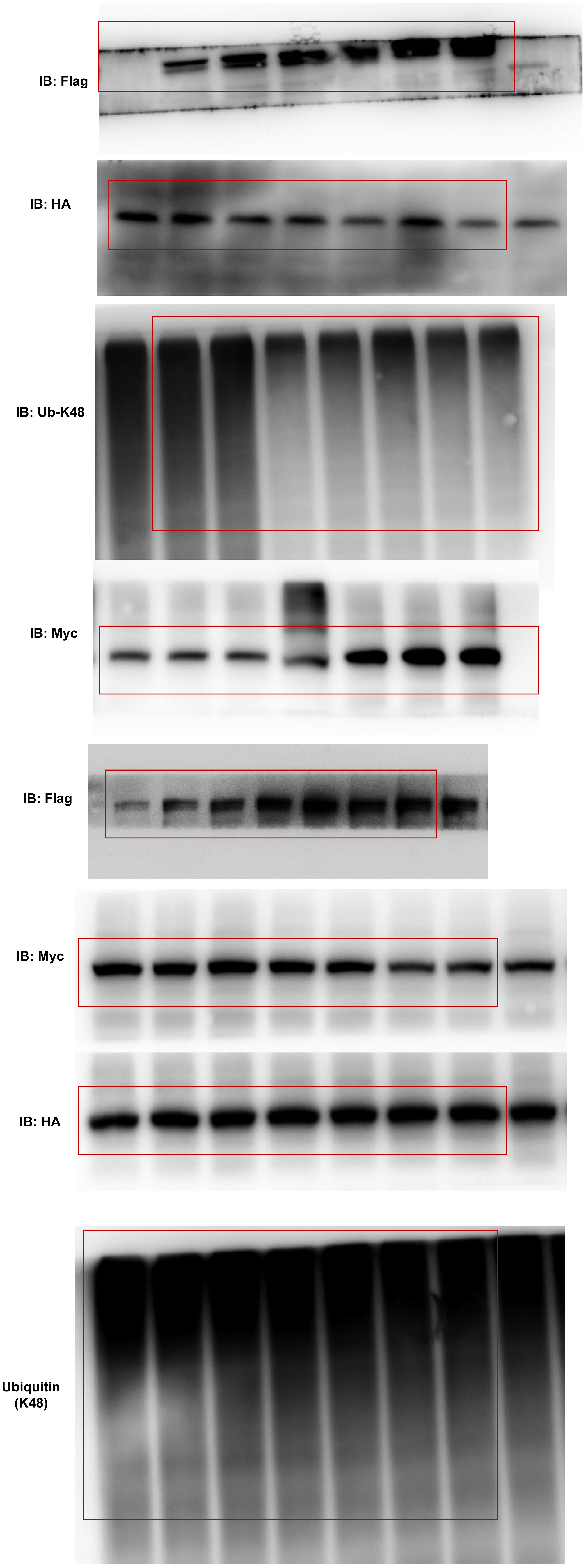
IB: Flag
IB: HA
IB: Ub-K48
IB: Myc
IB: Flag
IB: Myc
IB: HA
Ubiquitin (K48)

Figure S3B

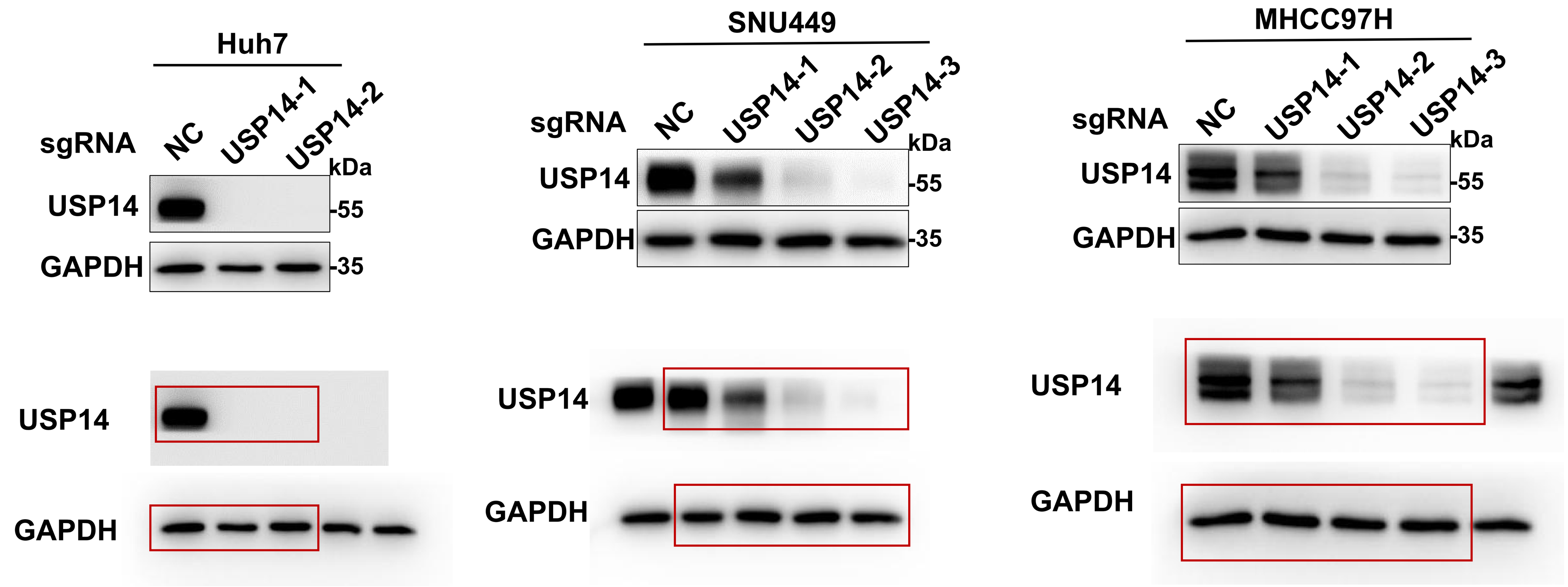

Figure S4A

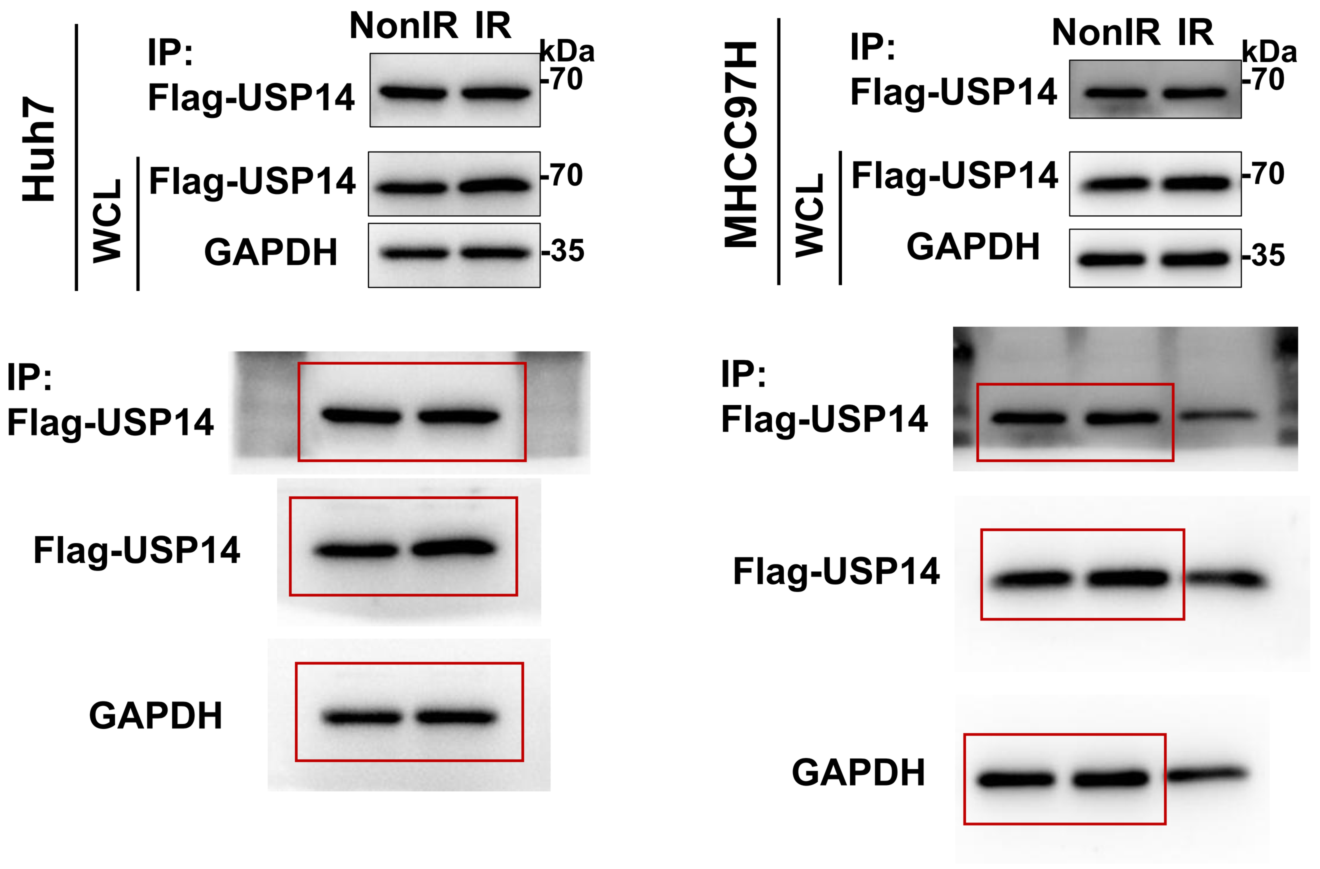

Figure S4B

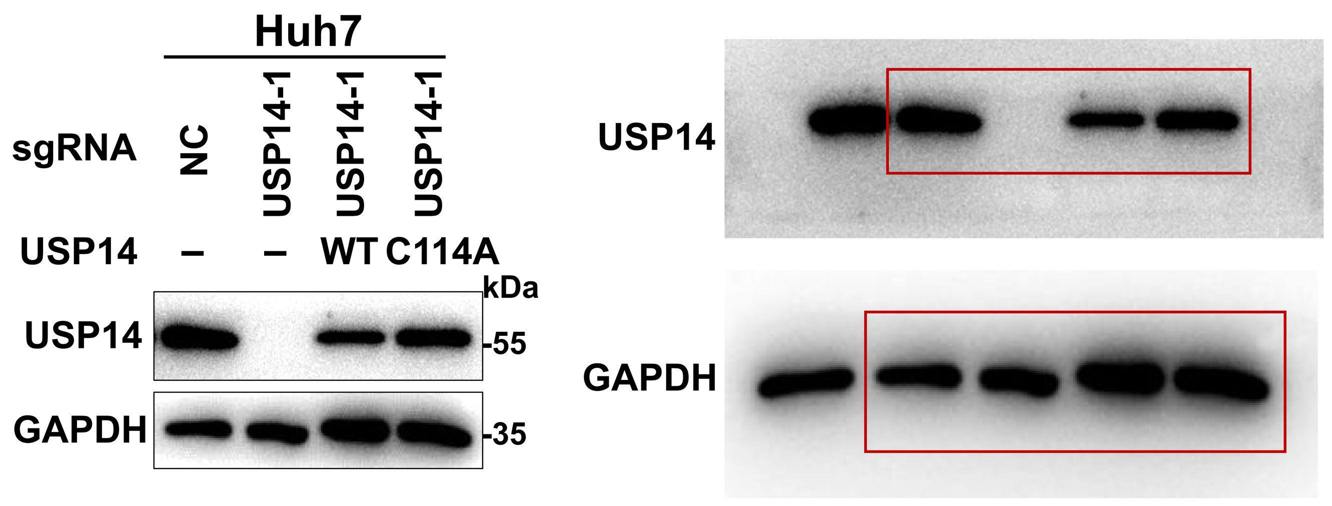

Figure S4C

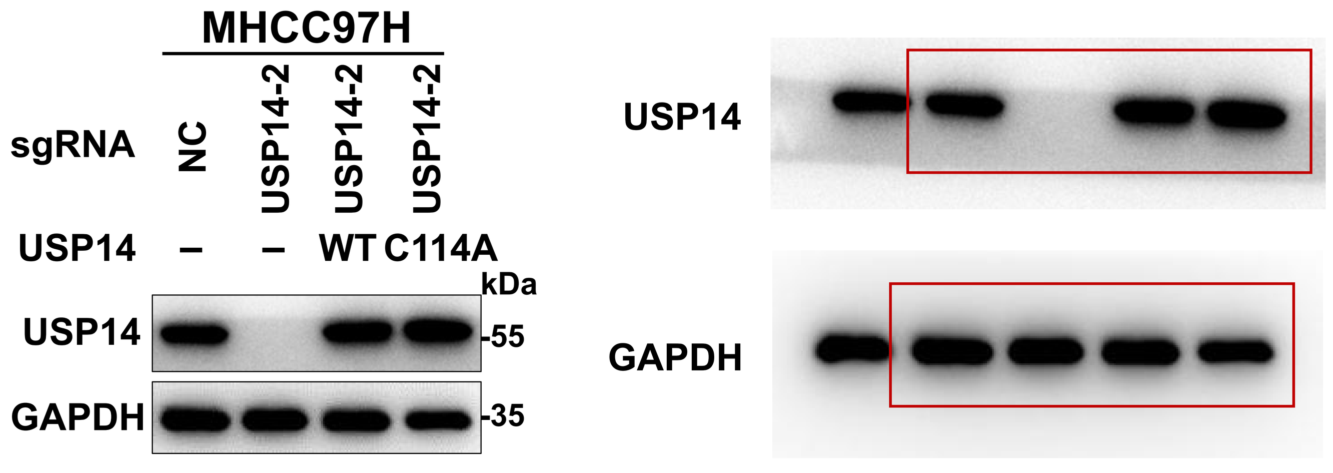

Figure S8A

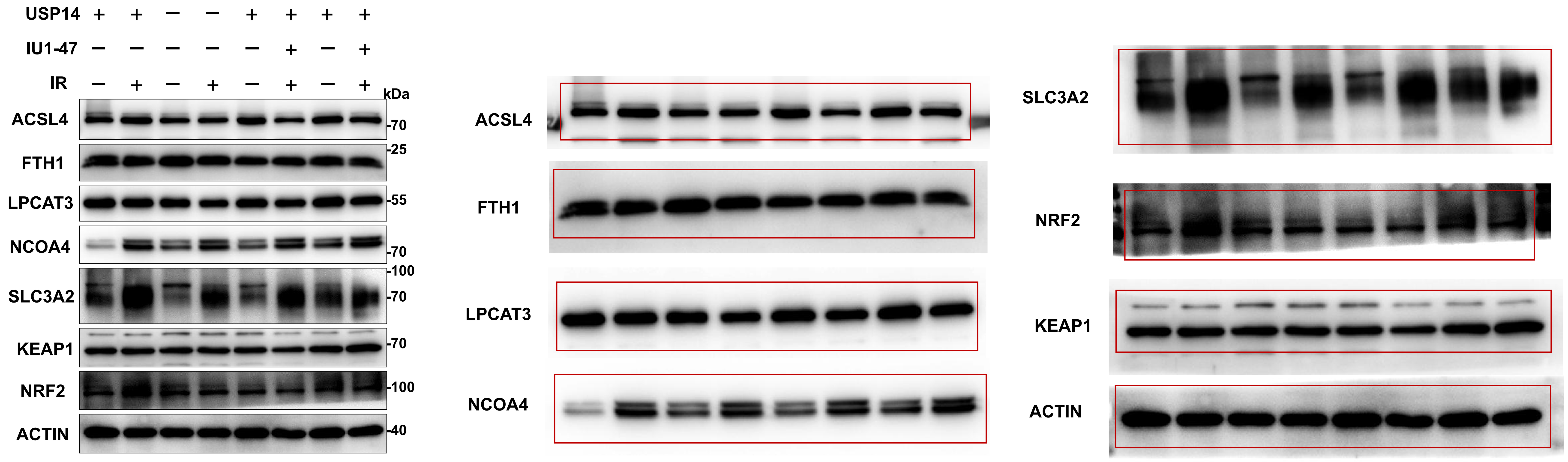

Figure S8B

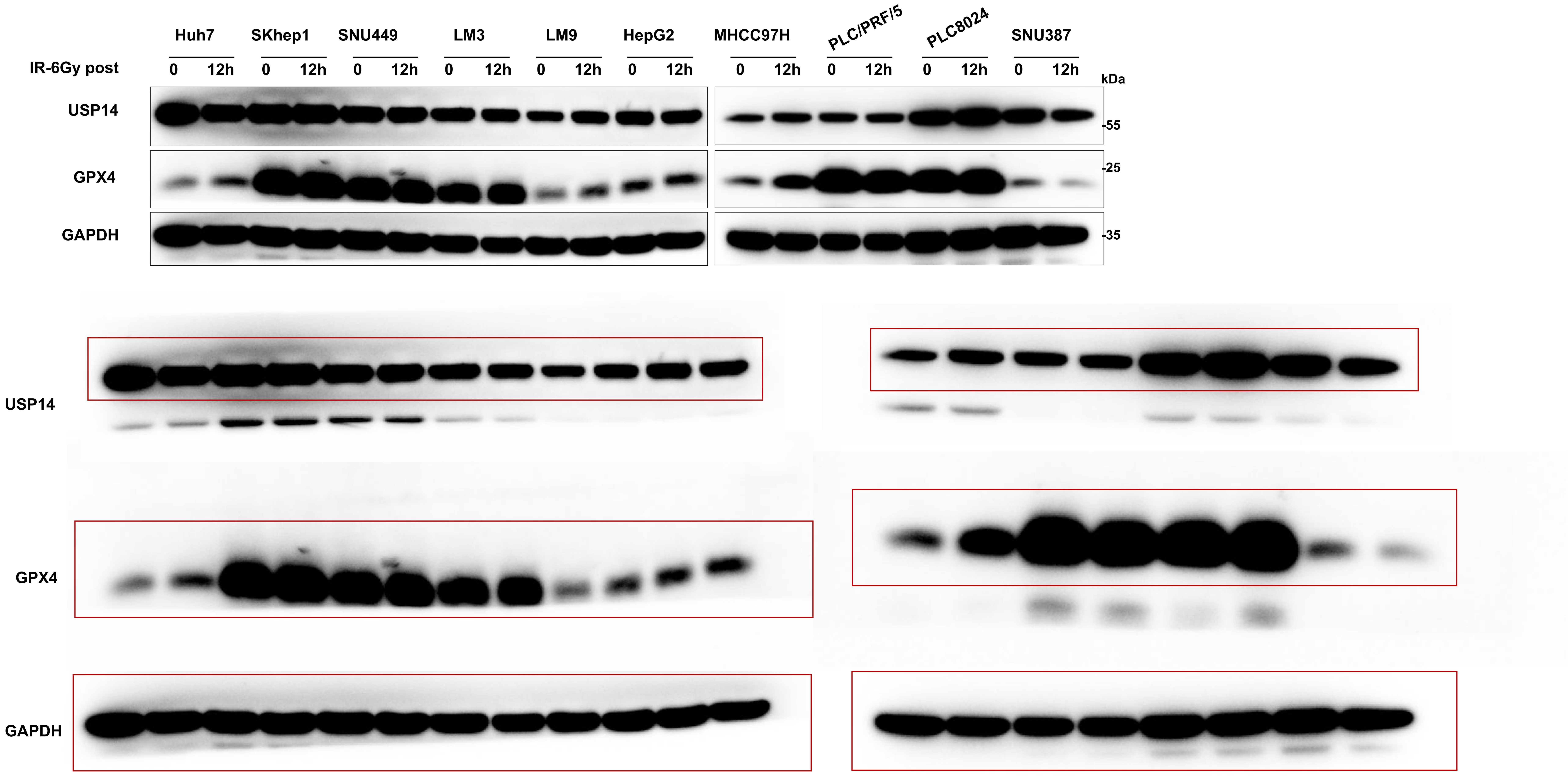

Figure S8C

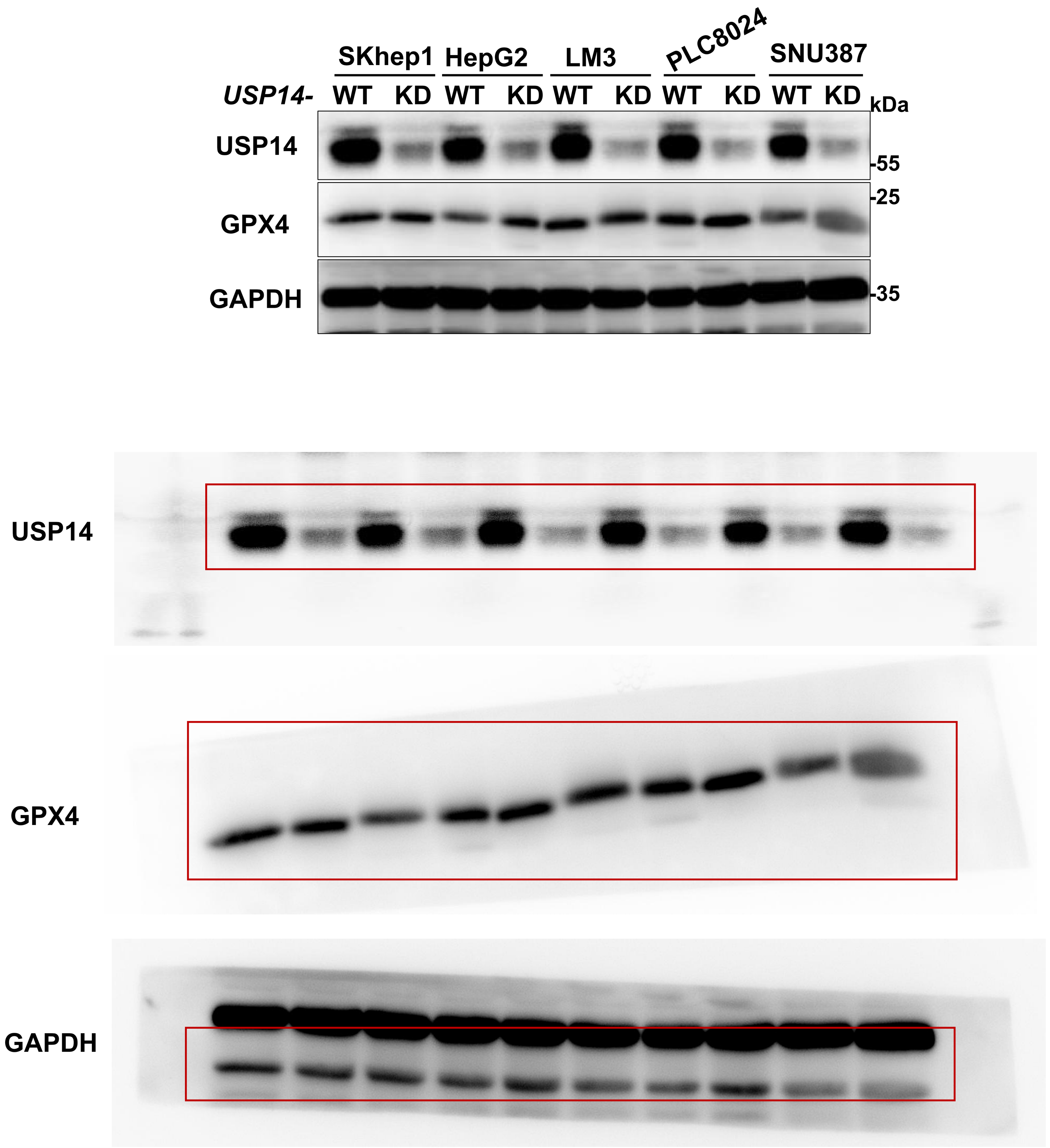

Figure S8D

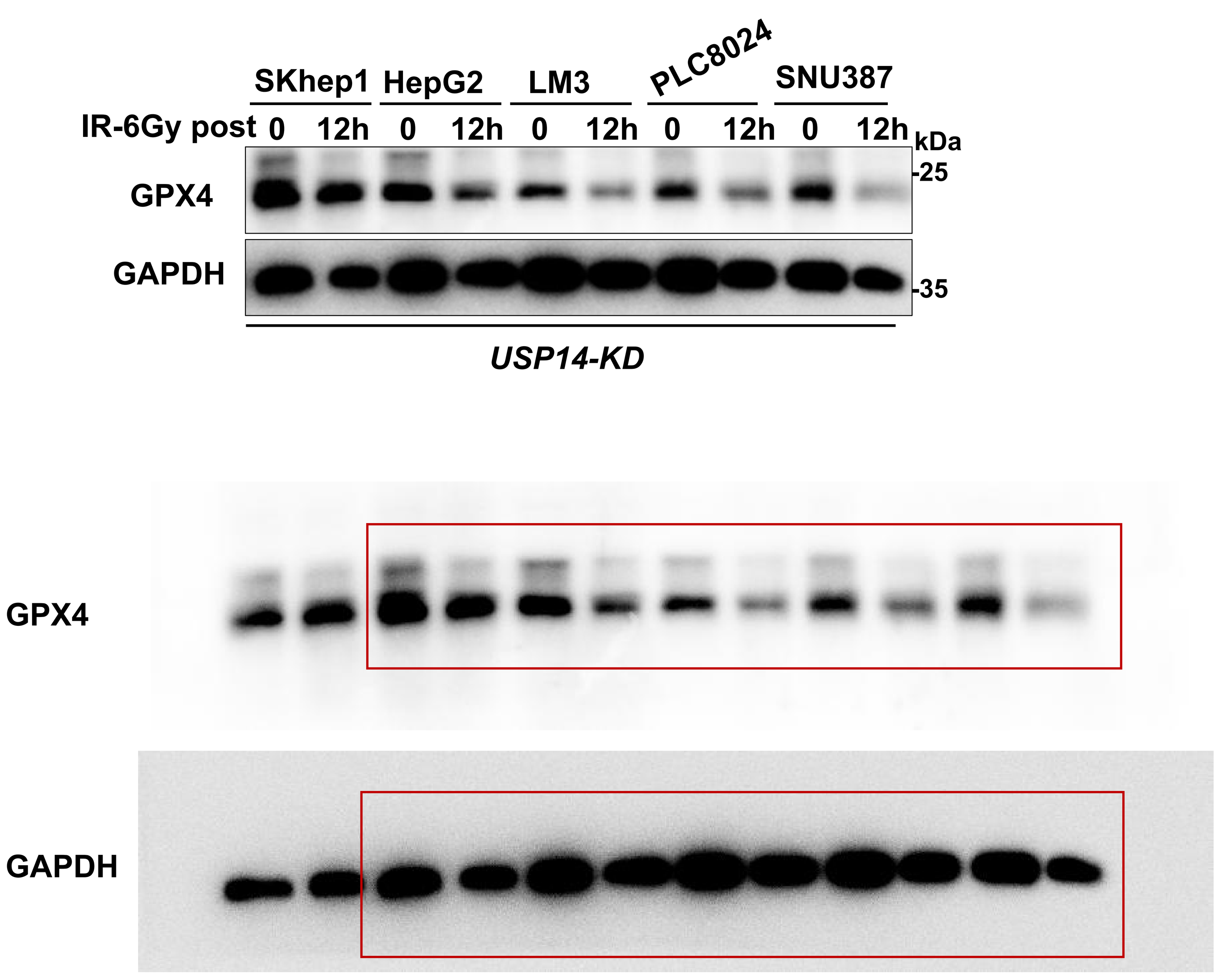

Figure S9C

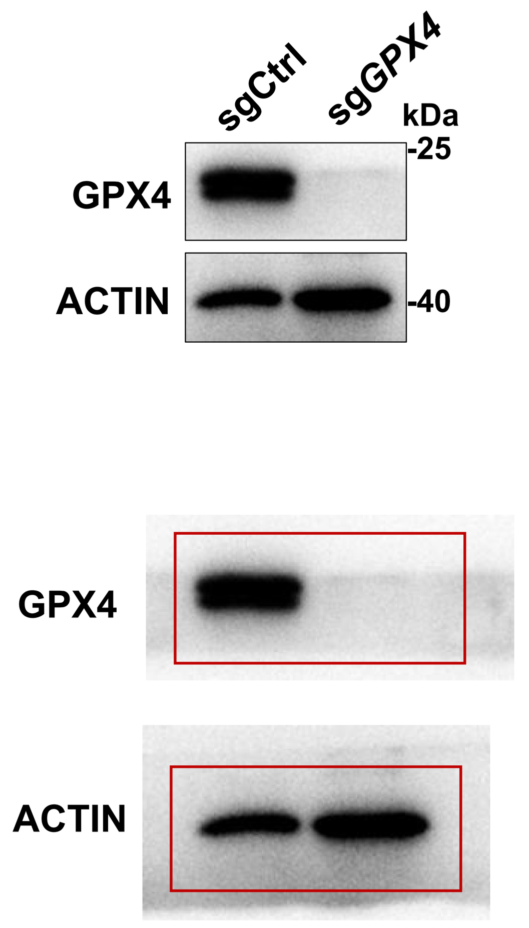

Figure S11A

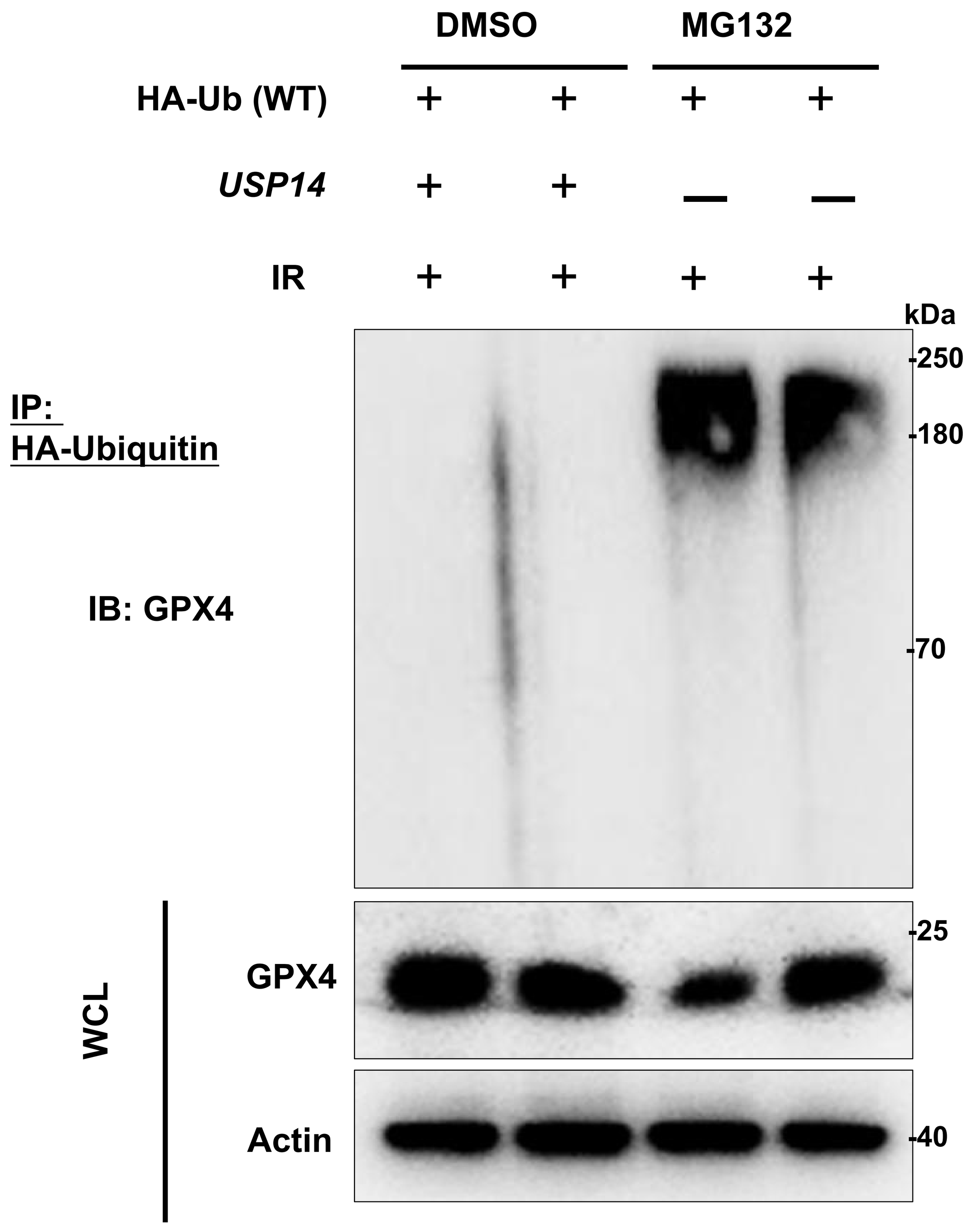

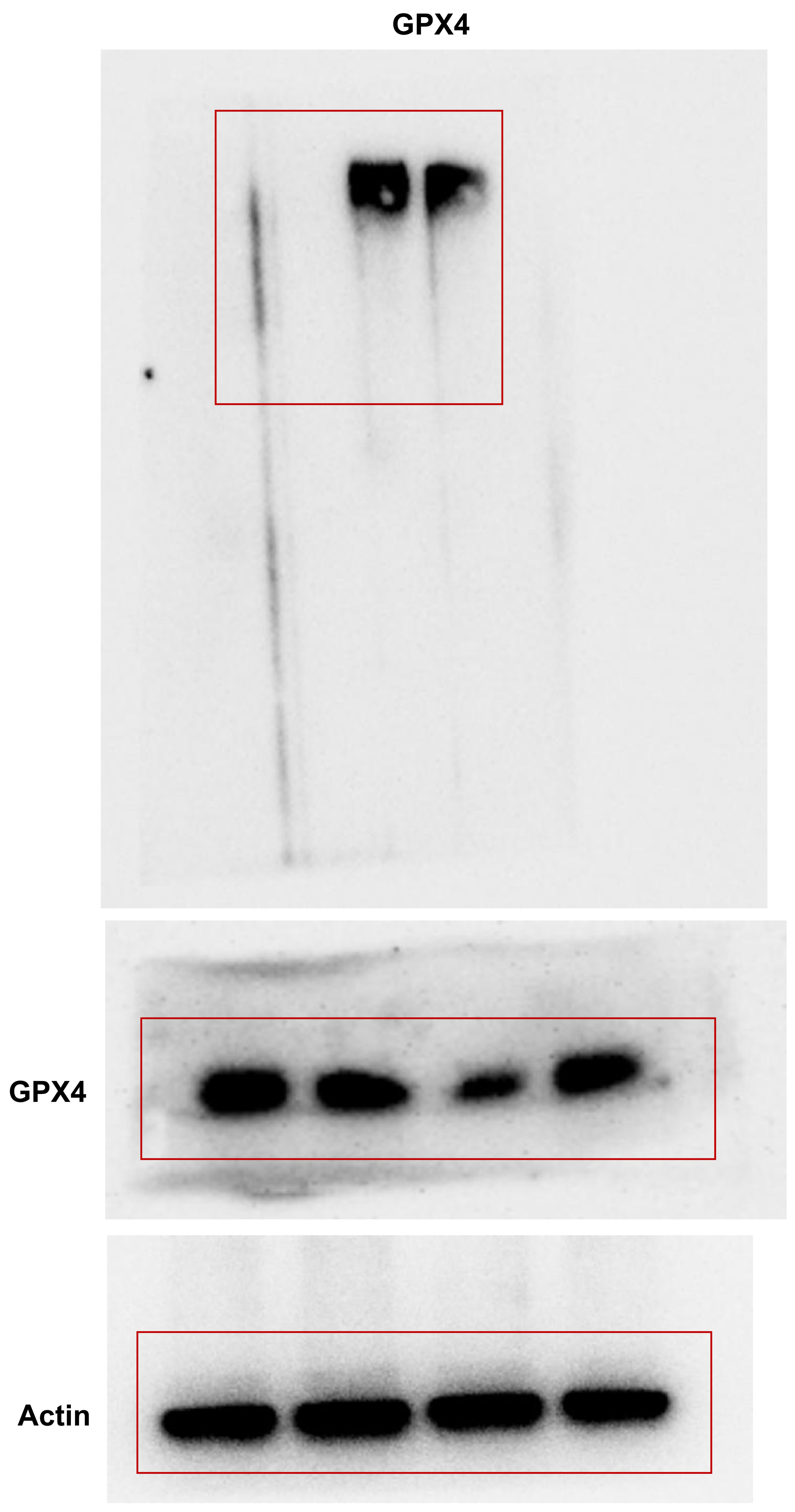

Figure S11B

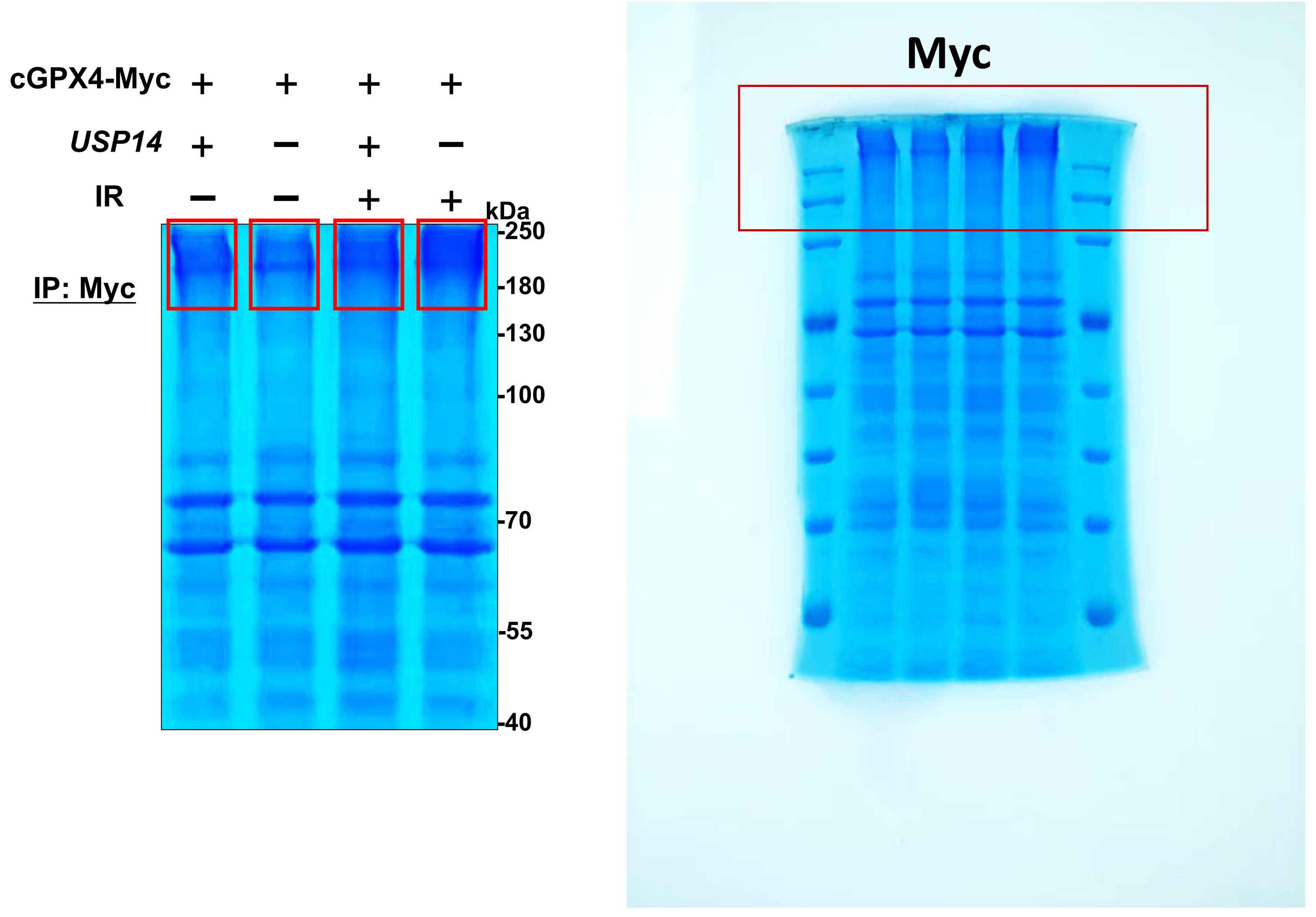

Figure S11C

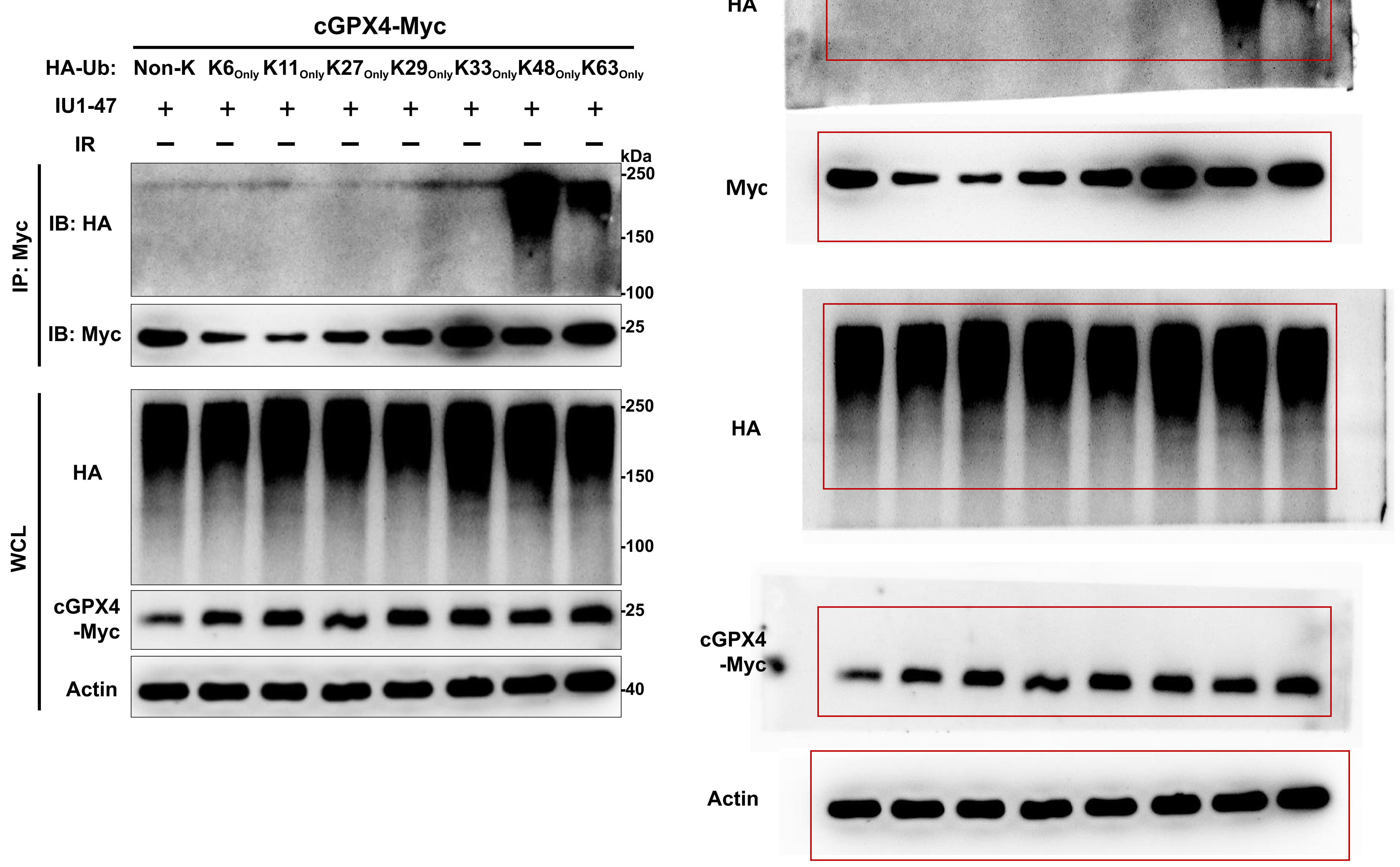

Figure S11D

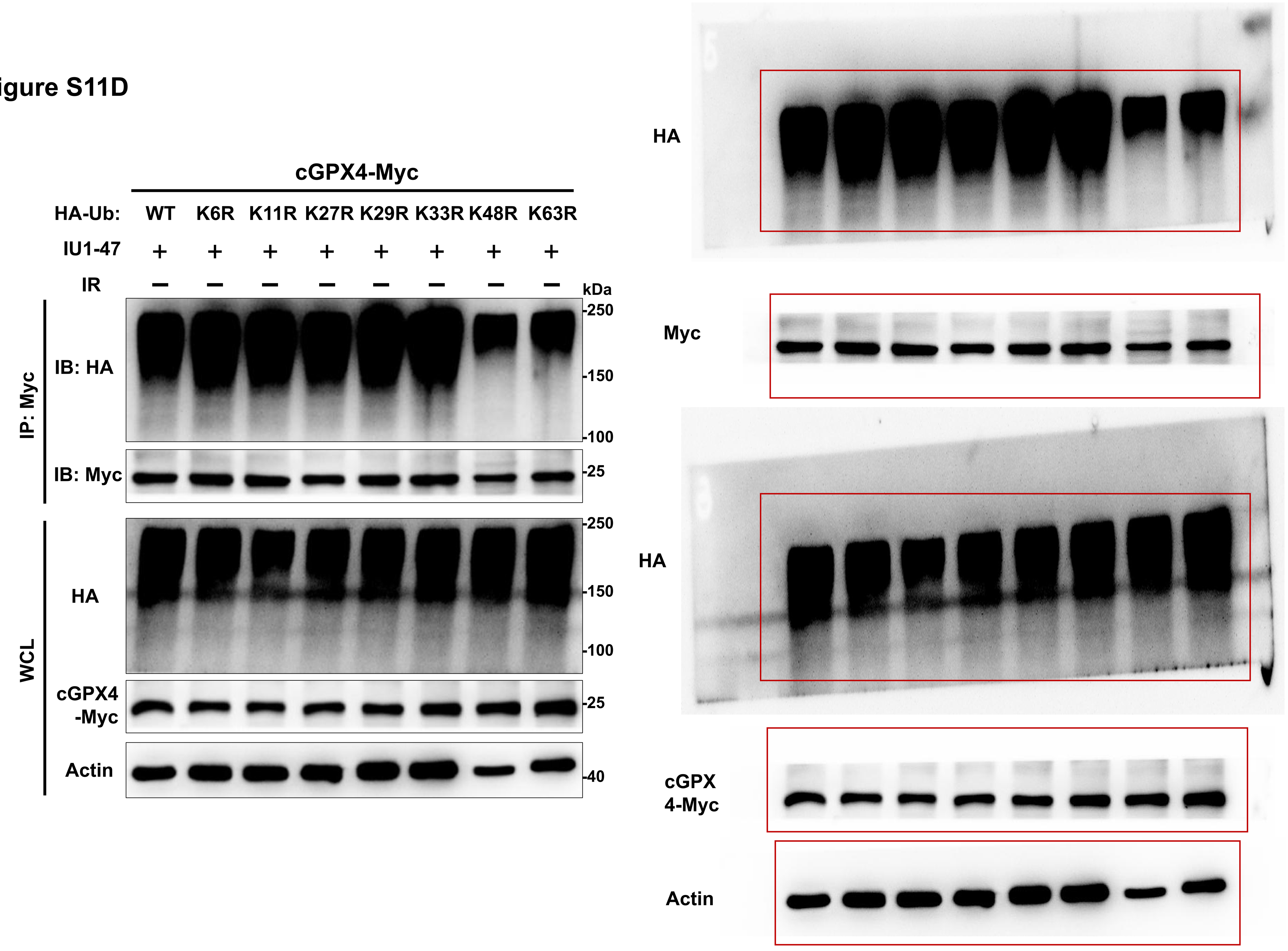

Figure S12A

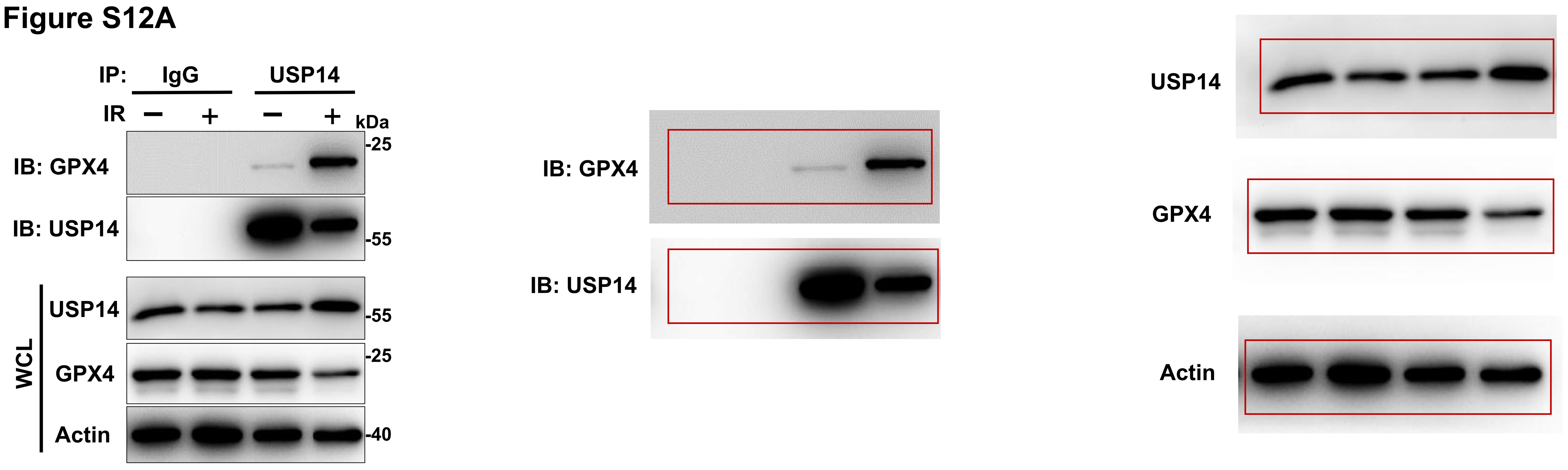

Figure S12B

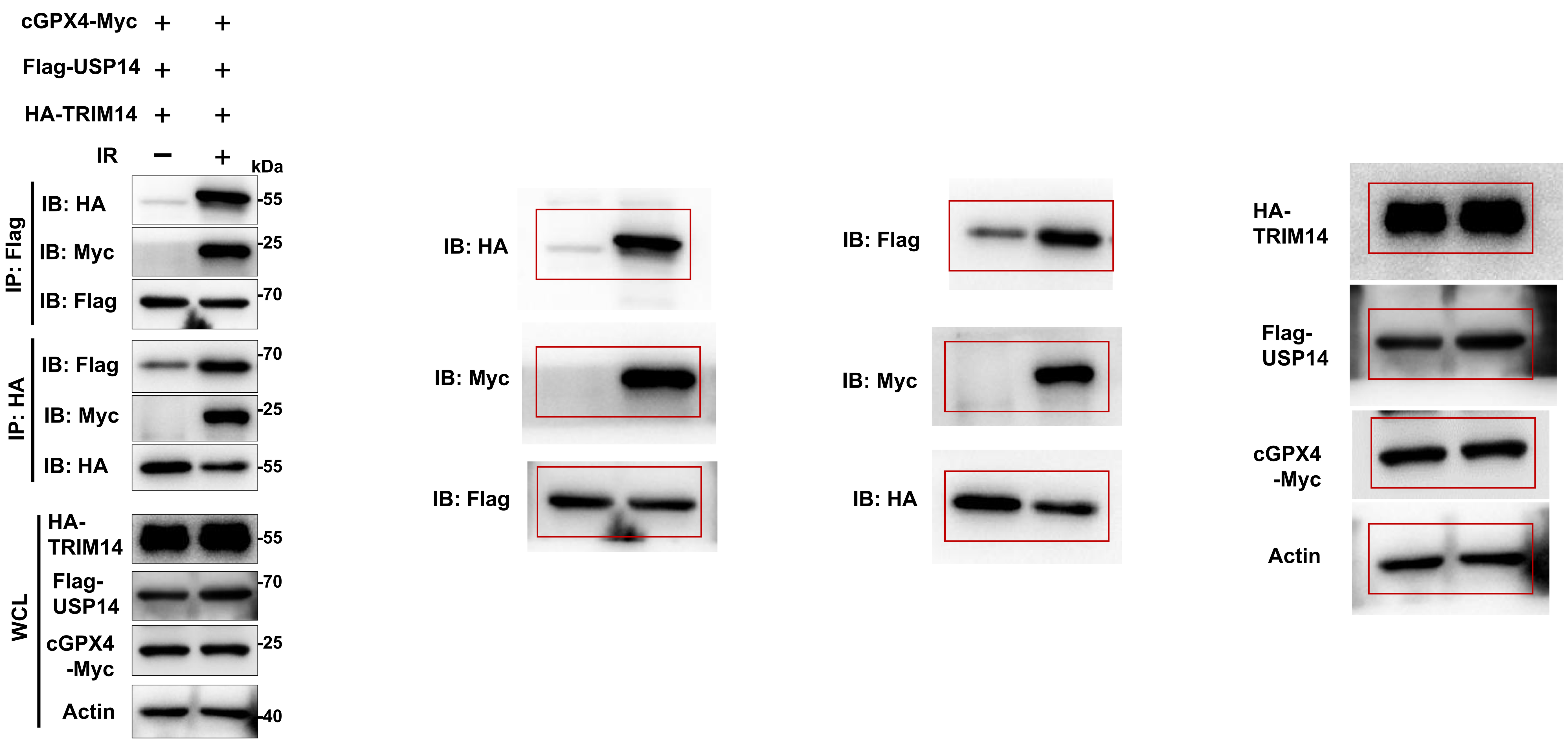

Figure S12C

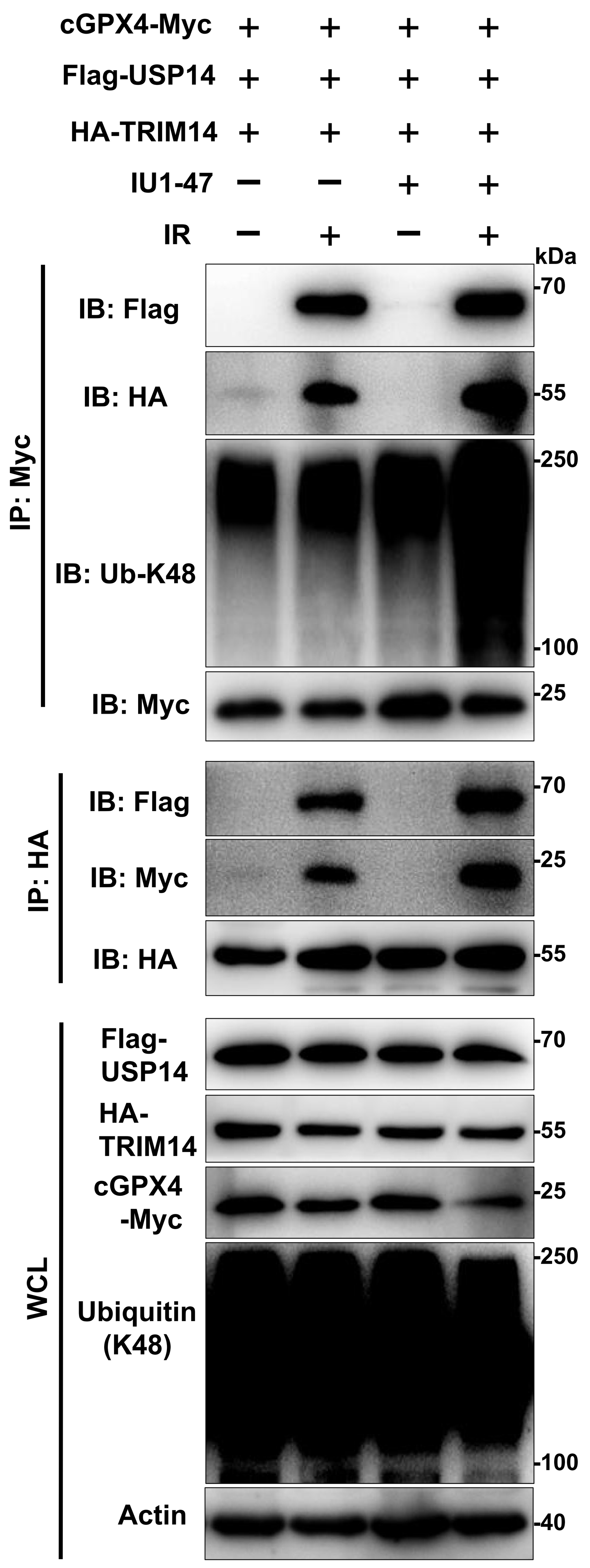

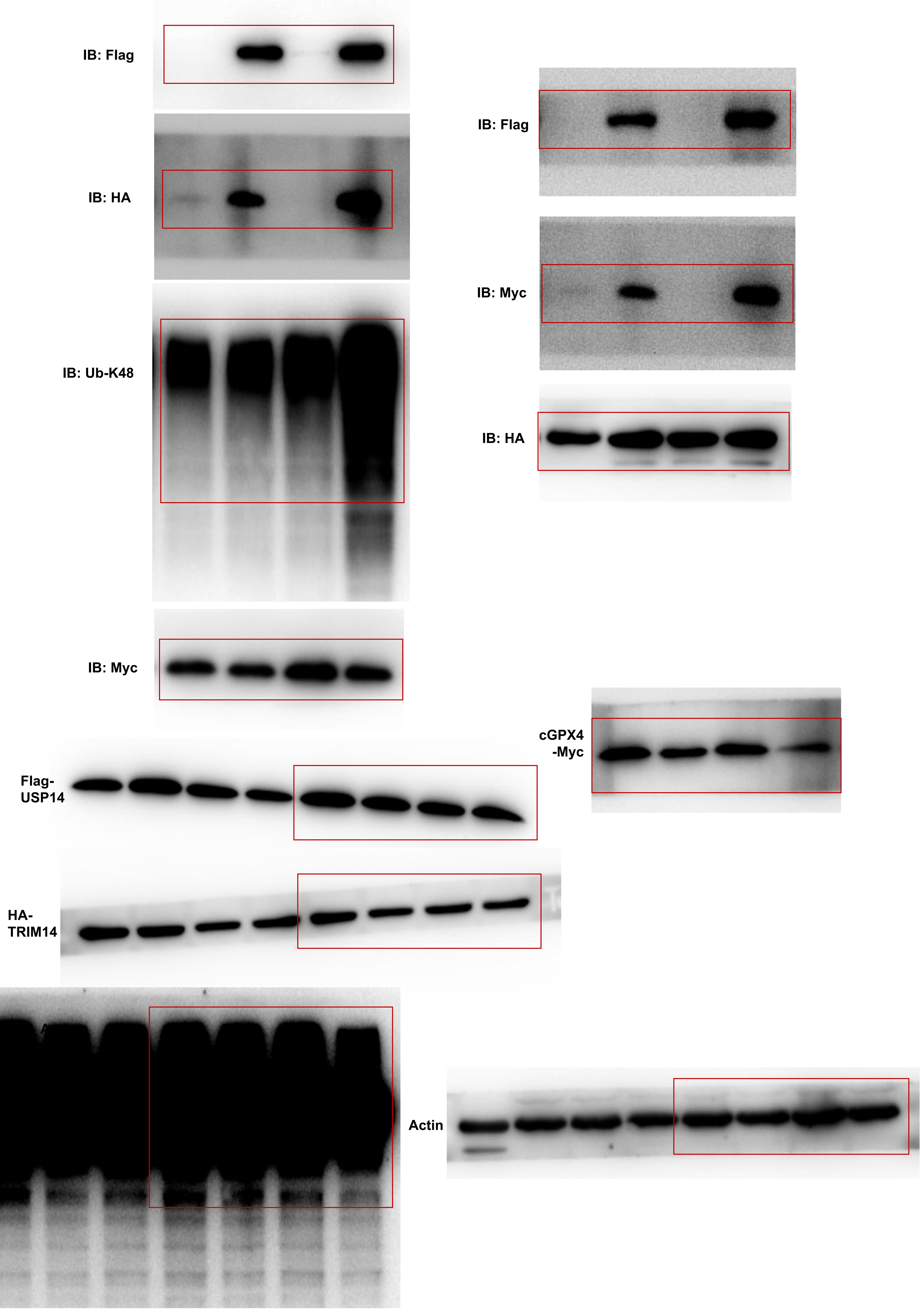

Figure S14A

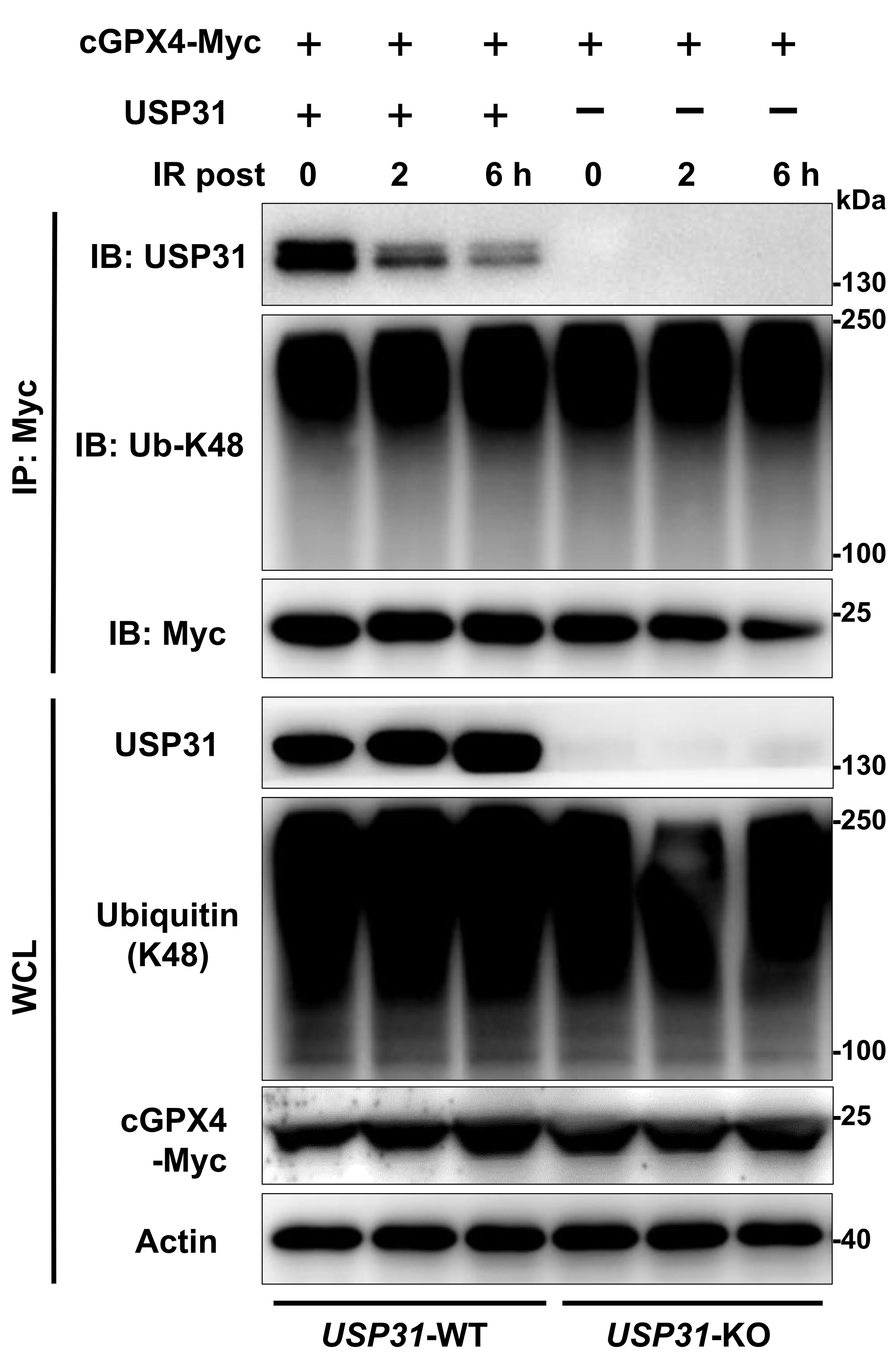

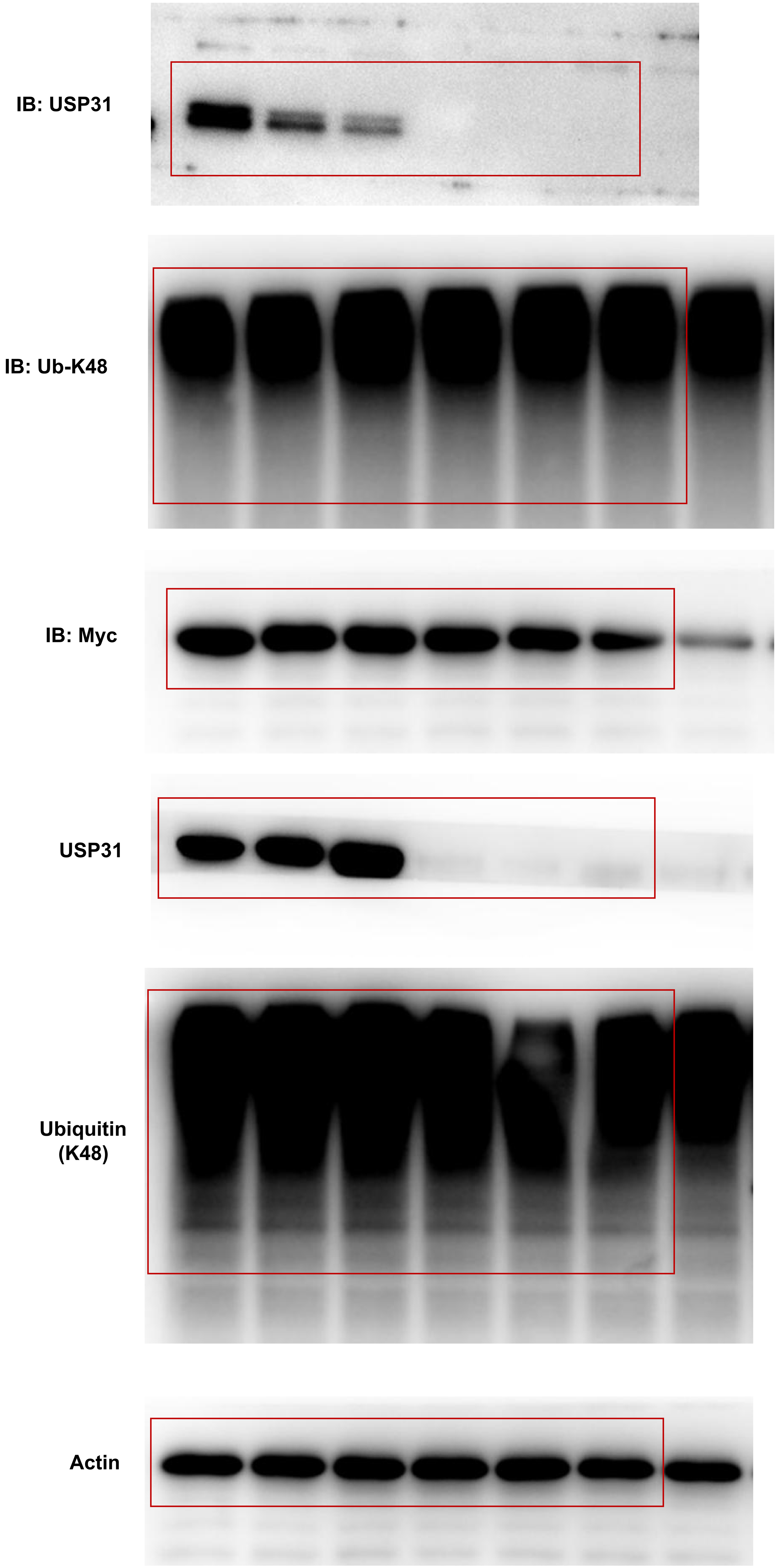

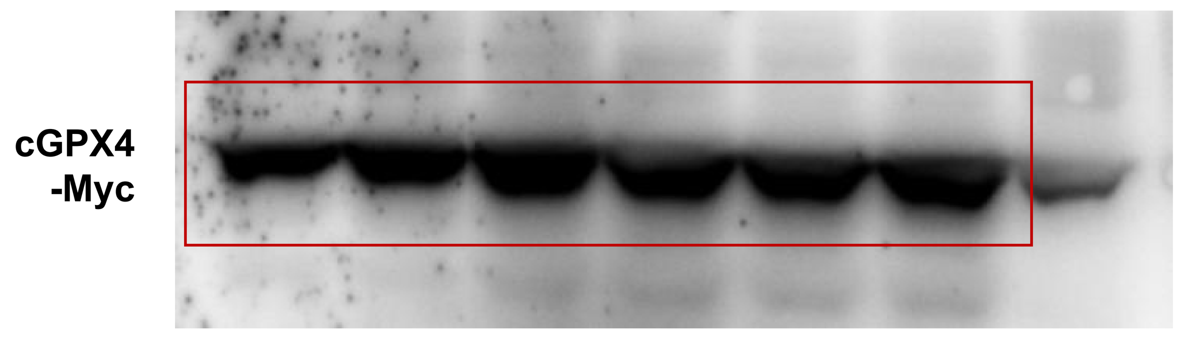

Figure S14C

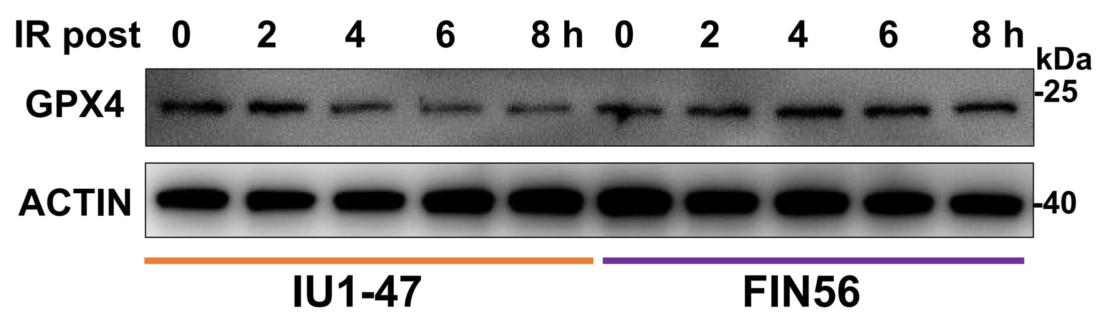

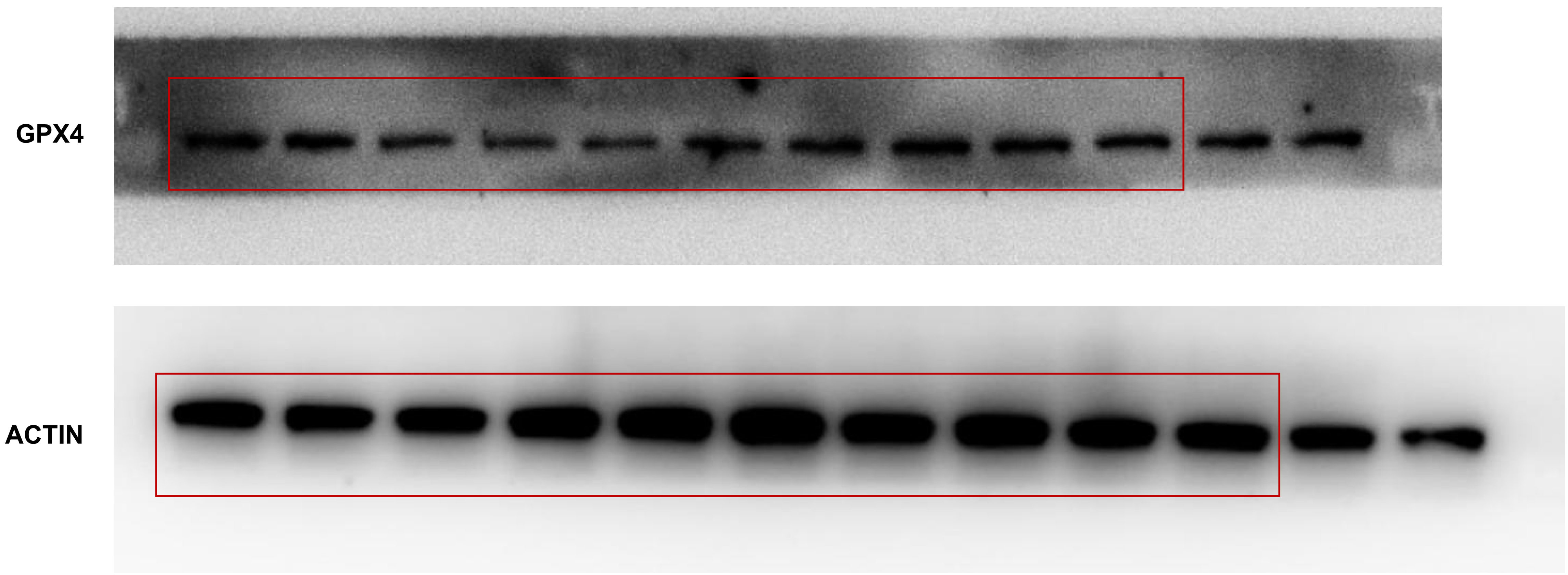

Supplement: Supplementary file 2 — Uncut WB Blot image [file 41419_2025_7807_MOESM2_ESM.pdf]
